# Engineering an Artificial Myxopyronin Derivative with Enhanced Metabolic Stability via Mutasynthesis

**DOI:** 10.1021/jacsau.5c00950

**Published:** 2025-11-18

**Authors:** Alexander F. Kiefer, Alexander Voltz, Domen Scherzer, Roman Reberšek, Andreas M. Kany, Gareth Prosser, Markus Neuber, Norbert Reiling, Anna K. H. Hirsch, Rolf Müller

**Affiliations:** † Helmholtz Institute for Pharmaceutical Research Saarland (HIPS), Campus E8.1, 66123 Saarbrücken, Germany; # Helmholtz Centre for Infection Research (HZI), Inhoffenstraße 7, 38124, Braunschweig, Germany; ‡ German Center for Infection Research (DZIF), Inhoffenstraße 7, 38124, Braunschweig, Germany; § PharmaScienceHub (PSH), Campus A2.3, 66123, Saarbrücken, Germany; ∥ Saarland University, Department of Pharmacy, Campus E8.1, 66123, Saarbrücken, Germany; ⊥ Microbial Interface Biology, Research Center Borstel, 28413 Leibniz Lung Center, Parkallee 1, 23845 Borstel, Germany

**Keywords:** Myxobacteria, myxopyronin, α-pyrone antibiotics, mutasynthesis, RNA polymerase (RNAP)

## Abstract

The rise of multidrug-resistant pathogens, such as *Staphylococcus aureus* and *Mycobacterium tuberculosis*, underscores an urgent need for therapeutic innovation. The antibiotic development pipeline targeting these bacteria is critically limited, with most discovered candidates exhibiting structurally similar features of prominent chemical entities and with well-established molecular targets or binding modes. The myxobacterial α-pyrone antibiotics, myxopyronins, represent a highly promising compound class due to their ability to inhibit RNA polymerase by binding to the “switch region”, a distinct binding site to that of standard-of-care antibiotics. Mutasynthesis, leveraging engineered microorganisms and tailored precursors, provides a viable alternative to total synthesis for generating novel derivatives. This study utilized a heterologous expression system in *Myxococcus xanthus* DK1622 to generate analogs. Two carrier protein domain mutants were engineered to facilitate mutasynthesis-based production of structurally diverse derivatives. A trifluoromethyl-modified analog, once accessible only through total synthesis but now obtained via mutasynthesis, exhibits potent antimicrobial activity against Gram-positive pathogens including *Mycobacterium tuberculosis* and favorable in vitro absorption, distribution, metabolism, excretion and toxicity properties. These findings highlight a promising pathway for developing optimized α-pyrone antibiotics to address the global antimicrobial-resistance crisis.

## Graphical abstract

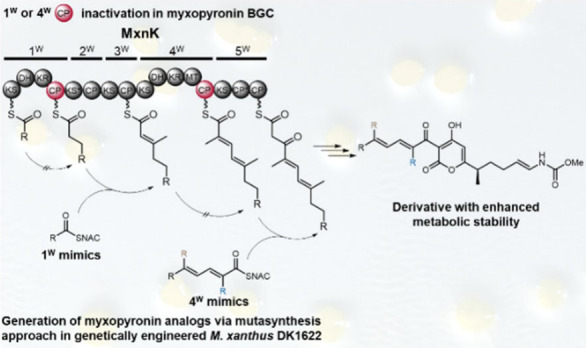

## Introduction

The rapid emergence of antimicrobial resistance (AMR) to clinically applied antibiotics is a critical global health threat, posing severe challenges to healthcare systems.^1^ In particular, multidrug resistant (MDR) pathogens, such as *Staphylococcus aureus* (*S. aureus*), or *Mycobacterium tuberculosis* (*M. tuberculosis*) have been classified as high to critical priorities by the World Health Organization due to the limited treatment options.^2,3^ The majority of antibiotics in the development pipeline are derivatives of defined chemical entities, with well-established molecular targets and binding modes.^4^ This approach often facilitates the rapid development of cross-resistance, as bacterial pathogens may have already evolved resistance mechanisms against these structurally similar compounds. Thus, prospective antibiotics should either incorporate innovative chemical scaffolds or target novel molecular binding sites.^5^ The α-pyrone antibiotics myxopyronins A (MYX A, **1**) and B (MYX B, **2**), first isolated in the 1980s from the Gram-negative soil bacterium *Myxococcus fulvus* (*M. fulvus*) Mx f50, hold significant potential in meeting this therapeutic need (Figure 1A).^6–8^ These natural products along with the structurally related corallopyronins A (COR A, **3**) and B (COR B, **4**),^9–11^ bind to the “switch region” of the bacterial RNA polymerase (RNAP),^12–14^ a site that is not targeted by standard-of-care antibiotics, making them potential candidates for the development of broad-spectrum antibacterial therapeutic agents.

**Figure 1:**
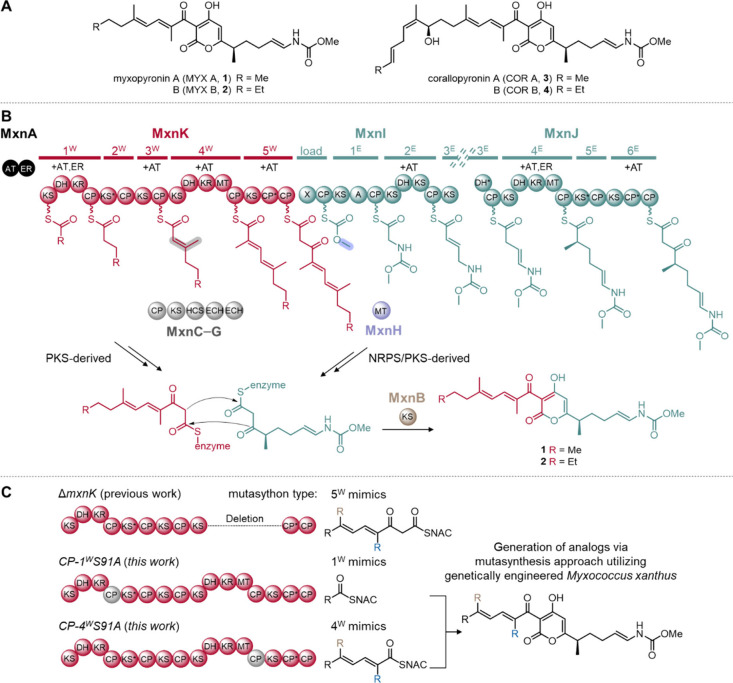
α-Pyrone containing natural products and MYX biosynthesis model based on analyses of the native producer strain Myxococcus fulvus Mx f50. (A) Structures of α-pyrone containing MYX (**1** and **2**) and COR (**3** and **4**). (B) Simplified biosynthetic machinery to assemble **1** and **2**. Western and Eastern chains are biosynthesized in two separate assembly lines. While MxnK is shown in red, MxnI/J is shown in green. Abbreviations for assembly line domains: PKS, polyketide synthase; NRPS, nonribosomal peptide synthetase; A, adenylation domain; CP, carrier protein domain; DH, dehydratase domain; KR, ketoreductase domain; KS, ketosynthase domain; MT, methyltransferase domain; X, putative inactive KR or truncated phosphoglucomutase/phosphomannomutase domain. The β-branching cassette for module 3^W^ intermediate is shown in gray, comprising of CP MxnC, KS MxnD, hydroxymethylglutaryl-CoA synthase (HCS) MxnE and two enoyl-CoA hydratases/isomerases (ECH) MxnF and MxnG. The Eastern chain starter unit is modified by an O-methyltransferase (MT; MxnH) shown in blue. Domains marked with an asterisk (*) are considered inactive. Modules 2W, 3E, and 5E in the assembly line are presumed not to participate in chain elongation. Required acyltransferase (AT) and enoylreductase (ER) activities are indicated for each module and supplied in trans by MxnA. After chain assembly, the KS MxnB shown in brown, catalyzes the α-pyrone ring formation. (C) Mutasynthesis approach for the generation of novel derivatives. Site-directed mutagenesis of mxnK by in-frame deletion in M. fulvus Mx f50 (previous work). Site-directed mutagenesis to inactivate the carrier protein domains from modules 1 and 4 (CP-1^W^S91A or CP-4^W^S91A) in overexpression mutant strain M. xanthus DK1622 (this work).

Although total synthesis has enabled access to the natural products (NPs) MYXs (**1** and **2**) and their derivatives, this strategy faces significant limitations.^15–24^ It often involves a high number of reaction steps, the use of costly or noncommercially available reagents, and frequently results in low yields or poor selectivity. In contrast, mutasynthesis offers an alternative hybrid approach that merges the synthesis and feeding of simple synthetic precursors, known as mutasynthons, with the utilization of genetically engineered microorganisms.^25^ Understanding the biosynthetic gene cluster responsible for NP synthesis allows for targeted mutations in the biosynthetic machinery, which inhibit NP production at a stage that can be complemented by addition of mutasynthons. Furthermore, this approach enables the generation of derivatives by introducing artificial precursors of biosynthetic intermediates.

The identification and characterization of the MYX biosynthetic pathway revealed that **1** and **2** are derived from two distinct multimodular polyketide (and peptide) assembly lines, referred to as the Western (red) and Eastern (green) chains of the molecule (Figure 1B).^26^ Furthermore, the assembly lines are part of *trans*-acyltransferase (*trans*-AT) polyketide synthase (PKS) systems, characterized by the absence of integrated AT domains.^26,27^ Instead, these systems rely on discrete, free-standing AT activities, exemplified by MxnA. In addition to its specificity for malonyl-coenzyme A as a substrate, MxnA exhibits enoylreductase (ER) activity, a function notably absent in the modular subunits of traditional PKS systems. The diene-containing Western chain is synthesized by the PKS MxnK, while the carbamate-containing Eastern chain is produced by the hybrid PKS/nonribosomal peptide synthetase (NRPS) system comprising MxnI and MxnJ. These chains undergo intramolecular macrocyclization, catalyzed by the stand-alone ketosynthase (KS) enzyme MxnB, resulting in the formation of the α-pyrone ring.

In an early mutasynthesis attempt, *mxnK* was inactivated, leading to the complete abolition of western chain biosynthesis.^28^ Subsequent feeding experiments in small-scale cultures confirmed successful restoration of NP production. However, isolation of derivatives was unsuccessful due to low production yields, likely attributed to insufficient uptake or the rapid degradation of the β-keto *N*-acetylcysteamine thioesters (β-keto SNACs) utilized in the process (Figure 1C).

The recently developed heterologous expression system *Myxococcus xanthus* (*M. xanthus*) DK1622*ΔmchA-tet*, yields **1** at titers exceeding 150 mg/L, thereby paving the way for the development of novel α-pyrone antibiotic analogs with enhanced pharmaceutical properties.^29,30^ *M. xanthus* DK1622*ΔmchA-tet* is a mutant in which the myxochromide A gene cluster has been deleted. Myxochromide A is one of the major compounds produced by *M. xanthus* DK1622, and its gene cluster deletion was expected to provide an increased precursor pool for production of other secondary metabolites. In brief, the myxochromide A gene cluster was completely deleted from *M. xanthus* DK1622 and replaced by a tetracycline resistance gene, which served as the target site for the chromosomal integration of the MYX expression construct.

In this study, we report the use of the heterologous expression system of compound **1** in *M. xanthus* DK1622*ΔmchA-tet* to generate two mutants via site-directed mutagenesis (Figure 1C). These mutations were designed to inactivate the carrier protein (CP) domains in Western chain modules one and four, establishing the groundwork for an advanced mutasynthesis-based strategy aimed at producing novel MYX derivatives. Analysis of the RNAP binding site of NP **1** facilitated the structure-guided design and synthesis of a diverse array of mutasynthons to assess the impact on bioactivity and in vitro absorption, distribution, metabolism, excretion and toxicity ADMET properties of the obtained derivatives. Additionally, the restoration of NP biosynthesis in the engineered mutants, along with an evaluation of substrate acceptance, was systematically investigated. Optimization of production conditions, followed by isolation and structure elucidation, led to a trifluoromethyl analog of **1**.^23^ The trifluoromethyl analog of **1** was initially only accessible via total synthesis as demonstrated by the Ebright group.^23^ Further assessment of its on-target activity against several bacterial RNAPs, growth inhibition of Gram-positive as well as Gram-negative bacterial strains and elaboration of the metabolic stability in human liver microsomes,^23^ prompted us to expand the compounds behavior comprehensively, including the antimicrobial activity against serval clinical significant Gram-positive pathogens including *M. tuberculosis* and further ADMET profiling.

## Results and Discussion

### Analog Design, Generation of Mutant Strains and Substrate Synthesis

Bacterial DNA-dependent RNAP is a multisubunit enzyme composed of a core of five subunits (α_2_ββ’ω), which plays a central role in bacterial transcription.^31^ These subunits are highly conserved across bacterial species but are distinct from the eukaryotic RNAP I–III. The “switch region” is part of the β and β′ subunits, facilitating conformational changes in the RNAP enzyme, enabling the loading of DNA into the active center cleft during transcription initiation. The α-pyrone antibiotic **1** is interacting with the “switch region” by adopting a U-shaped conformation in the β and β’ subunits, leading to an inactive promoter complex.^13^ While, previous structure–activity relationship (SAR) studies have primarily focused on modifications of the Eastern chain of the compound, revealing a significant loss in activity with even slight alterations, we focused on exploring modifications in the Western portion of the molecule.^15^ To design novel derivatives of **1**, we used the cocrystal structure of the “switch region” of *Thermus thermophilus* RNAP as a blueprint (Figure 2A). Mapping the molecular lipophilicity potential (MLP) of the binding site surface revealed the predominance of nonpolar regions, indicating the necessity for introducing primarily hydrophobic modifications to optimize interactions. We intended to modify the methylation pattern of the diene to investigate the minimum structural requirements necessary for condensation reactions during biosynthesis and sustaining biological activity (**5**–**7**) (Figure 2B). Given that MYX B (**2**) has a very short half-life of only 3 min in human liver microsomes,^23^ we selected a range of substituents, including trifluoromethyl (**8**) previously reported by Ebright et al.,^23^ propargyl (**9**) and various five- and six-membered (hetero)­cycles (**10**–**19**), to assess their impact on the ADMET properties of exemplified derivatives.

**Figure 2:**
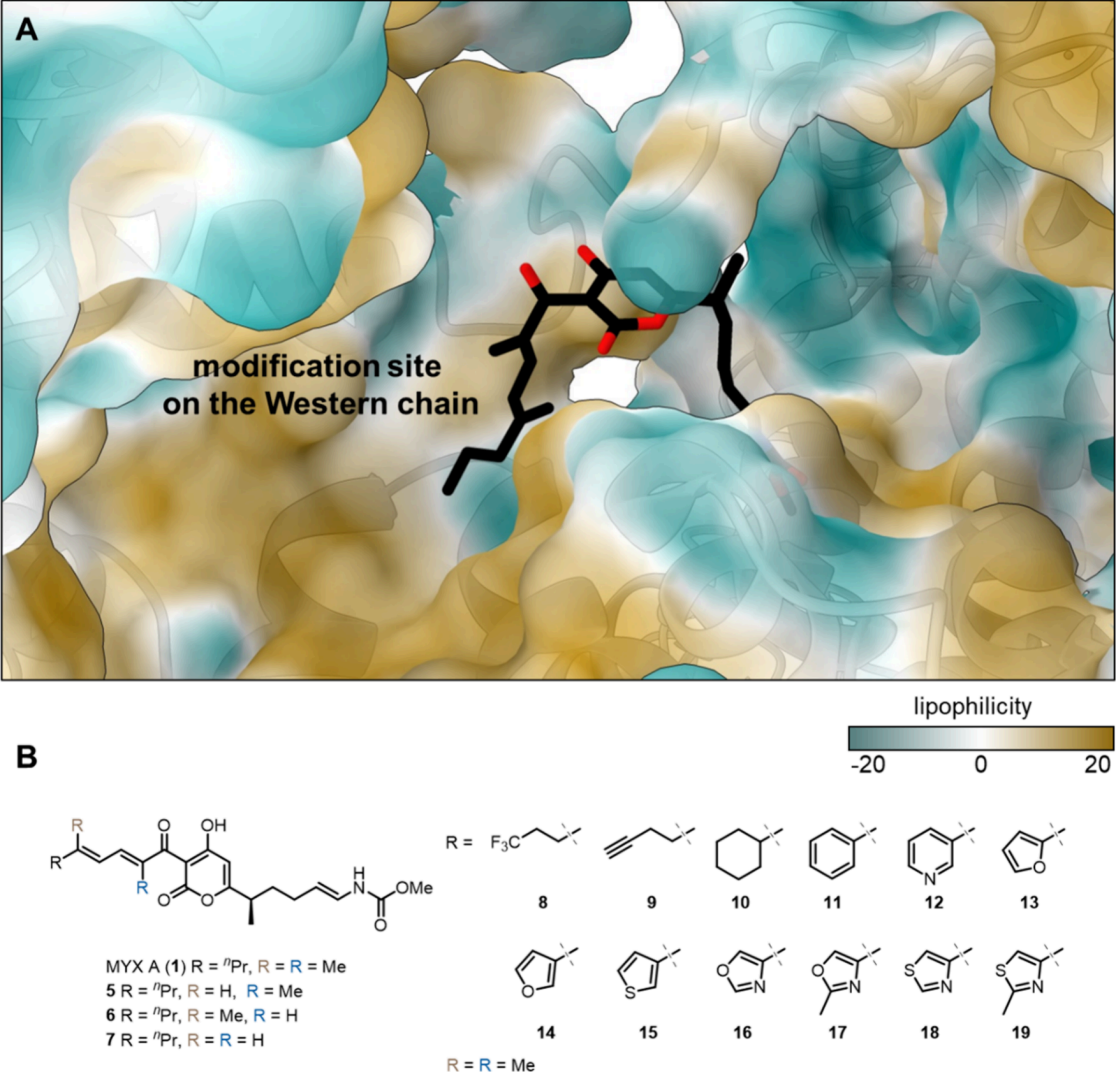
(A) Cocrystal structure of the “switch region” of *Thermus thermophilus* RNAP in complex with **1** (PDB ID: 3DXJ). Molecular lipophilicity potential (MLP) map with coloring on the molecular surface ranging from dark cyan (most hydrophilic) to white to dark goldenrod (most lipophilic). The image was generated using ChimeraX software. (B) Structures of MYX A (**1**) and planned artificial derivatives (**5**–**19**).

Docking simulations of analogs (**5**–**7**) featuring alternate methylation patterns did not reveal a significant impact on their calculated binding affinity to the RNAP “switch region”, as evidenced by their calculated energy scores when compared to MYX A (**1**) and MYX B (**2**) (Table S1). However, substitution of the terminal methyl group with a trifluoromethyl (**8**) or propargyl group (**9**) resulted in improved predicted binding affinity, as indicated by lower calculated energy scores. Additionally, modeled derivatives (**10**–**19**) incorporating five- or six-membered ring systems exhibited consistently lower energy scores relative to the unmodified NPs (**1** and **2**). Among these, the cyclohexane-modified derivative (**10**) showed the lowest energy score, followed by the phenyl-substituted variant (**11**). These findings suggest that the RNAP “switch region” can accommodate bulkier substituents near the terminal region of the Western chain, offering potential for further structural optimization.

In an initial mutasynthesis attempt, supplementation with modified β-keto SNACs in the Δ*mxnK* mutant strain of the wild-type producer *M. fulvus* Mx f50 did not yield novel derivatives. To improve the chances of generating new compounds, we opted to employ the heterologous host *M. xanthus* DK1622 *ΔmchA-tet:*:pHSU-mxn43 as this strain had previously demonstrated significantly enhanced NP production. Furthermore, to overcome the instability of β-keto SNACs, we aimed to introduce mutations at an earlier stage of Western chain biosynthesis. This approach would enable feeding experiments with simpler and more stable mutasynthons, potentially increasing the efficiency of derivative production. To enable mutasynthesis in the heterologous producer, we performed site-directed mutagenesis to introduce a serine-to-alanine substitution at the CP active site in the first and fourth modules of the myxopyronin MxnK megasynthase. To achieve this two synthetic DNA fragments were chemically synthesized with the respective single-nucleotide substitutions. These fragments were flanked by homology regions for recombination and included a chloramphenicol resistance gene for selection, imbedded by restriction sites for later removal. Following recombining in *E. coli*, mutated CP domains were inserted to replace native sequences in the *mxnK* gene. This resulted in the generation of the *M. xanthus* DK1622 Δ*mchA-tet*::pHSU-mxn43-ACP1 (*CP-1* ^*W*^ *S91A*) and *M. xanthus* DK1622 Δ*mchA-tet*::pHSU-mxn43-ACP4 (*CP-4* ^*W*^ *S91A*) mutant strains, respectively (Table S2–S4, Figure S1).

To generate a small library of mutasynthons suitable for the supplementation of the starter unit in the *CP-1* ^*W*^ *S91A* mutant strain, native mimics **20** and **21**, along with artificial mimics (**22**–**33**), were synthesized as SNAC esters (Figure 3).^32^ This synthesis was achieved either through direct coupling using acid chlorides (Condition A) or via Steglich esterification of the corresponding carboxylic acids (Condition B). It has been previously reported that in certain cases, thiophenols^33,34^ and even full-length pantetheine intermediates^35,36^ are more efficiently accepted by the PKS machinery compared to SNAC esters. To assess the integration efficiency of these compounds in our system, we synthesized thiophenols (**34**–**36**), as well as pantetheine derivatives (**37**–**39**) to supplement them into the culture medium for further evaluation.

**Figure 3:**
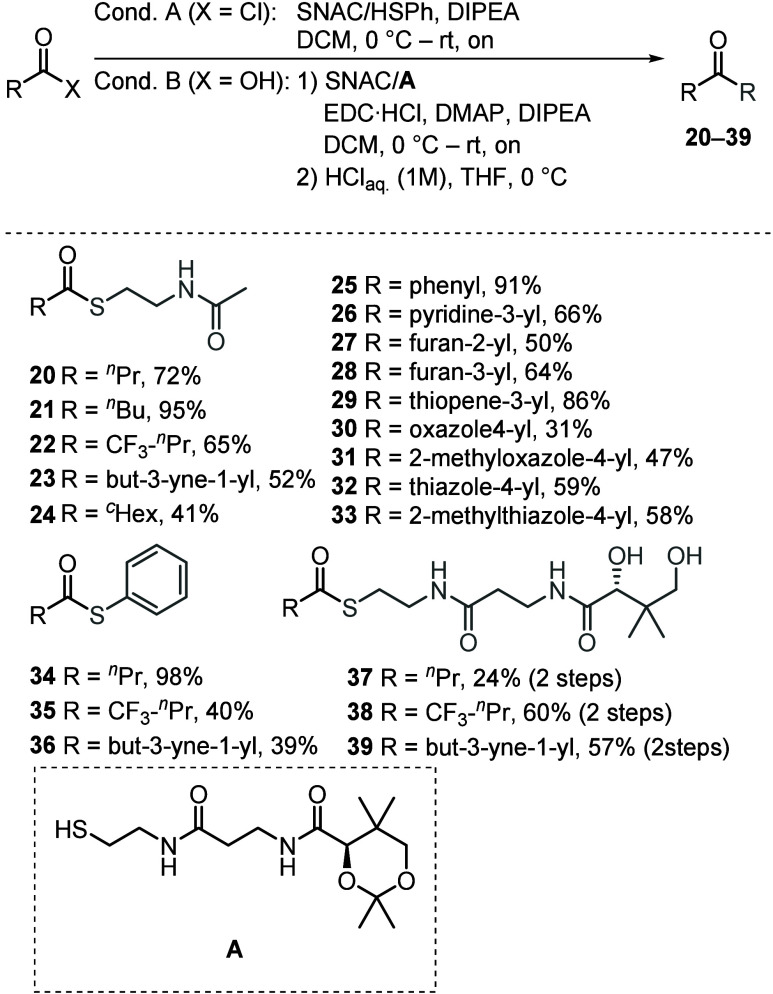
Synthesis of mutasynthons (**20**– **39**) for CP-1^W^S91A mutant supplementation. Formation was achieved either through direct coupling using acid chlorides (Condition A) or Steglich esterification of the corresponding carboxylic acids (Condition B).

Building on Rentsch and Kalesse’s work on the total synthesis of **2**, we synthesized a targeted set of mutasynthons for in vivo testing with the *CP-4* ^*W*^ *S91A* mutant strain (Figure 4). This set includes both native SNAC precursors **40** and **41**, and modified SNAC esters (**42**–**47**). Additionally, we prepared thiophenol **48** and a pantetheine **49** to evaluate and compare their efficiency with module 1 mimics. To introduce a variety of side chains, we envisioned a carbocupration-mediated *syn*-selective addition to ethyl but-2-ynoate.^37–39^ The conjugate addition of various nucleophiles to ethyl but-2-ynoate proceeded with complete stereocontrol, yielding the corresponding (*E*)-alkenes (**40a**–**44a**) exclusively. The second double bond was introduced via two complementary synthetic approaches. In the first method, the ester group was reduced with diisobutylaluminum hydride (DIBALH) to yield an alcohol intermediate, which subsequently underwent in situ allylic oxidation mediated by manganese dioxide (MnO_2_) and Wittig olefination, leading to the formation of dienoates (**40b**–**44b**).^40^ Alternatively, direct access to ethyl dienoates (**45b**–**47b**) was achieved by using the commercially available α,β-unsaturated aldehyde *trans*-2-hexenal as starting material. For all compounds except diene **44b** (*E*/*E* > 90%), exclusively the *E*/*E*-isomer was detected by NMR spectroscopy. Saponification of the ethyl dienoates (**40b**–**47b**) followed by Steglich esterification of the corresponding carboxylic acids (**40c**–**47c**), gave rise to the desired SNAC esters (**40**–**47**), thiophenol **48** and acetal **49a**. The latter was deprotected under acid conditions to yield pantetheine **49**.

**Figure 4:**
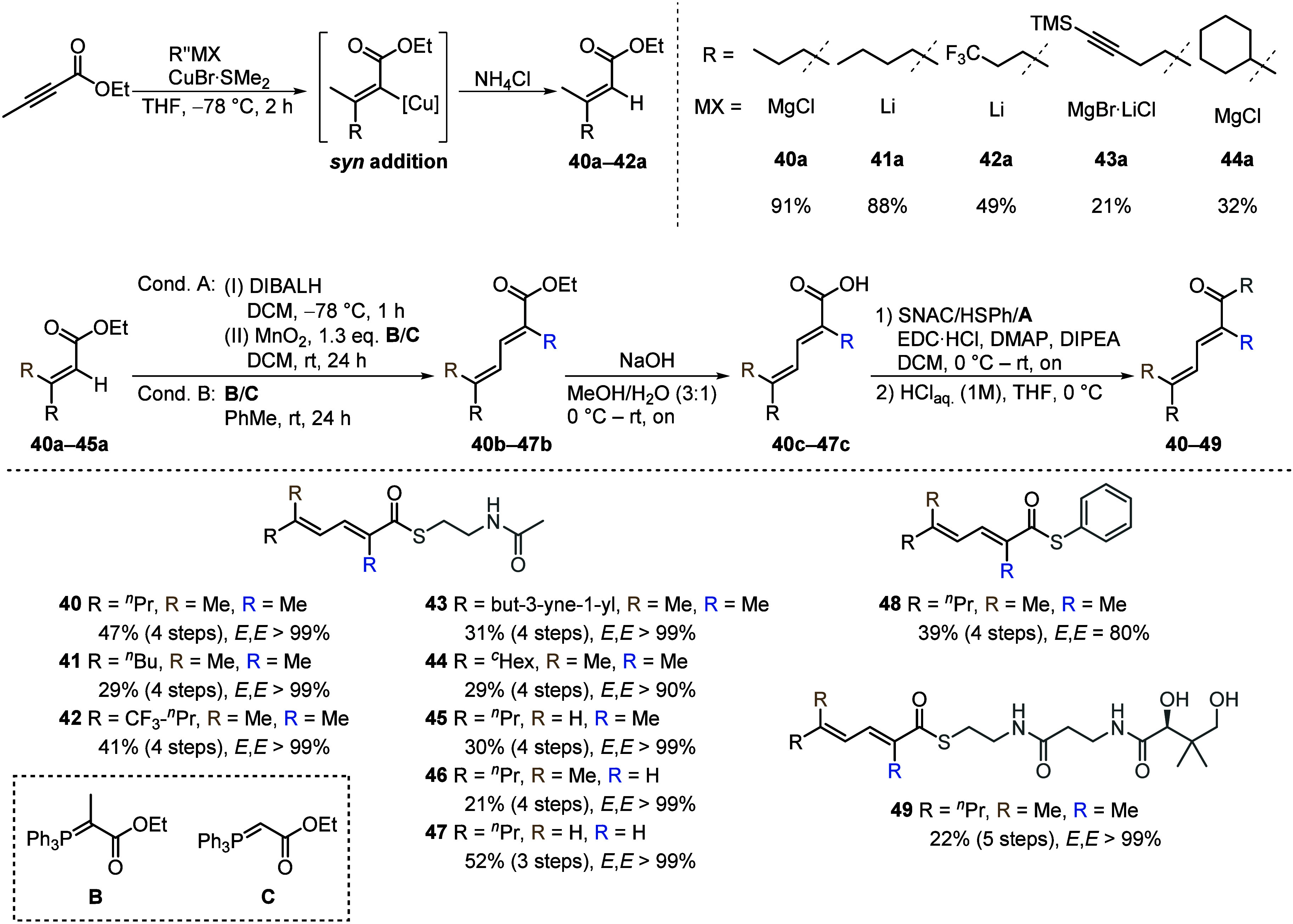
Synthesis of mutasynthons (**40**– **49**) for CP-4^W^S91A mutant supplementation. The dienoates were installed via a sequence comprising copper-mediated conjugate addition to ethyl but-2-ynoate, reduction to the corresponding alcohols, and a one-pot in situ allylic oxidation/Wittig olefination. Saponification and thioester formation led to desired mutasynthons.

### Probing Mutasynthon Acceptance and Isolation of Analogs

To confirm that synthetic mutasynthon SNAC esters can be taken up and incorporated by the *M. xanthus* DK1622 *CP-1* ^*W*^ *S91A* mutant to restore the myxopyronin production, we initially supplied **20**, corresponding to the native MYX A (**1**) first-module intermediate. The respective mutasynthon **20** was added to the cultures after 2 days of incubation, reaching a final concentration of 1 mM. The production cultures were subsequently harvested after a total cultivation period of 7 days and analyzed using HPLC-MS (see Supporting Information for details). We successfully restored the production of **1**, albeit at lower levels compared to the wild-type strain *M. fulvus* Mx f50 and the overexpression mutant strain *M. xanthus* DK1622 cultivated in parallel (Figure 5). To assess the integration efficiency of **20**, we additionally supplemented the culture broth with thiophenol (**34**) and pantetheine (**37**), which yielded detectable but comparatively lower production levels. Comparable experiments incorporating module four intermediate **40** into the *M. xanthus* DK1622 *CP-4* ^*W*^ *S91A* mutant demonstrated restoration of **1**. However, the restoration was achieved with lower efficiency relative to SNAC **20**. Further investigations revealed that supplementation with the corresponding thiophenol **48** exhibited toxicity to the strain at the tested concentrations and diminished the MYX A production completely. In contrast, supplementation with pantetheine **49** supported detectable production levels **1**.

**Figure 5:**
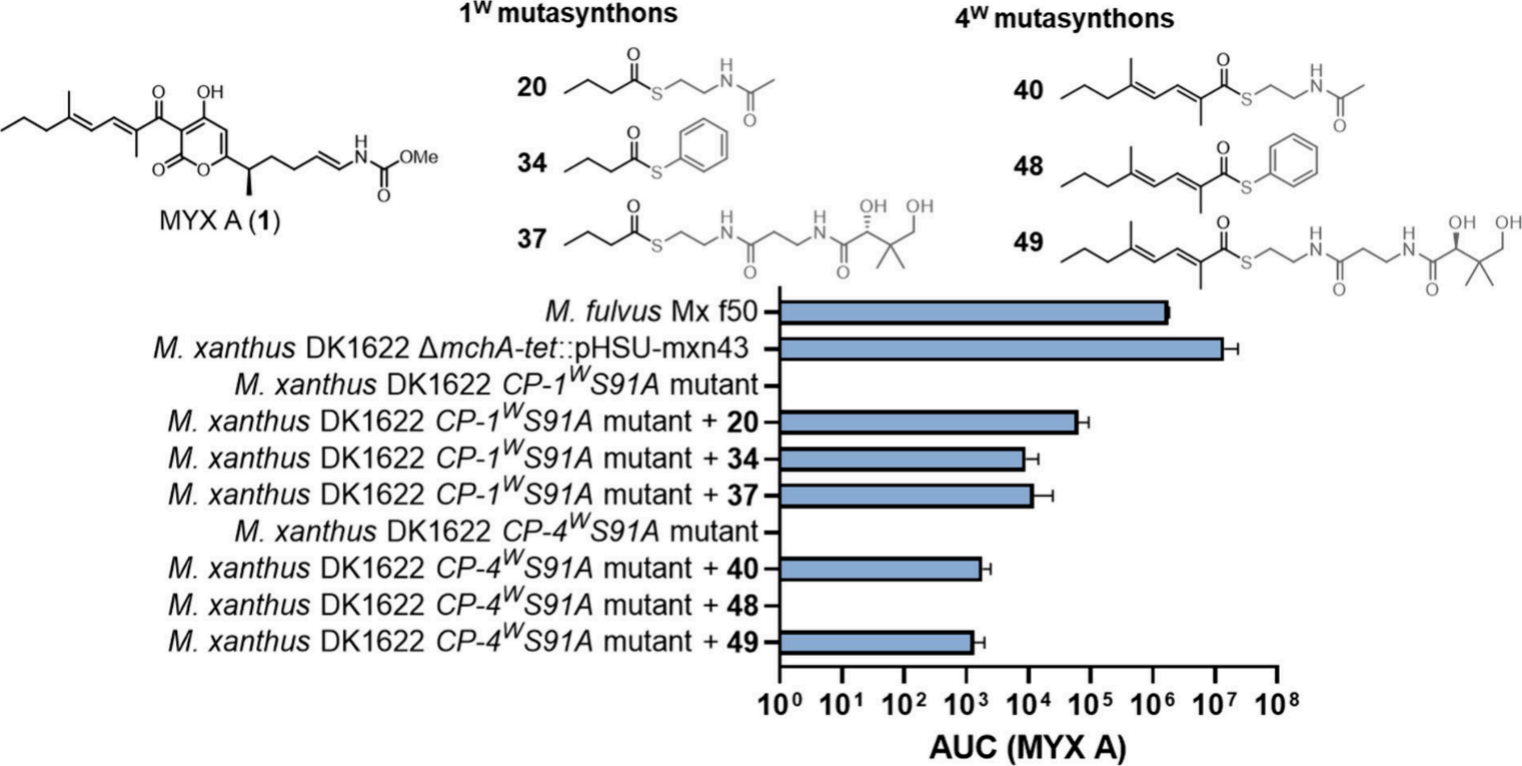
Evaluation of production restoration for compound 1 and mutasynthon integration efficiency using various thiol esters. MYX A (**1**) production in wild-type strain *Myxococcus fulvus* Mx f50, overexpression mutant strain *M. xanthus* DK1622 ΔmchA-tet::pHSU-mxn43, CP-1^W^S91A mutant, CP-1^W^S91A mutant supplemented with **20**, **34** and **37**, CP-4^W^S91A mutant, CP-4^W^S91A mutant supplemented with **40**, **48**, and **49**.

After restoring MYX A (**1**) production, we evaluated substrate flexibility by introducing the superior SNAC-based mutasynthons (**22**–**34**) into the *CP-1* ^*W*^ *S91A* mutant (Figure 6). Besides the incorporation of butyl building block **21** for MYX B (**2**) production, we explored the introduction of a trifluoromethyl group to enhance metabolic stability. Encouragingly, derivative **8** was produced, albeit at slightly lower levels compared to MYX A, when the mutant strain was supplemented with **22**. Building on this, we sought to introduce a terminal alkyne motif by feeding the mutant strain with the mutasynthon **23**. The obtained MYX derivative **9** could subsequently be modified via copper-catalyzed azide–alkyne cycloaddition (CuAAC) or other 1,3-dipolar cycloaddition reactions.^41–43^ These modifications would yield elongated COR-like structures, possibly with enhanced interactions with the adjacent lipophilic region of RNAP. Lastly, we attempted to incorporate a larger substituent by utilizing the cyclohexyl precursor (**24**), which resulted in production levels of **10** comparable to those of analog **9**.

**Figure 6:**
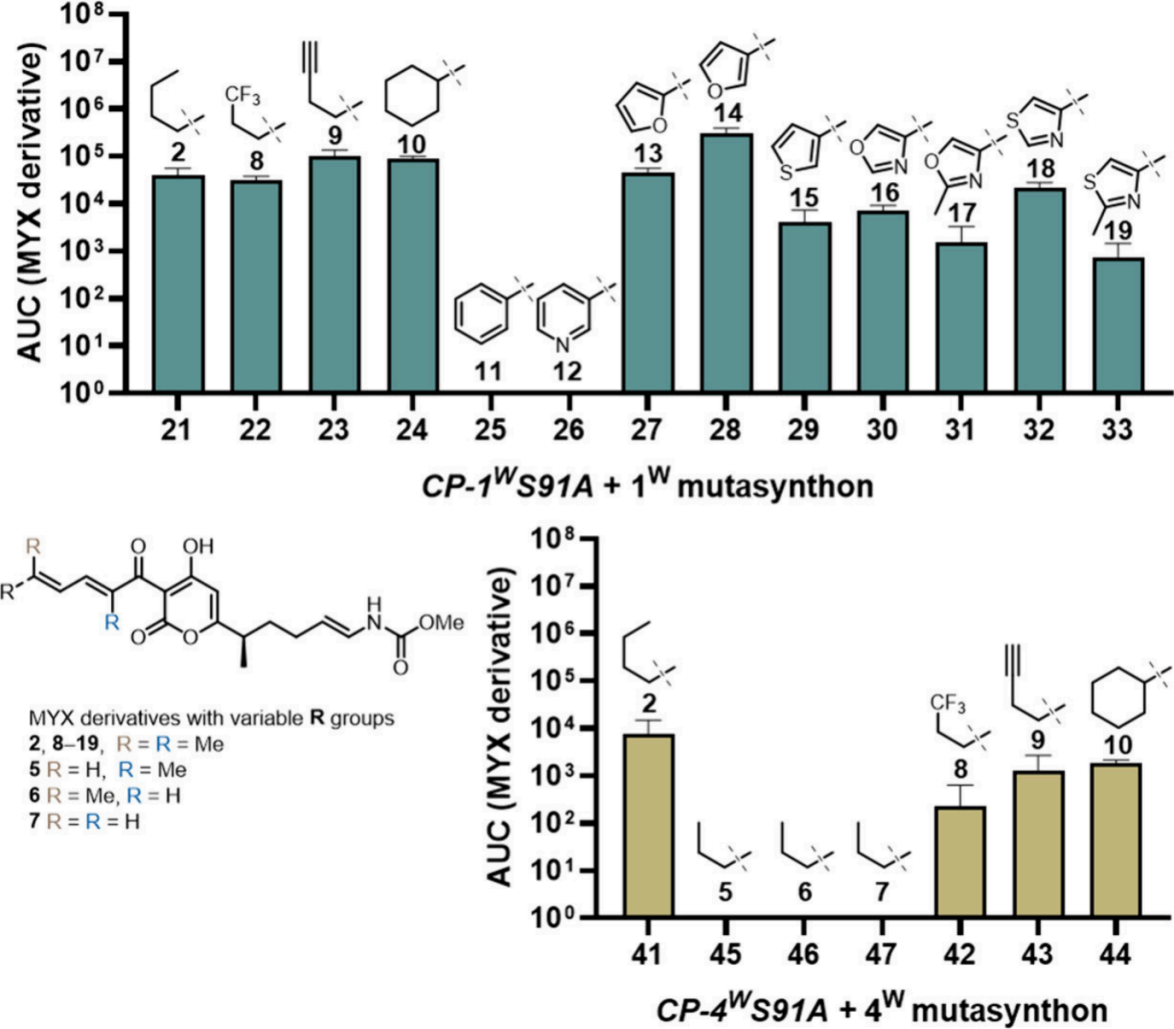
Restoration of production for **MYX B** (**2**), and derivatives **5**–**19**. CP-1^W^S91A mutant supplemented for production of **2**, and derivatives **8**–**19** with respective 1^W^ mutasynthons **21**–**33** (top). CP-4^W^S91A mutant supplemented for production of **2**, and derivatives **5**–**10** with 4^W^ mutasynthons **41**–**47** (bottom).

Moreover, we challenged our mutasynthesis platform by transitioning from alkyl precursors to aromatic ones. For six-membered (hetero)­aromatics, this significant structural modification resulted in the complete abolishment of production for analogs **11** and **12** when fed with phenyl-SNAC (**25**) or nicotinic acid-SNAC (**26**). Unexpectedly, the slightly smaller five-membered heterocyclic precursors **27**–**33**, were well-accepted for the production of analogs **13**–**19**.

Next, we investigated the substrate specificity of the *CP-4* ^*W*^ *S91A* mutant by altering the methylation pattern using mimics (**45**–**47**) (Figure 6 and Figure S2, Table S5). The removal of a single methyl group at either the α- or δ-position, or both, resulted in a complete loss of production of MYX (**5**–**7**). However, production of MYX (**8**–**10**) was restored when mutasynthons (**42**–**44**) were supplied, underscoring the importance of both methyl groups in substrate recognition by the KS domain of the *trans*-AT PKS.^26,44,45^ This observation aligns with the known substrate specificity of KS domains, which generally prefer substrates sharing similar structural characteristics around the thioester α- to γ-region.^44,46–48^ Additionally, those KS domains demonstrate flexibility in accommodating even more complex substrates with variations at the δ-position, as exemplified in COR (**3** and **4**) biosynthesis.

Based on the comprehensive information available, a subset of myxopyronin derivatives **8**, **9**, **10** and **14** were selected for scale-up fermentation to generate quantities sufficient for structure elucidation and bioactivity assessments. For each derivative, the most efficient starter unit was selected, utilizing the readily accessible module 1 SNACs **22**, **23**, **24** and **28**. These target structures were strategically chosen to serve as a proof-of-concept, addressing both structural diversity and potential utility as precursors for subsequent synthetic modifications.

We initially attempted the large-scale production of furan analog **14**. However, it quickly became apparent that the compound is highly susceptible to rapid decomposition. This instability is most likely attributed to the conjugated system present in both the Western chain and the α-pyrone core. Even efforts to exclude light during fermentation and isolation did not prevent its degradation; consequently, we decided to discontinue further experimentation. Next, we focused on optimizing the production of the trifluoromethyl-modified NP **8**, terminal alkyne derivative **9** and cyclohexyl analog **10**. While transitioning the production of **9** and **10** from small- to large-scale proved unsuccessful, **8** demonstrated comparable production titers in 1L cultures in shaking flasks. In myxobacteria, both growth and secondary metabolite biosynthesis are highly sensitive to cultivation parameters, including adhesion to the vessel surface, cell aggregation, and—in the case of *M. xanthus* DK 1622—possible phase variation.^49^ In addition, the interplay between flask geometry, size and filling volume can unpredictably influence oxygen availability, thereby affecting both biomass formation and secondary metabolite production.^50^

To enhance the supply of analog **8**, we conducted a preliminary screening in 1L cultivations, utilizing a Parallel Bioreactor System (Table S6). By adapting bioreactor conditions for COR A (**3**) (temperature (T) maintained at 30 °C and partial pressure of oxygen (pO_2_) at 20%),^51^ we first evaluated the impact on pH, and feeding strategies—either a single batch addition after 48 h or a progressive approach with SNAC **22** added at 24, 48, 72, and 96 h postinoculation (Figure 7A). Compared to conventional fermentation in shaking flasks, bioreactor-mediated production initially yielded only one-sixth of **8** under pH-controlled conditions with a single-batch addition of the mutasynthon. However, progressive addition of **22** under the same conditions increased production of **8** by one-third. In the absence of pH-controlled conditions, production of NP **8** decreased significantly.

**Figure 7:**
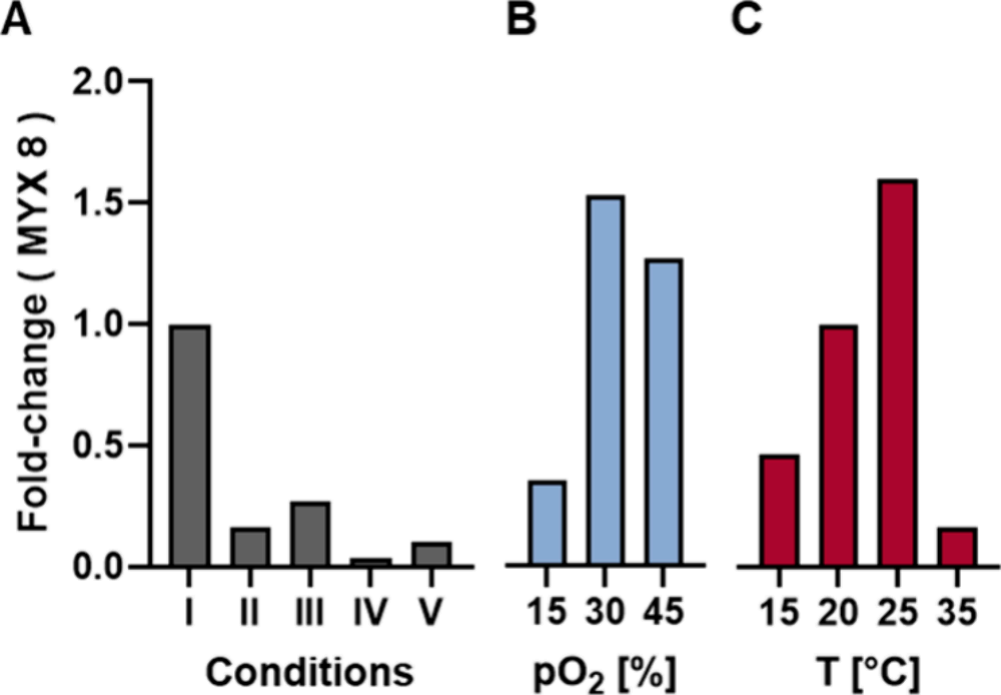
Initial screening of MYX **8** production in conventional shaking flasks versus bioreactor. (A) (I) shaking flask cultivation, (II) pO_2_: 20%, pH: 7.4, T: 30 °C, single batch addition of **22**, (III) pO_2_: 20%, pH: 7.4, T: 30 °C, single progressive feeding of **22**, (IV) pO_2_: 20%, no pH-control,, T: 30 °C, single batch addition of **22**, (V) pO_2_: 20%, no pH-control, T: 30 °C, progressive feeding of **22**. (B) Variation of pO_2_ [%]. (C) Variation of T [°C].

Based on these results, we aimed to optimize the oxygenation level near the previously tested pO_2_ value of 20% (Figure 7B). Oxygenation at 15% resulted in a production profile similar to that observed at 20%, while 30% oxygenation yielded the highest production of the trifluoromethyl derivative **8**, exhibiting a 50% increase compared to conventional cultivation. In contrast, at 45% oxygenation, a slight but not significant decrease in production was observed. Finally, we varied the temperature in 5 °C increments while maintaining pO_2_ at 30% (Figure 7C). Interestingly, even at 15 °C, the production of **8** exceeded that observed under the initial bioreactor conditions. At 20 °C, production levels were comparable to those achieved with shaking flask fermentation. Optimal production was observed at 25 °C, underscoring the distinction between the conditions required for favoring NP synthesis and the optimal growth temperature range of *M. xanthus*, typically 34–36 °C. This finding is further supported by the drastic decrease in analog **8** production at 35 °C, despite substantial *M. xanthus* growth, as indicated by a high carbon dioxide transfer rate.

After optimization of this process, we conducted a large-scale fermentation (10L) of *M. xanthus* DK1622 *CP-1* ^*W*^ *S91A* mutant supplemented with **22**. Following extractions with methanol and ethyl acetate and sample were concentrated, the crude material underwent purification via Sephadex LH-20 chromatography, and preparative high-performance liquid chromatography (HPLC) to yield 3.5 mg of the desired trifluoromethyl analog **8**. Structural assignment of **8** was performed with a combination of 1D and 2D NMR experiments as well as high resolution mass spectrometry (HRMS) (Figures S3–S5 and Table S7).

### Evaluation of Antimicrobial Activity and in Vitro ADMET Profiling

The isolated derivative **8** was evaluated for its potency by determining the minimal inhibitory concentrations (MICs) against a diverse panel of Gram-negative bacteria such as *Escherichia coli* (*E. coli*) and the efflux-deficient *E. coli* Δ*tolC* strain with the inactivated outer membrane channel TolC complexes as well as clinically relevant Gram-positive pathogens including *M. tuberculosis*. As standard-of-care antibiotics, rifampin and ciprofloxacin were used. Additionally, in vitro ADMET properties of MYX A (**1**) and analog **8** were examined.

Comparable to NP **1**, no antimicrobial effect was observed for the trifluoromethyl derivative **8** against wild-type *E. coli* (Table 1). However, both were confirmed to exhibited good activity, with MIC values of 1–2 and 4 μg/mL against *E. coli* Δ*tolC*, displaying an increased sensitivity to those antimicrobial agents.^8,23^ Intriguingly, **8** was confirmed to demonstrate sustained potency against Gram-positive *S. aureus* wild-type strains compared to **1**, achieving a MIC of 2 μg/mL.^8^ Most interestingly, it also exhibited inhibitory activity against antibiotic-resistant *S. aureus* strains, with MIC values of 2 μg/mL for a rifampin-resistant strain, 2–4 μg/mL for methicillin-resistant strain, and 1–2 μg/mL for multidrug-resistant strain (methicillin- and vancomycin-resistant) when compared to MYX A (**1**). Beyond that, no inhibition was observed for *Enterococcus faecalis* (*E. faecalis*), wild-type *E. faecium*, or vancomycin-resistant *E. faecium*. Furthermore, MICs were determined against *M. tuberculosis* wild-type and a rifampin-resistant strain. Notably, derivative **8** displayed inhibitory activity against both strains and demonstrated enhanced potency relative to the parent NP **1**.

**Table 1:** Antimicrobial Profiles for MYX A (**1**) and MYX (**8**)­^a^

	MIC [μg/mL]			
Strain	MYX A (**1**)	MYX (**8**)	rifampin	ciprofloxacin
wt-*E. coli* BW25113	>64	>64	16	0.0125–0.025
*E. coli* Δ*tolC*	1–2	4	16	0.0025–0.005
wt-*S. aureus* Newman	2–4	2	0.005	n.d.
Rif^R^-*S. aureus* Newman	2	2	>64	n.d.
M^R^ *-*S. aureus** N315	4	2–4	0.0025	n.d.
M^R^/V^R^-*S. aureus* Mu50	1–2	1–2	>64	n.d.
*E. faecalis* DSM-20478	>64	>64	1	n.d.
*E. faecium* DSM-20477	>64	>64	8–16	4
V^R^-*E. faecium* DSM-17050	>64	>64	0.03	2
wt-*M. tuberculosis* H37Rv	4	2	0.03	n.d.
Rif^R^-*M. tuberculosis* H37Rv	2–4	1	>64	n.d.

a The MIC determinations were performed in two independent experiments, each in duplicate. For *M. tuberculosis* wild-type and resistant strains, all assays were performed in triplicate. Definitions: *E. coli*, *Escherichia coli*; *S. aureus*, *Staphylococcus aureus*; *E. faecalis*, *Enterococcus faecalis*; *E. faecium*, *Enterococcus faecium*; *M. tuberculosis*, *Mycobacterium tuberculosis*, Wt, wild-type; Rif^R^, rifampin-resistant; M^R^, methicillin-resistant; V^R^, vancomycin-resistant; n.d., not determined.

Next, we determined the in vitro ADMET properties of trifluoromethyl derivative **8** in comparison to **1**, in order to estimate whether the structural changes introduced via mutasynthesis indeed impact pharmacokinetic properties of myxopyronins (Table 2). We observed metabolism of **1** in both mouse and human liver microsomes with high clearance in human liver microsomes (Cl_int_ 157 μL/mg/min), while lower turnover was observed in rat liver microsomes. A similar trend was found for **8**, yet with significantly increased half-life in murine and human liver microsomes, consistent with previous reports for the latter.^23^ More detailed investigations into the human microsomal metabolism revealed a major contribution by CYP2C9 (Figure S6). Interestingly, both compounds were found to be stable in murine hepatocytes, a finding which might be related to insufficient permeation into the liver cells or susceptibility to drug efflux as shown previously for COR A (**3**).^52^ No degradation in plasma was observed for both compounds across species and plasma protein binding (PPB) was found to be high (>98%), both in line with published data on COR A (**3**).^52^ Lastly slightly cytotoxic effects on human liver cancer cell (HepG2) viability at 100 μM of 39 ± 6% for MYX A (**1**) and 44 ± 9% for **8** were observed.

**Table 2:** In Vitro ADMET Profiling of **1** and **8** ^a^

Parameter	Species/Cells	MYX A (**1**)	MYX (**8**)
Liver microsomes *t*_1/2_ [min]/Cl_int_ [μL/mg/min]	Mouse	19 ± 4/76 ± 16	68 ± 7/21 ± 2
	Human	7.3 ± 2.5/211 ± 37	52 ± 13/28 ± 7
	Rat	∼120/∼11.6^a^	∼120/∼11.6^a^
Hepatocyte *t*_1/2_ [min]/Cl_int_ [μL/mg/10^6^ cells]	Mouse	>240/<3.8	>240/<3.8
Plasma *t*_1/2_ [min]	Mouse	>240	>240
	Human	>240	>240
	Rat	>240	>240
PPB [%]	Mouse	99.74 ± 0.02	99.64 ± 0.08
	Human	98.93 ± 0.21	98.38 ± 0.51
	Rat	99.46 ± 0.12	99.51 ± 0.10
Viability at 100 μM [%]	HepG2	39 ± 6	44 ± 9

a Values represent means ± SD of at least two independent experiments. C57BL/6 mice and Wistar rat microsomes/plasma were used. *t*_1/2_: half-life; Cl_int_: intrinsic clearance.

b Remaining amount of parent compound at 120 min: 51 ± 12% (**1**)/49 ± 13% (**8**).

Taken together, the mutasynthetic incorporation of a trifluoromethyl group did not significantly impact antimicrobial activity or cytotoxicity. However, it substantially increased metabolic stability in mouse and human liver microsomes, while showing no apparent effect on the lower turnover observed in rats or on PPB across species. These findings, particularly the rather high PPB, will be considered in the future development of the scaffold.

## Conclusions

This work builds on over a decade of research in microbial engineering, encompassing pivotal advancements from the initial mutasynthesis experiments in the native host *M. fulvus* Mx f50, to the heterologous expression of MYX (**1**–**2**) in *M. xanthus* DK1622 *ΔmchA-tet:*:pHSU-mxn43. The latter approach was accompanied by significant improvements in production titers, which laid the foundation for the successful mutasynthetic production of the α-pyrone antibiotic **8** described herein. Furthermore, the trifluoromethyl-modified analog **8** was confirmed to demonstrate potent antimicrobial activity against antibiotic-resistant *S. aureus* and *M. tuberculosis* strains and favorable in vitro ADMET parameters in particular increased metabolic stability.^23^

Traditionally, MYX compounds (**1** and **2**) and their derivatives have been accessed through total synthesis. The Panek group initially reported the racemic synthesis of (±)-MYX A (**1**) and B (**2**),^16^ which was subsequently adapted by Anadys Pharmaceuticals Inc. and the Ebright group to produce various analogs—requiring up to 14 steps, depending on the target compound.^15,17,19–24^ More recently, Kalesse and colleagues achieved the enantioselective synthesis of MYX B (**2**) in 22 steps, with an overall yield of ∼0.4%.^18^ However, the synthetic routes suffer in flexibility when it comes to modification of the Western chain, since each desired analog requires a full de novo synthesis of the entire scaffold. In contrast, the method presented here combines a simple, one-step SNAC synthesis with mutasynthetic production of analogs such as MYX **8**, achieving yields of ∼ 0.07% after extraction and purification. While further optimization is required, this approach could significantly accelerate analog generation, as most SNAC derivatives are well tolerated by the biosynthetic machinery. While each strategy—chemical synthesis and mutasynthesis—offers distinct advantages and limitations depending on the analog, their integration into a hybrid synthetic–biosynthetic strategy holds significant potential to access natural product analogs that are otherwise inaccessible through purely chemical or biological means.^27,53,54^

Moreover, the biotechnology platform presented herein not only enables the generation of new MYX congeners, but also opens up possibilities for enhancing their activity against Gram-negative pathogens. Future efforts will focus on scaffold modifications aimed at improving outer membrane permeability and evading active efflux, by optimizing hydrophilicity/hydrophobicity parameters (logP between −2 and 2) or introduce Zwitterionic motifs (e.g., such as in fluoroquinolones).

Beyond that, this platform also serves as a blueprint for designing novel COR-like analogs in the future. This is particularly significant because efforts to establish a mutasynthesis system in the native host of COR (**3** and **4**), *Corallococcus coralloides*, have been unsuccessful. These challenges arise from the organism’s limited genetic amenability, has thus far precluded pathway engineering.

While this study set the stage for the generation of MYX analogs, overall yields are still below requirements for further development of the scaffold as the mutasynthesis lowers production titers significantly (∼0.90 mg/L) versus the native system (∼156 mg/L).^29^ Thus, ongoing investigations are focused on enhancing production yields through genetic modifications in *M. xanthus* DK1622*ΔmchA-tet* such as introduction of strong promoters or optimization of expression levels of regulators.^55–59^ Beyond that alternative hosts such as yeast for improved precursor tolerance and flux will be taken into account to facilitate further optimization of these promising α-pyrone antibiotics.^60^

## Supplementary Material



## Notes

Additional experimental details, materials, and methods, including copies of NMR spectra for compounds **8**, **20**–**49** (PDF)
