# Supplementary material for: Engineering an Artificial Myxopyronin Derivative with Enhanced Metabolic Stability via Mutasynthesis
Source: JACS Au. 2025 Nov 18;5(12):6060–71. doi: 10.1021/jacsau.5c00950 (PMC12728655; doi:10.1021/jacsau.5c00950)
Supplement: Supplementary file 1 [file au5c00950_si_003.pdf]

Supporting Information

Engineering an Artificial Myxopyronin Derivative with Enhanced Metabolic Stability via Mutasynthesis

Alexander F. Kiefer[1,2,3,$], Alexander Voltz[1,2,3,4,$], Domen Scherzer[1,2,3], Roman Reberšek[1,2,3,4], Andreas M. Kany[1,2,3], Gareth Prosser[2,5], Markus Neuber[1,2,3], Norbert Reiling[2,5], Anna K. H. Hirsch[1,2,3,4,*] and Rolf Müller[1,2,3,4,*]

[1] Helmholtz Institute for Pharmaceutical Research Saarland (HIPS), Campus E8.1, 66123, Saarbrücken, Germany - Helmholtz Centre for Infection Research (HZI), Inhoffenstraße 7, 38124, Braunschweig, Germany

[2] German Center for Infection Research (DZIF), Inhoffenstraße 7, 38124, Braunschweig, Germany

[3] PharmaScienceHub (PSH), Campus A2.3, 66123, Saarbrücken, Germany

[4] Saarland University, Department of Pharmacy, Campus E8.1, 66123, Saarbrücken, Germany

[5] Microbial Interface Biology, Research Center Borstel, Leibniz Lung Center, Parkallee 1, 23845 Borstel, Germany

[$] These authors contributed equally.

E-mail: anna.hirsch@helmholtzhips.de and rolf.mueller@helmholtz-hips.de

Table of Contents

In silico docking studies S3
Construction and engineering of plasmids S4
Construction of pHSU-Mxn43-ACP1 and pHSU-Mxn43-ACP4 expression constructs S6
Transformation and chromosomal integration of expression constructs into *M. xanthus* DK1622 .... S7
Analysis of the *M. xanthus* DK1622Δ*mchA-tet*::pHSU-mxn43-ACP1 and DK1622Δ*mchA-tet*::pHSU-mxn43-ACP4 production profile S8
Production analysis by HPLC-HRMS S9
Production in fermenter system S11
Chemistry S12
General procedure A (GP A): Copper-catalyzed 1,4-addition to ethyl-2-butynoate S12
General procedure B (GP B): DibalH reduction & *in situ* alcohol oxidation–Wittig reaction S13
General procedure C (GP C): NaOH-mediated saponification S13
General procedure D (CP D): SNAC-esters formation from carboxylic acids S13
Bacterial assay S44
Metabolic stability in liver microsomes S45
Metabolic stability in mouse hepatocytes S46
Plasma stability S47
Plasma protein binding S47
Cytotoxicity assay S48
Copies of NMR spectra S49
References S117

In silico docking studies

MM2 energy optimization of MYX derivatives (**1**, **2**, **5–20**) was performed using Chem3D version 22.2.0.3300 (PerkinElmer). Docking was performed by using the UCSF Chimera version 1.17.3 and AutoDock Vina version 1.1.2. The receptor search volume was set to the center coordinates: –88, –27, –22 and a size of 24.5, 30 and 19.5. The docking was based on the co-crystal structure of MYX A (**1**) with *Thermus thermophiles* RNAP (PDB ID: 3DXJ).[1]

Table S1: *In silico* docking results of the naturally occurring myxopyronins (**1**–**2**) in comparison to the planned artificial derivatives (**5**–**19**)

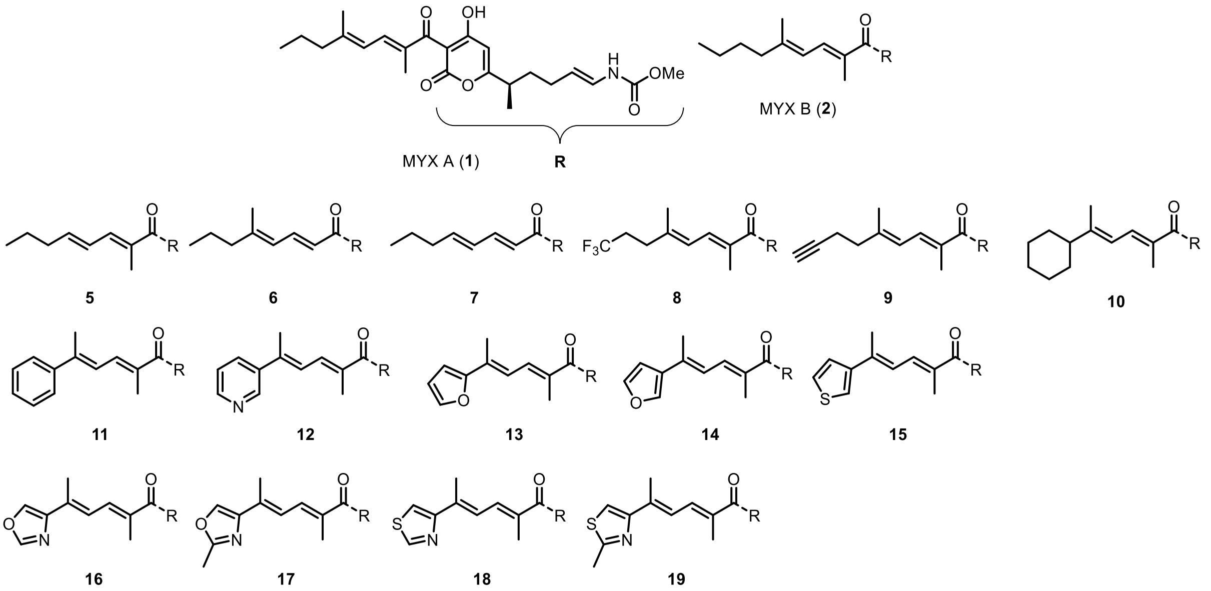

Compound	Calculated energy score
**MYX A (1)**	–9.4
**MYX B (2)**	–9.5
**5**	–9.1
**6**	–9.0
**7**	–9.5
**8**	–10.4
**9**	–10.0
**10**	–10.7
**11**	–10.8
**12**	–10.2
**13**	–10.0
**14**	–9.8
**15**	–9.9
**16**	–10.1
**17**	–10.1
**18**	–9.8
**19**	–10.3

Construction and engineering of plasmids

Routine handling of nucleic acids, such as isolation of plasmid DNA, restriction endonuclease hydrolysis, DNA ligation and other DNA manipulations, was performed according to standard protocols (Sambrook and Russell, 2001). *E. coli* HS996 (Invitrogen) was used as host for standard cloning experiments. *E. coli* strains were cultured in LB medium (1% tryptone, 0.5% yeast extract, 0.5% NaCl, (1.5% agar)) at 30–37 °C (and 200 rpm) overnight. Antibiotics were used as selection markers at the following final concentrations: 100 µg/mL ampicillin and 50 µg/mL kanamycin. Transformation of *E. coli* strains was achieved *via* electroporation in 0.1 cm-wide cuvettes at 1250 V, a resistance of 200 Ω, and a capacitance of 25 µF. Plasmid DNA was either purified by standard alkaline lysis,[2] by using the GeneJet Plasmid Miniprep Kit (Thermo Fisher Scientific) or the NucleoBond PC100 kit (Machery Nagel). Restriction endonucleases, alkaline phosphatase (FastAP) and T4 DNA ligase were obtained from Thermo Fisher Scientific. Oligonucleotides used for PCR and Sanger sequencing were obtained from Sigma-Aldrich and are listed in Table S1. DNA fragments from restriction hydrolysis were purified from agarose gel after agarose gel electrophoresis and isolated using the NucleoSpin® Gel and PCR Clean-up (Macherey-Nagel) or peqGold Gel Extraction (Peqlab). Red/ET recombineering experiments for plasmid modifications[3] were performed according to the manufacturers protocol (Gene Bridges GmbH) using the strain *E. coli* GB05-red. After selection with suitable antibiotics, clones harboring correct recombination products were identified by plasmid isolation followed by an independent hydrolysis with different endonucleases (leading to characteristic DNA band patterns in agarose-GE). Synthetic DNA fragments were obtained from ATG:biosynthetics GmbH and delivered in pGH based standard cloning vectors (Table S3). Details on the construction of plasmids generated in this study are given in Table S4.

Table S2: Oligonucleotides used in this study.

Primer name	Sequence (5´→3´)
dpo-mxn43-ACP1	GGGGATTCCGTCCAGGTG
dpo-mxn43-ACP4	CGCCTCGAGTCTCCGAAC
P1	CGAGCAATCCGCTATTGGC
P2	GAGAACCTGCGTGCAATC
P3	TCGGTTCAAAGAGTTGGTAGC
P4	CTGTGTCCTTCTGCGACGC

Table S3: Gene synthesis constructs obtained from ATG:biosynthetics GmbH.

Construct name	Fragment size	Description
Mxn_ACP1	1173	Synthetic construct containing the ACP1 domain of the myxopyronin biosynthetic gene cluster (BGC) with mutated active site (serine to alanine mutation), and chloramphenicol resistance gene (cmR) for selection after *in vivo* recombineering in *Escherichia coli*. The construct is flanked by 50 bp homology regions, homologous to the corresponding ACP1 region in the myxopyronin BGC and restriction sites (BglII and NotII) for release from the commercial pGH vector.
Mxn_ACP4	1218	Synthetic construct containing the ACP4 domain of the myxopyronin BGC with mutated active site (serine to alanine mutation), and chloramphenicol resistance gene (cmR) for selection after *in vivo* recombineering in *E. coli*. The construct is flanked by 50 bp homology regions, homologous to the corresponding ACP1 region in the myxopyonin BGC and restriction sites (BglII and NotII) for release from the commercial pGH vector.

Table S4: Plasmids and expression constructs generated in this study.

Plasmid name	Construction details	Features
pHSU-mxn43	Sucipto et al., 2017[4]	PnptII-mxnA-M, p15A ori, tetR, kanR
pHSU-mxn43-ACP1-cmR	ACP1 domain of pHSU-mxn43 replaced with 1173 bp fragment from pGH-ACP1-cm by *in vivo* recombination.	PnptII-mxnA-M, p15A ori, tetR, kanR, cmR, mxnK c.5059 T>G
pHSU-mxn43-ACP1	pHSU-mxn43-ACP1-cmR digested by XmaJI restriction endonuclease and re-ligated, to excise the cmR	PnptII-mxnA-M, p15A ori, tetR, kanR, mxnK c.5059 T>G
pHSU-mxn43-ACP4-cmR	ACP4 domain of pHSU-mxn43 replaced with 1218 bp fragment from pGH-ACP4-cm by *in vivo* recombination.	PnptII-mxnA-M, p15A ori, tetR, kanR, cmR, mxnK c.15505 T>G
pHSU-mxn43-ACP4	pHSU-mxn43-ACP4-cmR digested by XmaJI restriction endonuclease and re-ligated, to excise the cmR	PnptII-mxnA-M, p15A ori, tetR, kanR, mxnK c.15505 T>G

Construction of pHSU-Mxn43-ACP1 and pHSU-Mxn43-ACP4 expression constructs

The 1173 bp long Mxn_ACP1 and 1218 bp long Mxn_ACP4 synthetic DNA fragments were chemically synthesized and delivered in standard pGH cloning vectors (ATG:biosynthetics GmbH). The fragments were flanked by 50 bp homology regions homologous to the corresponding ACP1 and ACP4 regions in the myxopronin BGC. They contained the active site region of the corresponding ACP domain; however, serine in the active site was mutated by single-nucleotide substitution/mutation. Mutation was achieved by conversion of the native TCG codon to GCG for ACP1 and GCC for ACP4, thereby mutating serine to alanine. Both constructs also harbored a chloramphenicol resistance gene (*cmR)* for selection after *in vivo* recombineering in *E. coli*. The *cmR* gene was flanked by *Xma*JI restriction sites (R-sites) for subsequent excision and religation. As the pHSU-mxn43 expression construct is not BsaI free, we could not make use of previously described scarless plasmid modification method using type IIS restriction endonuclease (Pogorevc et al., 2019) and had to select one of the standard restriction enzymes instead. The *XmaJ*I R-site was selected due its recognition sequence (CCTAGG) which encodes P and R. The same amino acids are natively present at this position in the pHSU-mxn43 so the modifications only lead to the single-nucleotide substitution/mutation compared to the native sequence. Finally, the synthetic DNA fragment was flanked by unique restriction sites (*Bgl*II and *NotI*I) to allow excision from the commercial vector. After a single *in vivo* recombination step in *E. coli* GB05-red, the corresponding ACP1 or ACP4 sequence in the *mxnK* gene was replaced by the mutated ACP1 or ACP4 domain and *cmR* gene, correct *E. coli* clones were selected on LB medium supplemented with 12.5 µg/mL chloramphenicol. The resulting pHUS-mxn43-ACP1-cmR and pHUS-mxn43-ACP4-

cmR plasmids were digested by *Xma*JI restriction endonuclease and re-ligated, to excise the *cmR*. The sequences of the final pHUS-mxn43-ACP1 and pHUS-mxn43-ACP4 constructs were confirmed by Illumina sequencing, before transformation into *M. xanthus* DK1622.

Transformation and chromosomal integration of expression constructs into *M. xanthus* DK1622

According to a previously established electroporation procedure for *M. xanthus* DK1622[5] the host strain *M. xanthus* DK1622*ΔmchA-tet*[6] was transformed with the generated expression constructs pHUS-mxn43-ACP1 and pHUS-mxn43-ACP4 (Table S3). *M. xanthus* DK1622 mutants were cultivated at 30 °C in CTT medium (1% casitone, 10 mM Tris buffer pH 7.6, 1 mM KH_2PO_4 pH 7.6, 8 mM $MgSO_4$ (1.5% agar) with final pH 7.6). For cultivation of liquid cultures, the strains were grown in Erlenmeyer flasks on an orbital shaker at 180 rpm for 2–3 days. For selection of *M. xanthus* mutants, 50 µg/mL kanamycin was used. After electroporation, the transformed cells were resuspended in 1 mL CTT medium and incubated for 6 h under constant shaking. Afterward, the transformants were mixed with 3 mL of CTT soft agar (CTT with only 7.5 g /L agar) and poured onto a CTT agar plate. After hardening of the soft agar layer with embedded transformant cells, the plates were incubated at 30 °C for 5 days. Embedding in the soft agar prevented swarming of the transformant colonies over the agar surface, allowing the isolation of single clones and transfer onto a fresh CTT agar plate. Correct chromosomal integration of the expression construct into the *tetR* locus of the *M. xanthus* DK1622*ΔmchA-tet* mutant *via* homologous recombination was verified by PCR (Fig. S1). Transformant cells (1 cm^2) were scratched from the plate, resuspended in 50 µL of water and subsequently lysed by incubating at 95 °C for 30 min prior of being added to the PCR reaction (‘colony PCR’). For each expression construct correct chromosomal integration was confirmed using two primer combinations, revealing PCR products of the expected sizes: P1/P2 (1609 bp) and P3/P4 (1635 bp). Genomic DNA of *M. xanthus* DK1622*ΔmchA-tet* isolated using Qiagen Puregene Core Kit A, was used as negative control. The complementary experiment using primers P1/P4 revealed a 1461 bp PCR product for *M. xanthus* DK1622*ΔmchA-tet*. The same primer pair yielded no PCR product in the expression strain.

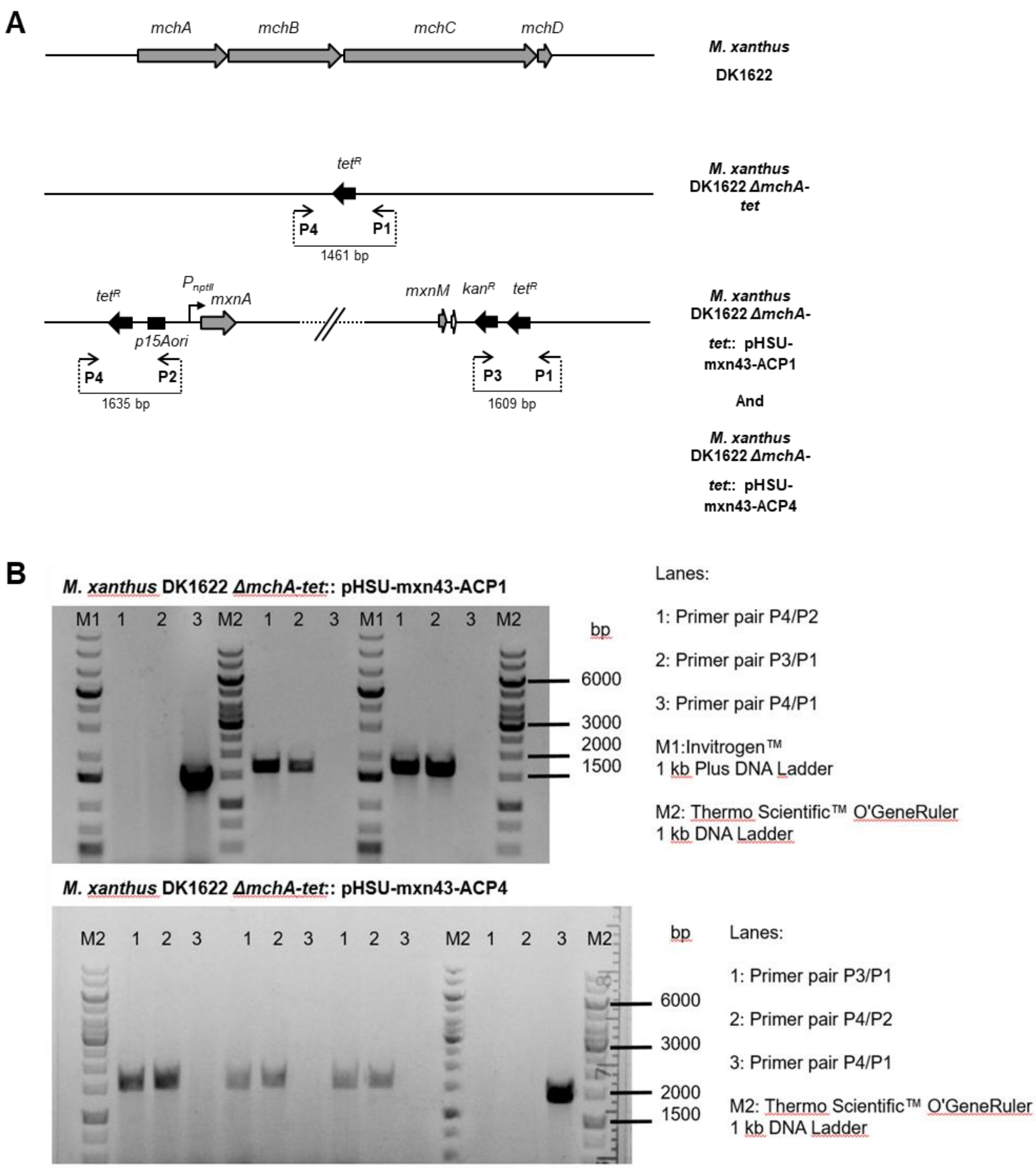

Figure S1: Genotypic verification of the integration of both clusters into the heterologous host *Myxococcus. xanthus* DK1622 Δ*mchA-tet* was performed. (A) The primer sets used for this verification, including their binding sites and amplicon sizes, are depicted. The nucleotide sequences of the primers are provided in Table S1, (B) confirmation by PCR followed by gel-electrophoresis.

Analysis of the *M. xanthus* DK1622*ΔmchA-tet*::pHSU-mxn43-ACP1 and DK1622*ΔmchA-tet*::pHSU-mxn43-ACP4 production profile

To verify that the mutation of the active site serine in the ACP1 and ACP4 of the mxnK resulted in abolished myxopyronin production the *M. xanthus* DK1622*ΔmchA-tet*::pHSU-mxn43-ACP1 and DK1622*ΔmchA-tet*::pHSU-mxn43-ACP4 were cultivated in parallel with the *M. xanthus*

DK1622*ΔmchA-tet*::pHSU-mxn43. The strains were inoculated from cryo stocks and grown on CTT agar plates supplemented with kanamycin 50 µg/mL for several days until the plates were mostly overgrown with swarming cells. All of the cells were scraped from the plates to inoculate M7/S4 medium (0.5% soy flour, 0.5% corn starch, 0.2% glucose, 0.1% yeast extract, 0.1% $MgSO_4$ x 7 H_2O, 0.1% $CaCl_2$ x 2 H_2O, 1% HEPES, with final pH 7.4 and supplemented with 0.1 mg/L of vitamin B12 and 5 mg/L of $FeCl_3$ after autoclaving) (15 mL medium in a 50 mL Erlenmeyer flask) and the seed cultures were cultivated at 30 °C, 180 rpm Orbitron an orbital shaker (Infors HT) for 48 h. 5 mL of the preculture was used to inoculate 50 mL of the M7/S4 medium in which the strain was grown under the same conditions for 6 days. All cultures were supplemented with kanamycin. All strains were initially cultivated in biological triplicates and technical triplicates of one clone were cultivated in the follow–up experiments. The cultivation took 7 days before extraction with 25 mL ethyl acetate. HPLC-HRMS analysis revealed expected MYX A (**1**) production by the *M. xanthus* DK1622*ΔmchA-tet*::pHSU-mxn43 strain, whereas no MYX A (**1**) could be detected in the extracts of *M. xanthus* DK1622*ΔmchA-tet*::pHSU-mxn43-ACP1 and DK1622*ΔmchA-tet*::pHSU-mxn43-ACP4.

The mutasynthetic fermentation experiments were conducted in three independent replicates in sterile 2 mL 96 well DeepWell™ Plates (Thermo Scientific™, Nunc™). The plates were filled with 900 µL of sterile M7/S4 medium, and 100 µL of the preculture was used for inoculation. The plates were covered with air–permeable lids with 1.5 mm ventilation holes (Kuhner, Duetz System, 104106) and fastened to an Orbitron orbital shaker (Infors HT) using a 4 position clamp for deepwell-plates (Kuhner, Duetz System 104167). Feeding with the substrate mimics was performed 48 h after inoculation. The substrates (100 mM stock solution in DMSO) were added to a final concentration of 1mM. After 7 days of cultivation, liquid-liquid partitioning with 1 mL of ethyl acetate was performed directly in the wells. The extraction was done using (Integra VIAFLO384) with 3 times 30, 1 mL mixing cycles. To separate the aqueous from the organic layer, the plates were centrifuged for 5 minutes at 4000 RPM (Eppendorf, Centrifuge 5810 R). 900 µL of the organic phase were removed and transferred into a new deep-well plate. Evaporation of the solvent was achieved by nitrogen flow across the open plate overnight. For analytical measurements, the dried extracts were dissolved in 36 µL methanol, centrifuged at 4°C with for 15 minutes 4.000 RPM (Eppendorf, Centrifuge 5810 R). The supernatant was transferred to conical 96 well-plates (AZENTA life sciences, 96 well skirted PCR plates, clear PP, low profile). Those were hot-sealed with pierceable aluminum foil (AZENTA Life Sciences, 4TI-0531) and used for HPLC-HRMS-measurements.

Production analysis by HPLC-HRMS

HPLC-HRMS measurements were performed on a Vanquish Flex UHPLC system (Thermo Scientific) equipped with (Waters) BEH C_{18} column (100 × 2.1 mm, 1.7 µm) equipped with a

VanGuard BEH C_{18} 1.7 µm guard column (Waters). Separation of 1 µL sample was achieved by a linear gradient from (A) H_2O + 0.1% FA to (B) ACN + 0.1% FA at a flow rate of 600 µL/min and 45 °C. The gradient was initiated by a 0.5 min isocratic step at 5% B, followed by an increase to 95% B in 18 min to end with a 2 min step at 95% B before re-equilibration with initial conditions. UV-vis spectra were recorded by a DAD in the range from 200 to 600 nm. The LC flow was split to 75 µL/min before entering the Bruker Daltonics (Bremen, Germany) maXis 4G HR-qToF mass spectrometer equipped with an Apollo II ESI source. The split was set up with fused silica capillaries of 75 and 100 µm I.D. and a low dead volume tee junction (Upchurch). Mass spectra were acquired in centroid mode ranging from 150-2500 *m/z* at a 2 Hz scan rate in centroid mode. Mass spectrometry source parameters were set to 500 V as end plate offset; +4000 V as capillary voltage; nebulizer gas pressure 1 bar; dry gas flow of 5 L/min and a dry temperature of 200 °C. Ion transfer and quadrupole settings were set to funnel RF 350 Vpp.; multipole RF 400 Vpp as transfer settings and ion energy of 5 eV as well as a low mass cut of 300 *m/z*. . Calibration was done automatically before every LC-MS run by injection of a basic sodium formate solution through a filled 20 µL loop switched into the LC flow at the beginning of each run. The data were analyzed using Mzmine version 3.9.0. The minimum absolute height was set to $5x10^3$, and the number of minimum consecuetive scans was set to 7. Features were annotated within 5 ppm error within the calculated mass. The following list was used for feature-annotation:

Table S5: Characterization of MYX derivatives (**1**, **2**, **5**–**19**). N.d.: not determined.

Compound	Chemical formula	Calculated mass *m/z* $[M+H]^+$	Observed mass *m/z* $[M+H]^+$	Retention time [min]
**MYX A (1)**	$C_{23}H_{31}NO_6$	418.2224	418.2225	12.34
**MYX B (2)**	$C_{24}H_{33}NO_6$	432.2381	432.2377	13.25
**5**	$C_{22}H_{30}NO_6$	404.2068	n.d.	n.d.
**6**	$C_{22}H_{30}NO_6$	404.2068	n.d.	n.d.
**7**	$C_{21}H_{28}NO_6$	390.1911	n.d.	n.d.
**8**	$C_{23}H_{28}F_3NO_6$	472.1942	472.1936	12.03
**9**	$C_{24}H_{29}NO_6$	428.2068	428.2059	11.15
**10**	$C_{26}H_{35}NO_6$	458.2537	458.2545	14.08
**11**	$C_{26}H_{30}NO_6$	452.2068	n.d.	n.d.
**12**	$C_{25}H_{29}N_2O_6$	453.2020	n.d.	n.d.
**13**	$C_{24}H_{27}NO_7$	442.1860	442.1861	11.43
**14**	$C_{24}H_{27}NO_7$	442.1860	442.1862	11.17
**15**	$C_{24}H_{27}NO_6S$	458.1632	458.1636	11.91
**16**	$C_{25}H_{30}N_2O_7$	443.1813	443.1814	9.56
**17**	$C_{26}H_{32}N_2O_7$	457.1969	457.1971	9.92
**18**	$C_{25}H_{30}N_2O_6S$	459.1584	459.1584	10.16
**19**	$C_{26}H_{32}N_2O_6S$	473.1741	473.1736	10.84

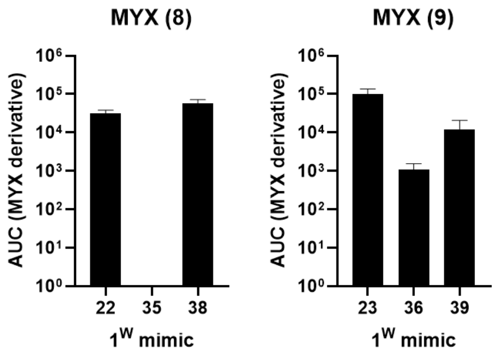

Figure S2: Additional small-scale experiments. Δ*CP-1*^{W} mutant supplemented for production of analog **8** with respective mutasynthons **22**,**35** and **38** and for production of analog **9** with respective precursors **22**,**36** and **39**.

Production in fermenter system

All bioreactor cultivations were performed with the parallel cultivation system DASGIP (Eppendorf) and DASware control (version 5.6.4) for process control. The vessels for microbial application had a total volume of 1.8 L and were equipped with two impellers (Rushton-type). The system was fitted with optical DO (Hamilton) and pH probe (Hamilton). The off-gas analyzer consisted of zirconium dioxide sensors for O_2 measurement and infrared sensors for CO_2 measurement (BlueSens). The DO concentration in the medium was kept at a constant level of 15, 20, 30, 45 or 80 % by increasing the stirrer speed (150 – 600 rpm), gas flow rate (0.16 – 0.33 vvm) and change of the gas composition (0 – 21% oxygen) if necessary, realized by an internal control cascade. The pH: 7.4 value was kept constant by adding 1M H_2SO_4 and 1M KOH during fermentation, if not stated otherwise. An internal control cascade added antifoam SE-15 (Sigma-Aldrich) when foaming occurred, using a peristaltic pump (speed 40 mL/h). All fermentations were carried out with a cultivation volume of 1 L for 172 h at 15, 20, 25, 30 and 35 °C. After inoculation, feeding of the precursor **22** was either performed in one batch after 48 h or with increasing amounts from 7.5 to 15, 30 and 47.5 % of the overall feeding amount after 24, 48, 72 and 96 h. The final concentration for all experiments was 1 mM.

Table S6: Control range of N (agitation), F (gas flow rate) and XO_2 (gas composition) with interaction of X_1 and X_2 of internal dissolved oxygen cascade. The Y values indicated the minimum and maximum range of the corresponding parameters N, F and XO_2.

DO cascade parameter	X_1	X_2	Y_1	Y_2
**N**	0%	40%	150 rpm	600 rpm
**F**	80%	100%	10 sL/h	20 sL/h
**XO_2**	0%	80%	0%	21%

Chemistry

All air- or moisture-sensitive reactions were carried out in dried glassware (>100 °C) under an atmosphere of nitrogen or argon. Dried solvents were distilled before use. Analytical TLC was performed on precoated silica gel plates (Macherey-Nagel, Polygram SIL G/UV254). Visualization was accomplished with UV-light, $KMnO_4$, or a ceric ammonium molybdate chamber. The products were purified by flash chromatography on silica gel columns (Macherey-Nagel 60, 0.04–0.063 mm). Preparative high–performance liquid chromatography (HPLC) was performed on an Autopurifier System (APS) (Waters) with a XBridge C18 column (100 × 19 mm, particle size 5 μm, 130 Å pore size, Waters) for preparative separation, at a flowrate of 25 mL/min. 1/30 of the flow was split into a low-resolution mass spectrometer (Waters, QDa) Desired fractions were obtained by time triggered fractionation after initial gradient optimization. ^{1}H and ^{13}C spectra were recorded with a AV 500 [500 MHz (^{1}H), 126 MHz (^{13}C)] spectrometer (Bruker BioSpin) in $CDCl_3$, DMSO-d_6, or MeOH-d_4 unless otherwise specified. Chemical shifts are given in parts per million (ppm) and referenced against the residual proton or carbon resonances of the >99% deuterated solvents as internal standard. Coupling constants (J) are given in hertz (Hz). Data are reported as follows: chemical shift, multiplicity (s = singlet, d = doublet, t = triplet, q = quartet, m = multiplet, dd = doublet of doublets, dt = doublet of triplets, br = broad, and combinations of these) coupling constants, and integration. NMR spectra were evaluated using ACDLabs 2019. Liquid chromatography–mass spectrometry (LC–MS) was performed on a LC–MS system consisting of a Dionex UltiMate 3000 pump, autosampler, column compartment, detector (Thermo Fisher Scientific), and ESI quadrupole MS (MSQ Plus or ISQ EC, Thermo Fisher Scientific). High-resolution mass was determined by LC–MS/MS using the Q Exactive Focus Orbitrap LC–MS/MS system (Thermo Fisher Scientific). The purity of the final compounds was determined by LC–MS using a gradient with (A) H_2O + 0.1% FA to (B) ACN + 0.1% FA at a flow rate of 600 μL/min and 45 °C. The gradient was initiated by a 1 min isocratic step at 5% B followed by an increase to 99% B in 15 min to end up with a 5 min step at 99% B before re-equilibration under the initial conditions. The purity of the final compounds was determined by using the area percentage method on the UV trace recorded at a wavelength of 254 nm and found to be >95%.

General procedure A (GP A): Copper-catalyzed 1,4-addition to ethyl-2-butynoate

To a suspension of $CuBrSMe_2$ (1.25 equiv.) in THF (0.75 M), the respective organometallic compound (1.25 equiv.) was slowly added at –45 °C. After stirring for 30 min, the solution was cooled to –78°C and ethyl-2-butynoate (1.0 equiv.) in THF (4.3 M) was added dropwise.[7] After stirring at this temperature for 2 h, the reaction was quenched with saturated NH_4Cl solution. After stirring at room temperature for 10 min, the reaction mixture was diluted with water and was extracted with Et_2O (3x). The combined organic phases were washed with saturated aq.

NaCl solution, dried over Na_2SO_4, filtered and concentrated *in vacuo* to yield in the desired product without further purification unless otherwise specified.

General procedure B (GP B): DibalH reduction & *in situ* alcohol oxidation–Wittig reaction

To a solution of ester (1.0 equiv.) in DCM (0.1 M), DibalH (3.0 equiv., 1 M in DCM) was added dropwise at –78 °C.[8] After complete conversion of the starting material (TLC), the reaction mixture was quenched by the addition of saturated aqueous disodium tartrate and warmed to room temperature. It was then diluted with DCM and vigorously stirred for an additional 2 h. The aqueous phase was extracted with DCM (3x), and the combined organic phases were washed with brine, dried over Na_2SO_4, filtered and concentrated *in vacuo*. The crude product was used without further purification for the next step. At room temperature, activated manganese dioxide (10.0 equiv.) was added in 5 portions over 5 h to a solution of alcohol (1.0 equiv.) and respective Wittig reagent (1.3 equiv.) in DCM (0.02M). After stirring the resulting mixture for 19 h, the manganese dioxide was removed by filtration through celite®, washed with DCM and the combined organic phases were concentrated *in vacuo*. The crude product was purified by flash column chromatography to afford the diene.

General procedure C (GP C): NaOH-mediated saponification

To a solution of ethyl ester (1.0 equiv.) in MeOH/H_2O (3:1, 0.2 M), NaOH (4.6 equiv., 1 M in H_2O) was added at 0 °C.[9] After complete conversion of the starting material (TLC), the solvent was evaporated, and the residue was dissolved in H_2O and cooled down to 0 °C. After acidification with HCl (1M) solution, the aqueous phase was extracted with EtOAc (3x). The combined organic phases were dried over Na_2SO_4, filtered and concentrated *in vacuo* to yield the carboxylic acid without further purification.

General procedure D (CP D): SNAC-esters formation from carboxylic acids

To a mixture of carboxylic acid (1.0 equiv.), EDC·HCl (1.0 equiv.), DMAP (0.1 equiv.) and DIPEA (1.0 equiv.) in DCM (0.5 M), *N*-acetyl cysteamine (1.0 equiv.) was added dropwise at 0 °C. The reaction mixture was stirred overnight and diluted with saturated NH_4Cl solution followed by the extraction with DCM (3x). The combined organic layers were washed with saturated $NaHCO_3$ solution, $KHSO_4$ (1N) solution and brine, dried over Na_2SO_4, filtered and concentrated *in vacuo*. The crude product was purified by flash column chromatography to obtain the desired SNAC-ester.

General procedure E (GP E): SNAC-ester formation from acyl chloride

To a solution of *N*-acetyl cysteamine (1.0 equiv.) and DIPEA (1.1 equiv.) in DCM (0.25 M), the corresponding acyl chloride (1.0 equiv.) was added dropwise at 0 °C. The reaction mixture was stirred overnight and diluted with saturated NH_4Cl solution followed by the extraction with DCM (3x). The combined organic layers were washed with saturated $NaHCO_3$ solution, $KHSO_4$ (1 N) solution and brine, dried over Na_2SO_4, filtered and concentrated *in vacuo* to furnish the desired compound without further purification.

***S*-(2-Acetamidoethyl) butanethioate (20)**

According to **GP E**, *N*-acetyl cysteamine (238.0 mg, 2.0 mmol) and DIPEA (348.0 µL, 2.2 mmol) in DCM (8.0 mL) were reacted with butyryl chloride (213.0 mg, 2.0 mmol) to give the desired SNAC-ester **20** (274.0 mg, 1.45 mmol, 72%) as a colorless oil.

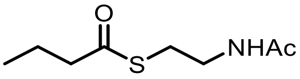

TLC: R_f(**20**) = 0.70 (DCM/EtOAc = 50:50). ^{1}H NMR (500 MHz, $CDCl_3$) δ: 0.96(t, *J*=7.32 Hz, 3 H), 1.64 - 1.75 (m, 2 H), 2.01 (s, 3 H), 2.56 (t, *J*=7.40 Hz, 2 H), 3.04 (t, *J*=6.41 Hz, 2 H), 3.45 (q, *J*=5.80 Hz, 2 H), 6.19 (br s, 1 H). ^{13}C NMR (126 MHz, $CDCl_3$) δ: 13.4, 19.2, 23.0, 28.3, 39.9, 45.9, 170.7, 200.1. HRMS(ESI+) m/z calculated for $C_8H_{16}NO_2S$ $[M+H]^+$ 190.0896; found, 190.0892.

***S*-(2-Acetamidoethyl) pentanethioate (21)**

According to **GP E**, *N*-acetyl cysteamine (238.0 mg, 2.0 mmol) and DIPEA (348.0 µL, 2.2 mmol) in DCM (8.0 mL) were reacted with valeryl chloride (241.2 mg, 2.0 mmol) to give the desired SNAC-ester **21** (387.7 mg, 1.91 mmol, 95%) as a colorless oil.

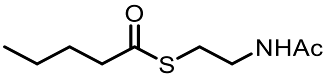

TLC: R_f(**21**) = 0.50 ($CHCl_3$/MeOH = 90:10). ^{1}H NMR (500 MHz, $CDCl_3$) δ: 0.92 (t, *J*=7.30 Hz, 3 H), 1.36 (sxt, *J*=7.60 Hz, 2 H), 1.65 (quin, *J*=6.90 Hz, 3 H), 1.97 (s, 3 H), 2.58 (t, *J*=7.80 Hz, 2 H), 3.02 (t, *J*=6.30 Hz, 2 H), 3.43 (q, *J*=6.10 Hz, 2 H), 5.89 (br s, 1 H). ^{13}C NMR (126 MHz, $CDCl_3$) δ: 13.7, 22.1, 23.2, 27.7, 28.4, 39.8, 43.8, 170.2, 200.3. HRMS(ESI+) m/z calculated for $C_9H_{18}NO_2S$ $[M+H]^+$ 204.1053; found, 204.1057.

***S*-(2-Acetamidoethyl) 4,4,4-trifluorobutanethioate (22)**

According to **GP D**, 4,4,4-trifluorobutanoic acid (0.28 g, 2.0 mmol) was reacted with EDC·HCl (0.38 g, 2.0 mmol), DMAP (24.0 mg, 0.2 mmol), DIPEA (0.33 mL, 2.0 mmol) and *N*-acetylcysteamine (0.24 g, 2.0 mmol) in DCM (4.0 mL). Purification by flash column

chromatography (SiO_2, $CHCl_3$/MeOH = 90:10) gave the desired SNAC-ester **22** (0.32 g, 1.29 mmol, 65%) as a colorless oil.

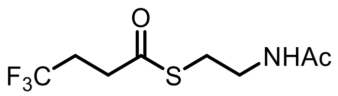

TLC: R_f(**22**) = 0.40 ($CHCl_3$/MeOH = 90:10). ^{1}H NMR (500 MHz, $CDCl_3$) δ: 1.98 (s, 3 H), 2.45 - 2.55 (m, 2 H), 2.83 - 2.88 (m, 2 H), 3.08 (t, *J*=6.48 Hz, 2 H), 3.45 (q, *J*=6.26 Hz, 2 H), 5.76 (br s, 1 H). ^{13}C NMR (126 MHz, $CDCl_3$) δ: 23.13, 28.76, 29.2 (q, *J*=30.33 Hz), 36.2 (q, *J*=2.76 Hz), 39.31, 125.1 (q, *J*=286,8 Hz), 170.3, 196.6. ^{19}F NMR (470 MHz, $CDCl_3$) δ: -66.64 (t, *J*=10.40 Hz). HRMS(ESI+) m/z calculated for $C_8H_{13}F_3NO_3S$ $[M+H]^+$ 244.0614; found, 244.0618.

_S_-(2-Acetamidoethyl) pent-4-ynethioate (23)

According to **GP D**, pent-4-ynoic acid (0.19 g, 2.00 mmol) was reacted with EDC·HCl (0.38 g, 2.00 mmol), DMAP (24.0 mg, 0.20 mmol), DIPEA (0.33 mL, 2.00 mmol) and *N*-acetylcysteamine (0.24 g, 2.00 mmol) in DCM (4.0 mL). Purification by flash column chromatography (SiO_2, $CHCl_3$/MeOH = 95:5) gave the desired SNAC-ester **23** (0.20 g, 1.03 mmol, 52%) as a colorless oil.

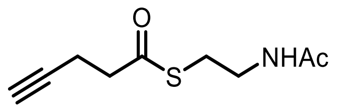

TLC: R_f(**23**) = 0.40 ($CHCl_3$/MeOH = 90:10). ^{1}H NMR (500 MHz, $CDCl_3$) δ: 1.97 (s, 3 H), 2.00 (t, *J*=2.59 Hz, 1 H), 2.55 (td, *J*=7.21, 2.67 Hz, 2 H), 2.81 (t, *J*=7.25 Hz, 2 H), 3.07 (t, *J*=6.33 Hz, 2 H), 3.45 (q, *J*=6.10 Hz, 2 H), 5.88 (br s, 1 H). ^{13}C NMR (126 MHz, $CDCl_3$) δ: 14.6, 23.2, 28.6, 39.5, 42.4, 69.5, 81.7, 170.3, 197.9. HRMS(ESI+) m/z calculated for $C_9H_{13}NO_2S$ $[M+H]^+$ 200.0740; found, 200.0743.

_S_-(2-Acetamidoethyl) cyclohexanecarbothioate (24)

According to **GP D**, cyclohexanecarboxylic acid (0.26 g, 2.00 mmol) was reacted with EDC·HCl (0.38 g, 2.00 mmol), DMAP (24.0 mg, 0.20 mmol), DIPEA (0.33 mL, 2.00 mmol) and *N*-acetylcysteamine (0.24 g, 2.00 mmol) in DCM (4.0 mL). Purification by flash column chromatography (SiO_2, $CHCl_3$/MeOH = 95:5) gave the desired SNAC-ester **24** (0.19 g, 0.82 mmol, 41%) as a colorless oil.

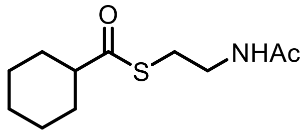

TLC: R_f(**24**) = 0.48 ($CHCl_3$/MeOH = 90:10). ^{1}H NMR (500 MHz, $CDCl_3$) δ: 1.10 - 1.27 (m, 3 H) 1.39 (qd, *J*=12.18, 2.98 Hz, 2 H) 1.60 (br dd, *J*=11.60, 3.51 Hz, 1 H) 1.69 - 1.76 (m, 2 H) 1.84 (br d, *J*=13.12 Hz, 2 H) 1.91 (s, 3 H) 2.44 (tt, *J*=11.50, 3.53 Hz, 1 H) 2.94 (t, *J*=6.33 Hz, 2 H) 3.37 (q, *J*=5.95 Hz, 2 H) 5.87 (br s, 1 H). ^{13}C NMR (126 MHz, $CDCl_3$) δ: 22.9, 25.4, 25.6, 27.9, 29.6, 40.1, 52.7, 171.0, 203.9. HRMS(ESI+) m/z calculated for $C_{11}H_{20}NO_2S$ $[M+H]^+$ 230.1209; found, 230.1219.

S-(2-Acetamidoethyl) benzothioate (25)

According to **GP E**, *N*-acetyl cysteamine (238.0 mg, 2.0 mmol) and DIPEA (348.0 µL, 2.2 mmol) in DCM (8.0 mL) were reacted with benzoyl chloride (281.0 mg, 2.0 mmol) to give the desired SNAC-ester **25** (406.4 mg, 1.82 mmol, 91%) as a colorless oil.

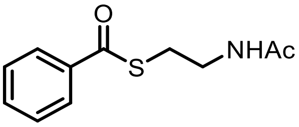

TLC: R_f(**25**) = 0.65 ($CHCl_3$/MeOH = 95:5). ^{1}H NMR (500 MHz, $CDCl_3$) δ: 2.02 (s, 3 H), 3.25 (t, *J*=6.41 Hz, 2 H), 3.48 - 3.62 (m, 2 H), 6.23 (br s, 1 H), 7.43 - 7.51 (m, 2 H), 7.57 - 7.64 (m, 1 H), 7.97 (d, *J*=7.53 Hz, 2 H). ^{13}C NMR (126 MHz, $CDCl_3$) δ: 22.9, 28.4, 39.9, 127.3, 128.71, 133.8, 136.6, 171.0, 192.2. HRMS(ESI+) m/z calculated for $C_{11}H_{14}NO_2S$ $[M+H]^+$ 224.0740; found, 224.0745.

S-(2-Acetamidoethyl) pyridine-3-carbothioate (26)

According to **GP D**, nicotinic acid (0.23 g, 2.00 mmol) was reacted with EDC·HCl (0.38 g, 2.00 mmol), DMAP (24.0 mg, 0.20 mmol), DIPEA (0.33 mL, 2.00 mmol) and *N*-acetylcysteamine (0.24 g, 2.00 mmol) in DCM (4.0 mL). Purification by flash column chromatography (SiO_2, $CHCl_3$/MeOH = 95:5) gave the desired SNAC-ester **26** (0.29 g, 1.32 mmol, 66%) as a colorless oil.

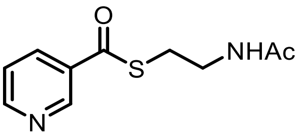

TLC: R_f(**26**) = 0.31 ($CHCl_3$/MeOH = 90:10). ^{1}H NMR (500 MHz, $CDCl_3$) δ: 2.00 (s, 3 H), 3.29 (t, *J*=6.41 Hz, 2 H), 3.56 (q, *J*=6.26 Hz, 2 H), 5.88 (br s, 1 H), 7.43 (dd, *J*=8.09, 4.88 Hz, 1 H), 8.22 (dt, *J*=7.93, 1.98 Hz, 1 H), 8.82 (dd, *J*=4.81, 1.60 Hz, 1 H), 9.19 (d, *J*=1.98 Hz, 1 H). ^{13}C NMR (126 MHz, $CDCl_3$) δ: 23.2, 28.7, 39.5, 123.6, 132.3, 134.6, 148.5, 154.1, 170.3, 190.8. HRMS(ESI+) m/z calculated for $C_{10}H_{13}N_2O_2S$ $[M+H]^+$ 225.0692; found, 225.0692.

S-(2-Acetamidoethyl) furan-2-carbothioate (27)

According to **GP D**, furan-2-carboxylic acid (0.22 g, 2.00 mmol) was reacted with EDC·HCl (0.38 g, 2.00 mmol), DMAP (24.0 mg, 0.20 mmol), DIPEA (0.33 mL, 2.00 mmol) and *N*-acetylcysteamine (0.24 g, 2.00 mmol) in 4.0 mL DCM. Purification by flash column chromatography (SiO_2, $CHCl_3$/MeOH = 95:5) gave the desired SNAC-ester **27** (0.21 g, 1.00 mmol, 50%) as a colorless oil.

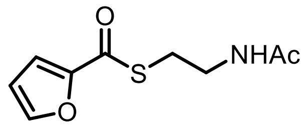

TLC: R_f(**27**) = 0.37 ($CHCl_3$/MeOH = 90:10). ^1H NMR (500 MHz, $CDCl_3$) δ: 1.98 (s, 3 H), 3.22 (t, *J*=6.33 Hz, 2 H), 3.52 (q, *J*=6.10 Hz, 2 H), 6.00 (br s, 1 H), 6.56 (dd, *J*=3.59, 1.60 Hz, 1 H), 7.22 (d, *J*=3.51 Hz, 1 H) 7.60 (s, 1 H). ^{13}C NMR (126 MHz, $CDCl_3$) δ: 23.2, 27.8, 39.7, 112.4, 116.1, 146.5, 150.5 170.3, 180.7. HRMS(ESI+) m/z calculated for $C_9H_{12}NO_3S$ $[M+H]^+$ 214.0532; found, 214.0539.

S-(2-Acetamidoethyl) furan-3-carbothioate (28)

According to **GP D**, furan-3-carboxylic acid (0.22 g, 2.00 mmol) was reacted with EDC·HCl (0.38 g, 2.00 mmol), DMAP (24.0 mg, 0.20 mmol), DIPEA (0.33 mL, 2.00 mmol) and *N*-acetylcysteamine (0.24 g, 2.00 mmol) in DCM (4.0 mL). Purification by flash column chromatography (SiO_2, $CHCl_3$/MeOH = 95:5) gave the desired SNAC-ester **28** (0.27 g, 1.28 mmol, 64%) as a colorless oil.

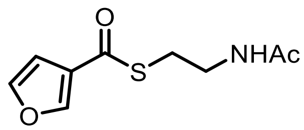

TLC: R_f(**28**) = 0.44 ($CHCl_3$/MeOH = 90:10). ^1H NMR (500 MHz, $CDCl_3$) δ: 1.98 (s, 3 H) 3.20 (t, *J*=6.41 Hz, 2 H) 3.52 (q, *J*=6.10 Hz, 2 H) 5.93 (br s, 1 H) 6.76 (dd, *J*=1.98, 0.76 Hz, 1 H) 7.47 (t, *J*=1.68 Hz, 1 H) 8.08 - 8.11 (m, 1 H). ^{13}C NMR (126 MHz, $CDCl_3$) δ: 23.2, 28.1, 39.8, 108.2, 126.7, 144.2, 146.0, 170.3, 185.4. HRMS(ESI+) m/z calculated for $C_9H_{12}NO_3S$ $[M+H]^+$ 214.0532; found, 214.0536.

S-(2-Acetamidoethyl) thiophene-3-carbothioate (29)

According to **GP D**, thiophene-3-carboxylic acid (0.26 g, 2.00 mmol) was reacted with EDC·HCl (0.38 g, 2.00 mmol), DMAP (24.0 mg, 0.20 mmol), DIPEA (0.33 mL, 2.00 mmol) and *N*-acetylcysteamine (0.24 g, 2.00 mmol) in DCM (4.0 mL). Purification by flash column

chromatography (SiO_2, $CHCl_3$/MeOH = 95:5) gave the desired SNAc-ester **29** (0.39 g, 1.72 mmol, 86%) as a colorless oil.

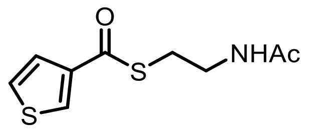

TLC: R_f(**29**) = 0.48 ($CHCl_3$/MeOH = 90:10). ^{1}H NMR (500 MHz, $CDCl_3$) δ: 1.99 (s, 3 H), 3.22 (t, *J*=6.33 Hz, 2 H), 3.54 (q, *J*=6.00 Hz, 2 H), 5.91 (br s, 1 H), 7.36 (dd, *J*=5.11, 2.98 Hz, 1 H), 7.54 (dd, *J*=5.11, 1.30 Hz, 1 H), 8.14 (dd, *J*=2.90, 1.22 Hz, 1 H). ^{13}C NMR (126 MHz, CHLOROFORM-*d*) δ ppm 23.3, 28.5, 30.9, 39.8, 126.0, 126.7, 131.0, 140.5, 170.3, 185.8. HRMS(ESI+) m/z calculated for $C_9H_{12}NO_2S_2$ $[M+H]^+$ 230.0304; found, 230.0304.

***S*-(2-Acetamidoethyl) oxazole-4-carbothioate (30)**

According to **GP D**, oxazole-4-carboxylic acid (0.23 g, 2.00 mmol) was reacted with EDC·HCl (0.38 g, 2.00 mmol), DMAP (24.0 mg, 0.20 mmol), DIPEA (0.33 mL, 2.00 mmol) and *N*-acetylcysteamine (0.24 g, 2.00 mmol) in DCM (4.0 mL). Purification by flash column chromatography (SiO_2, $CHCl_3$/MeOH = 95:5) gave the desired SNAC-ester **30** (0.13 g, 0.62 mmol, 31%) as a colorless oil.

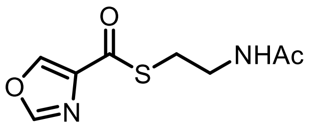

TLC: R_f(**30**) = 0.33 ($CHCl_3$/MeOH = 90:10). ^{1}H NMR (500 MHz, $CDCl_3$) δ: 1.98 (s, 3 H), 3.23 (t, *J*=6.33 Hz, 2 H), 3.54 (q, *J*=6.15 Hz, 2 H), 5.88 (br s, 1 H), 7.94 (d, *J*=0.76 Hz, 1 H), 8.27 (d, *J*=0.92 Hz, 1 H). ^{13}C NMR (126 MHz, $CDCl_3$) δ: 23.2, 28.1, 39.4, 139.0, 140.3, 151.1, 170.3,185.5. HRMS(ESI+) m/z calculated for $C_8H_{11}N_2O_3S$ $[M+H]^+$ 215.0485; found, 215.0493.

***S*-(2-Acetamidoethyl) 2-methyloxazole-4-carbothioate (31)**

According to **GP D**, 2-methyloxazole-4-carboxylic acid (0.25 g, 2.00 mmol) was reacted with EDC·HCl (0.38 g, 2.00 mmol), DMAP (24.0 mg, 0.20 mmol), DIPEA (0.33 mL, 2.00 mmol) and *N*-acetylcysteamine (0.24 g, 2.00 mmol) in DCM (4.0 mL). Purification by flash column chromatography (SiO_2, $CHCl_3$/MeOH = 95:5) gave the desired SNAC-ester **31** (0.22 g, 0.95 mmol, 47%) as a colorless oil.

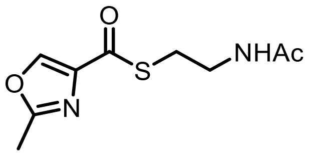

TLC: R_f(**31**) = 0.33 ($CHCl_3$/MeOH = 90:10). ^1H NMR (500 MHz, $CDCl_3$) δ: 1.97 (s, 3 H), 2.54 (s, 3 H), 3.21 (t, *J*=6.26 Hz, 2 H), 3.52 (q, *J*=5.95 Hz, 2 H), 5.90 (br s, 1 H), 8.12 (s, 1 H). ^{13}C NMR (126 MHz, $CDCl_3$) δ: 13.9, 23.2, 24.7, 28.0, 39.5, 139.3, 140.0, 162.3, 170.2, 185.5. HRMS(ESI+) m/z calculated for $C_9H_{13}N_2O_3S$ $[M+H]^+$ 229.0641; found, 229.0646.

***S*-(2-Acetamidoethyl) thiazole-4-carbothioate (32)**

According to **GP D**, thiazole-4-carboxylic acid (0.26 g, 2.00 mmol) was reacted with EDC·HCl (0.38 g, 2.00 mmol), DMAP (24.0 mg, 0.20 mmol), DIPEA (0.33 mL, 2.00 mmol) and *N*-acetylcysteamine (0.24 g, 2.00 mmol) in DCM (4.0 mL). Purification by flash column chromatography (SiO_2, $CHCl_3$/MeOH = 95:5) gave the desired SNAC-ester **32** (0.27 g, 1.17 mmol, 59%) as a colorless oil.

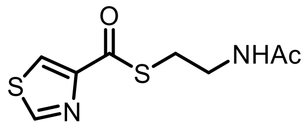

TLC: R_f(**32**) = 0.42 ($CHCl_3$/MeOH = 90:10). ^1H NMR (500 MHz, $CDCl_3$) δ: 1.98 (s, 3 H), 3.25 (t, *J*=6.33 Hz, 2 H), 3.55 (q, *J*=6.05 Hz, 2 H), 5.93 (br s, 1 H), 8.20 (d, *J*=2.14 Hz, 1 H), 8.87 (d, *J*=1.98 Hz, 1 H). ^{13}C NMR (126 MHz, $CDCl_3$) δ: 23.3, 28.6, 39.5, 123.3, 153.3, 153.4, 170.2, 186.1. HRMS(ESI+) m/z calculated for $C_8H_{11}N_2O_2S_2$ $[M+H]^+$ 231.0256; found, 231.0256.

***S*-(2-Acetamidoethyl) 2-methylthiazole-4-carbothioate (33)**

According to **GP D**, 2-methylthiazole-4-carboxylic acid (0.29 g, 2.00 mmol) was reacted with EDC·HCl (0.38 g, 2.00 mmol), DMAP (24.0 mg, 0.20 mmol), DIPEA (0.33 mL, 2.00 mmol) and *N*-acetylcysteamine (0.24 g, 2.00 mmol) in DCM (4.0 mL). Purification by flash column chromatography (SiO_2, $CHCl_3$/MeOH = 95:5) gave the desired SNAC-ester **33** (0.28 g, 1.16 mmol, 58%) as a colorless oil.

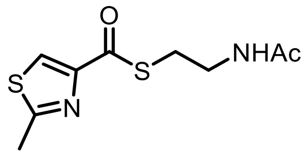

TLC: R_f(**33**) = 0.44 ($CHCl_3$/MeOH = 90:10). ^1H NMR (500 MHz, $CDCl_3$) δ: 1.97 (s, 3 H), 2.79 (s, 3 H), 3.22 (t, *J*=6.26 Hz, 2 H), 3.54 (q, *J*=6.05 Hz, 2 H), 5.91 (br s, 1 H), 7.99 (s, 1 H). ^{13}C NMR (126 MHz, $CDCl_3$) δ: 19.4, 23.3, 28.5, 39.5, 123.4, 124.1, 152.1, 167.0, 170.2, 186.0. HRMS(ESI+) m/z calculated for $C_9H_{13}N_2O_2S_2$ $[M+H]^+$ 245.0413; found, 245.0422.

S-Phenyl butanethioate (34)

To a solution of thiophenol (220.4 mg, 2.0 mmol) and DIPEA (348.0 µL, 2.2 mmol) in DCM (8.0 mL), butyryl chloride (213.0 mg, 2.0 mmol) was added dropwise at 0 °C. The reaction mixture was stirred overnight and diluted with saturated NH_4Cl solution followed by the extraction with DCM (3x). The combined organic layers were washed with saturated $NaHCO_3$ solution, $KHSO_4$ (1 N) solution and saturated aq. NaCl solution, dried over Na_2SO_4, filtered and concentrated *in vacuo* to furnish the desired compound **34** (355.1 mg, 1.97 mmol, 98%) without further purification.

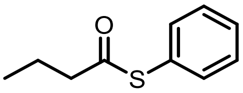

TLC: R_f(**34**) = 0.66 ($CHCl_3$/MeOH = 95:5). ^{1}H NMR (500 MHz, $CDCl_3$) δ: 1.01 (t, *J*=7.40 Hz, 3 H), 1.76 (sxt, *J*=7.39 Hz, 2 H), 2.65 (t, *J*=7.32 Hz, 2 H), 7.36 - 7.46 (m, 5 H). ^{13}C NMR (126 MHz, CHLOROFORM-*d*) δ ppm 13.5, 19.1, 45.5, 127.9, 129.1, 129.3, 134.5, 197.4. HRMS(ESI+) m/z calculated for $C_{10}H_{13}O_2S$ $[M+H]^+$ 181.0682; found, 181.0692.

S-Phenyl 4,4,4-trifluorobutanethioate (35)

To a solution of thiophenol (220.4 mg, 2.0 mmol) and DIPEA (348.0 µL, 2.2 mmol) in DCM (8.0 mL), 4,4,4-trifluorobutanoyl chloride (321.0 mg, 2.0 mmol) was added dropwise at 0 °C. The reaction mixture was stirred overnight and diluted with saturated NH_4Cl solution followed by the extraction with DCM (3x). The combined organic layers were washed with saturated $NaHCO_3$ solution, $KHSO_4$ (1 N) solution and saturated aq. NaCl solution, dried over Na_2SO_4, filtered and concentrated *in vacuo* to furnish the desired thioester **35** (186.0 mg, 0.79 mmol, 40%) without further purification.

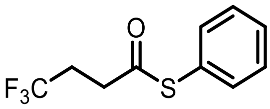

TLC: R_f(**35**) = 0.79 ($CHCl_3$/MeOH = 95:5). ^{1}H NMR (500 MHz, $CDCl_3$) δ: 2.39 - 2.50 (m, 2 H) 2.84 - 2.89 (m, 2 H) 7.33 - 7.39 (m, 5 H). ^{13}C NMR (126 MHz, $CDCl_3$) δ: 29.4 (q, *J*=30.33 Hz), 35.8 (q, *J*=2.76 Hz), 126.3 (q, *J*=275.76 Hz), 126.7, 129.4, 129.8, 134.6, 194.8. ^{19}F NMR (470 MHz, $CDCl_3$) δ: -66.61 (t, *J*=10.40 Hz). HRMS(ESI+) m/z calculated for $C_{10}H_{10}F_3OS$ $[M+H]^+$ 235.0399; found, 235.0420.

S-Phenyl pent-4-ynethioate (36)

To a solution of 4-pentynoic acid (400.0 mg, 4.08 mmol) in DCM (8.0 mL), thiophenol (493.6 mg, 4.48 mmol), EDC·HCl (859.8 mg, 4.48 mmol), DMAP (49.8 mg 0.41 mmol) and DIPEA

(782.5 µL, 4.48 mmol) were added at 0 °C. The reaction mixture was stirred overnight and diluted with saturated NH_4Cl solution followed by the extraction with DCM (3x). The combined organic phases were washed with saturated $NaHCO_3$ solution, $KHSO_4$ (1 N) solution and saturated aq. NaCl solution, dried over Na_2SO_4, filtered and concentrated *in vacuo*. Flash chromatography (SiO_2, DCM/MeOH = 98:2) afforded the desired product **36** (300.0 mg, 1.58 mmol, 39%) as a yellowish oil.

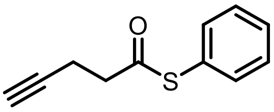

TLC: R_f(**36**) = 0.80 ($CHCl_3$/MeOH = 95:5). ^{1}H NMR (500 MHz, $CDCl_3$) δ: 2.03 (t, *J* = 2.67 Hz, 1 H) 2.59 (td, *J* = 7.40, 2.75 Hz, 2 H) 2.91 (t, *J* = 7.40 Hz, 2 H) 7.43 (s, 5 H). ^{13}C NMR (126 MHz, $CDCl_3$) δ: 14.6, 42.0, 69.5, 81.8, 129.2, 129.5, 134.5, 195.6. HRMS(ESI+) m/z calculated for $C_{11}H_{11}OS$ $[M+H]^+$ 191.0525; found, 191.0529.

(*R*)-*N*-(3-((2-Mercaptoethyl)amino)-3-oxopropyl)-2,2,5,5-tetramethyl-1,3-dioxane-4-carboxamide (A)[1]

To a solution of (*R*)-3-(2,2,5,5-tetramethyl-1,3-dioxane-4-carboxamido)propanoic acid (5.05 g, 19.47 mmol) in DCM/DMF (100 mL, 8:2), cysteamine hydrochloride (4.36 g, 38.36 mmol), PyBOP (15.2 g, 29.20 mmol) and DIPEA (16.9 mL, 94.82 mmol) were added at 0 °C. The reaction mixture was warmed up room temperature overnight and was diluted with DCM. After successively washing with sat. NH_4Cl solution, water, sat. $NaHCO_3$ solution, $KHSO_4$ (1 N) solution and sat. aq. NaCl solution, the organic phase was dried over anhydrous Na_2SO_4 and the solvent was removed under reduced pressure. Flash chromatography afforded the desired product **A** (2.28 g, 7.16 mmol, 37%) as colorless oil.

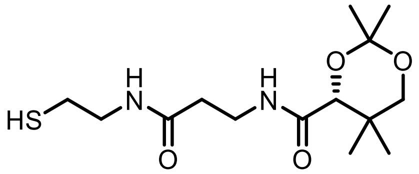

TLC: R_f(**A**) = 0.22 (EtOAc). ^{1}H NMR (500 MHz, $CDCl_3$) δ: 0.96 (s, 3 H), 1.02 (s, 3 H), 1.38 (t, *J*=8.47 Hz, 1 H), 1.41 (s, 3 H), 1.45 (s, 3 H), 2.46 (t, *J*=6.10 Hz, 2 H), 2.58 - 2.70 (m, 2 H), 3.27 (d, *J*=11.75 Hz, 1 H), 3.37 - 3.60 (m, 4 H), 3.67 (d, *J*=11.60 Hz, 1 H), 4.07 (s, 1 H), 6.48 (br s, 1 H), 7.03 (br s, 1 H). ^{13}C NMR (126 MHz, $CDCl_3$) δ: 18.6, 18.9, 22.1, 24.5, 29.4, 32.9, 34.8, 36.1, 42.4, 71.3, 77.1, 99.1, 170.2, 171.1. HRMS(ESI+) m/z calculated for $C_{14}H_{27}N_2O_4S$ $[M+H]^+$ 319.1686; found, 319.1689.

(*R*)-*S*-(2-(3-(2,2,5,5-Tetramethyl-1,3-dioxane-4-carboxamido)propanamido)ethyl) butanethioate (37a)

To a solution of (*R*)-*N*-(3-((2-mercaptoethyl)amino)-3-oxopropyl)-2,2,5,5-tetramethyl-1,3-dioxane-4-carboxamide **34a** (318.4 mg, 1.0 mmol) and DIPEA (192.0 µL, 1.1 mmol) in DCM (10 mL), butyryl chloride (117.2 mg, 1.1 mmol) were added at 0 °C. The reaction mixture was stirred overnight and diluted with saturated NH_4Cl solution followed by the extraction with DCM (3x). The combined organic phases were washed with saturated $NaHCO_3$ solution, $KHSO_4$ (1 N) solution and saturated aq. NaCl solution, dried over Na_2SO_4, filtered and concentrated *in vacuo*. The crude product was purified by flash column chromatography (SiO_2, DCM/MeOH = 95:5) to obtain the desired compound **37a** (132.5 mg, 0.34 mmol, 34%) as colorless oil.

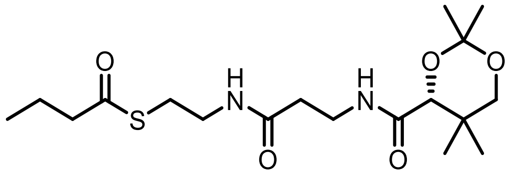

TLC: R_f(**37a**) = 0.20 (DCM/MeOH = 95:5). ^{1}H NMR (500 MHz, $CDCl_3$) δ: 0.95 (d, *J*=7.30 Hz, 3 H) 0.97 (s, 3 H) 1.04 (s, 3 H) 1.42 (s, 3 H) 1.47 (s, 3 H) 1.69 - 1.74 (m, 2 H) 2.43 (t, *J*=6.26 Hz, 2 H) 2.56 (t, *J*=7.40 Hz, 2 H) 3.02 (t, *J*=6.48 Hz, 2 H) 3.28 (d, *J*=11.75 Hz, 1 H) 3.38 - 3.61 (m, 4 H) 3.69 (d, *J*=11.60 Hz, 1 H) 4.08 (s, 1 H) 6.15 (br s, 1 H) 7.03 (br s, 1 H).. ^{13}C NMR (126 MHz, $CDCl_3$) δ: 12.5, 17.7, 17.9, 18.1, 21.1, 27.4, 28.5, 31.9, 33.8, 35.0, 38.6, 44.9, 70.4, 76.1, 98.1, 169.1, 170.1, 198.8. HRMS(ESI+) m/z calculated for $C_{18}H_{33}N_2O_5S$ $[M+H]^+$ 389.2105; found, 389.2108.

(*R*)-*S*-(2-(3-(2,4-Dihydroxy-3,3-dimethylbutanamido)propanamido)ethyl) butanethioate (37)

To a solution of (*R*)-*S*-(2-(3-(2,2,5,5-tetramethyl-1,3-dioxane-4-carboxamido)propanamido)-ethyl) butanethioate **37a** (130.1 mg, 0.34 mmol) in THF (3.4 mL), HCl (1 M) solution (0.34 mmol) was added at 0 °C. After full conversion of the starting material (LC-MS), the reaction mixture was diluted with DCM. The organic phase was washed with saturated aq. NaCl solution, dried over Na_2SO_4, filtered and the solvent was removed under reduced pressure to give compound **37** (80.9 mg, 0.23 mmol, 69%) as a colorless oil without further purification.

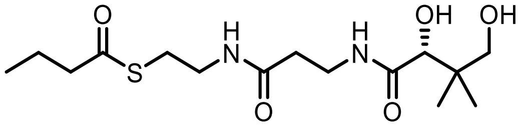

TLC: R_f(**37**) = 0.10 (DCM/MeOH = 95:5). ^{1}H NMR (500 MHz, $CDCl_3$) δ: 0.86 (s, 3 H), 0.89 (t, *J*=7.40 Hz, 3 H), 0.96 (s, 3 H) 1.61 - 1.65 (m, 2 H) 2.35 (t, *J*=6.30 Hz, 2 H), 2.49 (t, *J*=7.40 Hz, 2 H) 2.90 - 3.02 (m, 2 H) 3.25 (br s, 1 H) 3.31 (td, *J*=13.28, 5.80 Hz, 1 H) 3.38 - 3.42 (m, 1 H) 3.43 (d, *J*=5.19 Hz, 2 H), 3.48 - 3.50 (m, 1 H), 3.51 - 3.53 (m, 1 H), 3.70 (br s, 1 H) 3.93 (d,

J=2.59 Hz, 1 H) 6.08 (br s, 1 H) 7.28 (br s, 1 H). ^{13}C NMR (126 MHz, $CDCl_3$) δ: 13.4, 19.1, 20.3, 21.6, 28.3, 35.1, 35.5, 39.3, 39.7, 45.9, 70.9, 77.7, 171.6, 173.4, 200.3. HRMS(ESI+) m/z calculated for $C_{18}H_{29}N_2O_5S$ $[M+H]^+$ 349.1792; found, 349.1794.

(*R*)-*S*-(2-(3-(2,2,5,5-Tetramethyl-1,3-dioxane-4-carboxamido)propanamido)ethyl) 4,4,4-trifluorobutanethioate (38a)

To a solution of (*R*)-*N*-(3-((2-mercaptoethyl)amino)-3-oxopropyl)-2,2,5,5-tetramethyl-1,3-dioxane-4-carboxamide **A** (500.0 mg, 1.57 mmol) in DCM (16 mL), 4,4,4-trifluorobutanoic acid (245.5 mg, 1.72 mmol), EDC·HCl (329.7 mg, 1.72 mmol), DMAP (18.3 mg, 0.15 mmol) and DIPEA (679.0 µL, 3.93 mmol) were added at 0 °C. The reaction mixture was stirred overnight and diluted with saturated NH_4Cl solution followed by the extraction with DCM (3x). The combined organic phases were washed with saturated $NaHCO_3$ solution, $KHSO_4$ (1N) solution and saturated aq. NaCl solution, dried over Na_2SO_4, filtered and concentrated *in vacuo*. The crude product was purified by flash column chromatography (SiO_2, DCM/MeOH = 95:5) to obtain the desired compound **38a** (484.7 mg, 1.09 mmol, 70%) as colorless oil.

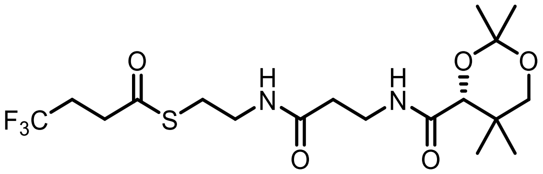

TLC: R_f(**38a**) = 0.25 (DCM/MeOH = 95:5). ^{1}H NMR (500 MHz, $CDCl_3$) δ: 0.97 (s, 3 H), 1.04 (s, 3 H), 1.40 - 1.48 (m, 6 H), 1.77 (s, 1 H), 2.43 (t, *J*=6.18 Hz, 2 H), 2.46 - 2.54 (m, 2 H), 2.82 - 2.87 (m, 2 H), 3.06 (t, *J*=6.56 Hz, 2 H), 3.28 (d, *J*=11.75 Hz, 1 H), 3.38 - 3.62 (m, 4 H), 3.69 (d, *J*=11.75 Hz, 1 H), 4.08 (s, 1 H), 6.24 (br s, 1 H), 7.01 (br t, *J*=5.57 Hz, 1 H). ^{13}C NMR (126 MHz, $CDCl_3$) δ: 18.6, 18.9, 22.1, 28.7, 29.2 (q, *J*=30.33 Hz), 29.4, 32.9, 34.7, 36.0, 36.2 (q, *J*=2.76 Hz), 39.2, 71.4, 77.1, 99.1, 126.3 (q, *J*=276.68 Hz), 170.2, 171.1, 196.3. ^{19}F NMR (470 MHz, $CDCl_3$) δ: -66.64 (t, *J*=10.40 Hz). HRMS(ESI+) m/z calculated for $C_{18}H_{30}F_3N_2O_5S$ $[M+H]^+$ 443.1822; found, 443.1843.

(*R*)-*S*-(2-(3-(2,4-Dihydroxy-3,3-dimethylbutanamido)propanamido)ethyl) 4,4,4-trifluoro-butanethioate (38)

To a solution of (*R*)-*S*-(2-(3-(2,2,5,5-tetramethyl-1,3-dioxane-4-carboxamido)propanamido)-ethyl) 4,4,4-trifluorobutanethioate **38a** (477.0 mg, 1.08 mmol) in THF (10 mL), HCl (1 M) solution (1.08 mmol) was added at 0 °C. After full conversion of the starting material (LC-MS), the reaction mixture was diluted with DCM. The organic phase was washed with saturated aq. NaCl solution, dried over Na_2SO_4, filtered and the solvent was removed under reduced pressure to give compound **38** (374.2 mg, 0.93 mmol, 86%) as a colorless oil without further purification.

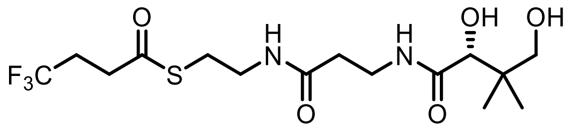

TLC: R_f(**38**) = 0.10 (DCM/MeOH = 95:5). ^{1}H NMR (500 MHz, $CDCl_3$) δ: 0.93 (s, 3 H), 1.00 (s, 3 H), 1.09 (s, 1 H), 1.24 (s, 1 H), 2.44 (t, *J*=5.95 Hz, 2 H), 2.46 - 2.56 (m, 2 H), 2.83 - 2.89 (m, 2 H), 3.02 - 3.11 (m, 2 H), 3.38 - 3.47 (m, 2 H), 3.49 (s, 2 H), 3.56 (q, *J*=5.85 Hz, 2 H), 4.00 (s, 1 H), 6.53 (br t, *J*=5.34 Hz, 1 H). ^{13}C NMR (126 MHz, $CDCl_3$) δ: 20.4, 21.4, 28.6, 29.2 (q, *J*=30.33 Hz), 35.2, 35.6, 36.2 (q, *J*=2.76 Hz), 39.3, 39.3, 40.9, 70.9, 77.5, 126.3 (q, *J*=276.07 Hz), 171.7, 173.7 196.7. ^{19}F NMR (470 MHz, $CDCl_3$) δ: -66.60 (t, *J*=10.40 Hz). HRMS(ESI+) m/z calculated for $C_{15}H_{26}F_3N_2O_5S$ $[M+H]^+$ 403.1509; found, 403.1501.

(*R*)-*S*-(2-(3-(2,2,5,5-tetramethyl-1,3-dioxane-4-carboxamido)propanamido)ethyl) pent-4-ynethioate (39a)

To a solution of (*R*)-*N*-(3-((2-mercaptoethyl)amino)-3-oxopropyl)-2,2,5,5-tetramethyl-1,3-dioxane-4-carboxamide **A** (155.5 mg, 0.49 mmol) in DCM (5 mL), 4-pentynoic acid (52.6 mg, 0.54 mmol), EDC·HCl (102.9 mg, 0.54 mmol), DMAP (5.9 mg, 0.05 mmol) and DIPEA (211.0 µL, 1.22 mmol) were added at 0 °C. The reaction mixture was stirred overnight and diluted with saturated NH_4Cl solution followed by the extraction with DCM (3x). The combined organic phases were washed with saturated $NaHCO_3$ solution, $KHSO_4$ (1 N) solution and saturated aq. NaCl solution, dried over Na_2SO_4, filtered and concentrated *in vacuo*. The crude product was purified by flash column chromatography (SiO_2, DCM/MeOH = 95:5) to obtain the desired compound **39a** (144.5 mg, 0.36 mmol, 73%) as colorless oil.

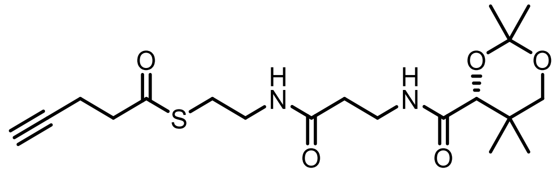

TLC: R_f(**39a**) = 0.33 (DCM/MeOH = 95:5). ^{1}H NMR (500 MHz, $CDCl_3$) δ: 0.98 (s, 3 H), 1.04 (s, 3 H), 1.43 (s, 3 H), 1.47 (s, 3 H), 2.02 (t, *J*=2.59 Hz, 1 H), 2.43 (t, *J*=6.18 Hz, 2 H), 2.55 (td, *J*=7.21, 2.67 Hz, 2 H), 2.81 (t, *J*=7.25 Hz, 2 H), 3.06 (t, *J*=6.41 Hz, 2 H), 3.29 (d, *J*=11.75 Hz, 1 H), 3.38 - 3.62 (m, 4 H), 3.69 (d, *J*=11.60 Hz, 1 H), 4.08 (s, 1 H), 6.14 (br s, 1 H), 6.98 - 7.07 (m, 1 H). ^{13}C NMR (126 MHz, MHz, $CDCl_3$) δ: 14.6, 18.7, 18.9, 22.1, 28.6, 29.5, 33.0, 34.7, 36.0, 39.4, 42.4, 69.6, 71.4, 77.1, 81.8, 99.1, 170.1, 171.2, 197.6. HRMS(ESI+) m/z calculated for $C_{19}H_{31}N_2O_5S$ $[M+H]^+$ 399.1948; found, 399.1962.

(*R*)-*S*-(2-(3-(2,4-Dihydroxy-3,3-dimethylbutanamido)propanamido)ethyl) pent-4-yne-thioate (39)

To a solution of (*R*)-*S*-(2-(3-(2,2,5,5-tetramethyl-1,3-dioxane-4-carboxamido)propanamido)ethyl) pent-4-ynethioate **39a** (144.5 mg, 0.36 mmol) in THF (3.6 mL), HCl (1 M) solution (0.36 mmol) was added at 0 °C. After full conversion of the starting material (LC-MS), the reaction mixture was diluted with DCM. The organic phase was washed with brine, dried over Na_2SO_4, filtered and the solvent was removed under reduced pressure to give compound **39** (100.7 mg, 0.28 mmol, 78%) as a colorless oil without further purification.

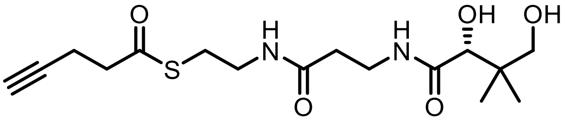

TLC: R_f(**39**) = 0.10 (DCM/MeOH = 95:5). ^{1}H NMR (500 MHz, $CDCl_3$) δ: 0.92 (s, 3 H), 1.00 (s, 3 H), 1.08 (s, 1 H), 1.23 (s, 1 H), 2.03 (t, *J*=2.59 Hz, 1 H), 2.43 (t, *J*=6.03 Hz, 2 H), 2.53 (td, *J*=7.21, 2.67 Hz, 2 H), 2.78 - 2.83 (m, 2 H), 2.98 - 3.11 (m, 2 H), 3.35 - 3.47 (m, 2 H), 3.48 (d, *J*=2.29 Hz, 2 H), 3.55 (q, *J*=6.10 Hz, 2 H), 4.00 (s, 1 H), 6.60 (br t, *J*=5.49 Hz, 1 H), 7.43 - 7.50 (m, 1 H). ^{13}C NMR (126 MHz, $CDCl_3$) δ: 14.6, 20.4, 21.4, 28.4, 35.2, 35.6, 39.3, 39.4, 42.3, 69.6, 70.8, 77.5, 81.8, 171.8, 173.8, 197.9. HRMS(ESI+) m/z calculated for $C_{16}H_{27}N_2O_5S$ $[M+H]^+$ 359.1635; found, 359.1633.

Ethyl (*E*)-3-methylhex-2-enoate (40a)

According to **GP A**, ethyl-2-butynoate (560.7 mg, 5.0 mmol) was reacted with propyl magnesium chloride (3.13 mL, 6.25 mmol, 2 M in Et_2O) and $CuBr{\cdot}SMe_2$ (1.28 g, 6.25 mmol) to obtain ethyl (*E*)-3-methtylhex-2-enoate **40a** (712.0 mg, 4.56 mmol, 91%) as a colorless oil without further purification.

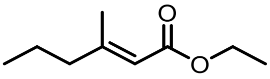

TLC: R_f(**40a**) = 0.50 (hexanes/EtOAc = 90:10). ^{1}H NMR (500 MHz, $CDCl_3$) δ: 0.92 (t, *J*=7.40 Hz, 3 H), 1.28 (t, *J*=7.10 Hz, 3 H), 1.51 (sxt, *J*=7.45 Hz, 2 H), 2.12 (dd, *J*=7.86, 7.25 Hz, 2 H), 2.15 (d, *J*=1.07 Hz, 3 H), 4.15 (q, *J*=7.07 Hz, 2 H), 5.66 (d, *J*=1.22 Hz, 1 H). ^{13}C NMR (126 MHz, $CDCl_3$) δ: 13.9, 14.3, 18.7, 22.3, 29.5, 40.6, 59.4, 115.4, 160.3, 166.9. HRMS(ESI+) m/z calculated for $C_9H_{17}O_2$ $[M+H]^+$ 157.1223; found, 157.1224.

Ethyl (2*E*,4*E*)-2,5-dimethylocta-2,4-dienoate (40b)

According to **GP B**, ester **19a** (345.3 mg, 2.21 mmol) was reacted with DibalH (6.63 mmol, 1 M in hexanes). The crude product was then converted with activated manganese dioxide (5.06

g, 22.10 mmol) and ethyl 2-(triphenylphosphoranylidene)propionate (1.04 g, 2.87 mmol). After purification *via* flash chromatography (SiO_2, hexanes/EtOAc = 95:5), the diene **40b** (214.8 mg, 1.09 mmol, 50%, *E*,*E* > 99%) was obtained as colorless oil.

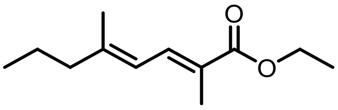

TLC: R_f(**40b**) = 0.43 (hexanes/EtOAc = 90:10). ^{1}H NMR (500 MHz, $CDCl_3$) δ: 0.91 (t, *J*=7.40 Hz, 3 H), 1.31 (t, *J*=7.17 Hz, 3 H), 1.51 (sxt, *J*=7.45 Hz, 2 H), 1.88 (s, 3 H), 1.93 (d, *J*=0.76 Hz, 3 H), 2.15 (t, *J*=7.55 Hz, 2 H), 4.22 (q, *J*=7.07 Hz, 2 H), 6.13 (d, *J*=11.72 Hz, 1 H), 7.48 (dd, *J*=11.90, 1.37 Hz, 1 H). ^{13}C NMR (126 MHz, $CDCl_3$) δ: 12.4, 13.7, 14.4, 17.1, 21.0, 42.8, 60.4, 120.7, 124.6, 134.4, 148.1, 169.0. HRMS(ESI+) m/z calculated for $C_{12}H_{21}O_2$ $[M+H]^+$ 197.1536; found, 197.1543.

(2*E*,4*E*)-2,5-Dimethylocta-2,4-dienoic acid (40c)

According to **GP C**, ethyl (2*E*,4*E*)-2,5-dimethylocta-2,4-dienoate **40b** (212.6 mg, 1.08 mmol) was reacted with NaOH (199.2 mg, 4.98 mmol) in MeOH/H_2O (3:1) (5.40 mL) to yield in acid **19c** (177.3 mg, 1.05 mmol, 97%) as a colorless oil.

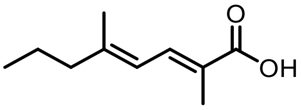

TLC: R_f(**40c**) = 0.18 (hexanes/EtOAc = 90:10). ^{1}H NMR (500 MHz, $CDCl_3$) δ: 0.92 (t, *J*=7.32 Hz, 3 H), 1.52 (dq, *J*=14.86, 7.41 Hz, 2 H), 1.90 (s, 3 H), 1.94 (s, 3 H), 2.13 - 2.19 (m, 2 H), 6.16 (br d, *J*=11.90 Hz, 1 H), 7.61 (br d, *J*=11.90 Hz, 1 H). ^{13}C NMR (126 MHz, $CDCl_3$) δ: 12.0, 13.8, 17.2, 21.0, 42.8, 120.8, 123.4, 136.8, 149.9, 174.1. HRMS(ESI+) m/z calculated for $C_{10}H_{17}O_2$ $[M+H]^+$ 169.1223; found, 169.1226.

***S*-(2-Acetamidoethyl) (2*E*,4*E*)-2,5-dimethylocta-2,4-dienethioate (40)**

According to **GP D**, (2*E*,4*E*)-2,5-dimethylocta-2,4-dienoic acid **40c** (169.8 mg, 1.01 mmol) was reacted with EDC·HCl (193.4 mg, 1.01 mmol), DMAP (12.2 mg, 0.10 mmol), DIPEA (175.6 µL, 1.01 mmol) and *N*-acetylcysteamine (120.3 mg, 1.01 mmol) in DCM (2.00 mL). Purification by flash column chromatography (SiO_2, $CHCl_3$/MeOH = 90:10) gave the desired SNAC-ester **40** (260.8 mg, 0.97 mmol, 96%) as a colorless oil.

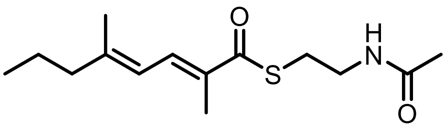

TLC: R_f(**40**) = 0.58 ($CHCl_3$/MeOH = 90:10). ^1H NMR (500 MHz, $CDCl_3$) δ: 0.92 (t, *J*=7.40 Hz, 3 H), 1.52 (sxt, *J*=7.45 Hz, 2 H), 1.91 (s, 3 H), 1.97 (s, 3 H), 1.98 (s, 3 H), 2.17 (t, *J*=7.55 Hz, 2 H), 3.10 (t, *J*=6.33 Hz, 2 H), 3.47 (q, *J*=5.95 Hz, 2 H), 5.96 (br s, 1 H), 6.16 (d, *J*=11.49 Hz, 1 H), 7.50 (d, *J*=11.54 Hz, 1 H). ^{13}C NMR (126 MHz, $CDCl_3$) δ: 12.5, 13.7, 17.4, 21.0, 23.2, 28.4, 40.0, 42.9, 120.5, 132.0, 134.1, 150.9, 170.3, 193.9. HRMS(ESI+) m/z calculated for $C_{14}H_{24}NO_2S$ $[M+H]^+$ 270.1522; found, 270.1525.

Ethyl (*E*)-3-methylhept-2-enoate (41a)

According to **GP A**, ethyl-2-butynoate (560.7 mg, 5.0 mmol) was reacted with *n*-butyllithium (3.90 mL, 6.25 mmol, 1.6 M in hexanes) and CuBr·SMe_2 (1.28 g, 6.25 mmol) to obtain ethyl (*E*)-3-methtylhept-2-enoate **41a** (748.1 mg, 4.39 mmol, 88%) as a colorless oil without further purification.

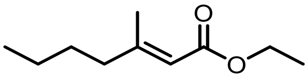

TLC: R_f(**41a**) = 0.65 (hexanes/EtOAc = 70:30). ^1H NMR (500 MHz, $CDCl_3$) δ: 0.91 (t, *J*=7.32 Hz, 4 H), 1.20 - 1.41 (m, 6 H), 1.42 - 1.49 (m, 2 H), 2.12 - 2.17 (m, 5 H), 4.15 (q, *J*=7.12 Hz, 2 H), 5.65 - 5.67 (m, 1 H). ^{13}C NMR (126 MHz, $CDCl_3$) δ: 13.89, 14.3, 18.7, 22.3, 29.5, 40.6, 59.4, 115.4, 160.4, 166.9. HRMS(ESI+) m/z calculated for $C_{10}H_{19}O_2$ $[M+H]^+$ 171.1380; found, 171.1383.

Ethyl (2*E*,4*E*)-2,5-dimethylnona-2,4-dienoate (41b)

According to **GP B**, ester **20a** (313.3 mg, 1.84 mmol) was reacted with DibalH (5.52 mmol, 1 M in hexanes). The crude product was then converted with activated manganese dioxide (4.20 g, 18.36 mmol) and ethyl 2-(triphenylphosphoranylidene)propionate (862.6 mg, 2.38 mmol). After purification *via* flash chromatography (SiO_2, hexanes/EtOAc = 95:5), the diene **41b** (120.0 mg, 0.57 mmol, 31%, *E*,*E* > 99%) was obtained as colorless oil.

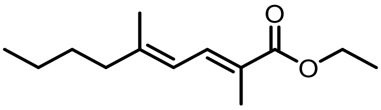

TLC: R_f(**41b**) = 0.36 (hexanes/EtOAc = 95 : 5). ^1H NMR (500 MHz, $CDCl_3$) δ: 0.92 (t, *J*=7.32 Hz, 3 H), 1.31 (t, *J*=7.17 Hz, 3 H), 1.42 - 1.49 (m, 2 H), 1.88 (s, 3 H), 1.94 (s, 3 H), 2.17 (t, *J*=7.63 Hz, 2 H), 4.22 (q, *J*=7.17 Hz, 2 H), 6.13 (d, *J*=11.81 Hz, 1 H), 7.48 (dd, *J*=11.90, 1.37 Hz, 1 H). ^{13}C NMR (126 MHz, $CDCl_3$) δ: 12.4, 13.9, 14.4, 17.2, 22.4, 30.0, 40.4, 60.4, 120.5, 124.5, 134.4, 148.4, 169.0. HRMS(ESI+) m/z calculated for $C_{13}H_{23}O_2$ $[M+H]^+$ 211.1693; found, 211.1693.

(2*E*,4*E*)-2,5-Dimethylnona-2,4-dienoic acid (41c)

According to **GP C**, ethyl (2*E*,4*E*)-2,5-dimethylnona-2,4-dienoate **41b** (116.4 mg, 0.55 mmol) was reacted with NaOH (101.8 mg, 2.45 mmol) in MeOH/H_2O (3:1) (2.75 mL) to yield in acid **41c** (98.0 mg, 0.54 mmol, 98%) as a colorless oil.

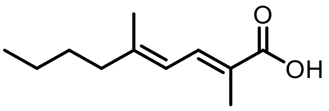

TLC: R_f(**41c**) = 0.17 (hexanes/EtOAc = 90 : 10). ^{1}H NMR (500 MHz, $CDCl_3$) δ: 0.93 (t, *J*=7.25 Hz, 3 H) 1.33 (dq, *J*=14.97, 7.42 Hz, 2 H) 1.43 - 1.51 (m, 2 H) 1.90 (s, 3 H) 1.95 (s, 3 H) 2.19 (t, *J*=7.63 Hz, 2 H) 6.15 (d, *J*=11.90 Hz, 1 H) 7.61 (d, *J*=11.90 Hz, 1 H) 11.23 (bs, 1 H). ^{13}C NMR (126 MHz, $CDCl_3$) δ: 12.0, 13.9, 17.3, 22.4, 30, 40.5, 120.6, 123.3, 136.8, 150.2, 173.6. HRMS(ESI+) m/z calculated for $C_{11}H_{19}O_2$ $[M+H]^+$ 183.1380; found, 183.1387.

***S*-(2-Acetamidoethyl) (2*E*,4*E*)-2,5-dimethylnona-2,4-dienethioate (41)**

According to **GP D**, (2*E*,4*E*)-2,5-dimethylnona-2,4-dienoic acid **20c** (98.0 mg, 0.54 mmol) was reacted with EDC·HCl (103.5 mg, 0.54 mmol), DMAP (6.6 mg, 0.05 mmol), DIPEA (93.0 µL, 0.54 mmol) and *N*-acetylcysteamine (64.4 mg, 0.54 mmol) in DCM (1.08 mL). Purification by flash column chromatography (SiO_2, $CHCl_3$/MeOH = 90:10) gave the desired SNAC-ester **41** (260.8 mg, 0.97 mmol, 96%) as a colorless oil.

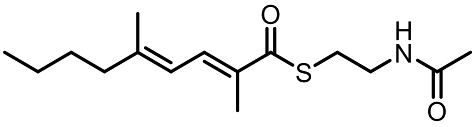

TLC: R_f(**41**) = 0.55 ($CHCl_3$/MeOH = 90:10). ^{1}H NMR (500 MHz, $CDCl_3$) δ: 0.93 (t, *J*=7.32 Hz, 3 H) 1.30 – 1.37 (m, 2 H) 1.43 - 1.51 (m, 2 H) 1.92 (s, 3 H) 1.97 (s, 3 H) 1.99 (s, 3 H) 2.19 (t, *J*=7.63 Hz, 2 H) 3.10 (t, *J*=6.33 Hz, 2 H) 3.48 (q, *J*=5.85 Hz, 2 H) 5.92 (br s, 1 H) 6.16 (dd, *J*=11.60, 1.22 Hz, 1 H) 7.50 (dd, *J*=11.60, 1.22 Hz, 1 H) ^{13}C NMR (126 MHz, $CDCl_3$) δ: 12.5, 13.9,) 17.5, 22.4, 23.3, 28.4, 30.0, 40.0, 40.6, 120.3, 131.9, 134.2, 151.2, 170.2, 193.9. HRMS(ESI+) m/z calculated for $C_{11}H_{19}O_2$ $[M+H]^+$ 284.1679; found, 284.1694.

Ethyl (*E*)-6,6,6-trifluoro-3-methylhex-2-enoate (42a)

To a –78 °C cold solution of 1,1,1-trifluoro-3-iodopropane (1.00 g, 4.47 mmol) in Et_2O/pentane (32.0 mL, 6:4), *tert*-butyl lithium (5.58 mL, 8.94 mmol, 1.6M in pentane) was added dropwise.[10] After stirring for 15 min, the reaction was warmed to 0 °C before THF (0.70 mL) was added. The reaction was further stirred for 15 min, cooled down to –45 °C, then added dropwise to a suspension of $CuBr·SMe_2$ (0.92 g, 4.47 mmol) in THF (35.0 mL) at –45 °C. After stirring for 30 min, the solution was cooled to –78°C and ethyl-2-butynoate (0.42 mL, 3.57 mmol) in THF (3.57 mL) was added dropwise. After stirring for 2 h at this temperature, the reaction was quenched with saturated NH_4Cl solution. After stirring for 10 min at room temperature the reaction mixture was diluted with water and was extracted with Et_2O (3x). The combined organic phases were washed with saturated aq. NaCl solution, dried over Na_2SO_4, filtered and concentrated *in vacuo*. Flash chromatography (SiO_2, hexanes/EtOAc = 90:10) afforded the desired product **42a** (0.46 g, 2.19 mmol, 49%) as colorless oil.

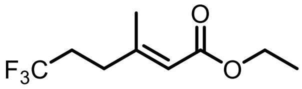

TLC: R_f(**42a**) = 0.45 (hexanes/EtOAc = 90:10). ^1H NMR (500 MHz, $CDCl_3$) δ: 1.29 (t, *J*=7.17 Hz, 3 H) 2.19 (d, *J*=1.22 Hz, 3 H) 2.22 - 2.32 (m, 2 H) 2.37 - 2.42 (m, 2 H) 4.16 (q, *J*=7.07 Hz, 2 H) 5.69 - 5.71 (m, 1 H). ^{13}C NMR (126 MHz, $CDCl_3$) δ: 14.3, 18.5, 31.9, (q, *J*=28.50 Hz) 32.8 (q, *J*=3.06 Hz) 59.8, 117.0, 126.6 (q, *J*=276.68 Hz) 155.3, 166.3. ^{19}F NMR (470 MHz, $CDCl_3$) δ: -66.52 (t, *J*=10.41 Hz). HRMS(ESI+) m/z calculated for $C_9H_{14}F_3O_2$ $[M+H]^+$ 211.0940; found, 211.0946.

Ethyl (2*E*,4*E*)-8,8,8-trifluoro-2,5-dimethylocta-2,4-dienoate (42b)

According to **GP B** ethyl (*E*)-6,6,6-trifluoro-3-methylhex-2-enoate **42a** (0.37 g, 1.74 mmol) was reacted with DibalH (5.23 mL, 5.23 mmol, 1M solution in DCM) in DCM (17 mL) to give an oil which was used directly in the next step. The crude product was dissolved in DCM (87 mL) and was reacted with activated manganese dioxide (3.98 g, 17.4 mmol) and ethyl 2-(triphenylphosphoranylidene)propionate (0.82 g, 2.26 mmol) for 24 h. After filtration through celite® and concentration *in vacuo*, purification by flash column chromatography (SiO_2, hexanes/EtOAc = 95:5) afforded compound **42b** (0.21 g, 0.85 mmol, 49%, *E*,*E* > 99%) as a colorless liquid.

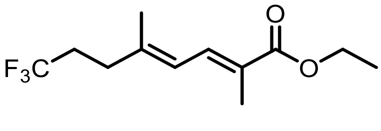

TLC: R_f(**42b**) = 0.35 (hexanes/EtOAc = 90:10). ^{1}H NMR (500 MHz, $CDCl_3$) δ: 1.29 - 1.35 (m, 3 H) 1.91 (s, 3 H) 1.94 (s, 3 H) 2.19 - 2.38 (m, 2 H) 2.39 - 2.45 (m, 2 H) 4.18 - 4.28 (m, 2 H) 6.17 (br d, *J*=11.75 Hz, 1 H) 7.43 (br d, *J*=11.75 Hz, 1 H). ^{13}C NMR (126 MHz, $CDCl_3$) δ: 12.5, 14.3, 17.0, 32.3 (q, *J*=28.50 Hz) 32.6 (q, *J*=2.76 Hz) 60.6, 121.8, 126.8 (q, *J*=276.37 Hz) 126.3, 133.4, 143.3, 168.7. ^{19}F NMR (470 MHz, $CDCl_3$) δ: -66.42 (t, *J*=10.40 Hz). HRMS(ESI+) m/z calculated for $C_{12}H_{18}F_3O_2$ $[M+H]^+$ 251.1253; found, 251.1258.

(2*E*,4*E*)-8,8,8-Trifluoro-2,5-dimethylocta-2,4-dienoic acid (42c)

According to **GP C**, ethyl (2*E*,4*E*)-8,8,8-trifluoro-2,5-dimethylocta-2,4-dienoate **42b** (0.21 g, 0.85 mmol) was reacted with NaOH (0.16 g, 3.90 mmol) in MeOH/H_2O (4.3 mL, 3:1) to yield in acid **42c** (0.17 g, 0.77 mmol, 90%) as a colorless oil.

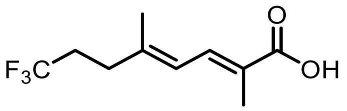

TLC: R_f(**42c**) = 0.16 (hexanes/EtOAc = 90:10). ^{1}H NMR (500 MHz, $CDCl_3$) δ: 1.93 (s, 3 H), 1.95 (s, 3 H), 2.23 - 2.35 (m, 2 H), 2.42 - 2.49 (m, 2 H), 6.20 (d, *J*=11.75 Hz, 1 H), 7.57 (d, *J*=11.75 Hz, 1 H). ^{13}C NMR (126 MHz, $CDCl_3$) δ: 12.1, 17.1, 32.3 (q, *J*=28.50 Hz), 32.7 (q, *J*=2.80 Hz), 121.8, 125.2, 126.7 (q, *J*=276.70 Hz), 135.76, 145.0, 174.0. ^{19}F NMR (470 MHz, $CDCl_3$) δ: -66.40 (t, *J*=10.40 Hz). HRMS(ESI+) m/z calculated for $C_{10}H_{14}F_3O_2$ $[M+H]^+$ 223.0940; found, 223.0934.

***S*-(2-Acetamidoethyl) (2*E*,4*E*)-8,8,8-trifluoro-2,5-dimethylocta-2,4-dienethioate (42)**

According to **GP D**, (2*E*,4*E*)-8,8,8-trifluoro-2,5-dimethylocta-2,4-dienoic acid **42c** (0.17 g, 0.77 mmol) was reacted with EDC·HCl (0.15 g, 0.77 mmol), DMAP (9.0 mg, 0.08 mmol), DIPEA (0.14 mL, 0.77 mmol) and *N*-acetylcysteamine (0.09 g, 0.77 mmol) in DCM (1.54 mL). Purification by flash column chromatography (SiO_2, $CHCl_3$/MeOH = 90:10) gave the desired SNAC-ester **42** (0.23 g, 0.71 mmol, 92%) as a colorless oil.

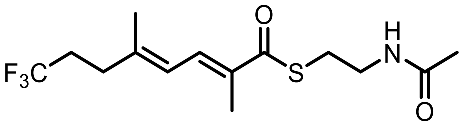

TLC: R_f(**42**) = 0.50 ($CHCl_3$/MeOH = 90:10). ^{1}H NMR (500 MHz, $CDCl_3$) δ: 1.90 - 2.10 (m, 9 H), 2.23 - 2.34 (m, 2 H), 2.40 - 2.49 (m, 2 H), 3.11 (t, *J*=6.41 Hz, 2 H), 3.48 (q, *J*=6.21 Hz, 2 H), 5.91 (br s, 1 H), 6.20 (dd, *J*=11.44, 0.92 Hz, 1 H), 7.44 (d, *J*=11.44 Hz, 1 H). ^{13}C NMR (126 MHz, $CDCl_3$) δ: 12.6, 17.2, 23.3, 28.5, 32.3 (q, *J*=28.50 Hz, 1 C) 32.7 (q, *J*=3.70 Hz, 1 C) 39.9, 121.6, 126.7 (q, *J*=276.70 Hz, 1 C) 132.8, 133.5, 145.6, 170.3, 193.8. ^{19}F NMR (470 MHz,

$CDCl_3$) δ: -66.39 (t, *J*=10.4). HRMS(ESI+) m/z calculated for $C_{14}H_{21}F_3NO_2S$ $[M+H]^+$ 324.1240; found, 324.1248.

(4-Bromobut-1-yn-1-yl)trimethylsilane (43aa)

Triphenylphosphine (3.15 g, 12.0 mmol) was added to a solution of 4-(trimethylsilyl)but-3-yn-1-ol (1.42 g, 10.0 mmol) in DCM (20 mL) at –20 °C before *N*-bromo succinimide (1.96 g, 11.0 mmol) was slowly added at this temperature and the reaction mixture was allowed to warm up to room temperature overnight.[11] After the addition of a saturated $NaHCO_3$ solution the reaction mixture was extracted with Et_2O (2x). The combined organic phases were dried over Na_2SO_4, filtered and the solvent was removed under reduced pressure. The crude product was purified by column chromatography (SiO_2, pentane/Et_2O = 99:1) to afford (4-bromobut-1-yn-1-yl)trimethylsilane **43aa** (1.75 g, 8.53 mmol, 85%) as a colorless oil.

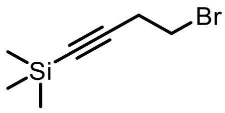

TLC: R_f(**43aa**) = 0.26 (pentane/Et_2O = 99:1). ^1H NMR (500 MHz, $CDCl_3$) δ: 0.17 (s, 9 H) 2.78 (t, *J*=7.55 Hz, 2 H) 3.44 (t, *J*=7.48 Hz, 2 H).^{13}C NMR (126 MHz, $CDCl_3$) δ: -0.1, 24.3, 29.2, 87.0, 103.2. NMR spectral data were consistent with those available in the literature.

Ethyl (*E*)-3-methyl-7-(trimethylsilyl)hept-2-en-6-ynoate (43a)

A dry and argon-flushed Schlenk tube was charged with dry LiCl (233.2 mg, 5.5 mmol) and magnesium turnings (145.9 mg, 6.0 mmol) in THF (4.0 mL). Bromide **43aa** (1.03 g, 5.0 mmol) in THF (2.0 mL) was added slowly at 0 °C. The reaction mixture was allowed to stir at room temperature for 2 h before it was cooled down to –45 °C, and added dropwise to a suspension of CuBr·SMe_2 (1.03 g, 5.0 mmol) in THF (8.0 mL) at –45 °C. After stirring for 30 min, the solution was cooled to –78°C and ethyl-2-butynoate (448.5 mg, 4.0 mmol) in THF (1.0 mL) was added dropwise. After stirring for 2 h at this temperature, the reaction was quenched with saturated NH_4Cl solution. After stirring for 10 min at room temperature the reaction mixture was diluted with water and was extracted with Et_2O (3x). The combined organic phases were washed with saturated aq. NaCl solution, dried over Na_2SO_4, filtered and concentrated *in vacuo*. Flash chromatography (SiO_2, pentane/Et_2O = 99:1) afforded the desired product **43a** (203.0 mg, 0.85 mmol, 21%) as colorless oil.

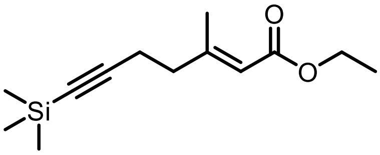

TLC: R_f(**43a**) = 0.48 (pentane/Et_2O = 90:10). ^{1}H NMR (500 MHz, $CDCl_3$) δ: 0.15 (d, *J*=1.22 Hz, 9 H), 1.28 (td, *J*=7.13, 1.14 Hz, 3 H), 2.17 (s, 3 H), 2.32 - 2.37 (m, 2 H), 2.38 - 2.43 (m, 2 H), 4.16 (qd, *J*=7.15, 1.14 Hz, 2 H), 5.69 - 5.73 (m, 1 H). ^{13}C NMR (126 MHz, $CDCl_3$) δ: 0.0, 14.3, 18.5, 18.6, 39.5, 59.6, 85.8, 105.6, 116.7, 157.2, 166.6. HRMS(ESI+) m/z calculated for $C_{13}H_{23}O_2Si$ $[M+H]^+$ 239.1462; found, 239.1466.

Ethyl (2*E*,4*E*)-2,5-dimethyl-9-(trimethylsilyl)nona-2,4-dien-8-ynoate (43b)

According to **GP B**, ester **43a** (208.0 mg, 0.87 mmol) was reacted with DibalH (2.61 mmol, 1 M in hexanes). The crude product was then converted with activated manganese dioxide (1.99 g, 8.70 mmol) and ethyl 2-(triphenylphosphoranylidene)propionate (409.5 mg, 1.13 mmol). After purification *via* flash chromatography (SiO_2, hexanes/EtOAc = 95:5), the diene **43b** (121.5 mg, 0,44 mmol, 50%, *E*,*E* > 99%) was obtained as colorless oil.

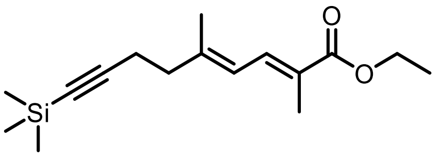

TLC: R_f(**43b**) = 0.41 (hexanes/EtOAc = 90 : 10). ^{1}H NMR (500 MHz, $CDCl_3$) δ: 0.14 (s, 9 H), 1.32 (t, *J*=7.10 Hz, 3 H), 1.90 (s, 3 H), 1.94 (s, 3 H), 2.36 - 2.43 (m, 4 H), 4.22 (q, *J*=7.07 Hz, 2 H), 6.19 (d, *J*=11.71 Hz, 1 H), 7.46 (dd, *J*=11.75, 1.37 Hz, 1 H). ^{13}C NMR (126 MHz, $CDCl_3$) δ: 0.1, 12.5, 14.3, 17.1, 18.9, 39.4, 60.4, 85.4, 106.2, 121.7, 125.5, 133.9, 145.3, 168.9. HRMS(ESI+) m/z calculated for $C_{16}H_{27}O_2Si$ $[M+H]^+$ 279.1775; found, 279.1780.

(2*E*,4*E*)-2,5-Dimethylnona-2,4-dien-8-ynoic acid (43c)

According to **GP C**, ethyl (2*E*,4*E*)-2,5-dimethyl-9-(trimethylsilyl)nona-2,4-dien-8-ynoate **43b** (0.12 g, 0.43 mmol) was reacted with NaOH (0.08 g, 1.98 mmol) in MeOH/H_2O (2.2 mL, 3:1) to yield in acid **43c** (0.06 g, 0.38 mmol, 88%) as a colorless oil.

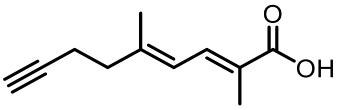

TLC: R_f(**43c**) = 0.10 (hexanes/EtOAc = 90 : 10). ^{1}H NMR (500 MHz, $CDCl_3$) δ: 1.93 (s, 3 H), 1.96 (s, 3 H), 2.00 (t, *J*=2.37 Hz, 1 H), 2.34 - 2.44 (m, 4 H), 6.23 (d, *J*=11.74 Hz, 1 H), 7.59 (dd, *J*=11.75, 1.22 Hz, 1 H). ^{13}C NMR (126 MHz, $CDCl_3$) δ: 12.1, 17.2, 17.3, 39.1, 69.1, 83.3, 121.7, 124.5, 136.2, 146.7, 173.5. HRMS(ESI+) m/z calculated for $C_{11}H_{14}O_2$ $[M+H]^+$ 179.1067; found, 179.1062.

S-(2-Acetamidoethyl) (2*E*,4*E*)-2,5-dimethylnona-2,4-dien-8-ynethioate (43)

According to **GP D**, (2*E*,4*E*)-2,5-dimethylnona-2,4-dien-8-ynoic acid **43c** (0.07 g, 0.39 mmol) was reacted with EDC·HCl (0.08 g, 0.39 mmol), DMAP (4.80 mg, 0.04 mmol), DIPEA (0.06 mL, 0.39 mmol) and *N*-acetylcysteamine (0.05 g, 0.39 mmol) in DCM (0.8 mL). Purification by flash column chromatography (SiO_2, $CHCl_3$/MeOH = 90:10) gave the desired SNAC-ester **43** (0.08 g, 0.28 mmol, 71%) as a colorless oil.

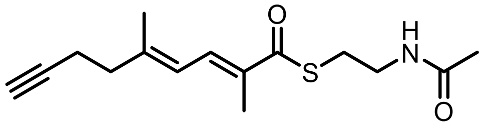

TLC: R_f(**43**) = 0.55 ($CHCl_3$/MeOH = 90 : 10). ^1H NMR (500 MHz, $CDCl_3$) δ:1.95 (s, 3 H), 1.98 (s, 3 H), 2.00 (s, 3 H), 2.36 - 2.45 (m, 4 H), 3.11 (t, *J*=6.33 Hz, 2 H), 3.48 (q, *J*=5.95 Hz, 2 H), 5.93 (br s, 1 H), 6.23 (d, *J*=11.40 Hz, 1 H), 7.48 (d, *J*=11.47 Hz, 1 H). ^{13}C NMR (126 MHz, $CDCl_3$) δ:12.5, 17.3, 17.3, 23.3, 28.5, 39.2, 40.0, 69.2, 83.2, 121.4, 133.0, 133.4, 147.5, 170.3, 193.9. HRMS(ESI+) m/z calculated for $C_{15}H_{22}NO_2S$ $[M+H]^+$ 280.1366; found, 280.1379.

Ethyl (*E*)-3-cyclohexylbut-2-enoate (44a)

According to **GP A**, ethyl-2-butynoate (560.7 mg, 5.0 mmol) was reacted with cyclohexyl magnesium chloride (3.13 mL, 6.25 mmol, 2 M in Et_2O) and $CuBr·SMe_2$ (1.28 g, 6.25 mmol). Flash chromatographic purification (SiO_2, hexanes/EtOAc = 96:4) yielded in ethyl (*E*)-3-cyclohexylbut-2-enoate **44a** (315.8 mg, 1.61 mmol, 32%) as a colorless oil.

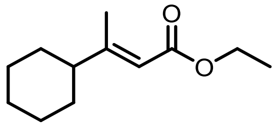

TLC: R_f(**44a**) = 0.30 (hexanes/EtOAc = 96:4). ^1H NMR (500 MHz, $CDCl_3$) δ: 1.12 - 1.36 (m, 9 H), 1.68 - 1.77 (m, 3 H), 1.77 - 1.83 (m, 2 H), 1.94 - 2.02 (m, 1 H), 2.15 (d, *J*=1.22 Hz, 3 H), 4.15 (q, *J*=7.07 Hz, 2 H), 5.64 - 5.67 (m, 1 H). ^{13}C NMR (126 MHz, CHLOROFORM-*d*) δ ppm 14.3, 17.4, 26.1, 26.4, 31.3, 48.7, 59.4, 113.9, 164.9, 167.3. HRMS(ESI+) m/z calculated for $C_{12}H_{21}O_2$ $[M+H]^+$ 197.1536; found, 197.1542.

Ethyl (2*E*,4*E*)-5-cyclohexyl-2-methylhexa-2,4-dienoate (44b)

According to **GP B**, ester **44a** (305.0 mg, 1.55 mmol) was reacted with DibalH (4.67 mmol, 1 M in hexanes). The crude product was then converted with activated manganese dioxide (1.59 g, 15.50 mmol) and ethyl 2-(triphenylphosphoranylidene)propionate (0.73 g, 2.02 mmol). After purification *via* flash chromatography (SiO_2, hexanes/EtOAc = 90:10), the diene **44b** (115.4 mg, 0.49 mmol, 32%, *E*,*E* > 90%) was obtained as colorless oil.

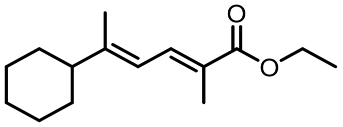

TLC: R_f(**44b**) = 0.34 (hexanes/EtOAc = 90:10). Mixture of isomers. Major isomer: ^{1}H NMR (500 MHz, $CDCl_3$) δ: 0.79 - 0.93 (m, 1 H), 1.16 - 1.34 (m, 10 H), 1.72 (br d, *J*=9.16 Hz, 3 H), 1.77 - 1.85 (m, 3 H), 1.87 (s, 3 H), 1.92 - 1.96 (m, 3 H), 1.96 - 2.05 (m, 1 H), 4.19 - 4.25 (m, 2 H), 6.14 (d, *J*=11.75 Hz, 1 H), 7.50 (d, *J*=11.76 Hz, 1 H). ^{13}C NMR (126 MHz, $CDCl_3$) δ: 12.4, 14.4, 15.6, 26.2, 26.5, 31.6, 48.5, 60.3, 118.8, 124.7, 134.6, 153.2, 169.0. Minor isomer (selected signals): ^{1}H NMR (500 MHz, $CDCl_3$) δ: 6.06 (d, *J*=12.21 Hz, 1 H), 7.53 - 7.58 (m, 1 H). ^{13}C NMR (126 MHz, $CDCl_3$) δ: 12.3, 14.4, 15.1, 26.3, 26.6, 31.1, 48.2, 60.4, 124.5, 133.3, 153.1, 169.2. HRMS(ESI+) m/z calculated for $C_{15}H_{25}O_2$ $[M+H]^+$ 237.1849; found, 237.1850.

(2*E*,4*E*)-5-Cyclohexyl-2-methylhexa-2,4-dienoic acid (44c)

According to **GP C**, ethyl (2*E*,4*E*)-5-cyclohexyl-2-methylhexa-2,4-dienoate **44c** (105.4 mg, 0.45 mmol) was reacted with NaOH (82.0 mg, 2.05 mmol) in MeOH/H_2O (2.20 mL, 3:1) to yield in acid **44c** (83.4 mg, 0.40 mmol, 90%, *E*,*E* > 90%) as a colorless oil.

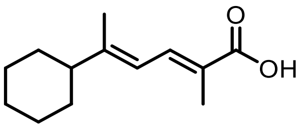

TLC: R_f(**44c**) = 0.24 (hexanes/EtOAc = 90 : 10). Mixture of isomers. Major isomer: ^{1}H NMR (500 MHz, $CDCl_3$) δ: 0.80 - 0.92 (m, 1 H), 1.17 - 1.39 (m, 8 H), 1.68 - 1.75 (m, 2 H), 1.77 - 1.86 (m, 3 H), 1.89 (s, 3 H), 1.95 (d, *J*=0.76 Hz, 3 H), 1.98 - 2.10 (m, 2 H), 6.18 (d, *J*=11.90 Hz, 1 H), 7.63 (d, *J*=11.86 Hz, 1 H), 11.14 (bs, 1 H). ^{13}C NMR (126 MHz, $CDCl_3$) δ: 12.1, 15.7, 26.3, 26.5, 31.6, 48.6, 118.8, 123.4, 137.0, 154.9, 173.4. Minor isomer (selected signals): ^{1}H NMR (500 MHz, $CDCl_3$) δ: 6.09 (dd, *J*=12.05, 1.07 Hz, 1 H), 7.68 (d, *J*=12.05 Hz, 1 H).HRMS(ESI+) m/z calculated for $C_{13}H_{21}O_2$ $[M+H]^+$ 209.1536; found, 209.1541.

***S*-(2-Acetamidoethyl) (2*E*,4*E*)-5-cyclohexyl-2-methylhexa-2,4-dienethioate (44)**

According to **GP D**, (2*E*,4*E*)-5-cyclohexyl-2-methylhexa-2,4-dienoic acid **44c** (82.0 mg, 0.39 mmol) was reacted with EDC·HCl (75.5 mg, 0.39 mmol), DMAP (4.8 mg, 0.04 mmol), DIPEA (54.0 µL, 0.39 mmol) and *N*-acetyl cysteamine (46.9 mg, 0.39 mmol) in DCM (0.80 mL). Purification by flash column chromatography (SiO_2, $CHCl_3$/MeOH = 90:10) gave the desired SNAC-ester **44** (120.7 mg, 0.39 mmol, quant., *E*,*E* > 90%) as a colorless oil.

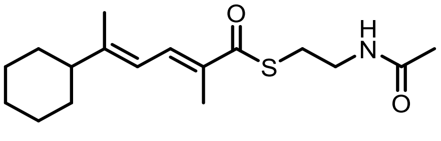

TLC: R_f(**44**) = 0.48 ($CHCl_3$/MeOH = 90:10). Mixture of isomers. Major isomer: ^{1}H NMR (500 MHz, $CDCl_3$) δ: 0.76 - 0.92 (m, 1 H) 1.13 - 1.44 (m, 8 H) 1.68 - 1.78 (m, 4 H) 1.78 - 1.89 (m, 4 H) 1.91 (d, *J*=0.61 Hz, 3 H) 1.97 (s, 3 H) 1.98 - 2.00 (m, 4 H) 3.08 - 3.12 (m, 2 H) 3.43 - 3.51 (m, 2 H) 5.95 (bs, 1 H) 6.15 - 6.19 (m, 1 H) 7.53 (dd, *J*=11.52, 1.30 Hz, 1 H). ^{13}C NMR (126 MHz, $CDCl_3$) δ: 12.5, 15.9, 23.3, 26.1, 26.3, 26.5, 28.4, 31.6, 40.0, 48.7, 118.6, 132.1, 134.4, 155.9, 170.3, 193.8. Minor isomer (selected signals): ^{1}H NMR (500 MHz, $CDCl_3$) δ: 6.08 (dd, *J*=11.83, 0.84 Hz, 1 H), 7.57 - 7.62 (m, 1 H). ^{13}C NMR (126 MHz, $CDCl_3$) δ: 12.3, 15.7, 21.4, 26.3, 26.5, 31.2, 39.9, 48.6, 118.8, 131.8, 134.9. HRMS(ESI+) m/z calculated for $C_{17}H_{28}NO_2S$ $[M+H]^+$ 310.1835; found, 310.1848.

Ethyl (2*E*,4*E*)-2-methylocta-2,4-dienoate (45b)

To a solution of *trans*-hexenal (0.23 mL, 2.0 mmol) in 4.0 mL toluene was added ethyl 2-(triphenylphosphoranylidene)propionate **B** (0.87 g, 2.4 mmol). The mixture was stirred overnight at room temperature. The solvent was removed *in vacuo*. The crude product was purified by flash column chromatography (SiO_2, hexanes/EtOAc = 98:2). The purified product **45b** (0.14 g, 0.77 mmol, 39%, *E*,*E* > 99%) was obtained as a colorless liquid.

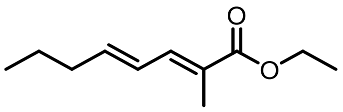

TLC: R_f(**45b**) = 0.63 (hexanes/EtOAc = 70:30). ^{1}H NMR (500 MHz, $CDCl_3$) δ: 0.94 (t, *J*=7.40 Hz, 3 H), 1.31 (t, *J*=7.17 Hz, 3 H), 1.47 (sxt, *J*=7.60 Hz, 2 H), 1.93 (d, *J*=1.00 Hz, 3 H), 2.18 (q, *J*=7.38 Hz, 2 H), 4.15 - 4.25 (m, 2 H), 6.08 (dt, *J*=14.80, 7.25 Hz, 1 H), 6.35 (ddt, *J*=15.11, 11.14, 1.40, 1.40 Hz, 1 H), 7.17 (d, *J*=11.29 Hz, 1 H). ^{13}C NMR (126 MHz, $CDCl_3$) δ: 12.5, 13.7, 14.3, 22.2, 35.3, 60.4, 125.1, 126.1, 138.6, 142.9, 168.7. HRMS(ESI+) m/z calculated for $C_{11}H_{19}O_2$ $[M+H]^+$ 183.1380; found, 183.1385.

(2*E*,4*E*)-2-Methylocta-2,4-dienoic acid (45c)

According to **GP C**, ethyl (2*E*,4*E*)-2-methylocta-2,4-dienoate **45b** (0.14 g, 0.77 mmol) was reacted with NaOH (0.14 g, 3.56 mmol) in MeOH/H_2O (4.0 mL, 3:1) to yield in acid **45c** (0.12 g, 0.77 mmol, quant.) as a colorless oil.

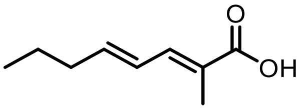

TLC: R_f(**45c**) = 0.22 (hexanes/EtOAc = 90:10). ^{1}H NMR (500 MHz, $CDCl_3$) δ: 0.94 (t, *J*=7.32 Hz, 3 H), 1.41 - 1.52 (m, 2 H), 1.94 (s, 3 H), 2.16 - 2.24 (m, 2 H), 6.15 (dt, *J*=14.84, 7.15 Hz, 1 H), 6.37 (t, *J*=13.05 Hz, 1 H), 7.29 (d, *J*=11.31 Hz, 1 H), 11.18 - 12.46 (m, 1 H),

11.79 (s, 1 H). ^{13}C NMR (126 MHz, $CDCl_3$) δ: 12.2, 13.7, 22.1, 35.4, 124.0, 126.1, 141.0, 144.5, 173.8. HRMS(ESI+) m/z calculated for $C_9H_{15}O_2$ $[M+H]^+$ 155.1067; found, 155.1067.

_S_-(2-Acetamidoethyl) (2_E_,4_E_)-2-methylocta-2,4-dienethioate (45)

According to **GP D**, (2*E*,4*E*)-2-methylocta-2,4-dienoic acid **45c** (0.11 g, 0.73 mmol) was reacted with EDC·HCl (0.14 g, 0.73 mmol), DMAP (8.90 mg, 0.07 mmol), DIPEA (0.12 mL, 0.73 mmol) and *N*-acetylcysteamine (0.09 g, 0.73 mmol) in DCM (1.4 mL). Purification by flash column chromatography (SiO_2, $CHCl_3$/MeOH = 95:5) gave the desired SNAC-ester **45** (0.14 g, 0.56 mmol, 77%) as a colorless oil.

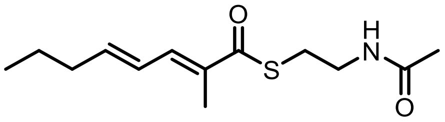

TLC: R_f(**45**) = 0.38 ($CHCl_3$/MeOH = 90:10). ^1H NMR (500 MHz, $CDCl_3$) δ: 0.94 (t, *J*=7.32 Hz, 3 H), 1.46 - 1.52 (m, 2 H), 1.97 (s, 3 H), 1.98 (s, 3 H), 2.20 (q, *J*=7.22 Hz, 2 H), 3.09 (t, *J*=6.33 Hz, 2 H), 3.44 - 3.50 (m, 2 H), 5.94 (br s, 1 H), 6.19 (dt, *J*=14.84, 7.15 Hz, 1 H), 6.37 (ddt, 1 H), 7.18 (d, *J*=10.83 Hz, 1 H). ^{13}C NMR (126 MHz, $CDCl_3$) δ: 12.6, 13.7, 22.0, 23.2, 28.4, 35.5, 39.9, 125.8, 132.5, 138.3, 145.2, 170.3, 193.7. HRMS(ESI+) m/z calculated for $C_{13}H_{22}NO_2S$ $[M+H]^+$ 256.1366; found, 256.1377.

Ethyl (2_E_,4_E_)-5-methylocta-2,4-dienoate (46b)

According to **GP B**, ester **41a** (345.3 mg, 2.21 mmol) was reacted with DibalH (6.63 mmol, 1 M in hexanes). The crude product was then converted with activated manganese dioxide (5.06 g, 22.10 mmol) and ethyl 2-(triphenylphosphoranylidene)acetate (1.00 g, 2.87 mmol). After purification *via* flash chromatography (SiO_2, hexanes/EtOAc = 95:5), diene **46b** (106.90 mg, 0.59 mmol, 27%, *E*,*E* > 99%) was obtained as colorless oil.

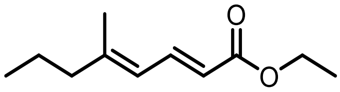

TLC: R_f(**46b**) = 0.41 (hexanes/EtOAc = 90:10). ^1H NMR (500 MHz, $CDCl_3$) δ: 0.90 (t, *J*=7.40 Hz, 3 H), 1.30 (t, *J*=7.17 Hz, 3 H), 1.49 (sxt, *J*=7.45 Hz, 2 H), 1.88 (d, *J*=1.00 Hz, 3 H), 2.11 (t, *J*=7.48 Hz, 2 H), 4.21 (q, *J*=7.07 Hz, 2 H), 5.78 (d, *J*=15.11 Hz, 1 H), 5.99 (d, *J*=11.60 Hz, 1 H), 7.59 (dd, *J*=15.11, 11.60 Hz, 1 H). ^{13}C NMR (126 MHz, $CDCl_3$) δ: 13.7, 14.3, 17.2, 20.8, 42.3, 60.1, 118.8, 123.2, 141.0, 150.0, 167.7. HRMS(ESI+) m/z calculated for $C_{11}H_{19}O_2$ $[M+H]^+$ 183.1380; found, 183.1386.

(2_E_,4_E_)-5-Methylocta-2,4-dienoic acid (46c)

According to **GP C**, ethyl (2*E*,4*E*)-5-methylocta-2,4-dienoate **46b** (0.10 g, 0.55 mmol) was reacted with NaOH (0.10 g, 2.56 mmol) in MeOH/H_2O (2.8 mL, 3:1) to yield in acid **46c** (0.08 g, 0.55 mmol, 99%) as a colorless oil.

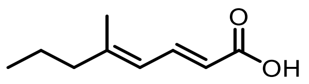

TLC: R_f(**46c**) = 0.10 (hexanes/EtOAc = 90:10). ^{1}H NMR (500 MHz, $CDCl_3$) δ: 0.91 (t, *J*=7.32 Hz, 3 H), 1.51 (sxt, *J*=7.45 Hz, 2 H), 1.90 (s, 3 H), 2.14 (t, *J*=7.48 Hz, 2 H), 5.79 (d, *J*=15.11 Hz, 1 H), 6.04 (br d, *J*=11.60 Hz, 1 H), 7.69 (dd, *J*=15.11, 11.60 Hz, 1 H). ^{13}C NMR (126 MHz, $CDCl_3$) δ: 13.7, 17.4, 20.8, 42.4, 117.7, 123.2, 143.3, 151.8, 172.4. HRMS(ESI+) m/z calculated for $C_9H_{15}O_2$ $[M+H]^+$ 155.1067; found, 155.1064.

S-(2-Acetamidoethyl) (2*E*,4*E*)-5-methylocta-2,4-dienethioate (46)

According to **GP D**, (2*E*,4*E*)-5-methylocta-2,4-dienoic acid **46c** (0.08 g, 0.53 mmol) was reacted with EDC·HCl (0.10 g, 0.53 mmol), DMAP (6.47 mg, 0.05 mmol), DIPEA (0.09 mL, 0.53 mmol) and *N*-acetylcysteamine (0.06 g, 0.53 mmol) in DCM (1.0 mL). Purification by flash column chromatography (SiO_2, $CHCl_3$/MeOH = 95:5) gave the desired SNAc-ester **46** (0.11 g, 0.42 mmol, 79%) as a colorless oil.

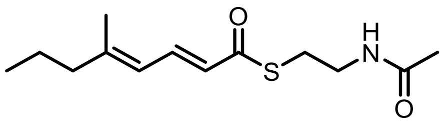

TLC: R_f(**46**) = 0.55 ($CHCl_3$/MeOH = 90:10). ^{1}H NMR (500 MHz, $CDCl_3$) δ: 0.91 (t, *J*=7.40 Hz, 3 H), 1.51 (sxt, *J*=7.45 Hz, 2 H), 1.91 (d, *J*=0.92 Hz, 3 H), 1.97 (s, 3 H), 2.14 (t, *J*=7.48 Hz, 2 H), 3.12 (t, *J*=6.26 Hz, 2 H), 3.48 (q, *J*=5.80 Hz, 2 H), 5.97 (br d, *J*=11.60 Hz, 2 H), 6.11 (d, *J*=14.95 Hz, 1 H), 7.55 (dd, *J*=14.80, 11.60 Hz, 1 H). ^{13}C NMR (126 MHz, $CDCl_3$) δ: ppm 13.7, 17.5, 20.8, 23.2, 28.3, 40.0, 42.5, 123.1, 125.4, 137.9, 153.3, 170.3, 190.6. HRMS(ESI+) m/z calculated for $C_{13}H_{22}NO_2S$ $[M+H]^+$ 256.1366; found, 256.1372.

Ethyl (2*E*,4*E*)-octa-2,4-dienoate (47b)

To a solution of *trans*-hexenal (0.23 mL, 2.0 mmol) in toluene (4.0 mL) was added ethyl 2-(triphenylphosphoranylidene)acetate (0.84 g, 2.4 mmol). The mixture was stirred overnight at room temperature. The solvent was removed *in vacuo*. The crude product was purified by flash column chromatography (SiO_2, hexanes/EtOAc = 98:2). The purified product **47b** (0.23 g, 1.37 mmol, 68%, *E*,*E* > 99%) was obtained as a colorless liquid.

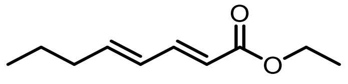

TLC: R_f(**47b**) = 0.40 (hexanes/EtOAc = 90:10). ^{1}H NMR (500 MHz, $CDCl_3$) δ: 0.92 (t, *J*=5.0 Hz, 3 H), 1.29(t, *J*=5.0 Hz, 3 H), 1.40 - 1.60 (m, 2 H), 2.10 - 2.21 (m, 2 H), 4.15 - 4.27 (m, 2 H), 5.78 (d, *J*=15.41 Hz, 1 H), 6.08 - 6.20 (m, 2 H), 7.22 - 7.31 (m, 1 H). ^{13}C NMR (126 MHz, $CDCl_3$) δ: 13.6, 14.3, 21.9, 35.0, 60.1, 119.2, 128.5, 144.5, 145.1, 167.3. HRMS(ESI+) m/z calculated for $C_{10}H_{17}O_2$ $[M+H]^+$ 169.1223; found, 169.1227.

(2*E*,4*E*)-Octa-2,4-dienoic acid (47c)

According to **GP C**, ethyl (2*E*,4*E*)-octa-2,4-dienoate **47b** (0.22 g, 1.30 mmol) was reacted with NaOH (0.24 g, 5.98 mmol) in MeOH/H_2O (6.3 mL, 3:1) to yield in acid **47c** (0.18 g, 1.30 mmol, quant.) amorphous white solid.

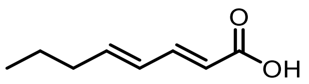

TLC: R_f(**47c**) = 0.22 (hexanes/EtOAc = 90:10). ^{1}H NMR (500 MHz, $CDCl_3$) δ: 0.94 (t, *J*=7.40 Hz, 3 H), 1.42 - 1.52 (m, 2 H), 2.12 - 2.22 (m, 2 H), 5.80 (d, *J*=15.41 Hz, 1 H), 6.18 - 6.25 (m, 2 H), 7.29 - 7.42 (m, 1 H), 10.86 - 12.54 (m, 1 H), 11.77 (br s, 1 H). ^{13}C NMR (126 MHz, $CDCl_3$) δ: 13.7, 21.8, 35.1, 118.2, 128.4, 146.0, 147.5, 172.5. HRMS(ESI+) m/z calculated for $C_8H_{13}O_2$ $[M+H]^+$ 141.0910; found, 141.0908.

***S*-(2-Acetamidoethyl) (2*E*,4*E*)-octa-2,4-dienethioate (47)**

According to **GP D**, (2*E*,4*E*)-octa-2,4-dienoic acid **47c** (0.18 g, 1.30 mmol) was reacted with EDC·HCl (0.25 g, 1.30 mmol), DMAP (15.88 mg, 0.13 mmol), DIPEA (0.21 mL, 1.30 mmol) and *N*-acetylcysteamine (0.15 g, 1.30 mmol) in DCM (3.0 mL). Purification by flash column chromatography (SiO_2, $CHCl_3$/MeOH = 95:5) gave the desired SNAC-ester **47** (0.24 g, 1.00 mmol, 77%) as a colorless oil.

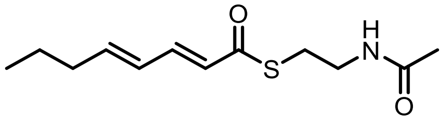

TLC: R_f(**47**) = 0.44 ($CHCl_3$/MeOH = 90:10). ^{1}H NMR (500 MHz, $CDCl_3$) δ: 0.93 (t, *J*=7.40 Hz, 3 H), 1.48 (sxt, *J*=7.60 Hz, 2 H), 1.97 (s, 3 H), 2.18 (q, *J*=6.97 Hz, 2 H), 3.12 (t, *J*=6.26 Hz, 2 H), 3.48 (q, *J*=5.80 Hz, 2 H), 5.91 (br s, 1 H), 6.11 (d, *J*=15.26 Hz, 1 H), 6.15 - 6.20 (m, 1 H), 6.22 - 6.28 (m, 1 H), 7.20 - 7.26 (m, 1 H). ^{13}C NMR (126 MHz, $CDCl_3$) δ: 13.7, 21.8, 23.2, 28.3, 35.2, 39.9, 125.8, 128.2, 142.0, 147.2, 170.3, 190.5. HRMS(ESI+) m/z calculated for $C_{12}H_{20}NO_2S$ $[M+H]^+$ 242.1209; found, 242.1216.

***S*-Phenyl (2*E*,4*E*)-2,5-dimethylocta-2,4-dienethioate (48)**

To a solution of (2*E*,4*E*)-2,5-dimethylocta-2,4-dienoic acid **40c** (100.0 mg, 0.59 mmol) in DCM (5.90 mL), thiophenol (65.5 mg, 0.59 mmol), EDC·HCl (101.5 mg, 0.65 mmol), DMAP (7.3 mg 0.06 mmol) and DIPEA (113.2 µL, 0.65 mmol) were added at 0 °C. The reaction mixture was stirred overnight and diluted with saturated NH_4Cl solution followed by the extraction with DCM (3x). The combined organic phases were washed with saturated $NaHCO_3$ solution, $KHSO_4$ (1N) solution and saturated aq. NaCl solution, dried over Na_2SO_4, filtered and concentrated *in vacuo*. Flash chromatography (SiO_2, DCM/MeOH = 95:5) afforded the desired product **48** (431.1 mg, 0.26 mmol, 81 %, *E*,*E* = 80%) as a yellowish oil.

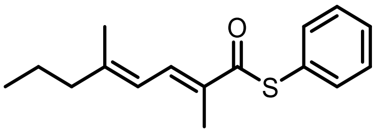

TLC: R_f(**48**) = 0.55 (DCM/MeOH = 90:10). Mixture of isomers. Major isomer: ^{1}H NMR (500 MHz, $CDCl_3$) δ: 0.93 (t, *J* = 7.3 Hz, 3H), 1.56 – 1.49 (m, 2H), 1.93 (s, 3H), 2.22 – 2.15 (m, 2H), 6.24 – 6.15 (m, 1H), 7.43 – 7.39 (m, 3H), 7.48 – 7.43 (m, 2H), 7.59 (d, *J* = 11.5 Hz, 1H). ^{13}C NMR (126 MHz, $CDCl_3$) δ: 12.7, 13.9, 17.6, 21.1, 43.1, 120.7, 128.54, 129.19, 132.2, 134.35, 135.3, 143.5, 150.8, 191.8. Minor isomer (selected signals): ^{1}H NMR (500 MHz, $CDCl_3$) δ: 0.96 (t, *J* = 7.4 Hz, 3H), 1.56 – 1.49 (m, 2H), 1.98 (s, 3H), 1.96 (s, 3H), 2.34 – 2.29 (m, 2H), 6.24 – 6.15 (m, 1H), 7.43 – 7.39 (m, 3H), 7.48 – 7.43 (m, 2H), 7.86 (d, *J* = 11.9 Hz, 1H). ^{13}C NMR (126 MHz, $CDCl_3$) δ: 12.7, 14.1, 21.8, 25.1, 35.0, 121.5, 129.0, 129.2, 131.9, 134.0, 135,1, 143.8, 151.09. HRMS(ESI+) m/z calculated for $C_{16}H_{19}OS$ $[M+H]^+$ 259.1151; found, 259.1150.

***S*-(2-(3-((*R*)-2,2,5,5-Tetramethyl-1,3-dioxane-4-carboxamido)propanamido)ethyl) (2*E*,4*E*)-2,5-dimethylocta-2,4-dienethioate (49a)**

To a solution of (*R*)-*N*-(3-((2-mercaptoethyl)amino)-3-oxopropyl)-2,2,5,5-tetramethyl-1,3-dioxane-4-carboxamide **A** (100.0 mg, 0.31 mmol) in DCM (3.1 mL), (2*E*,4*E*)-2,5-dimethylocta-2,4-dienoic acid **40c** (52.8 mg, 0.31 mmol), EDC·HCl (53.6 mg, 0.34 mmol), DMAP (3.8 mg, 0.03 mmol) and DIPEA (59.2 µL, 0.34 mmol) were added at 0 °C. The reaction mixture was stirred overnight and diluted with saturated NH_4Cl solution followed by the extraction with DCM (3x). The combined organic phases were washed with saturated $NaHCO_3$ solution, $KHSO_4$ (1N) solution and saturated aq. NaCl solution, dried over Na_2SO_4, filtered and concentrated *in vacuo*. The crude product was purified by flash column chromatography (SiO_2, DCM/MeOH = 95:5) to obtain the desired compound **49a** (96.9 mg, 0.21 mmol, 66%) as yellowish oil.

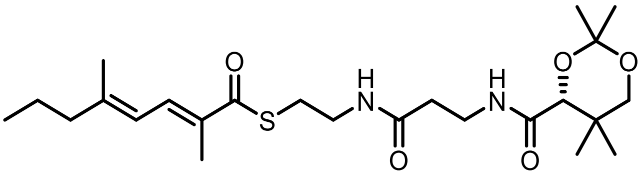

TLC: R_f(**49a**) = 0.39 (DCM/MeOH = 90:10). ^{1}H NMR (500 MHz, $CDCl_3$) δ: 0.91 (t, *J* = 7.3 Hz, 3H), 0.97 (s, 3H), 1.03 (s, 3H), 1.41 (s, 3H), 1.46 (s, 3H), 1.55 – 1.47 (m, 2H), 1.91 (s, 3H), 1.97 (s, 3H), 2.16 (dd, *J* = 8.8, 6.3 Hz, 2H), 2.43 (t, *J* = 5.7 Hz, 2H), 3.08 (t, *J* = 6.3 Hz, 2H), 3.27 (d, *J* = 11.7 Hz, 1H), 3.53 – 3.40 (m, 3H), 3.60 – 3.54 (m, 1H), 3.67 (d, *J* = 11.7 Hz, 1H), 4.07 (s, 1H), 6.15 (d, *J* = 11.5 Hz, 1H), 6.20 (s, 1H), 7.04 (s, 1H), 7.48 (d, *J* = 11.5 Hz, 1H). ^{13}C NMR (126 MHz, $CDCl_3$) δ: 12.6, 13.9, 17.6, 18.8, 19.1, 21.1, 22.3, 28.5, 29.6, 33.1, 34,9, 36.0, 40.1, 43.1, 71.6, 77.3, 99.2, 120.6, 132.1, 134.3, 151.1, 170.2, 171.4, 193.7. HRMS(ESI+) m/z calculated for $C_{24}H_{41}N_2O_5S$ $[M+H]^+$ 469.2731; found, 469.2704.

***S*-(2-(3-((*R*)-2,4-Dihydroxy-3,3-dimethylbutanamido)propanamido)ethyl) (2*E*,4*E*)-2,5-dimethylocta-2,4-dienethioate (49)**

To a solution of *S*-(2-(3-((*R*)-2,2,5,5-tetramethyl-1,3-dioxane-4-carboxamido)propanamido)-ethyl) (2*E*,4*E*)-2,5-dimethylocta-2,4-dienethioate **49a** (96.6 mg, 0.21 mmol) in THF (2.1 mL), HCl (1 M) solution (0.23 mmol) was added at 0 °C. After full conversion of the starting material (LC-MS), the reaction mixture was diluted with DCM. The organic phase was washed with saturated aq. NaCl solution, dried over Na_2SO_4, filtered and the solvent was removed under reduced pressure to give compound **49** (60.2 mg, 0.14 mmol, 67%) as a colorless oil without further purification.

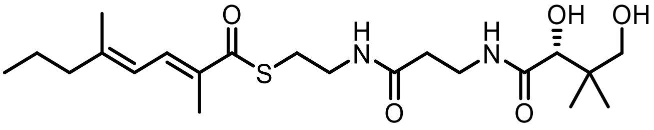

TLC: R_f(**49**) = 0.37 (DCM/MeOH = 90:10). ^{1}H NMR (500 MHz, $CDCl_3$) δ: 0.89 (d, *J* = 7.5 Hz, 3H), 0.90 (d, *J* = 7.2 Hz, 3H), 0.97 (s, 3H), 1.49 (m, 2H), 1.89 (s, 3H), 1.94 (s, 3H), 2.14 (t, *J* = 7.5 Hz, 2H), 2.44 – 2.35 (m, 2H), 3.15 – 3.00 (m, 2H), 3.49 – 3.33 (m, 5H), 3.53 (dd, *J* = 12.0, 6.1 Hz, 2H), 3.98 (s, 1H), 6.12 (d, *J* = 11.5 Hz, 1H), 6.63 (t, *J* = 5.5 Hz, 1H), 7.52 – 7.40 (m, 2H). ^{13}C NMR (126 MHz, $CDCl_3$) δ: 12.6, 13.8, 17.5, 20.5, 21.1, 21.6, 28.3, 35.3, 35.8, 39.4, 41.0, 43.0, 71.0, 77.6, 120.6, 132.0, 134.4, 151.3, 171.9, 173.9, 193.9. HRMS(ESI+) m/z calculated for $C_{21}H_{37}N_2O_5S$ $[M+H]^+$ 429.2418; found, 429.2421.

Methyl ((*R*,*E*)-5-(4-hydroxy-2-oxo-3-((2*E*,4*E*)-8,8,8-trifluoro-2,5-dimethylocta-2,4-dienoyl)-2H-pyran-6-yl)hex-1-en-1-yl)carbamate (8)

For the production of MYX (**8**), fermentations were performed using the previously described parallel cultivation system DASGIP (Eppendorf). The fermentations were conducted using M7/S4 media supplemented with 50 mg/L kanamycin and inoculated with 10% pre-culture. Throughout the fermentation process, the dissolved oxygen (DO) concentration in the medium was maintained at a constant level of 30% by adjusting the stirrer speed (150–600 rpm), gas flow rate (0.16–0.33 vvm), and gas composition (0–21% oxygen), realized by an internal control cascade. The pH: 7.4 was kept constant by the addition of H_2SO_4 (1 M) and KOH (1 M) as required. Foaming during fermentation was controlled by an internal control cascade that added antifoam SE-15 (Sigma-Aldrich) via a peristaltic pump operating at a flow rate of 40 mL/h. The batch fermentations were carried out in a cultivation volume of 1 L for 172 h at 25 °C. Following inoculation, the precursor **22** (10 mM DMSO stock) was fed in increasing amounts corresponding to 7.5%, 15%, 30%, and 47.5% of the total feeding amount at 24, 48, 72, and 96 h, respectively. The final concentration of the precursor in the medium was 1 mM. After 172 h of fermentation, the culture broth was extracted with ethyl acetate. After drying over $NaSO_4$ and filtration the organic solvent was removed under reduced pressure and the crude extract was further purified. Purification was achieved by size exclusion chromatography (Sephadex® LH20) using methanol as eluent. The MYX (**8**) containing fractions were then further purified using the previously described setup preparative scale HPLC setup. Separation of 500 µL sample was was performed with linear gradient of (A) H_2O + 0.1% FA to (B) ACN + 0.1% FA at a flow rate of 25 mL/min. The gradient was initiated by a 0.1 min isocratic step at 5% B, followed by an increase to 55% B in 6 min. The separation was achieved by a linear gradient from 55 to 60% B over 19 min. After a transition to 95% in 1 minute an isocratic flow of 95% B was applied before re-equilibration with initial conditions. To remove remaining impurities, a second HPLC purification step was conducted, using a Xbridge BEH Phenyl OBD Prep Column (Waters, 130 Å, 5 µm, 19 x 100 mm). The gradient was initiated by a 0.1 min isocratic step at 5% B, followed by an increase to 45% B in 6 min. The separation was achieved by a linear gradient from 45 to 50 % B over 19 min. After a transition to 95% in 1 min an isocratic flow of 95 % B was applied before re-equilibration with initial conditions. In total 3.5 mg of the desired product **8** were obtained as yellow oil. HRMS(ESI+) m/z calculated for $C_{23}H_{29}F_3NO_6$ $[M+H]^+$ 472.1941; found, 472.1942.

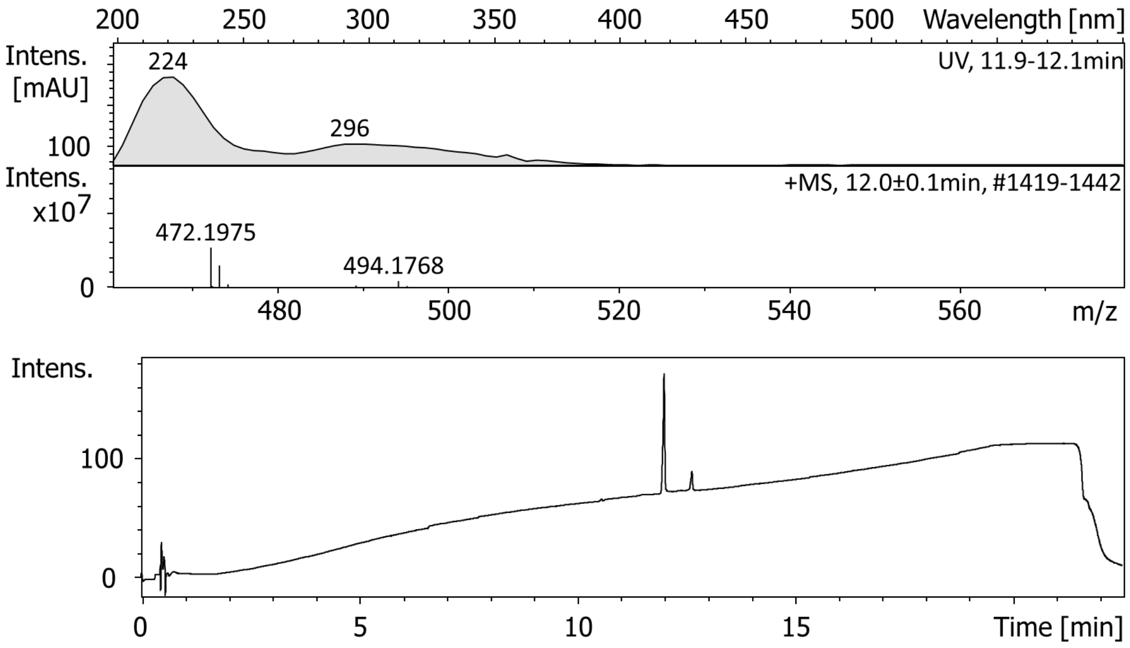

Figure S3: UV-Vis spectrum (top), HRMS spectrum (middle) and UHPLC chromatogram at 254 nm (bottom) of MYX (**8**).

Table S7: NMR chemical shifts of MYX (**8**) in MeOH-d_4 (500/126 MHz).

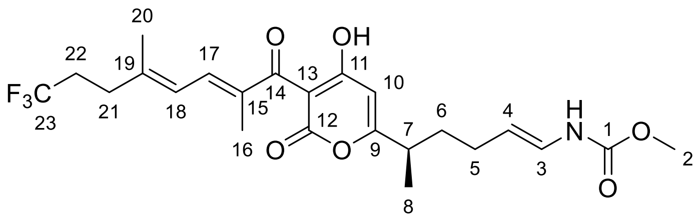

#	δ ¹³C [ppm]	δ ¹H [ppm]
**1**	156.8	–
**2**	52.6	3.66 (s, 3H)
**3**	125.8	6.39 (d, *J* = 14.1 Hz, 1H)
**4**	110.8	5.10 – 5.01 (m, 1H)
**5**	28.6	2.03 – 1.98 (m, 2H)
**6**	35.9	1.79 – 1.69 (m, 1H), 1.51 – 1.59 (m, 1H)
**7**	38.8	2.60 – 2.49 (q, *J* = 6.1 Hz, 1H)
**8**	18.6	1.22 (d, *J* = 6.8 Hz, 3H)
**9**	167.1	–
**10**	106.0	5.82 (s, 1H)
**11**	179.8	–
**12**	170.1	–
**13**	102.5	–
**14**	200.6	–
**15**	137.5	–
**16**	12.0	1.94 (s, 3H)
**17**	136.4	7.21 (d, *J* = 10.5 Hz, 1H)
**18**	123.6	6.32 (d, *J* = 10.6 Hz, 1H)
**19**	145.2	–
**20**	16.9	1.83 (s, 3H)
**21**	33.6 (d, *J* = 2.6 Hz)	1.36 – 1.31 (m, 2H)
**22**	33.0 (q, *J* = 28.3 Hz)	2.46 – 2.41 (m, 1H), 2.39 – 2.32 (m, 1H)
**23**	128.8 (q, *J* = 228.7 Hz)	–
**NH**	–	8.30 (s, 1H)

^{19}F NMR (470 MHz, MeOH-d_4) δ: -67.97 (t, *J*=10.41 Hz).

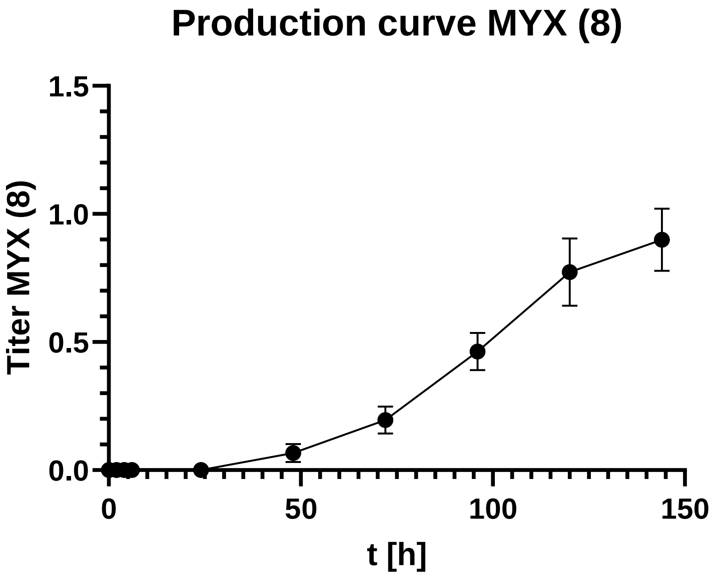

Figure S4: Production curve of MYX (**8**) in fermenter system. All fermentations were carried out with a cultivation volume of 1 L for 172 hours at 25 °C. After inoculation, feeding of the precursor **22** was performed with increasing amounts form 7.5, 15, 30 and 47.5 % of the overall feeding amount after 24, 48, 72 and 96 hours. Average production titer of MYX (**8**) is 0.90 ± 0.12 mg/L.

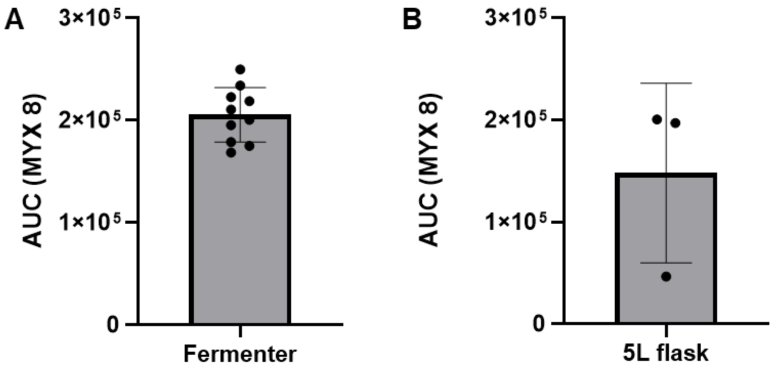

Figure S5: Comparison of MYX (8) in fermenter system and shaking flasks. (**A**) Fermentations in the fermenter system (n = 10) were conducted with a cultivation volume of 1 L for 172 hours at 25 °C. Precursor **22** was fed in increasing portions corresponding to 7.5, 15, 30, and 47.5% of the total feeding amount at 24, 48, 72, and 96 hours post-inoculation, respectively. (**B**) Fermentations in 5 L shaking flasks were carried out with a cultivation volume of 1.66 L for 172 hours at 30 °C, with precursor **22** added in a single portion at 48 hours post-inoculation, resulting in the same final concentration as used in the fermenter system experiments.

Bacterial assay

Bacteria were inoculated into cation-adjusted Mueller Hinton Broth to obtain a final inoculum of 10^5 colony-forming units (CFU)/mL. The tested compounds were prepared as DMSO stocks. Serial dilutions of derivatives in the growth medium were prepared in sterile 96-well plates and the bacterial suspensions were added. Growth inhibition was assessed after static incubation at 37 °C for 24 h. Obtain MIC visually.

Mycobacterium tuberculosis H37Rv (ATCC 27294) was used as the wild-type (WT) strain for all experiments. The rifampicin (RIF) mono-resistant strain was generated in-house by plating

1 x 10^9 CFU of H37Rv (WT) onto Middlebrook 7H10 (supplemented with 1 x OADC and 0.4 % glycerol) agar plates containing 3.5 uM RIF and selecting and culturing a single colony after 4 weeks incubation at 37 C. Following confirmation of RIF-resistance by broth micro-dilution MIC testing (see below), the resistance-determining mutation was identified by whole genome sequencing. The isolate contained a single nucleotide polymorphism (c1336g) within the *rpoB* gene, rv0667, leading to a H445D amino acid substitution in the RpoB protein. This mutation has previously been identified in rifampicin-resistant clinical *M. tuberculosis* isolates, and is listed in the WHO catalogue of *M. tuberculosis* drug resistance-causing mutations as being associated with RIF-resistance.[12]

Bacteria were routinely cultured in, and MIC assays performed in, Middlebrook 7H9 liquid media supplemented with 5 g/L bovine serum albumin, 0.85 g/L NaCl, 4 g/L glucose, and 0.5 ml/L tyloxapol ('7H9 ADNTy' complete medium). Compound efficacy was tested using a broth micro dilution protocol, as previously described[13] but with a few modifications. Briefly, clear, U-bottomed 96-well plates were prepared with each test compound present in an 11 point, 2-fold dilution series. Bacteria were diluted from exponentially growing cultures to a final OD_{600} of 0.001 in each well (~5000-10,000 cells per well), with each well containing a final volume of 100 µl. Final DMSO concentrations never exceeded 1 % (v/v). Plates were left to incubate at 37 °C, in ziplock bags within humidified sealed boxes for 7 days (WT H37Rv) or 13 days (RIF-resistant isolate), prior to visual examination of bacterial growth using an inverted mirror device. 30 ul of a 0.02 % (w/v) aqueous solution of resazurin was then added to each well and the plates left to incubate at 37 C for a further 24-48 h. Reduction of resazurin to resorufin, indicative of cell viability, was measured with a fluorescence plate reader (Synergy2, Agilent) at wavelengths 540(ex)/590(em) nm. Fluorescence values were converted to percentage growth relative to untreated (100 % growth) and 10 µM streptomycin treated (0 % growth) control wells. IC_{50} values were calculated based on non-linear regression analysis of resulting data sets, using GraphPad Prism software (sigmoidal 4 parameter logistic curve). All assays were performed in triplicate.

Metabolic stability in liver microsomes

For the evaluation of phase I metabolic stability, the compound (1 µM) was incubated with 0.5 mg/mL pooled mouse (C57BL/6), human or Wistar rat liver microsomes (Xenotech, Kansas City, USA), 2 mM NADPH, 10 mM $MgCl_2$ at 37 °C for 120 min on a microplate shaker (Eppendorf, Hamburg, Germany). The metabolic stability of testosterone, verapamil and ketoconazole were determined in parallel to confirm the enzymatic activity of mouse/rat liver microsomes. For human liver microsomes, testosterone, diclofenac and propranolol were used. The incubation was stopped after defined time points by precipitation of aliquots of the incubation mixture with 2 volumes of cold internal standard solution (15 nM diphenhydramine

in 10% methanol/acetonitrile). Samples were stored on ice until the end of the incubation and precipitated protein was removed by centrifugation (15 min, 4 °C, and 4,000 g). Concentration of the remaining test compound at the different time points was analyzed by HPLC-MS/MS (Vanquish Flex coupled to a TSQ Altis Plus, Thermo Fisher, Dreieich, Germany) and used to determine half-life ($t_{1/2}$).

Metabolic stability in mouse hepatocytes

For the evaluation of combined phase I and phase II metabolic stability, the compound (1 µM) was incubated with 0.25 x 10^6 cells/mL of pooled mouse hepatocytes (Xenotech, Kansas City, USA). Cells were thawed in Leibovitz's L-15 medium without phenol red (ThermoFisher Scientific, Waltham, USA). Briefly, cells were transferred into 50 mL of medium, followed by centrifugation at 55 g for 3 min. Supernatant was discarded and the cell count determined after gently resuspending the cell pellet in 1 mL of medium. Hepatocytes were diluted to 0.5 x 10^6 cells/mL and incubated at 37 °C, 700 rpm for 10 min, to achieve the desired final cell count after addition of an equal volume of test compounds in medium, leading to final test concentration of 1 µM at 1% DMSO. Samples were incubated at 37 °C, for 240 min, 700 rpm and the incubation was stopped after defined time points by precipitation of aliquots in 4 volumes cold internal standard solution (12.5 nM diphenhydramine in 10% methanol/acetonitrile). The metabolic stability of testosterone, verapamil, ketoconazole and 7-hydroxycoumarine were determined in parallel to confirm the enzymatic activity of mouse hepatocytes. Samples were stored on ice until the end of the incubation and precipitated protein was removed by centrifugation (15 min, 4 °C, 4.000 g). Concentration of the remaining test compound at the different time points was analyzed by HPLC-MS/MS (Vanquish Flex coupled to a TSQ Altis Plus, Thermo Fisher, Dreieich, Germany) and used to determine half-life ($t_{1/2}$).

The involvement of specific CYP enzymes in compound metabolism was assessed by incubation in human liver microsomes in presence and absence of the following CYP inhibitors at 10 µM: ketoconazole (CYP3A4), fluvoxamine (CYP1A2), sulfaphenazole (CYP2C9) and quinidine (CYP2D6).

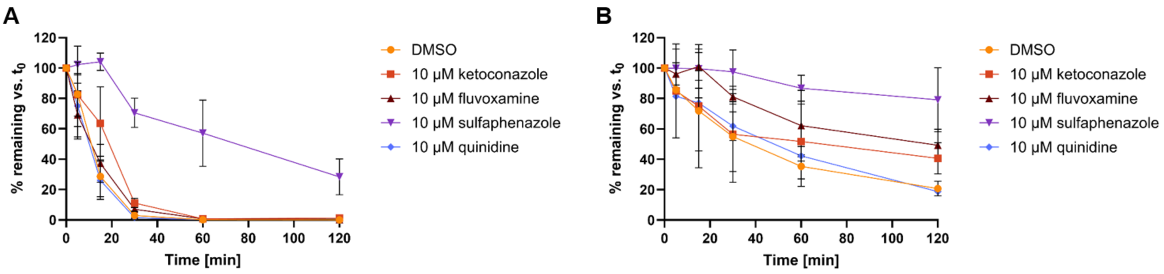

C

$t_{1/2}$ [min] (fold increase vs. DMSO)	MYX (1)	MYX (8)
DMSO*	5.5 ± 1.3	52.2 ± 5.9
Ketoconazole (3A4)	8.0 ± 0.3 (1.5 ± 0.4)	76 ± 39 (1.4 ± 0.6)
Fluvoxamine (1A2)	8.0 ± 1.9 (1.5 ± 0.1)	>80 (>1.5)
Sulfaphenazole (2C9)	82 ± 17 (11.4 ± 0.8)	>120 (>2.3)
Quinidine (2D6)	5.2 ± 1.0 (1.0 ± 0.1)	51.4 ± 0.4 (1.0 ± 0.1)

Figure S6: Metabolic stability in human liver microsomes with CYP isozyme involvement. (**A**) Human microsomal metabolism of MXY A (**1**) and (**B**) for MXY (**8**) in presence of 10 µM CYP inhibitors for CYP3A4 (ketoconazole), CYP1A2 (fluvoxamine), CYP2C9 (sulfaphenazole) or CYP2D6 (quinidine). The remaining test compound compared to t=0 min is given. (**C**) Corresponding half-lives ($t_{1/2}$s) and fold increase compared to $t_{1/2}$ of the compound in absence of inhibitors. Values represent means ± standard deviation (SD) of at least 3 independent measurements. *Mean half-life in presence of DMSO represents only assays where direct comparison to CYP inhibitors was performed; Table 2 of the main text includes additional replicates.

Plasma stability

To determine stability in plasma, the compound (1 µM) was incubated with pooled CD-1 mouse/human or Wistar rat plasma (Neo Biotech, Nanterre, France). Samples were taken by mixing aliquots with 4 volumes of cold internal standard solution (12.5 nM diphenhydramine in 10% methanol/acetonitrile). The plasma stability of procain, propantheline and diltiazem were determined in parallel to confirm the enzymatic activity. Samples were stored on ice until the end of the incubation and precipitated protein was removed by centrifugation (15 min, 4 °C, 4,000 g, two centrifugation steps). Concentration of the remaining test compound at the different time points was analyzed by HPLC-MS/MS (Vanquish Flex coupled to a TSQ Altis Plus, Thermo Fisher, Dreieich, Germany). The plasma stability of procain, propantheline and diltiazem were determined in parallel to confirm the enzymatic activity.

Plasma protein binding

Plasma protein binding was determined using the Rapid Equilibrium Dialysis (RED) system (Thermo Fisher Scientific, Waltham MA, USA). Compounds were diluted to 10 µM in 50% murine (CD-1), human or Wistar rat plasma (Neo Biotech, Nanterre, France) in PBS pH 7.4 and added to the respective chamber according to the manufacturer's protocol, followed by addition of PBS pH 7.4 to the opposite chamber. Samples were taken immediately after addition to the plate as well as after 2, 4 and 5 h by mixing 10 µL with 80 µL ice-cold ice-cold internal standard solution (12.5 nM diphenhydramine in 10% methanol/acetonitrile), followed

by addition of 10 µL plasma to samples taken from PBS and vice versa. Samples were stored on ice until the end of the incubation and precipitated protein was removed by centrifugation (15 min, 4 °C, 4,000 g, 2 centrifugation steps). Concentration of the remaining test compound at the different time points was analyzed by HPLC-MS/MS (Vanquish Flex coupled to a TSQ Altis Plus, Thermo Fisher, Dreieich, Germany). The amount of compound bound to protein was calculated using the equation PPB [%] = 100 – 100 x (amount in buffer chamber/amount in plasma chamber).

Cytotoxicity assay

To obtain information regarding the toxicity of test compounds, their impact on the viability of human cells was investigated. HepG2 cells ($2x10^4$ cells per well) were seeded in 96-well, flat-bottomed culture plates in 100 µL culture medium (DMEM containing 10% fetal calve serum, 1% penicillin-streptomycin). Twenty-four hours after seeding the cells, medium was removed and replaced by medium containing test compounds in a final DMSO concentration of 1%. Compounds were tested in duplicates at a single concentration or, for CC_{50} determination, at 8 concentrations that were prepared *via* 2-fold serial dilutions in 1% DMSO/medium. Epirubicin and doxorubicin were used as positive controls in serial dilutions starting from 10 µM, and rifampicin was used as a negative control (at 100 µM). The living cell mass was determined 48 h after treatment with compounds by adding 0.1 volumes of 3-(4,5-dimethylthiazol-2-yl)-2,5-diphenyltetrazolium bromide (MTT) solution (5 mg/mL sterile PBS) (Sigma, St. Louis, MO) to the wells. After incubating the cells at 37 °C for 30 min (atmosphere containing 5% CO_2), medium was removed and MTT crystals were dissolved in 75 µL of a solution containing 10% SDS and 0.5% acetic acid in DMSO. The optical density (OD) of the samples was determined photometrically at 570 nm in a PHERAstar Omega plate reader (BMG labtech, Ortenberg, Germany). To obtain percent viability for each sample, their ODs were related to those of DMSO controls. At least three independent measurements were performed for each compound. The calculation of CC_{50} was performed using the nonlinear regression function of GraphPad Prism 10 (GraphPad Software, San Diego, CA, USA).

Copies of NMR spectra

S-(2-Acetamidoethyl) butanethioate (20)

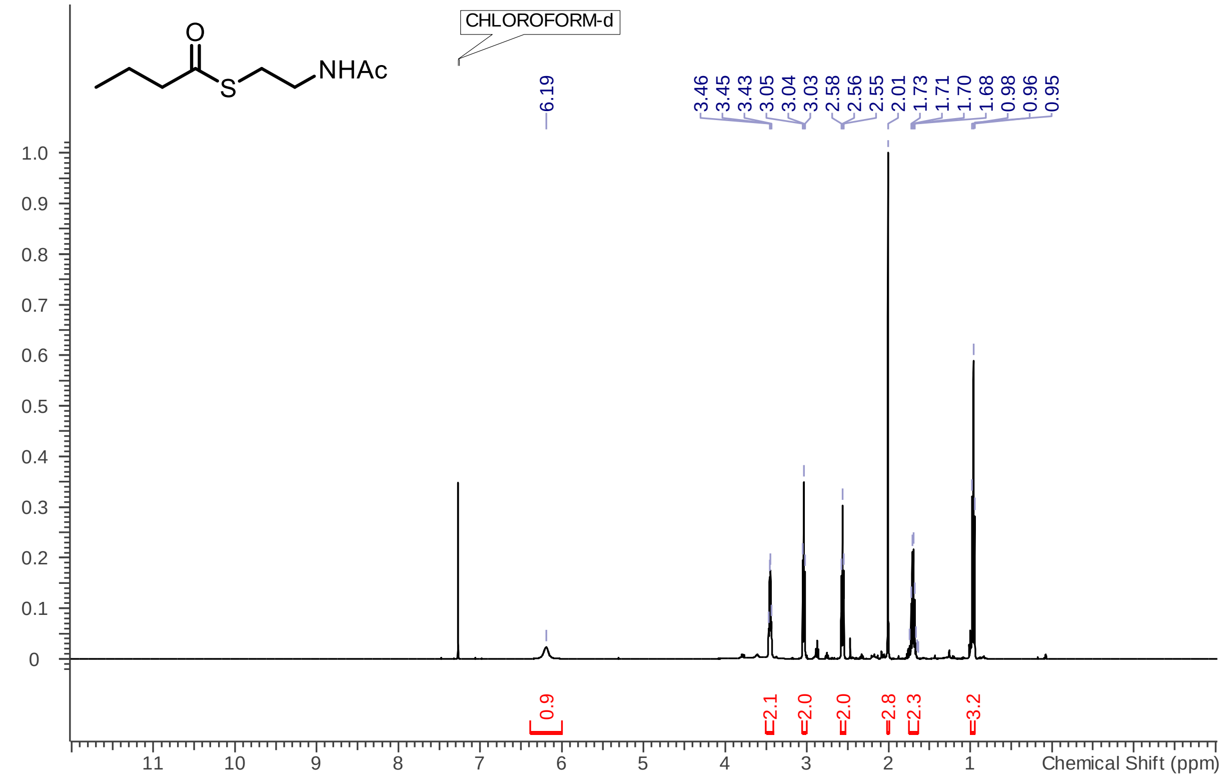

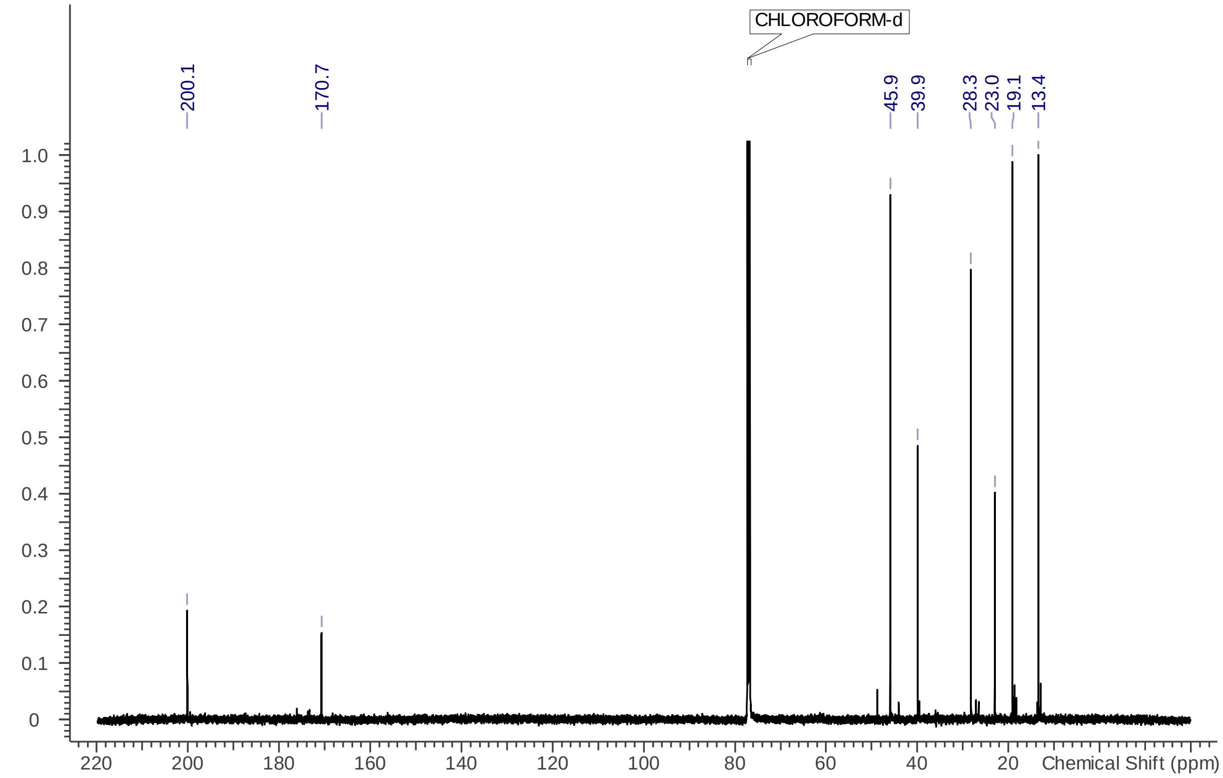

S-(2-Acetamidoethyl) pentanethioate (21)

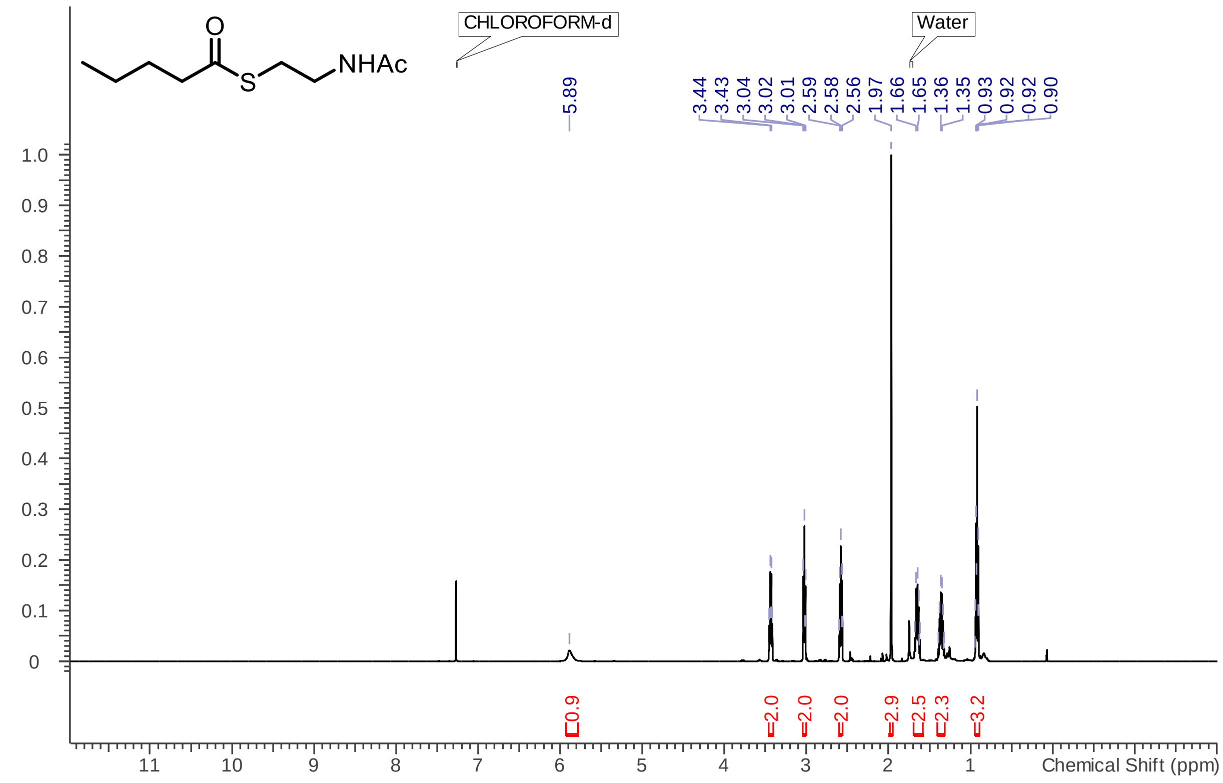

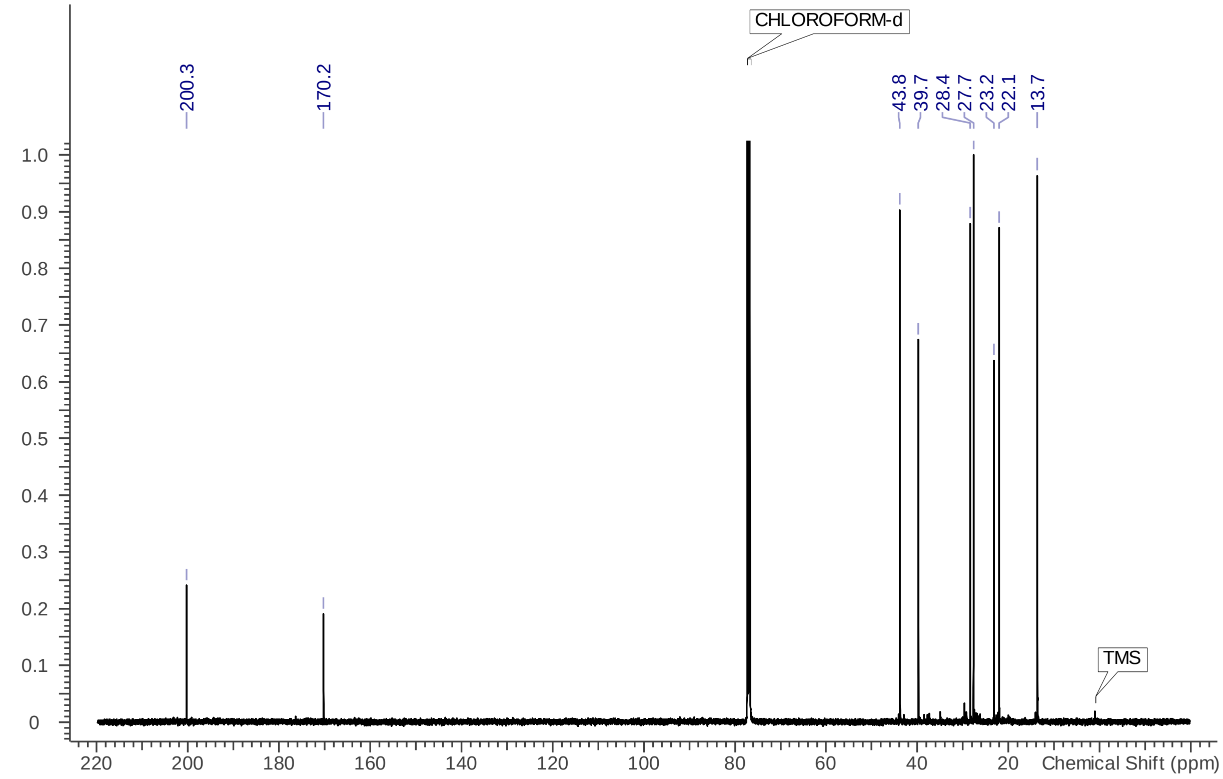

S-(2-Acetamidoethyl) 4,4,4-trifluorobutanethioate (22)

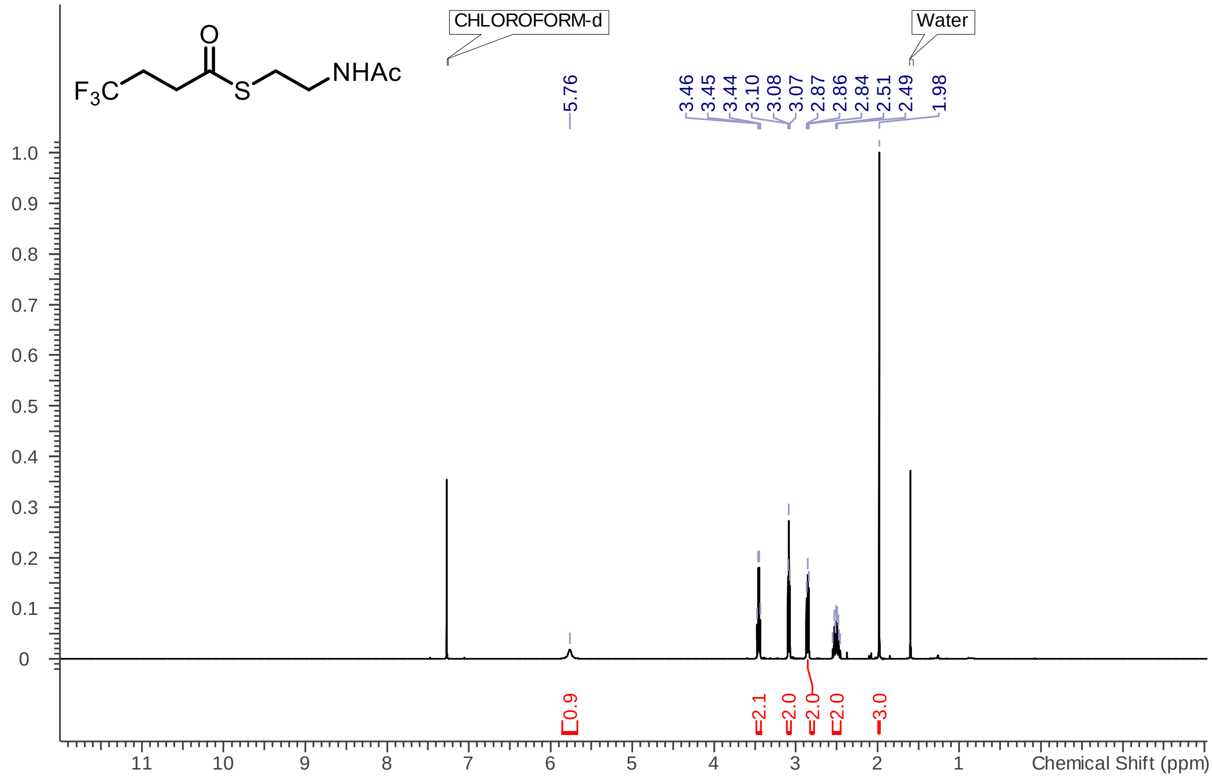

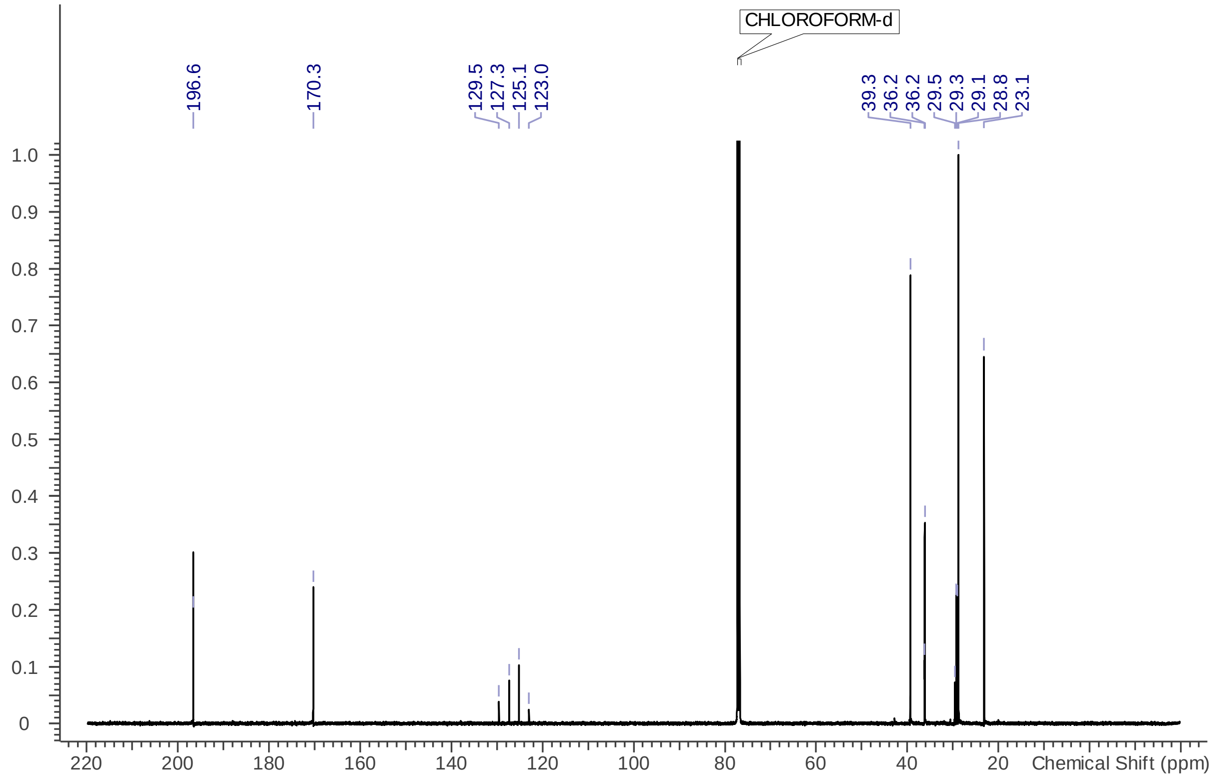

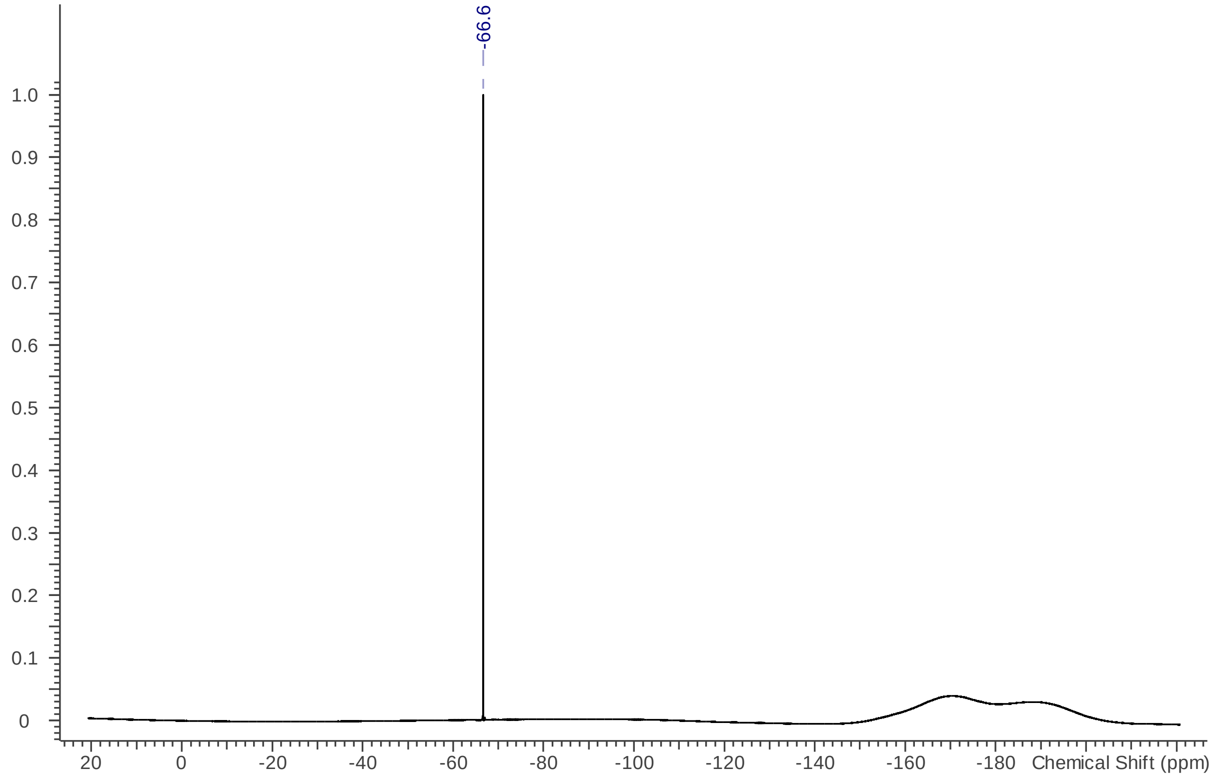
-66.6
1.0
0.9
0.8
0.7
0.6
0.5
0.4
0.3
0.2
0.1
0
20
0
-20
-40
-60
-80
-100
-120
-140
-160
-180
Chemical Shift (ppm)

S-(2-Acetamidoethyl) pent-4-ynethioate (23)

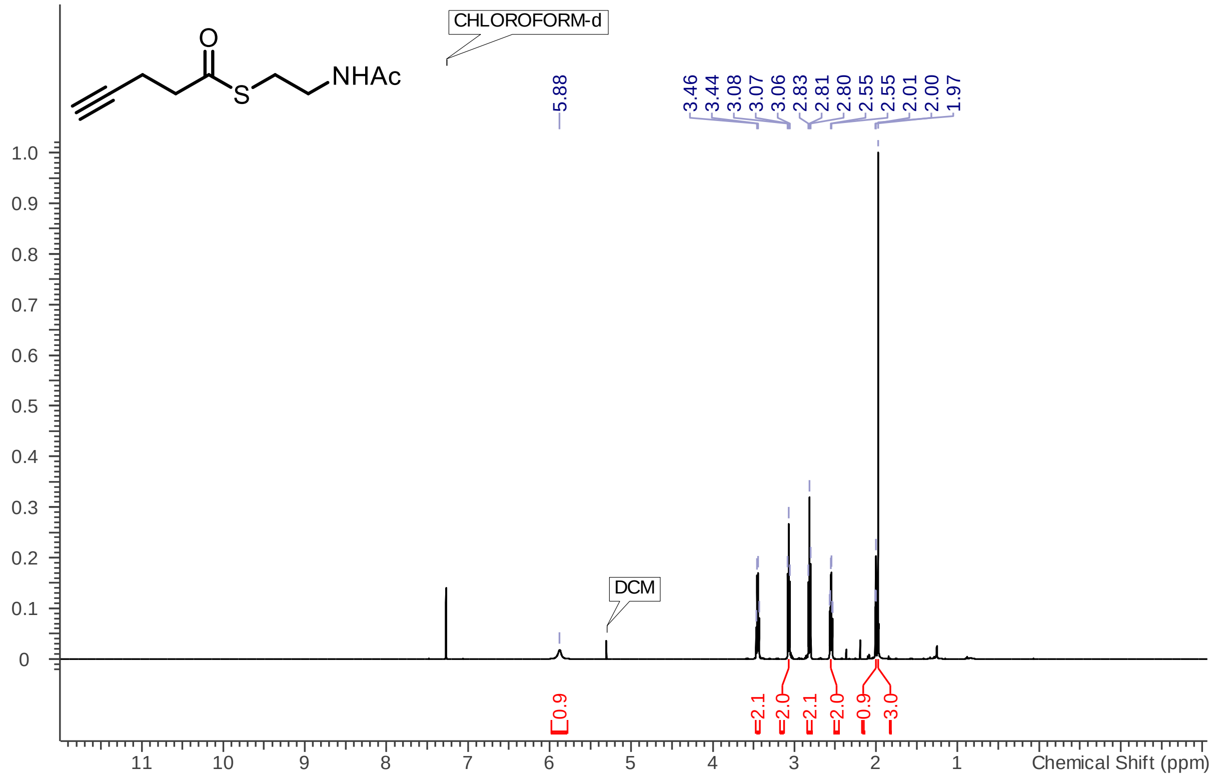

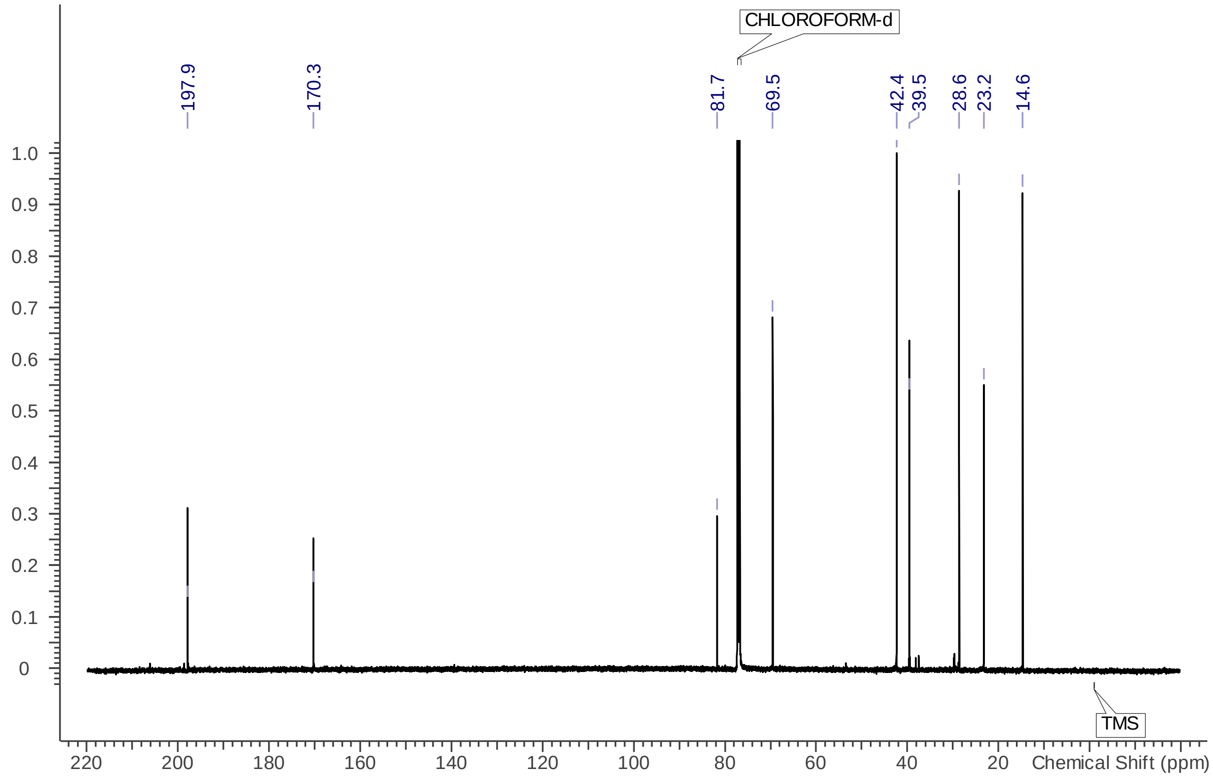

S-(2-acetamidoethyl) cyclohexanecarbothioate (24)

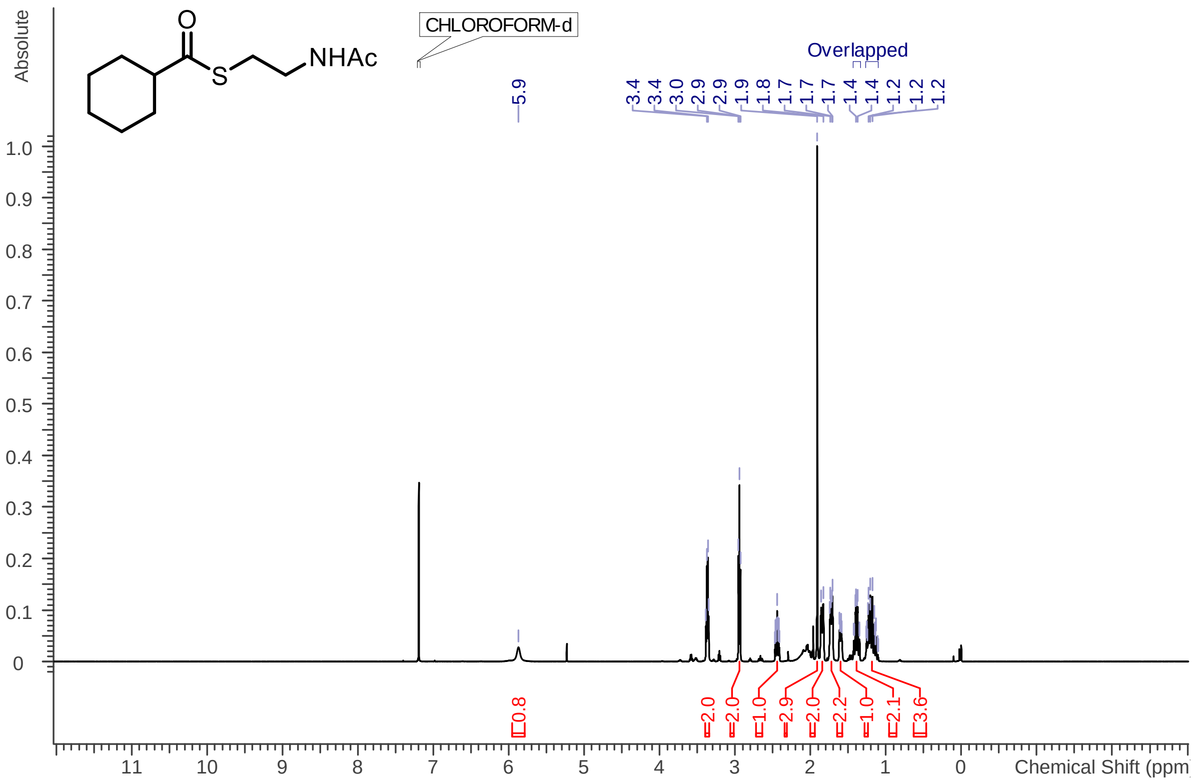

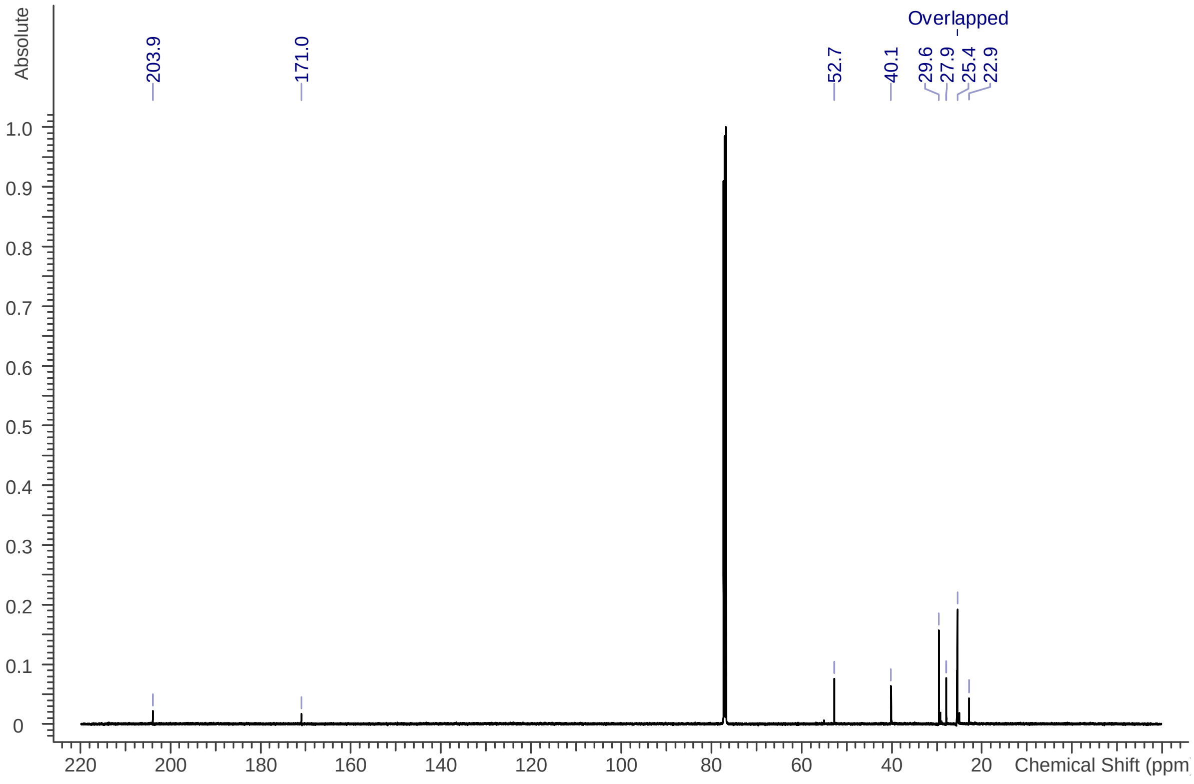

S-(2-Acetamidoethyl) benzothioate (25)

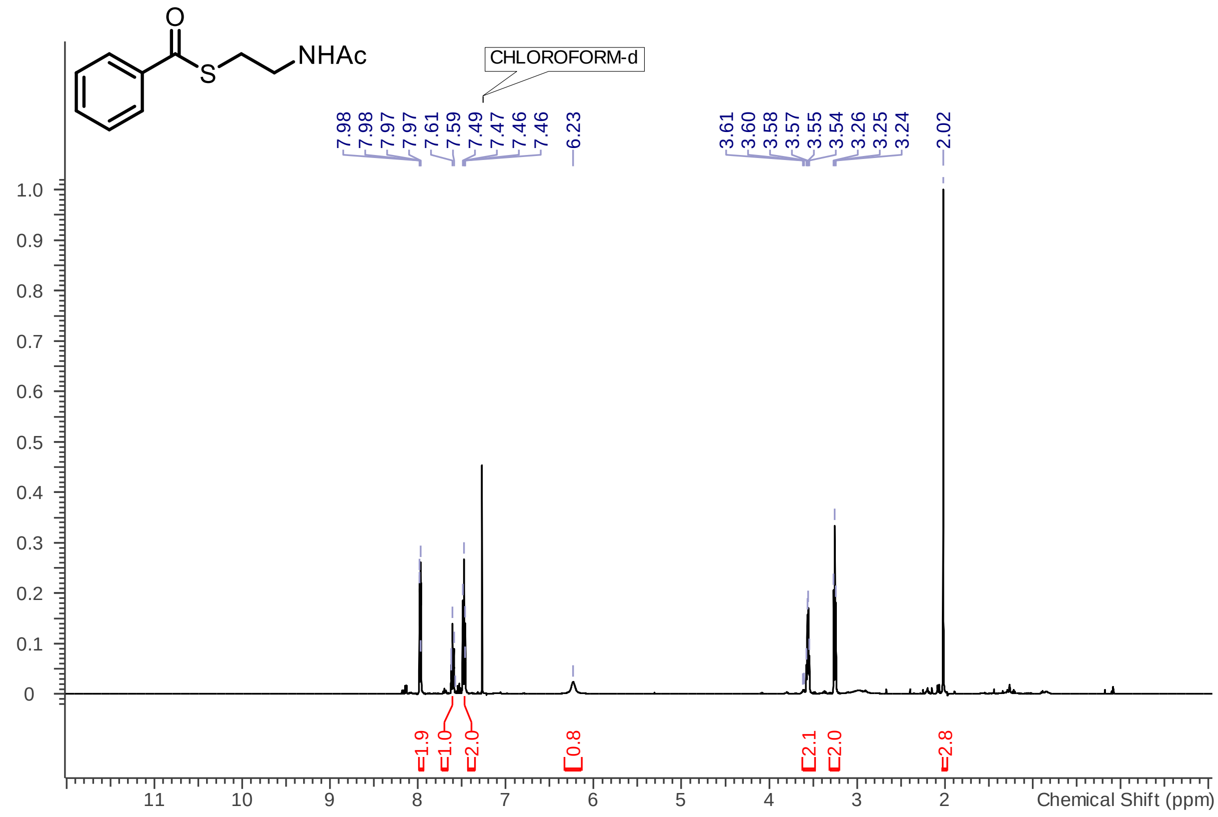

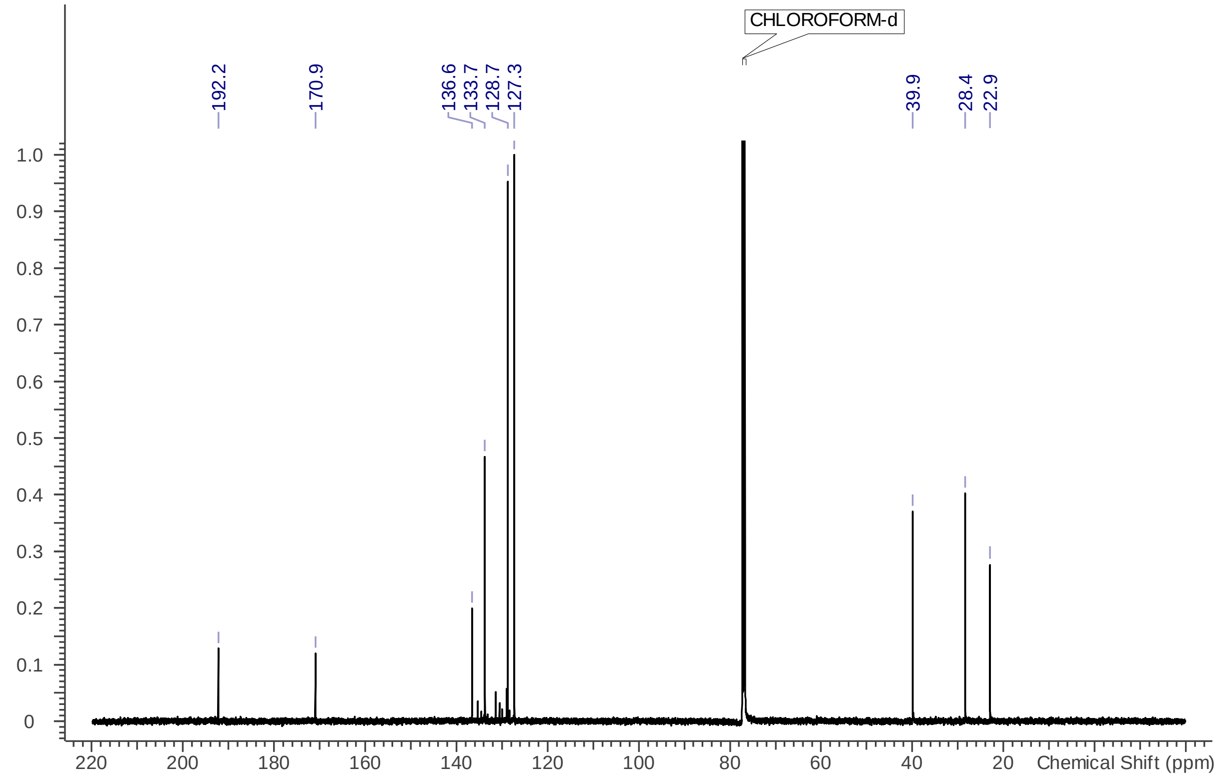

S-(2-Acetamidoethyl) pyridine-3-carbothioate (26)

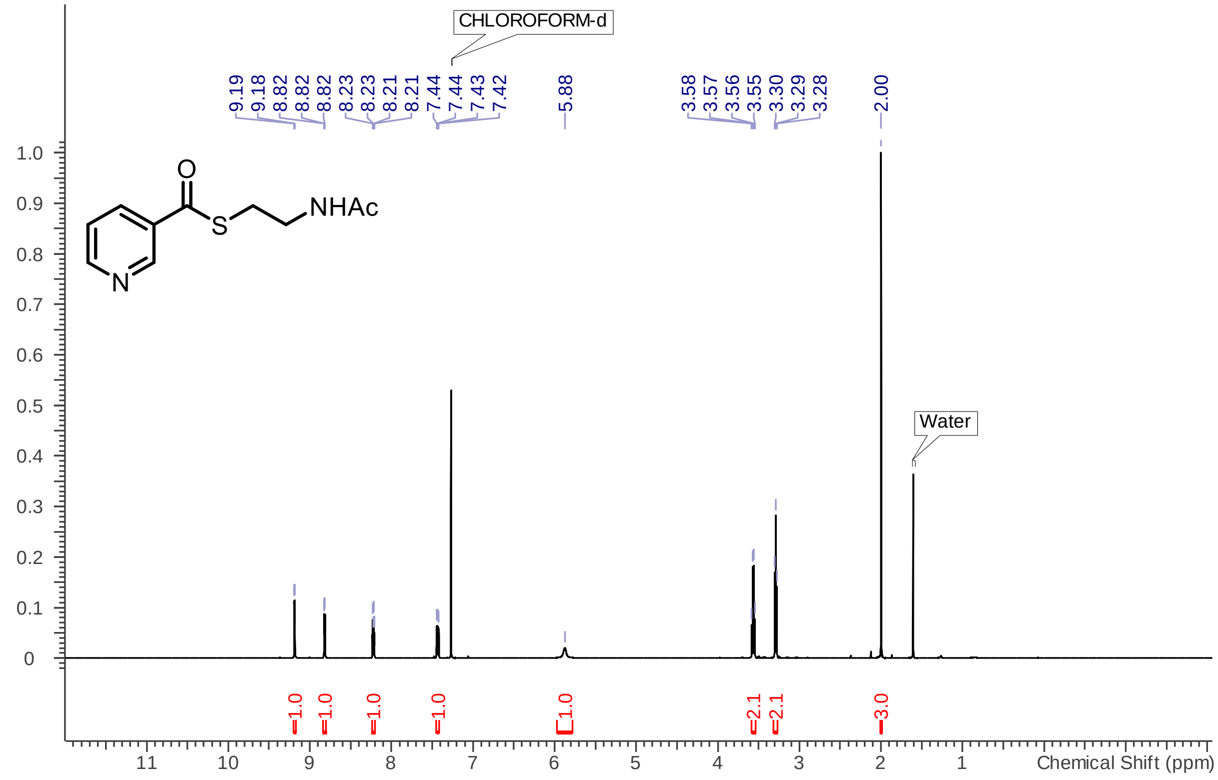

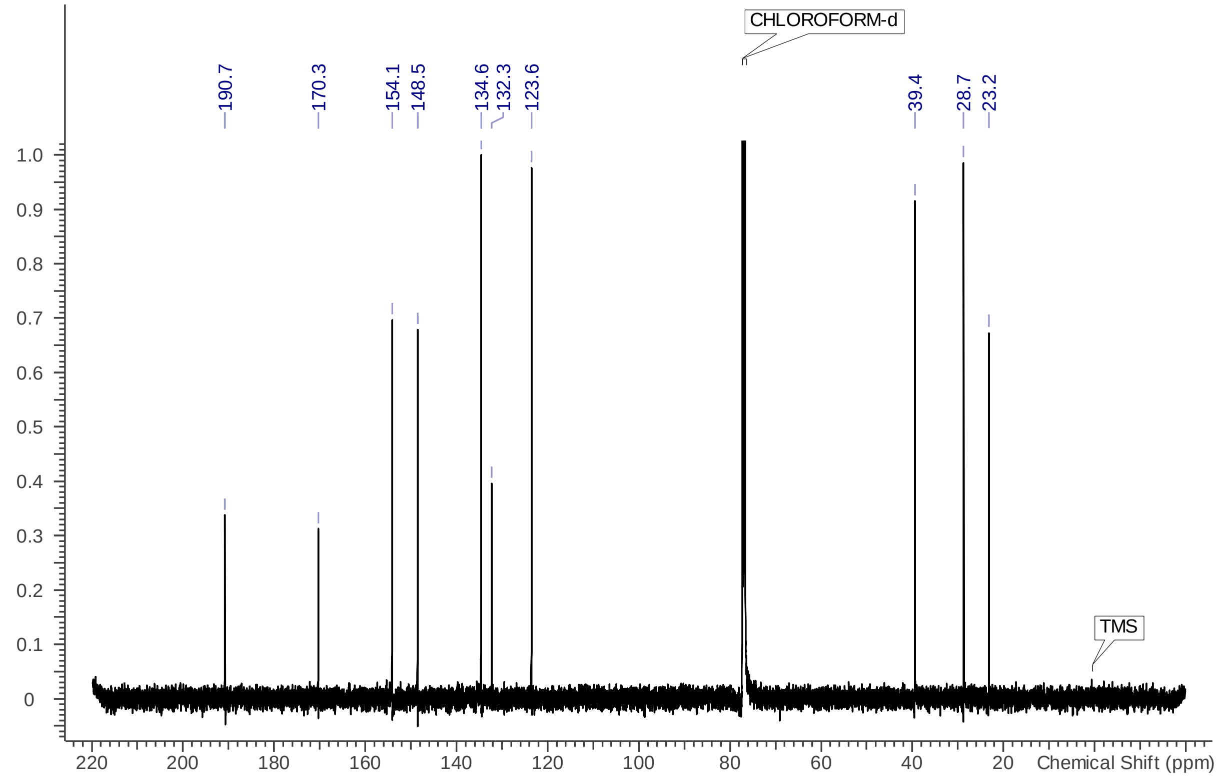

S-(2-Acetamidoethyl) furan-2-carbothioate (27)

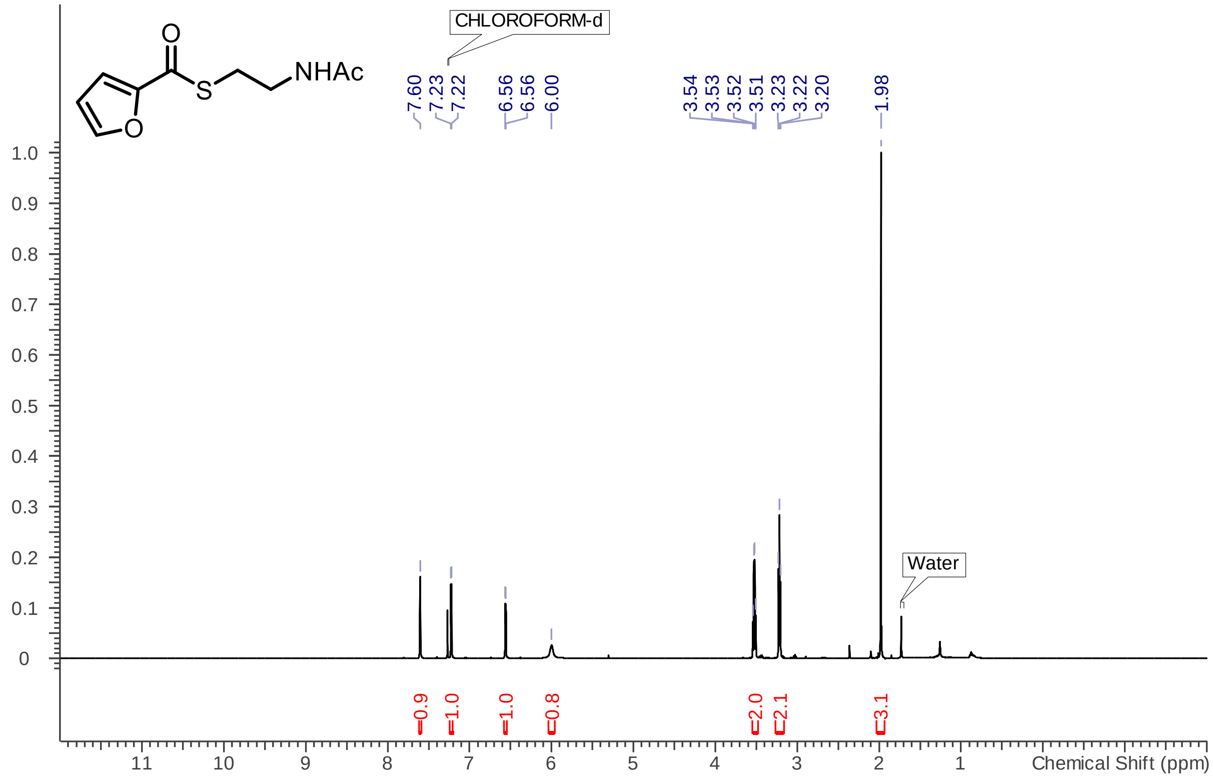

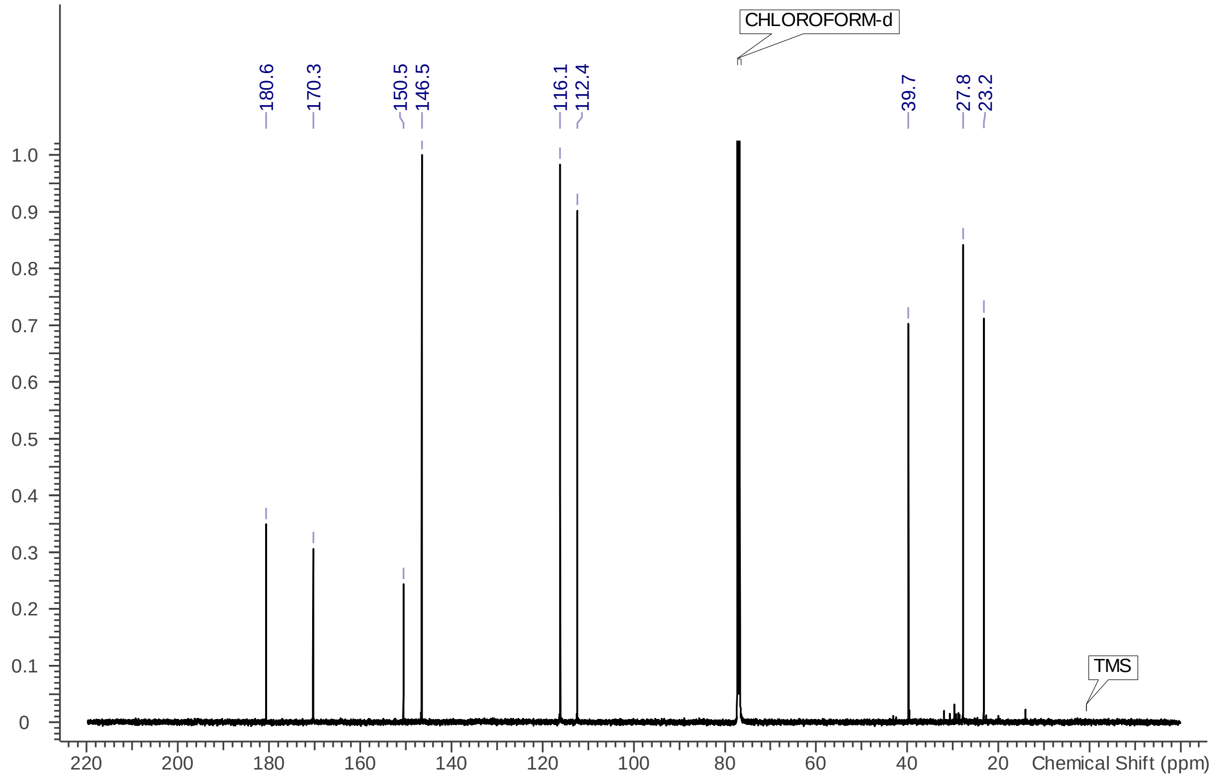

S-(2-Acetamidoethyl) furan-3-carbothioate (28)

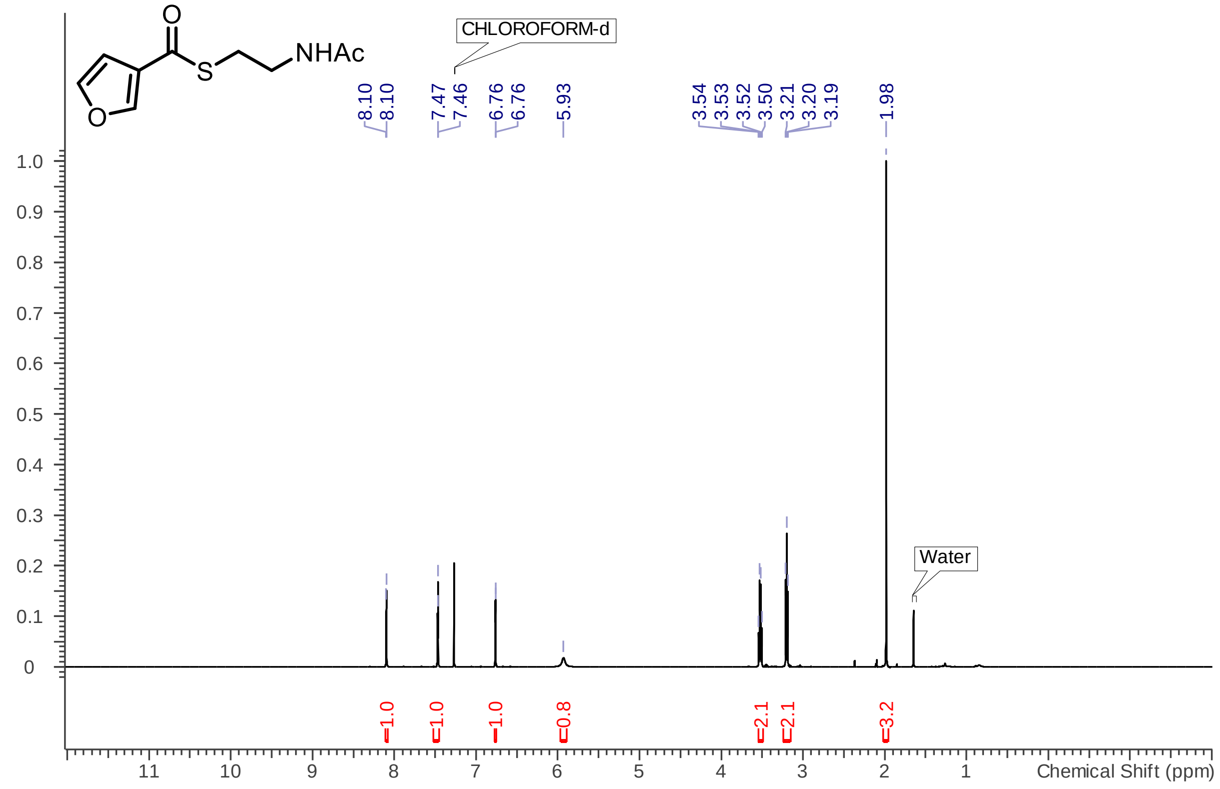

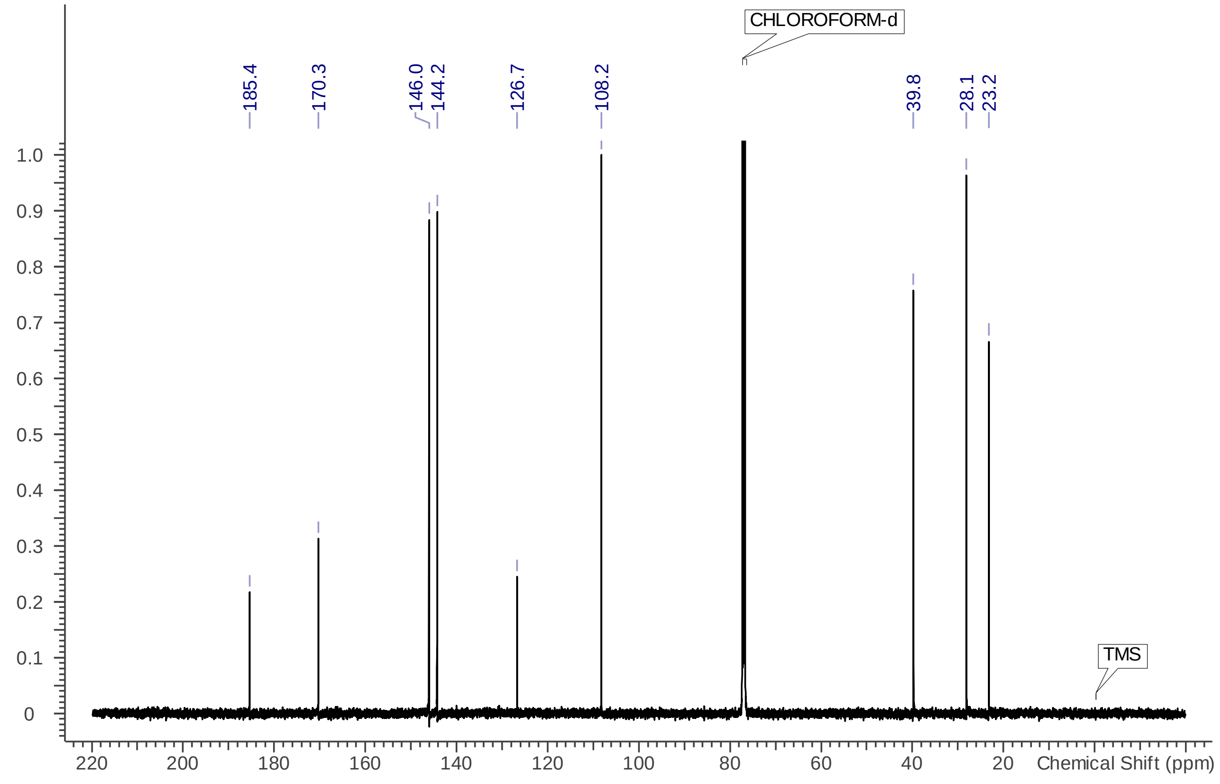

***S*-(2-Acetamidoethyl) thiophene-3-carbothioate (29)**

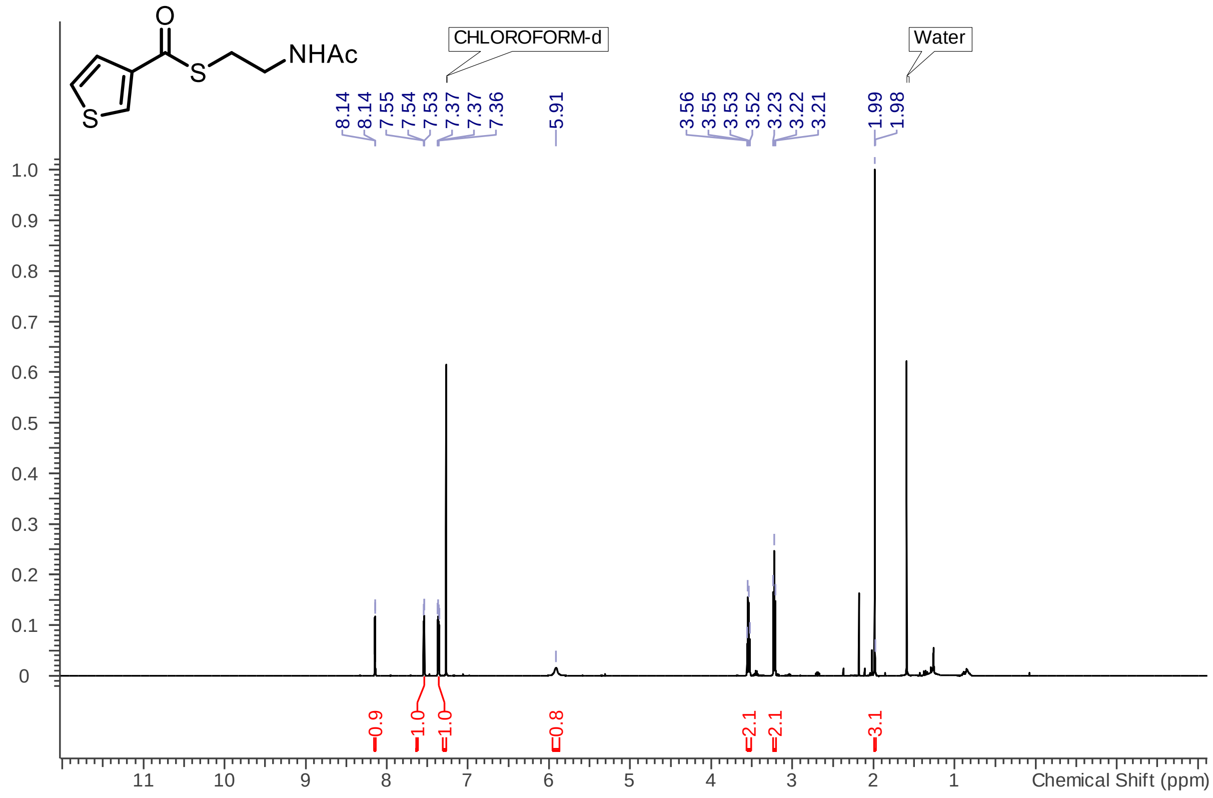

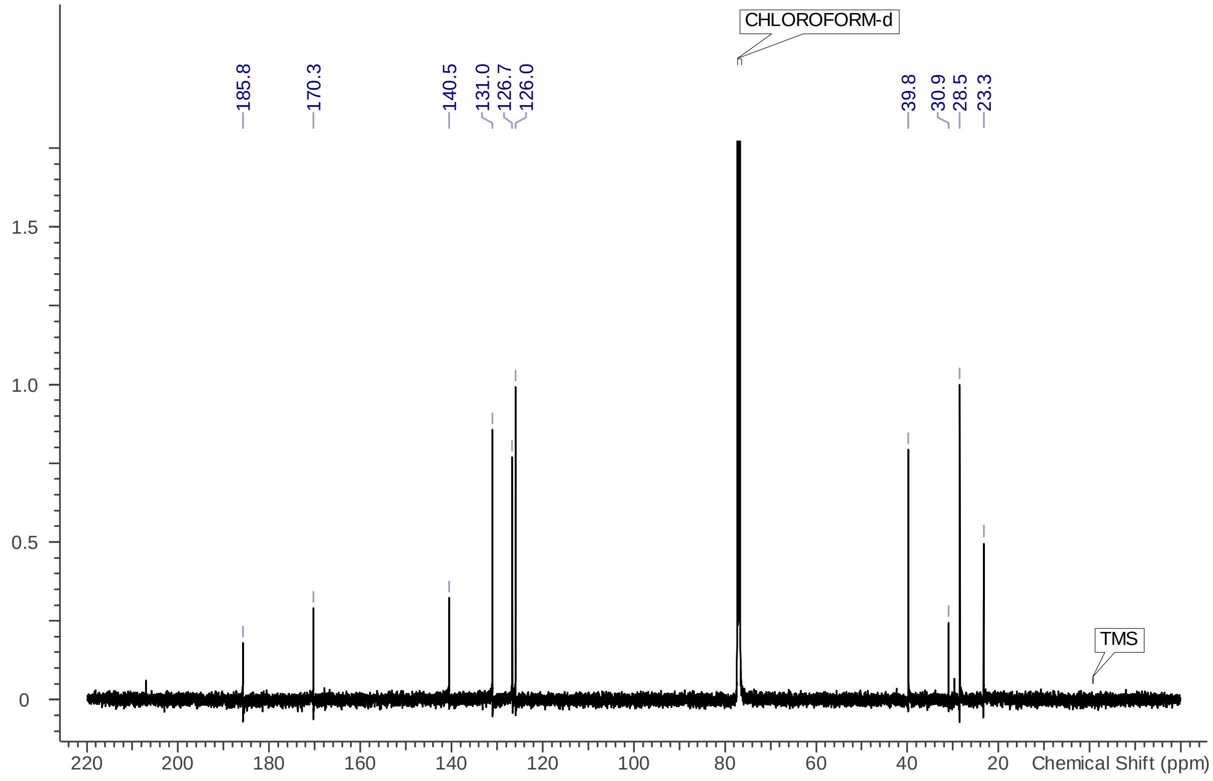

S-(2-Acetamidoethyl) oxazole-4-carbothioate (30)

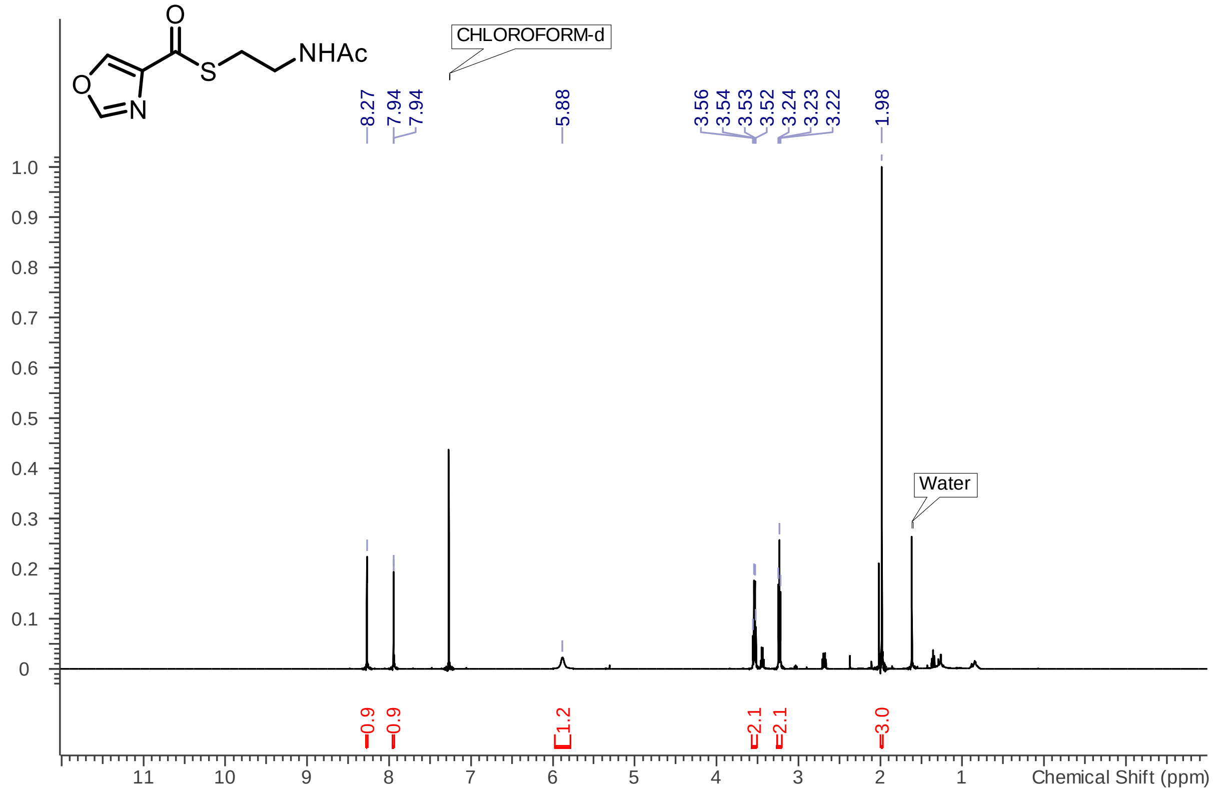

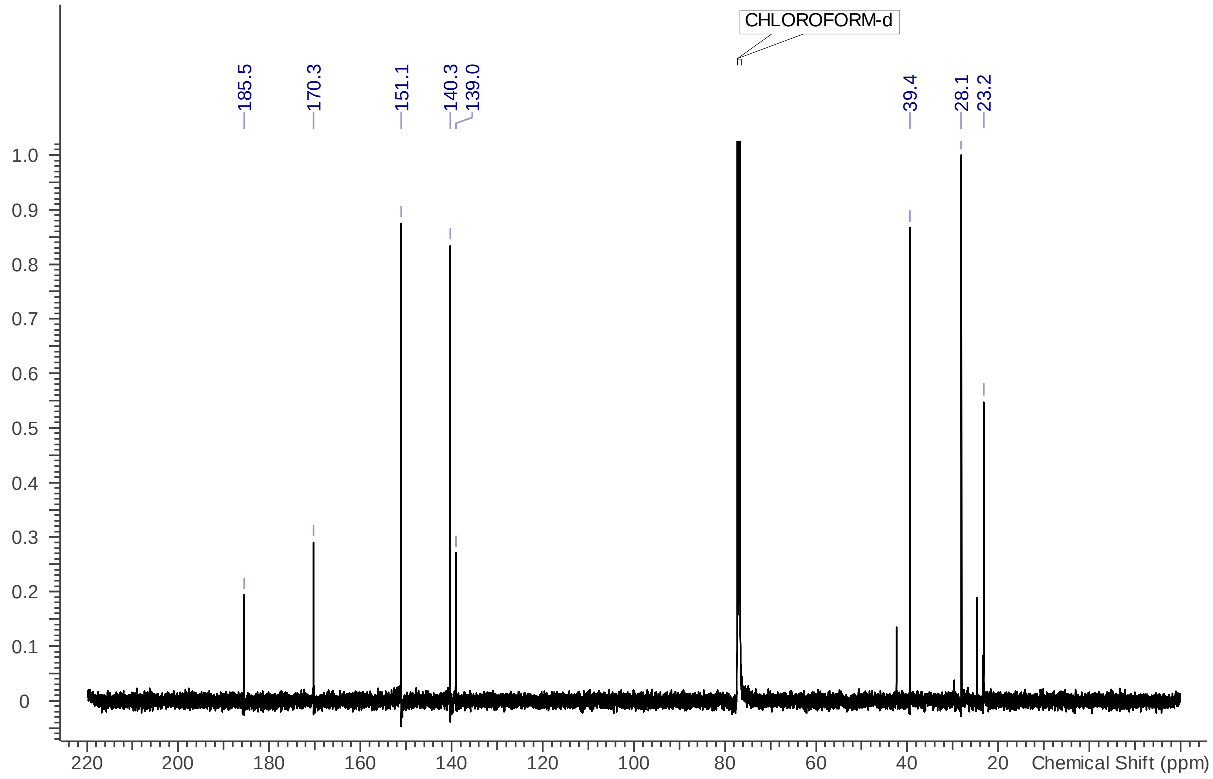

S-(2-Acetamidoethyl) 2-methyloxazole-4-carbothioate (31)

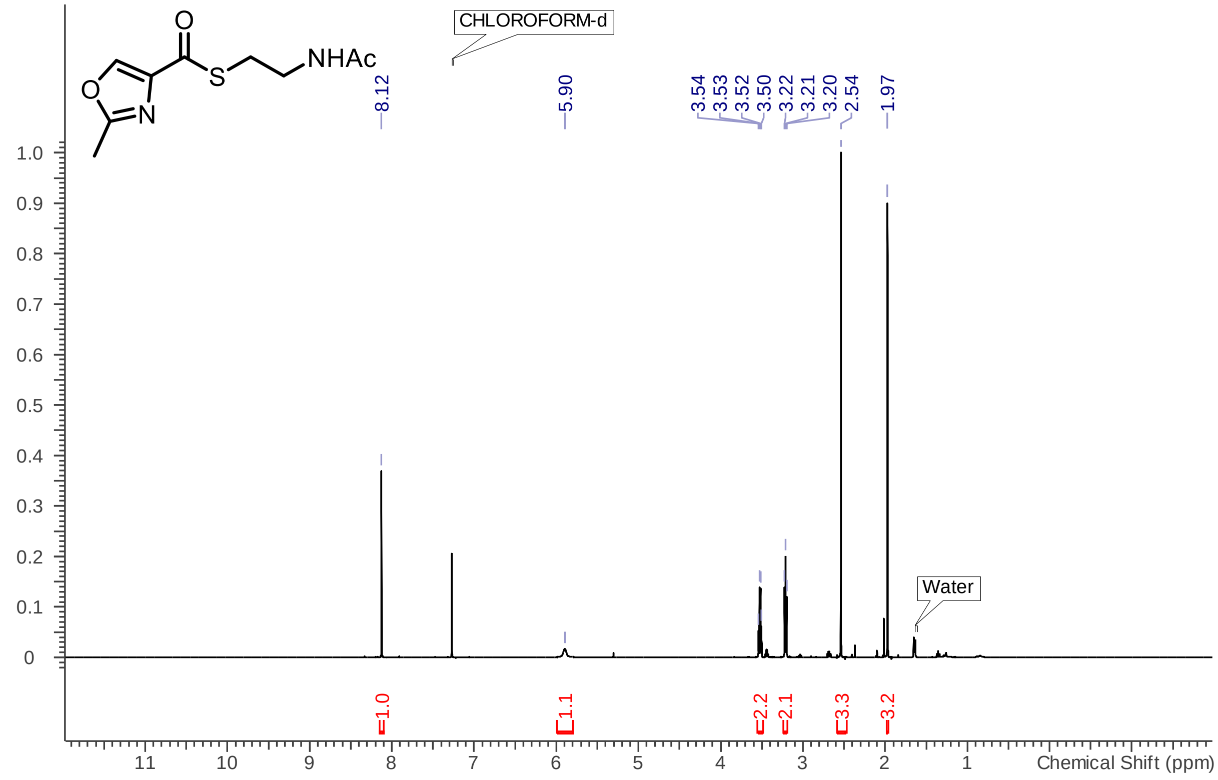

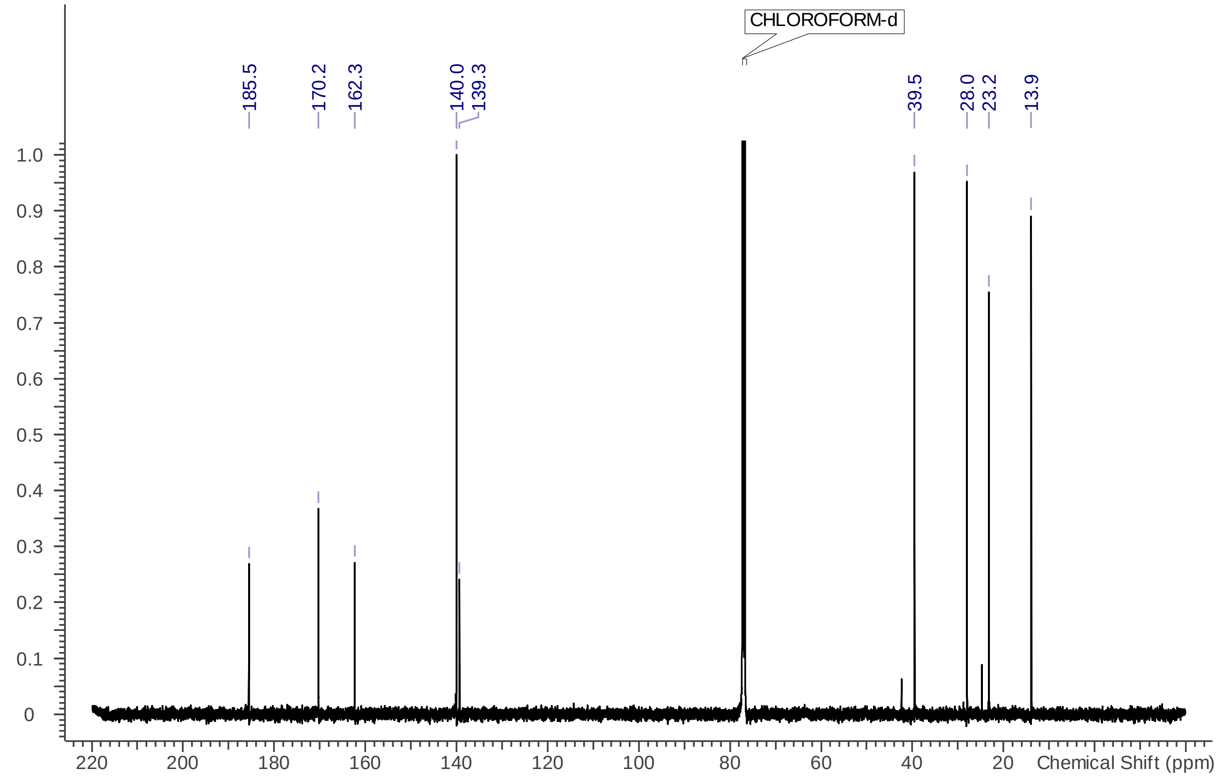

S-(2-Acetamidoethyl) thiazole-4-carbothioate (32)

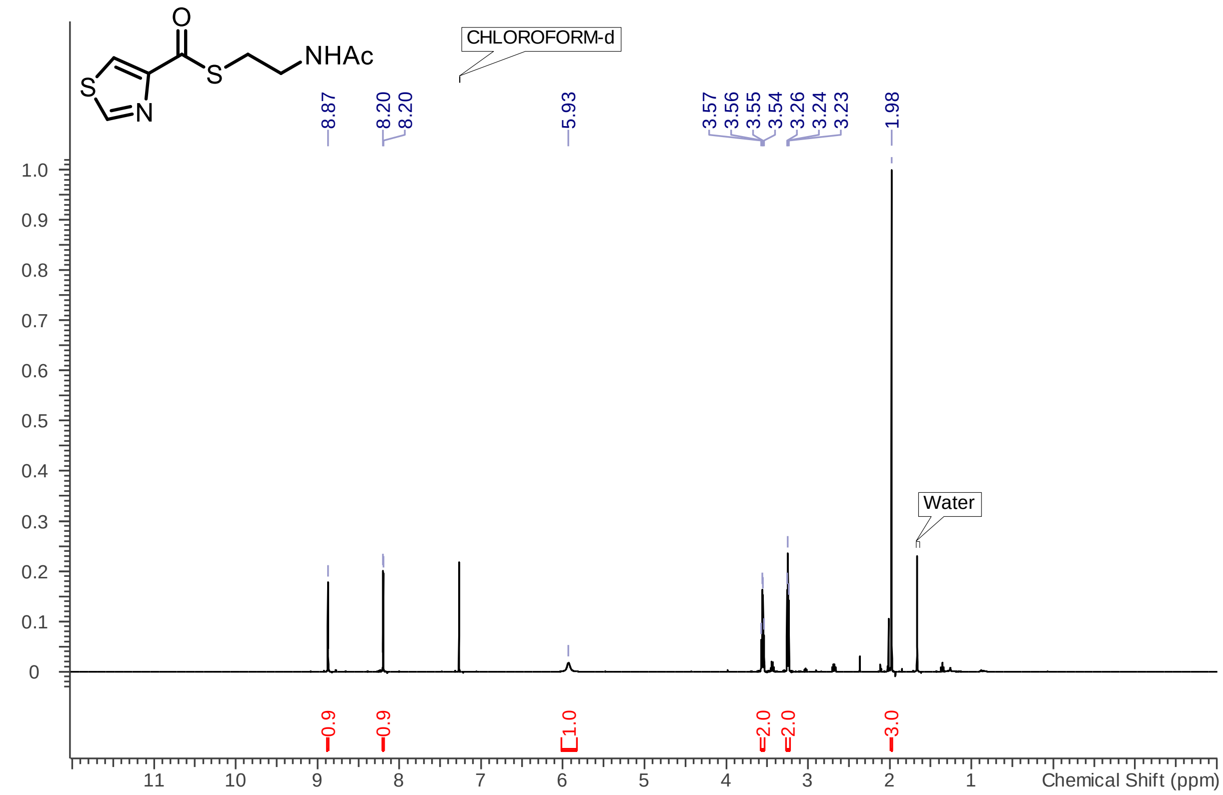

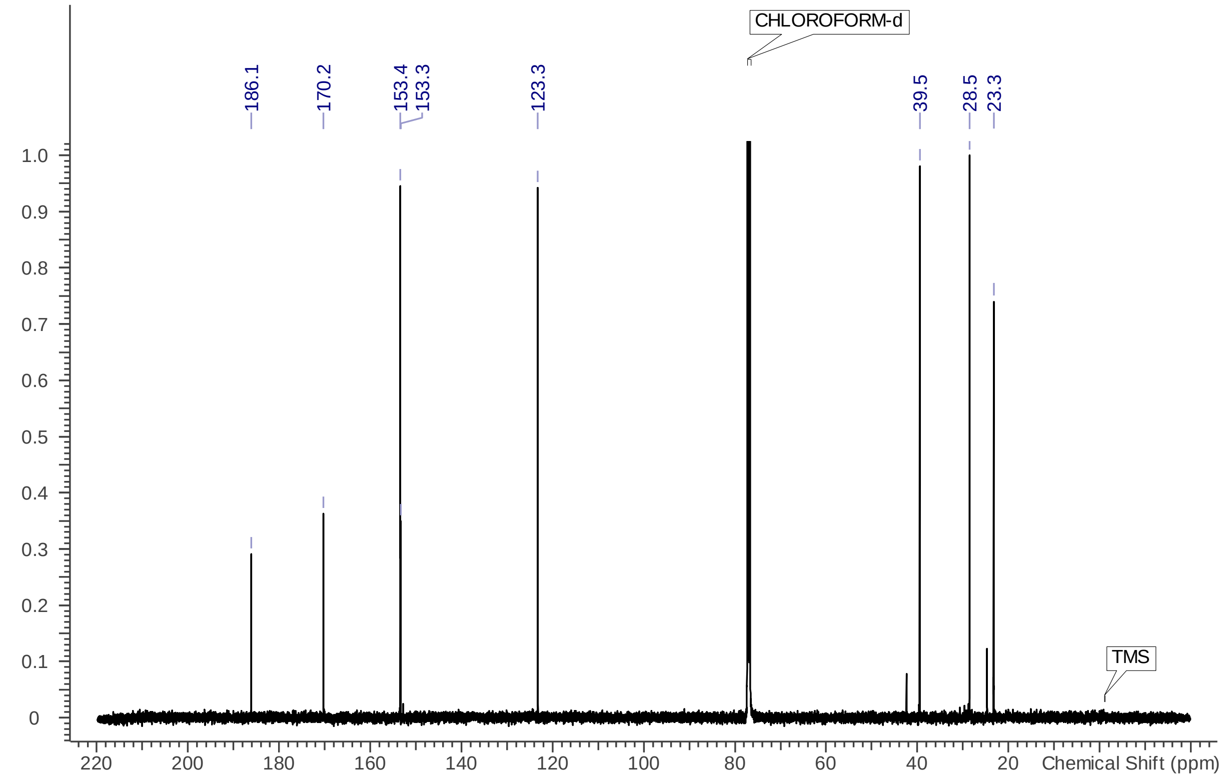

S-(2-Acetamidoethyl) 2-methylthiazole-4-carbothioate (33)

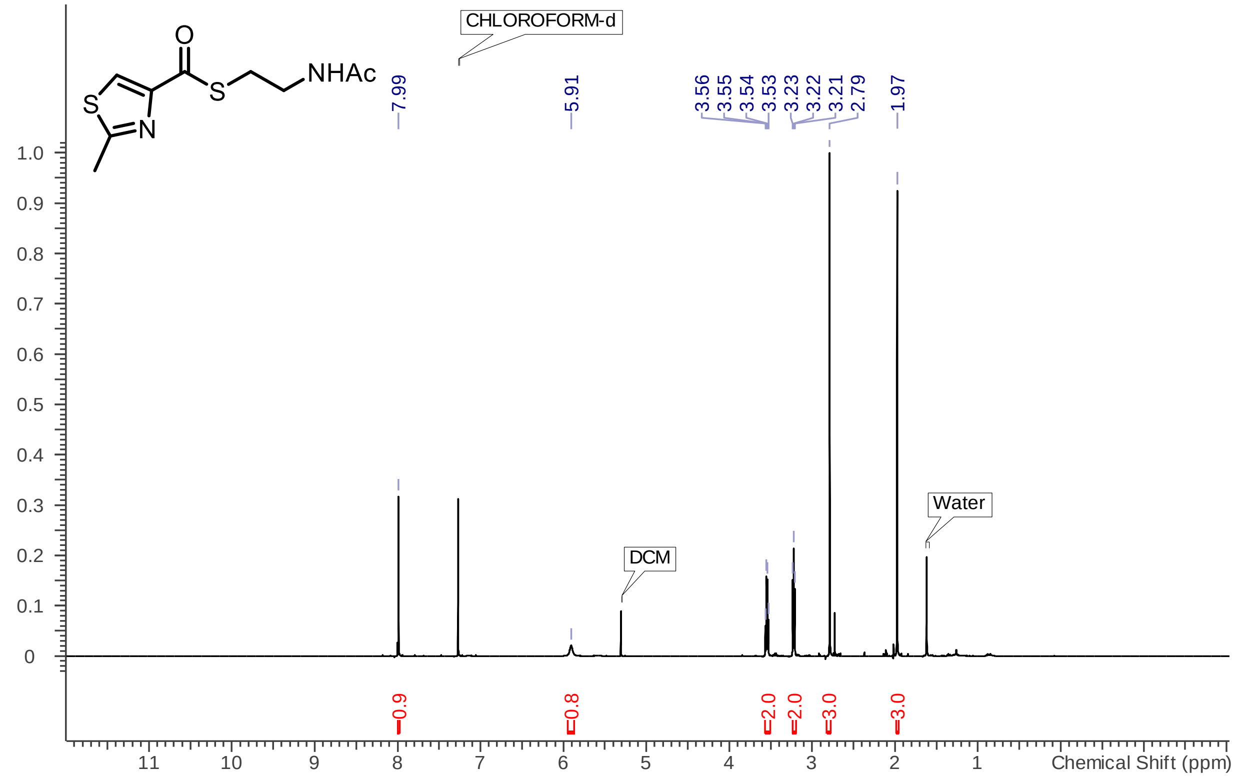

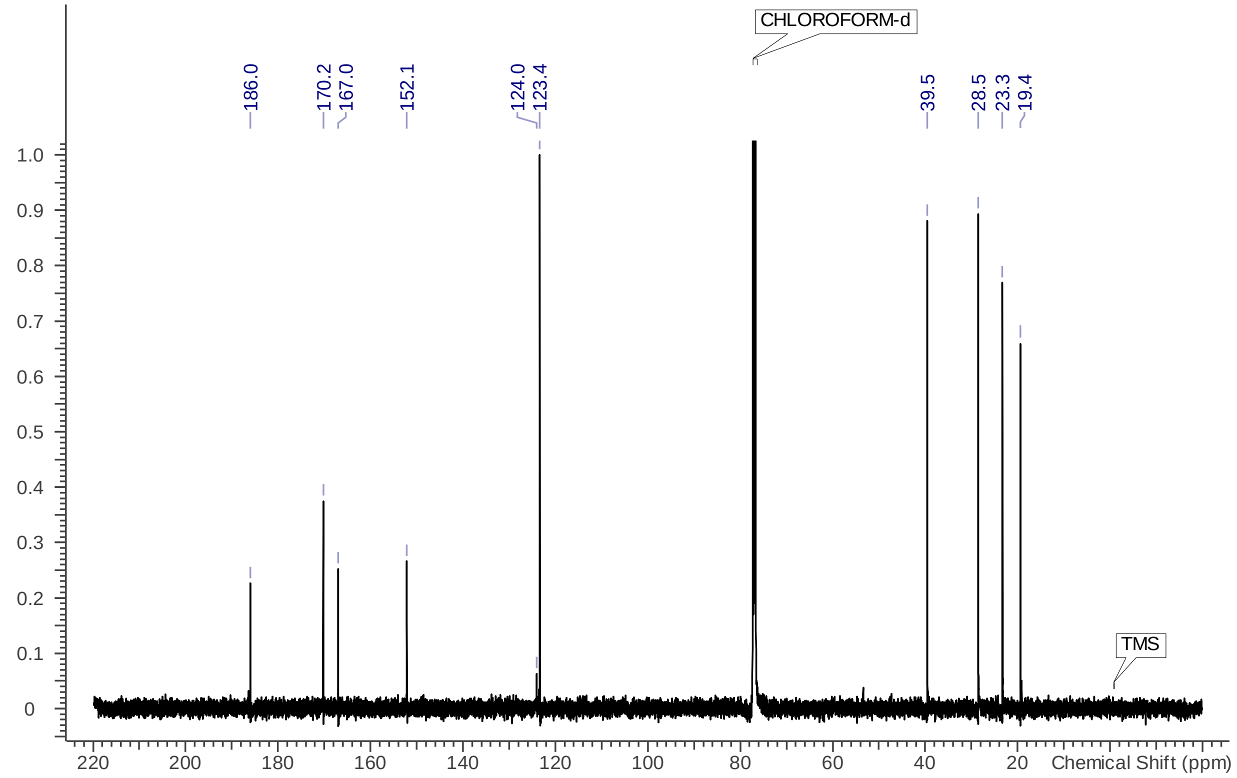

_S_-Phenyl butanethioate (34)

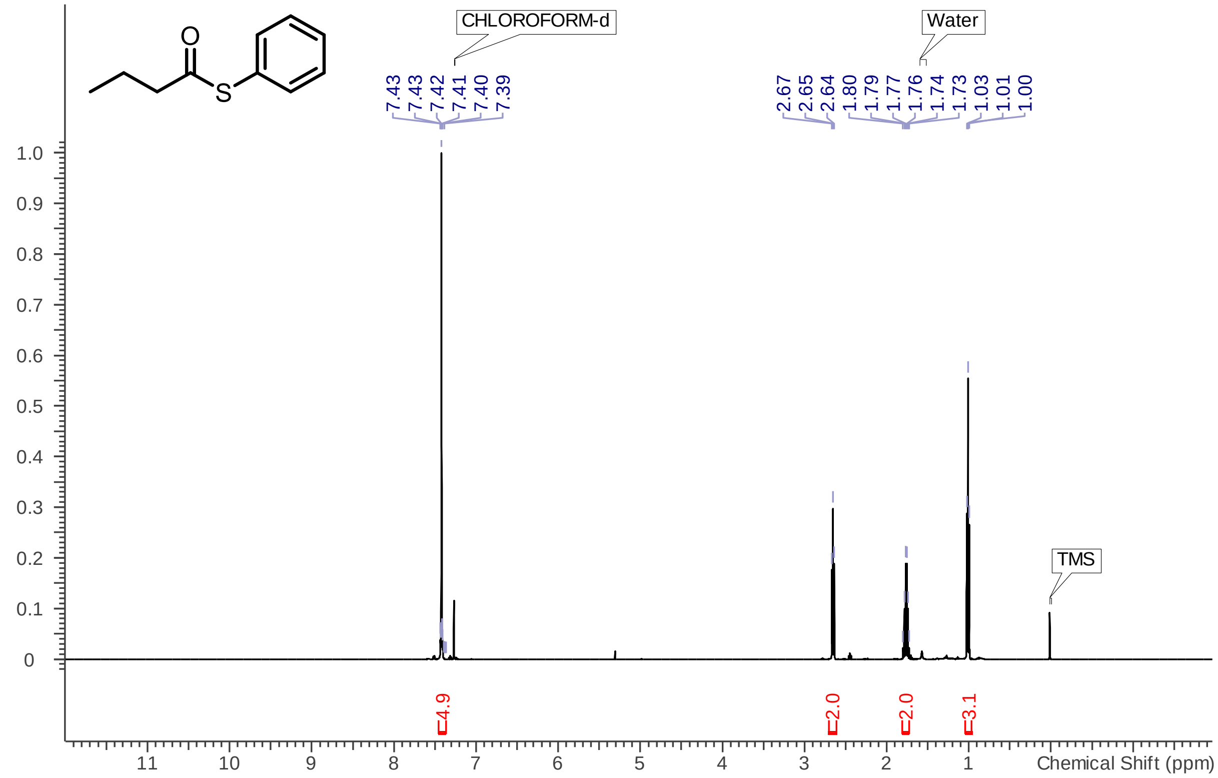

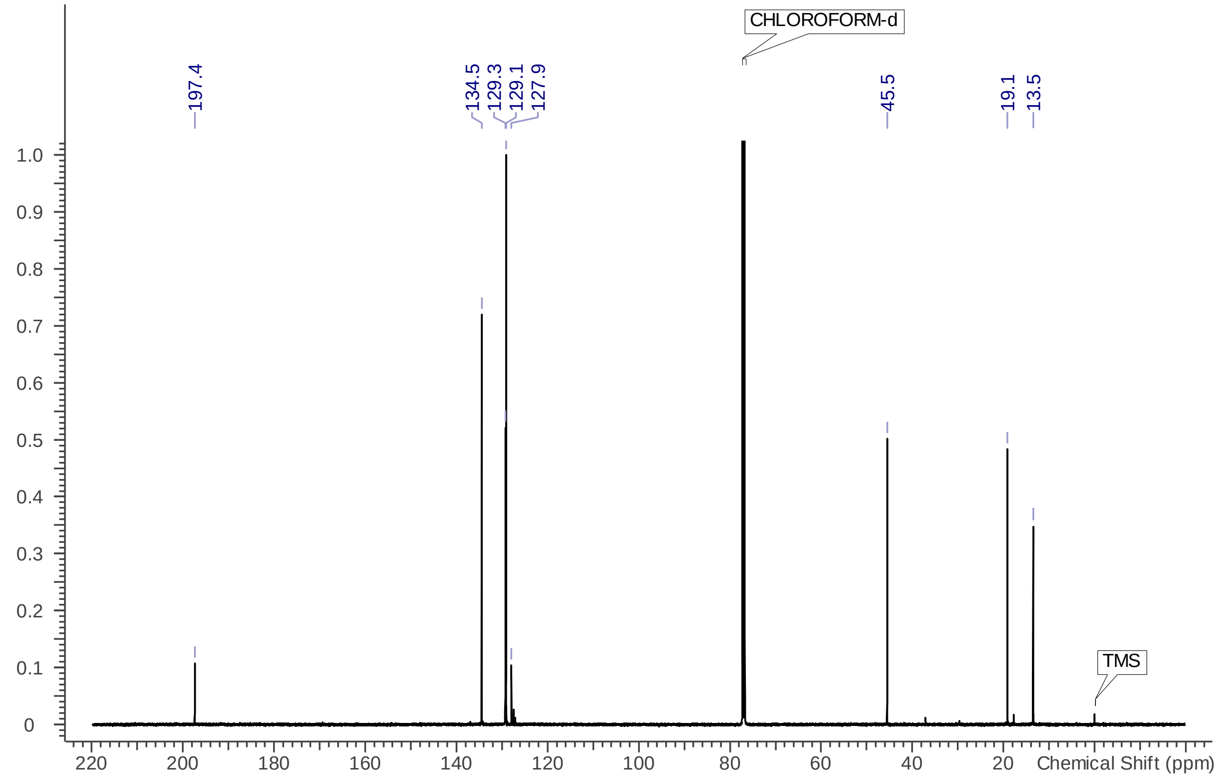

S-Phenyl 4,4,4-trifluorobutanethioate (35)

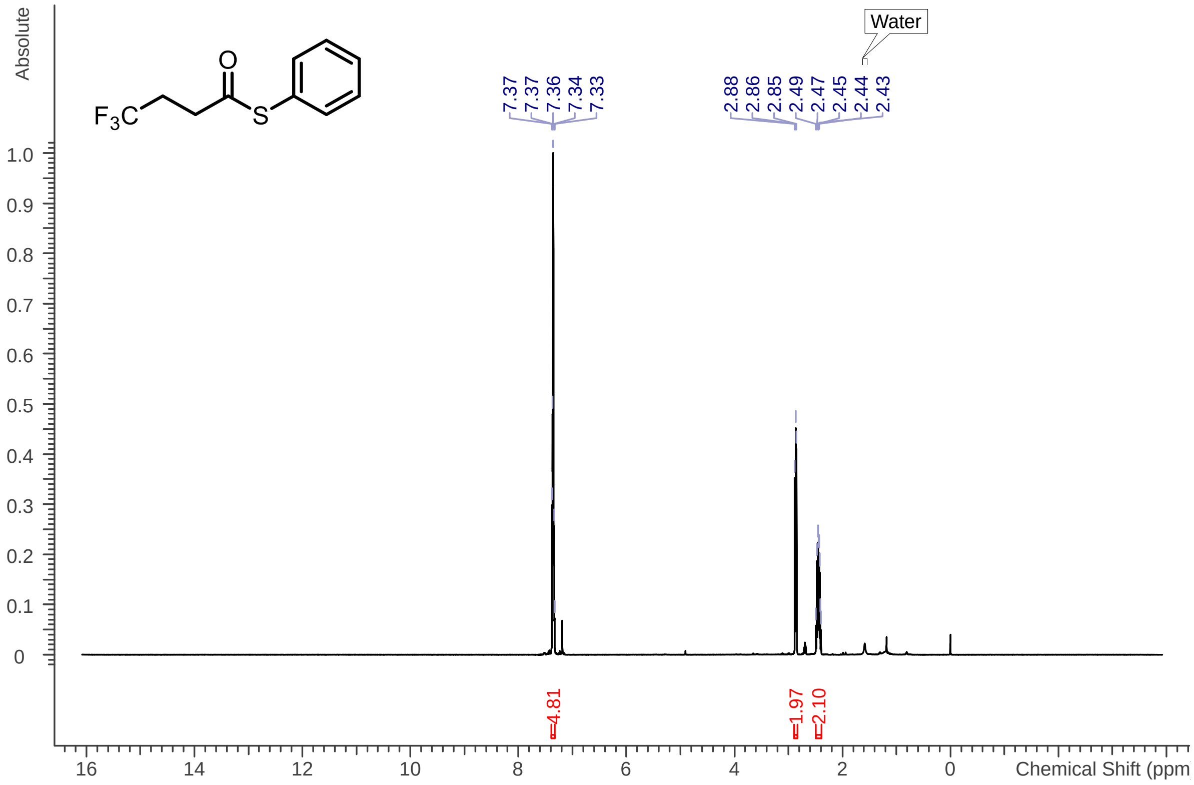

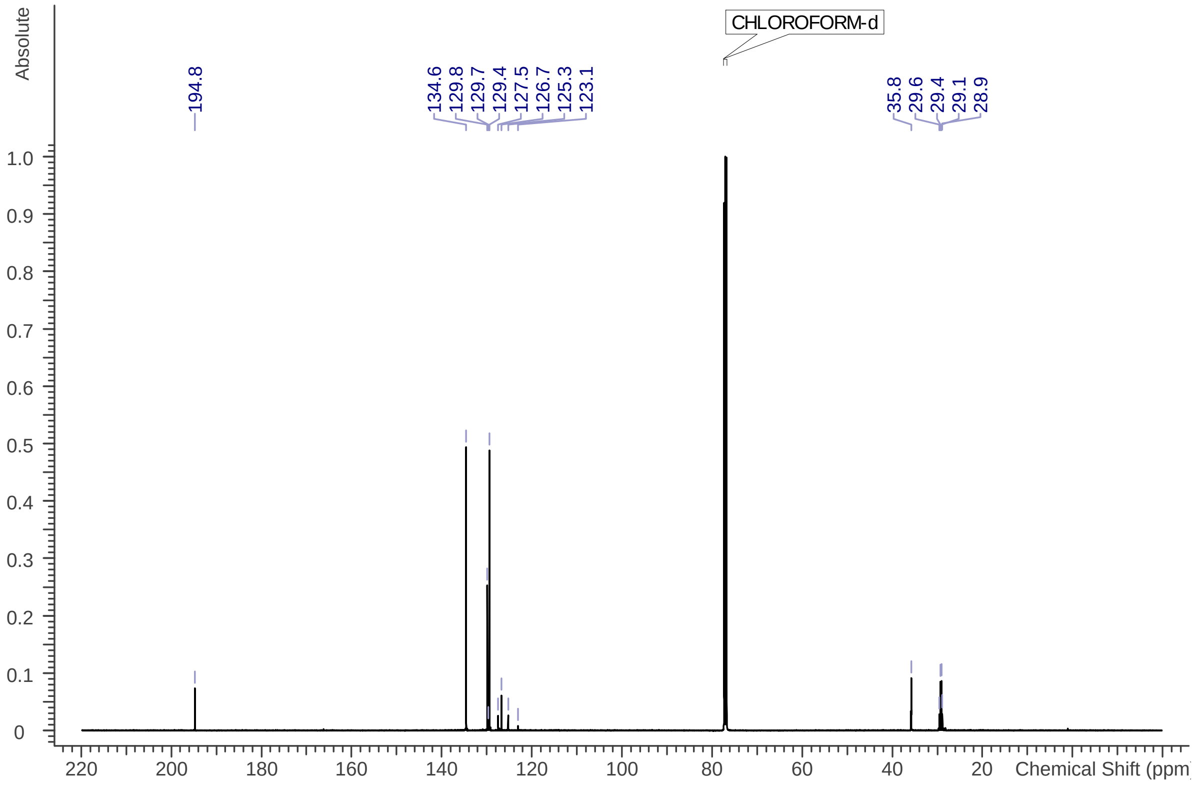

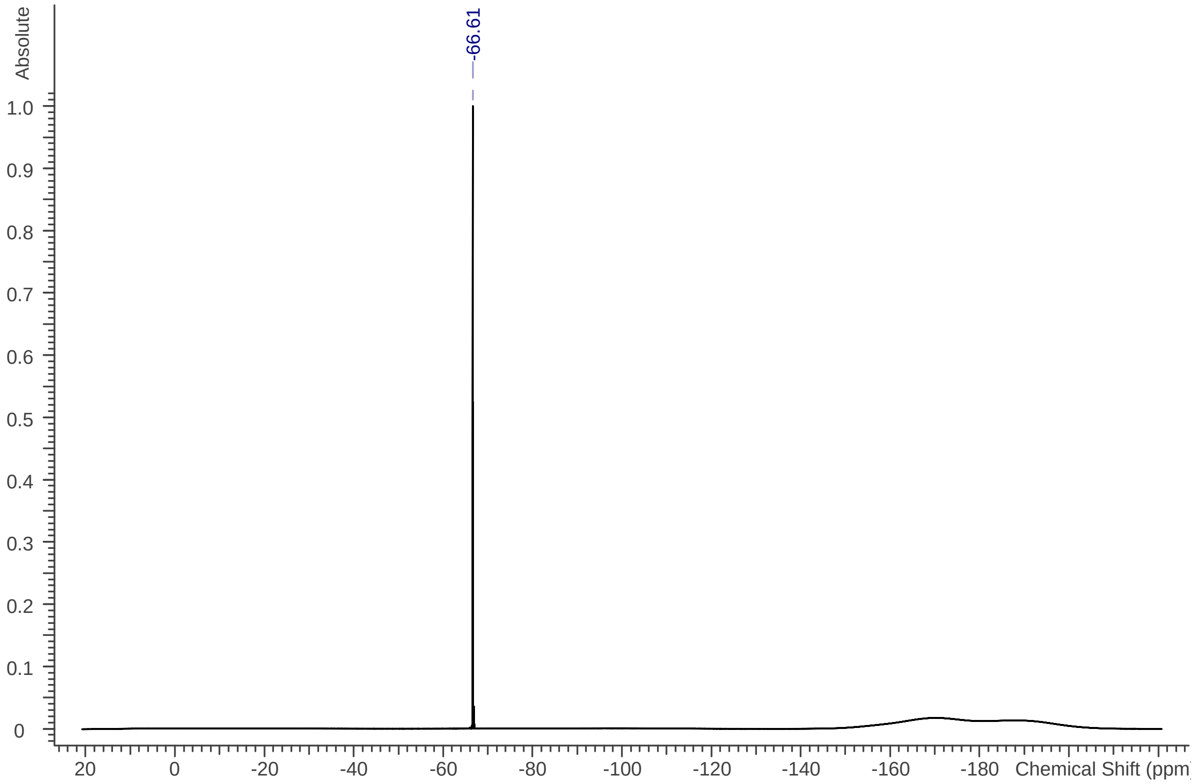
-66.61
Absolute
1.0
0.9
0.8
0.7
0.6
0.5
0.4
0.3
0.2
0.1
0
20
0
-20
-40
-60
-80
-100
-120
-140
-160
-180
Chemical Shift (ppm)

S-Phenyl pent-4-ynethioate (36)

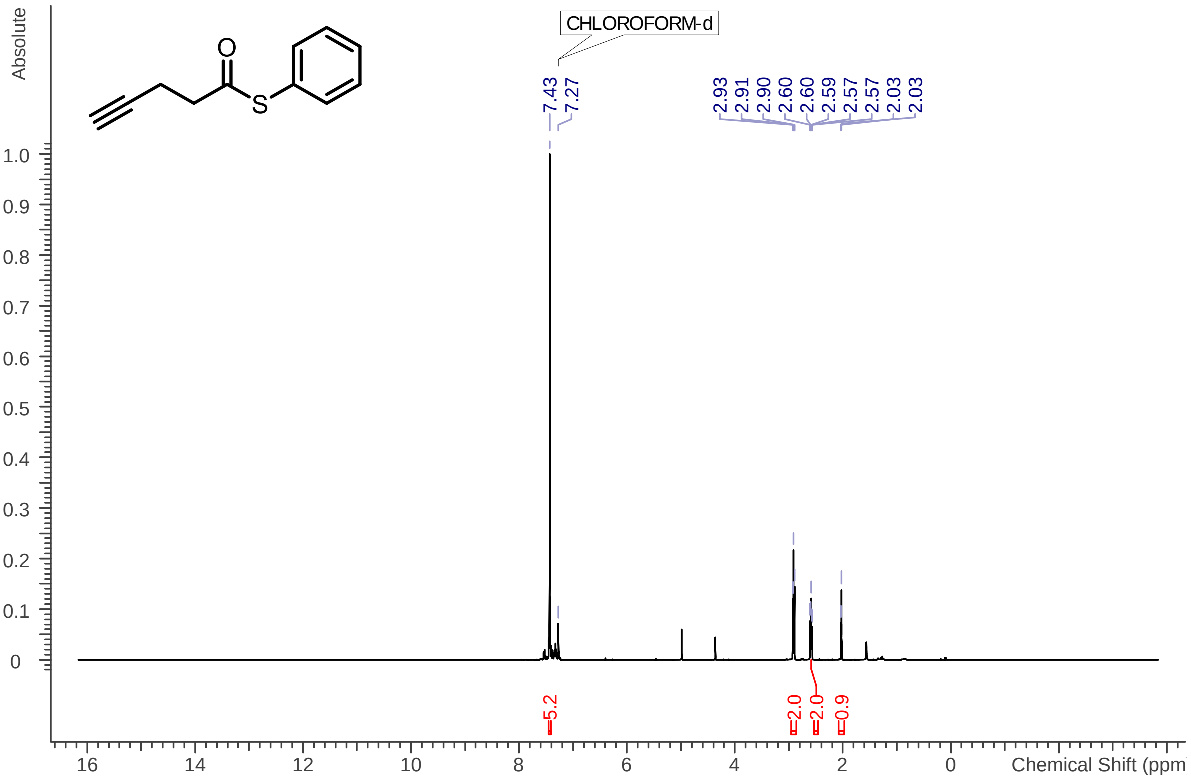

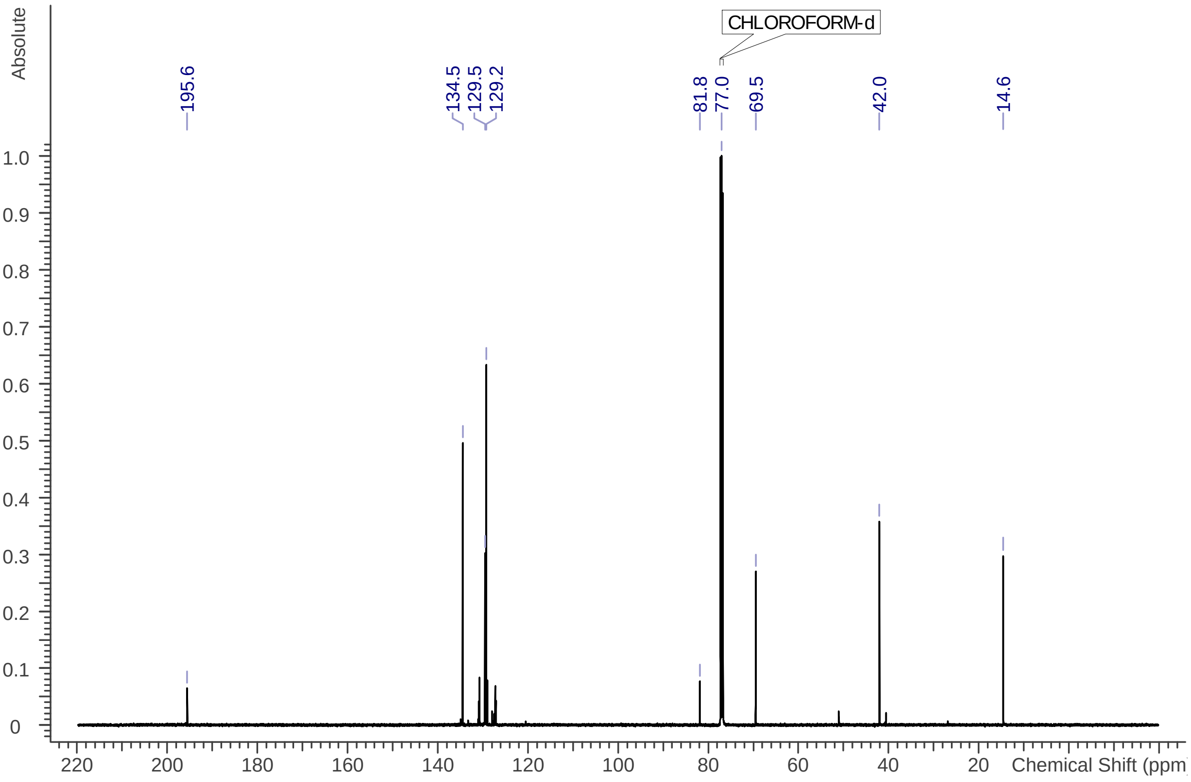

(*R*)-*N*-(3-((2-Mercaptoethyl)amino)-3-oxopropyl)-2,2,5,5-tetramethyl-1,3-dioxane-4-carboxamide (A)

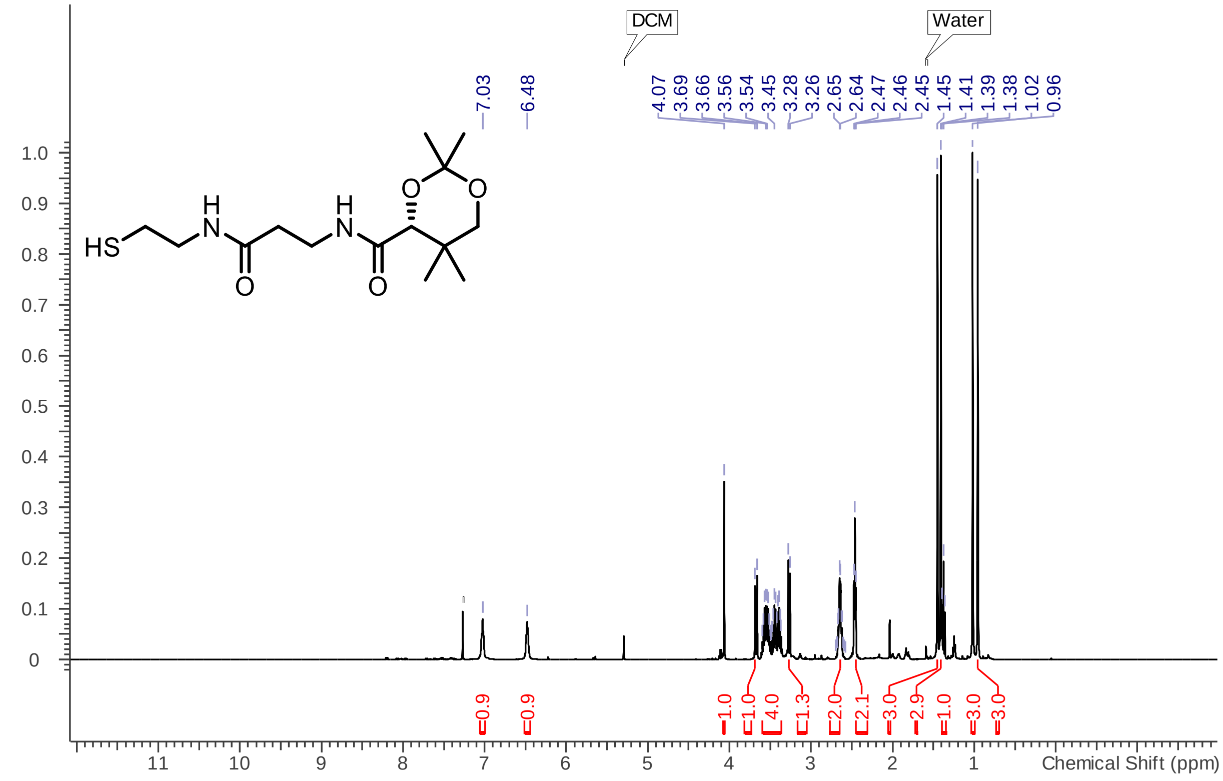

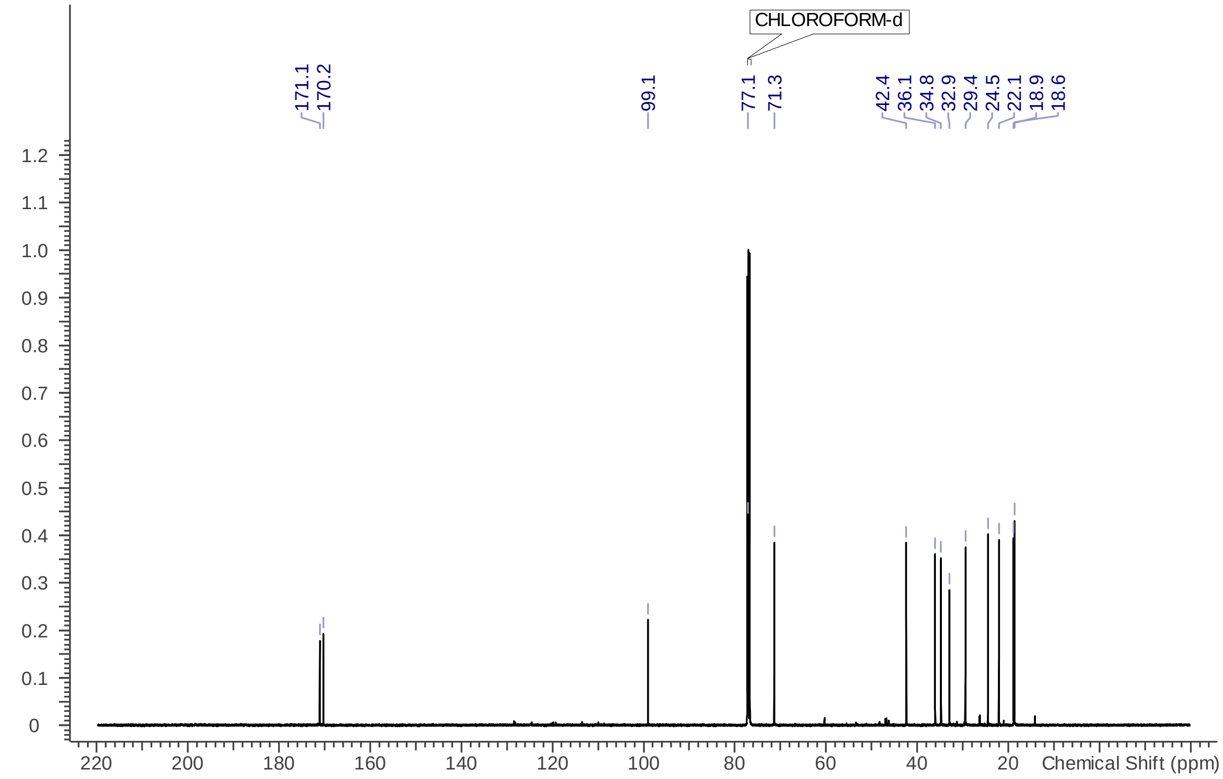

(*R*)-*S*-(2-(3-(2,2,5,5-Tetramethyl-1,3-dioxane-4-carboxamido)propanamido)ethyl) butanethioate (37a)

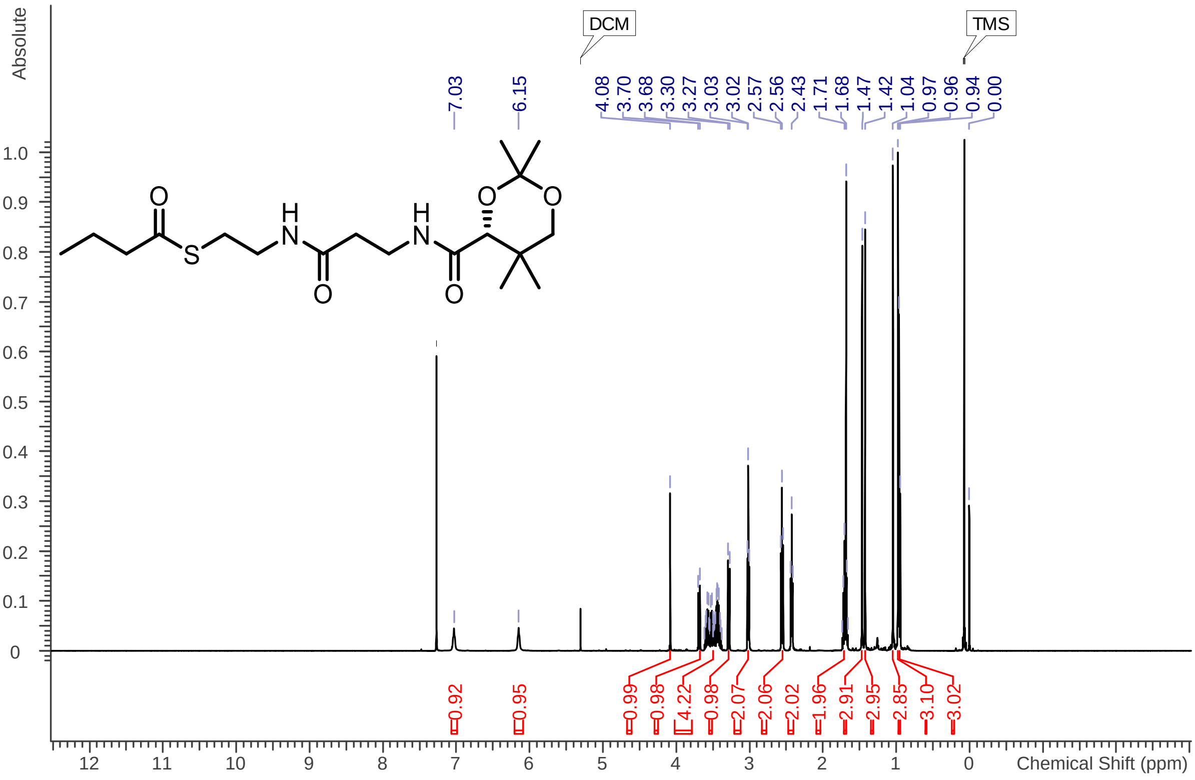

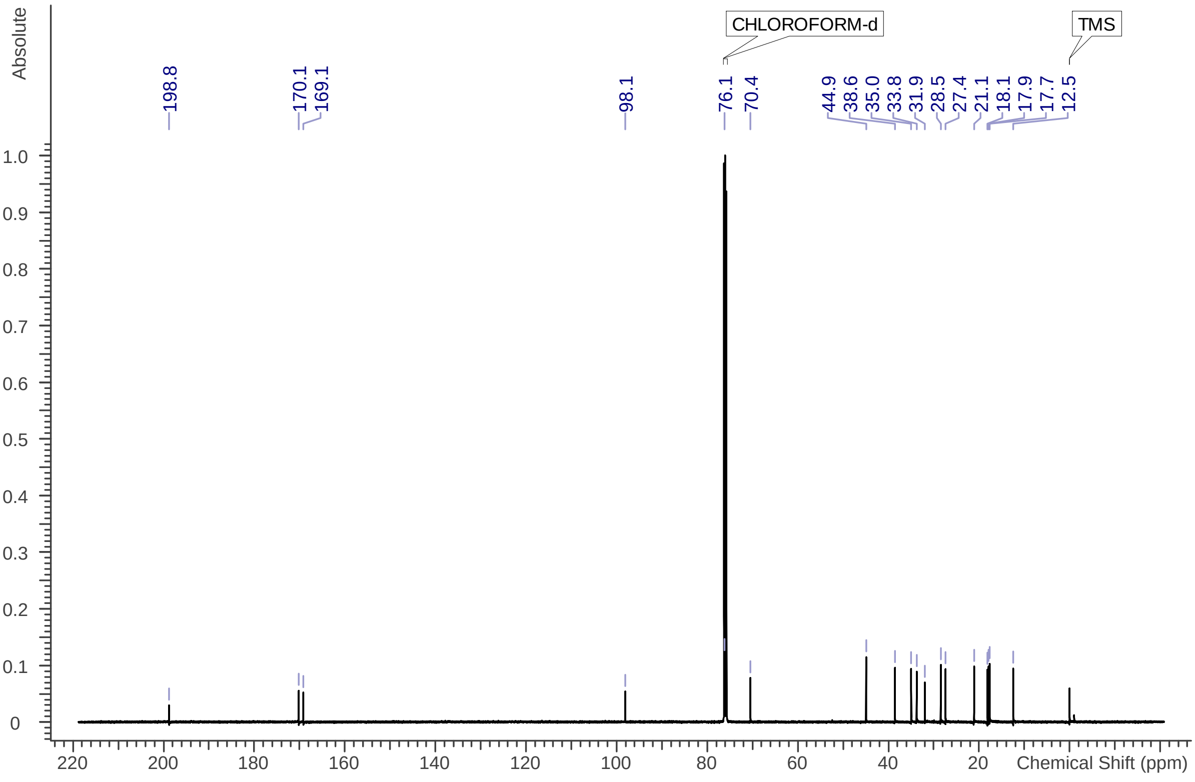

(*R*)-*S*-(2-(3-(2,4-Dihydroxy-3,3-dimethylbutanamido)propanamido)ethyl) butanethioate (37)

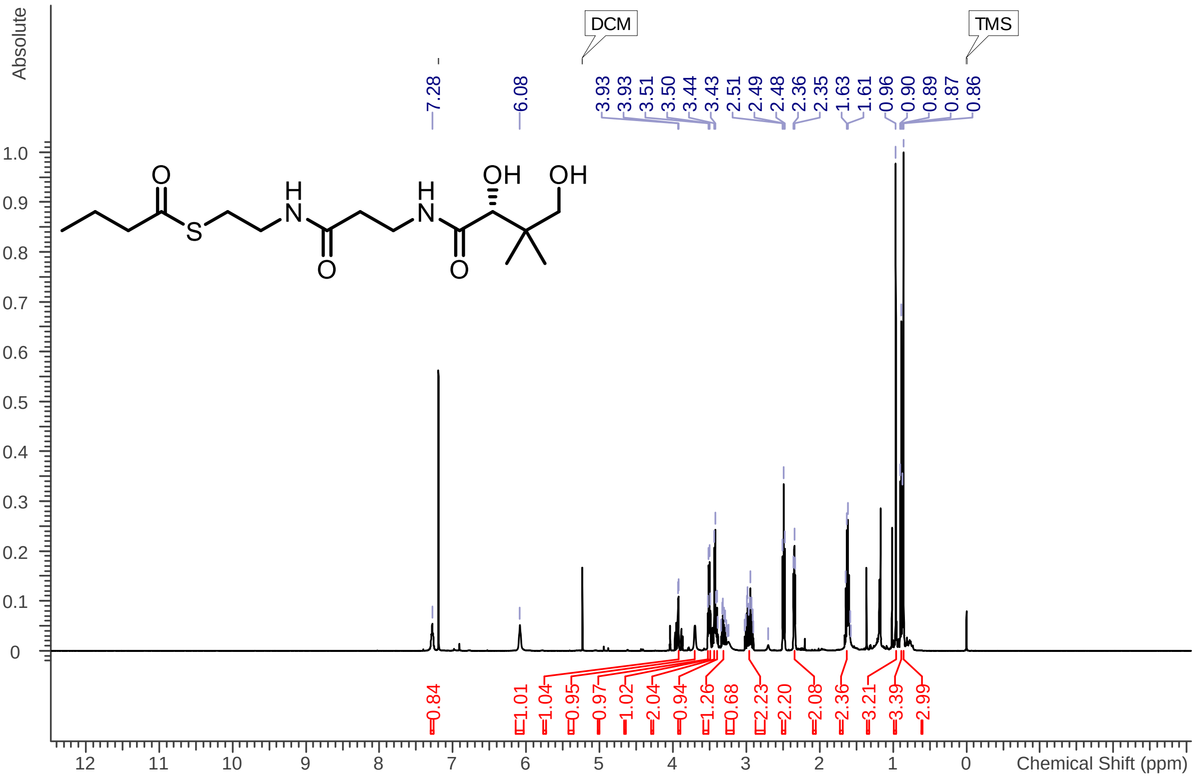

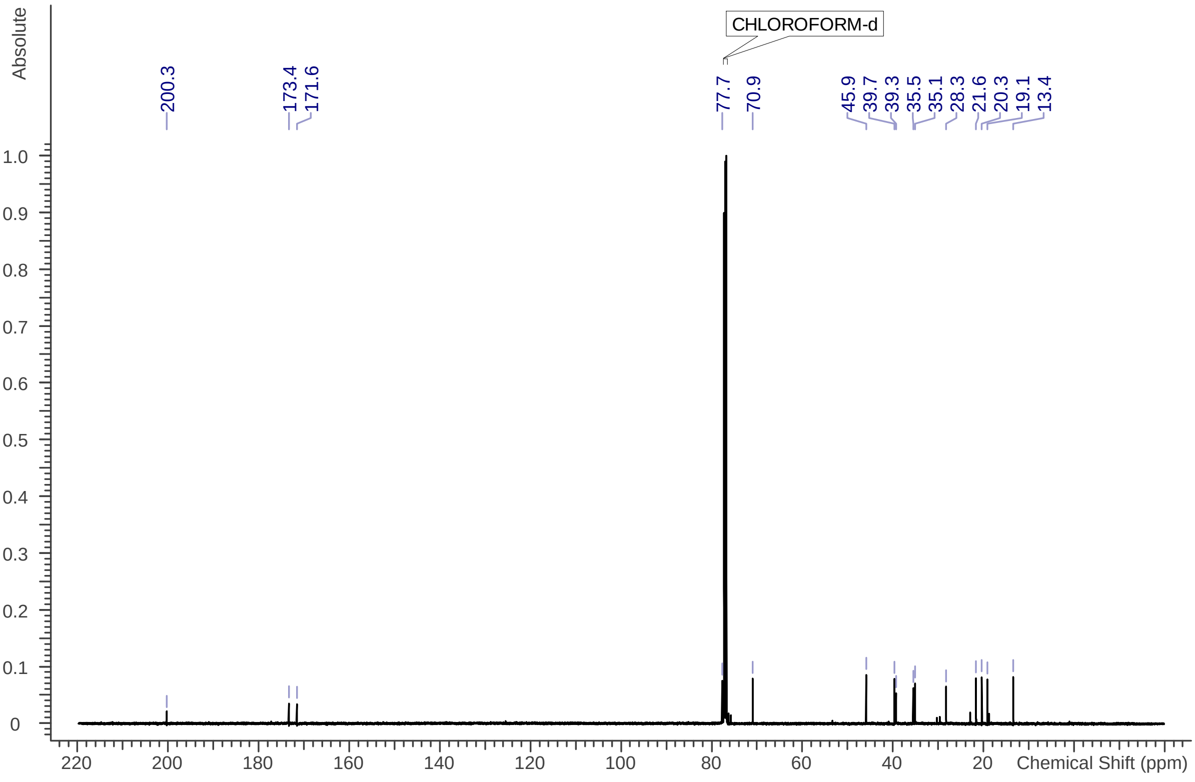

(*R*)-*S*-(2-(3-(2,2,5,5-Tetramethyl-1,3-dioxane-4-carboxamido)propanamido)ethyl) 4,4,4-trifluorobutanethioate (38a)

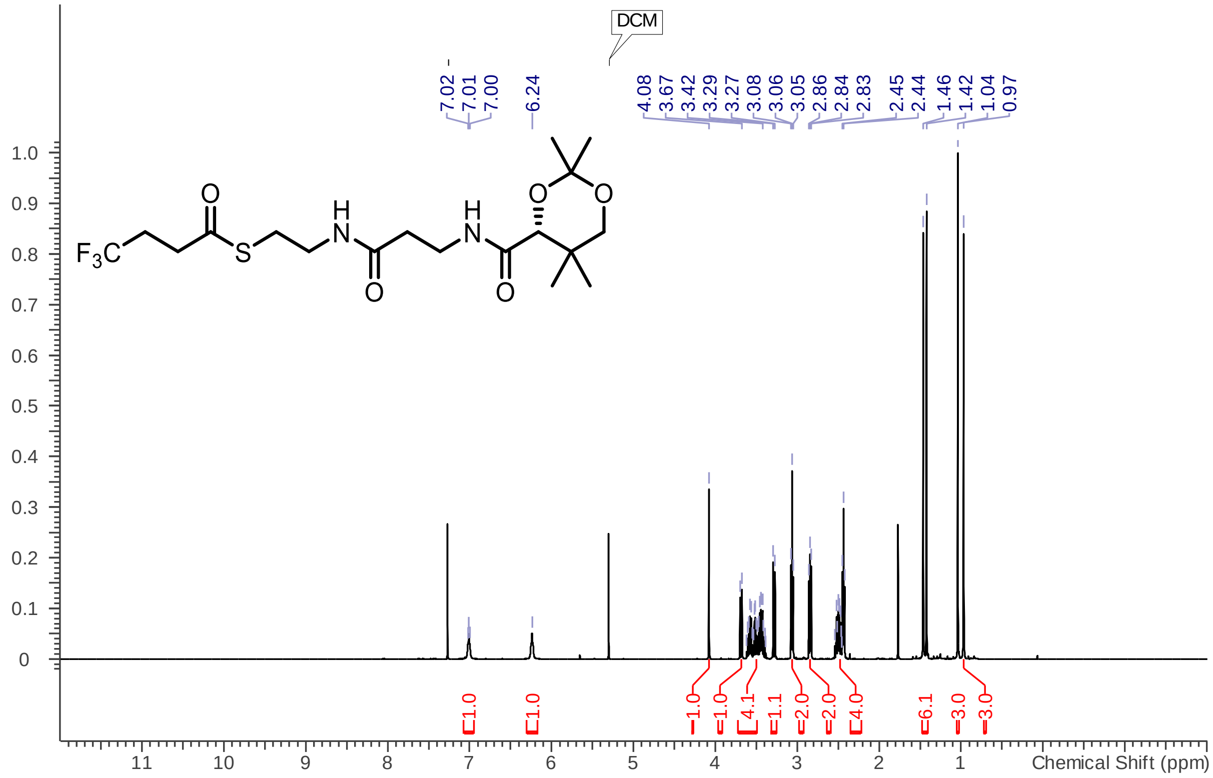

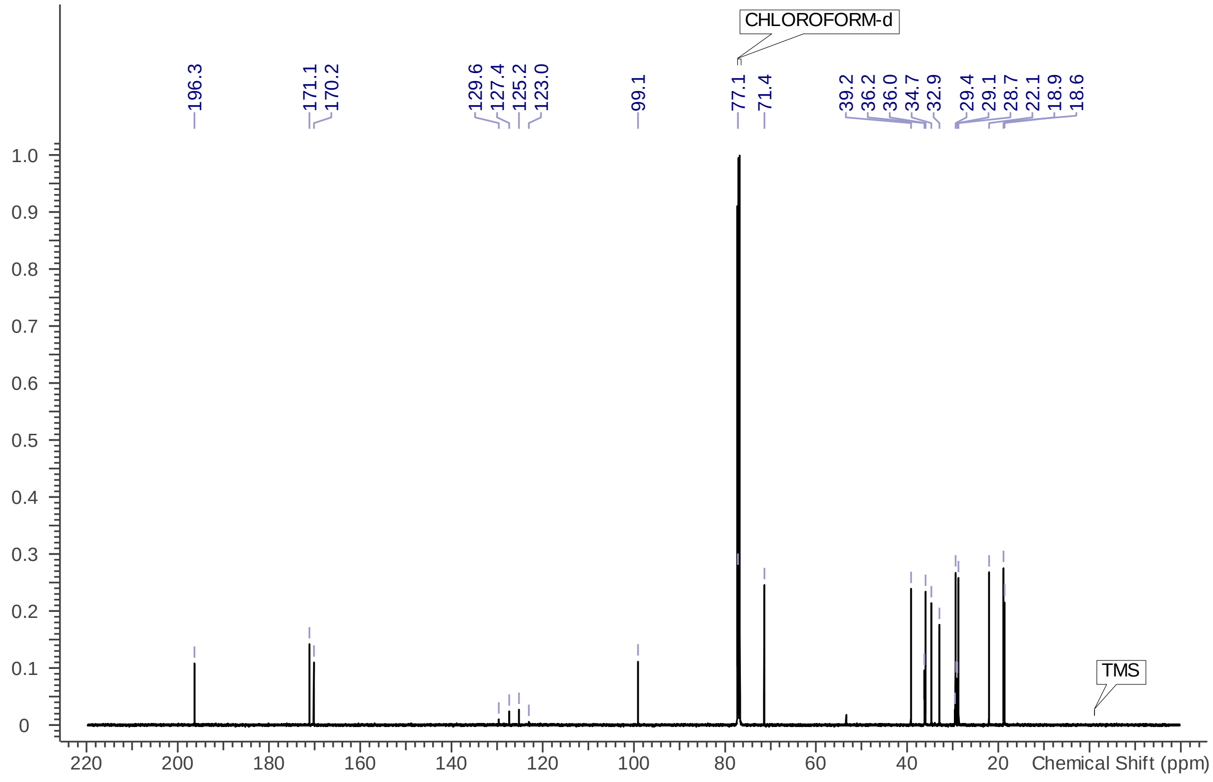

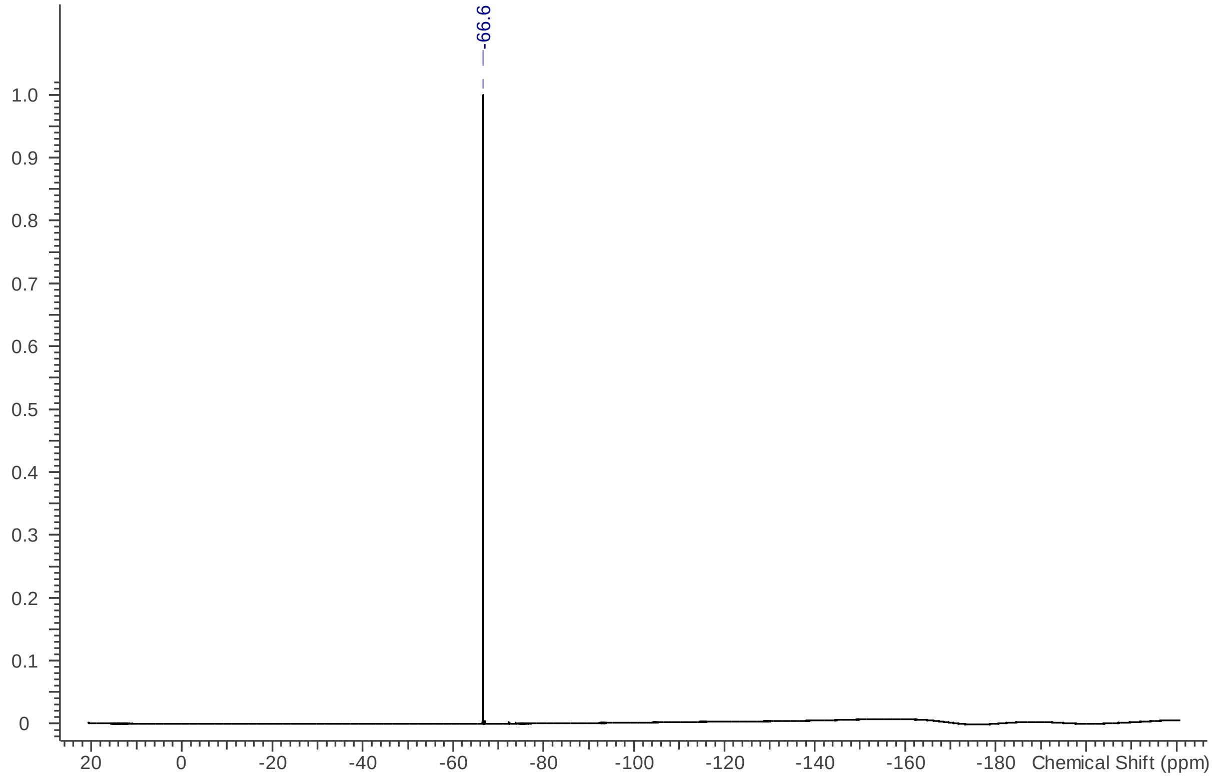
-66.6
1.0
0.9
0.8
0.7
0.6
0.5
0.4
0.3
0.2
0.1
0
20
0
-20
-40
-60
-80
-100
-120
-140
-160
-180
Chemical Shift (ppm)

(*R*)-*S*-(2-(3-(2,4-Dihydroxy-3,3-dimethylbutanamido)propanamido)ethyl) 4,4,4-trifluoro-butanethioate (38)

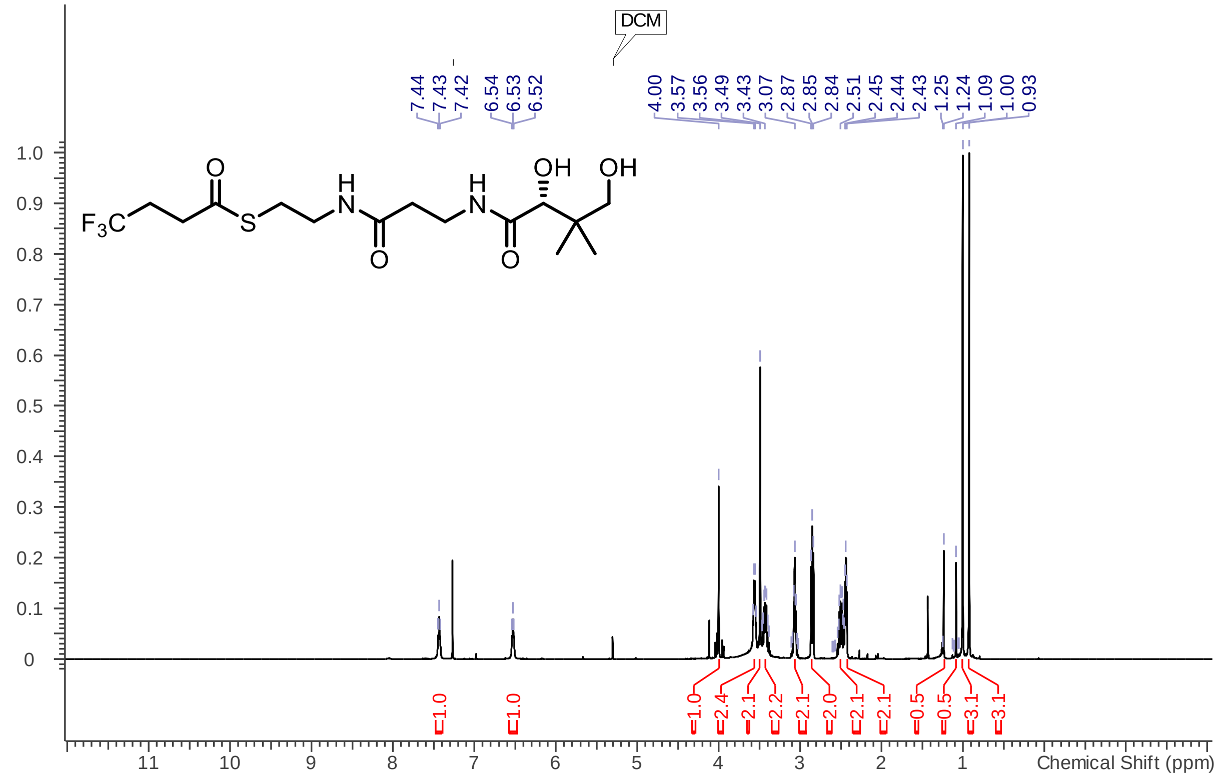

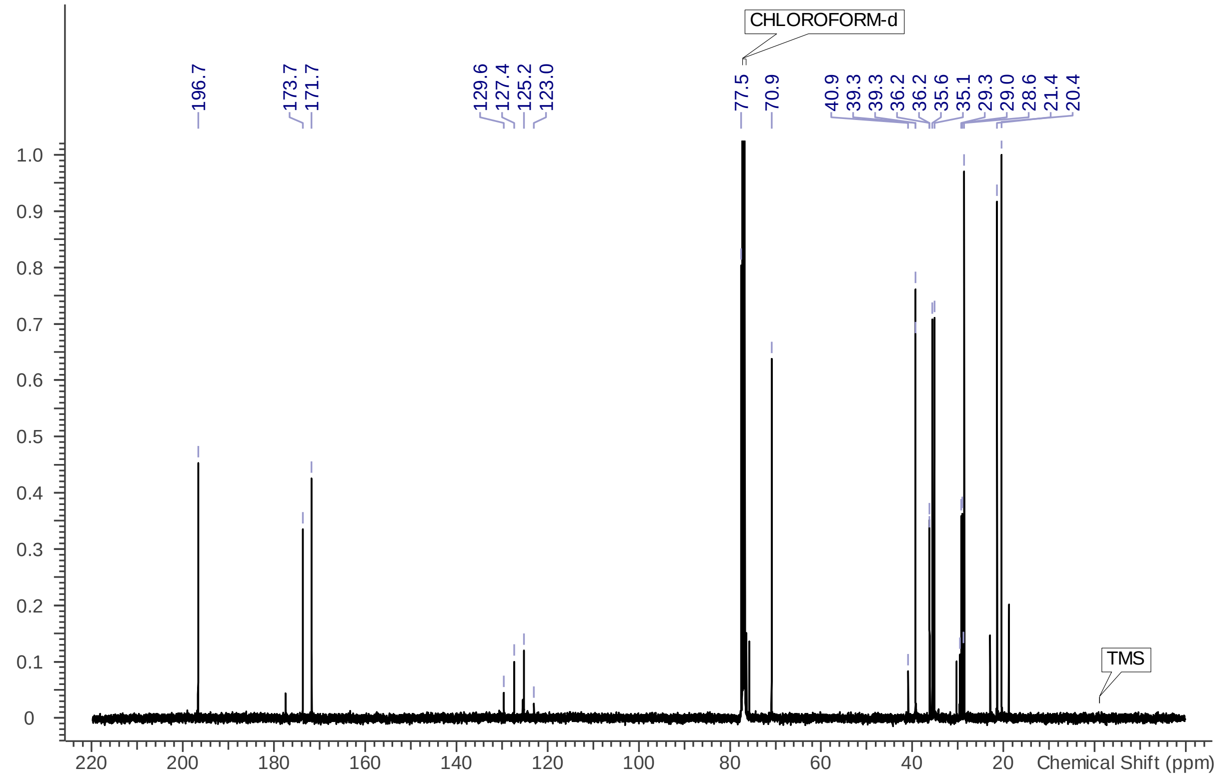

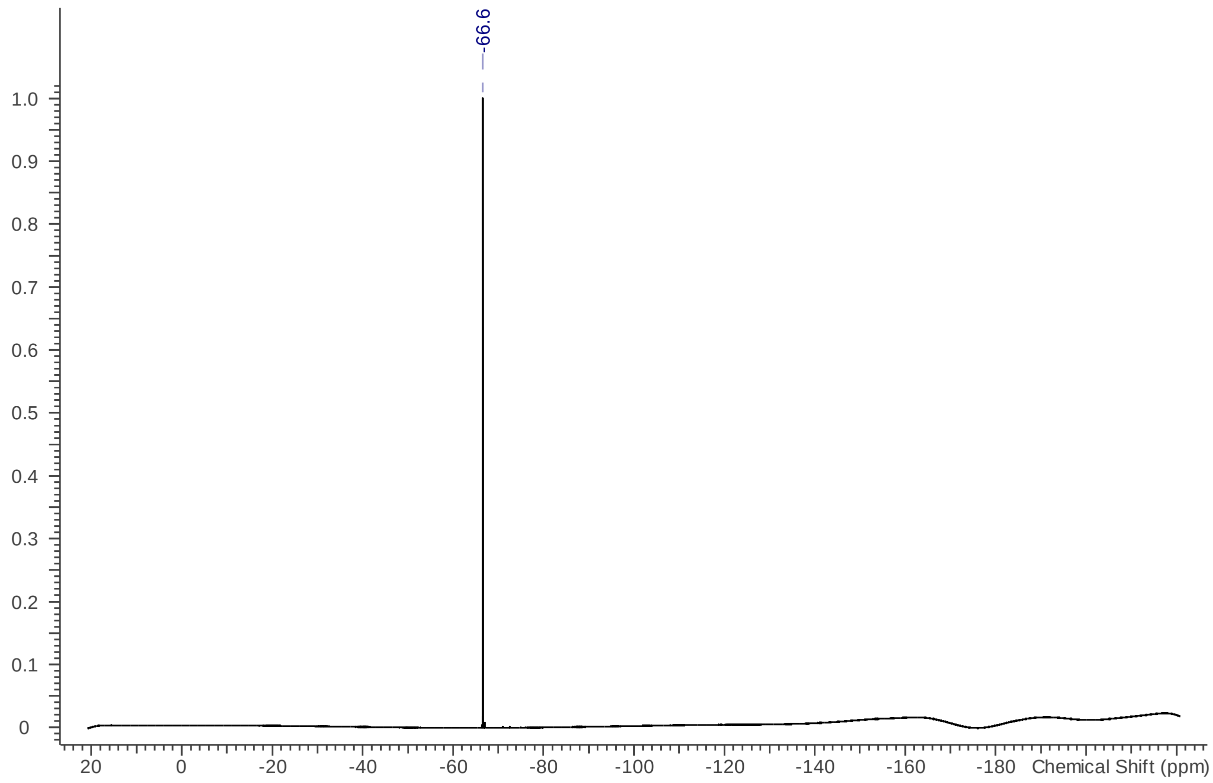

-66.6
1.0
0.9
0.8
0.7
0.6
0.5
0.4
0.3
0.2
0.1
0
20
0
-20
-40
-60
-80
-100
-120
-140
-160
-180
Chemical Shift (ppm)

(*R*)-*S*-(2-(3-(2,2,5,5-Tetramethyl-1,3-dioxane-4-carboxamido)propanamido)ethyl) pent-4-ynethioate (39a)

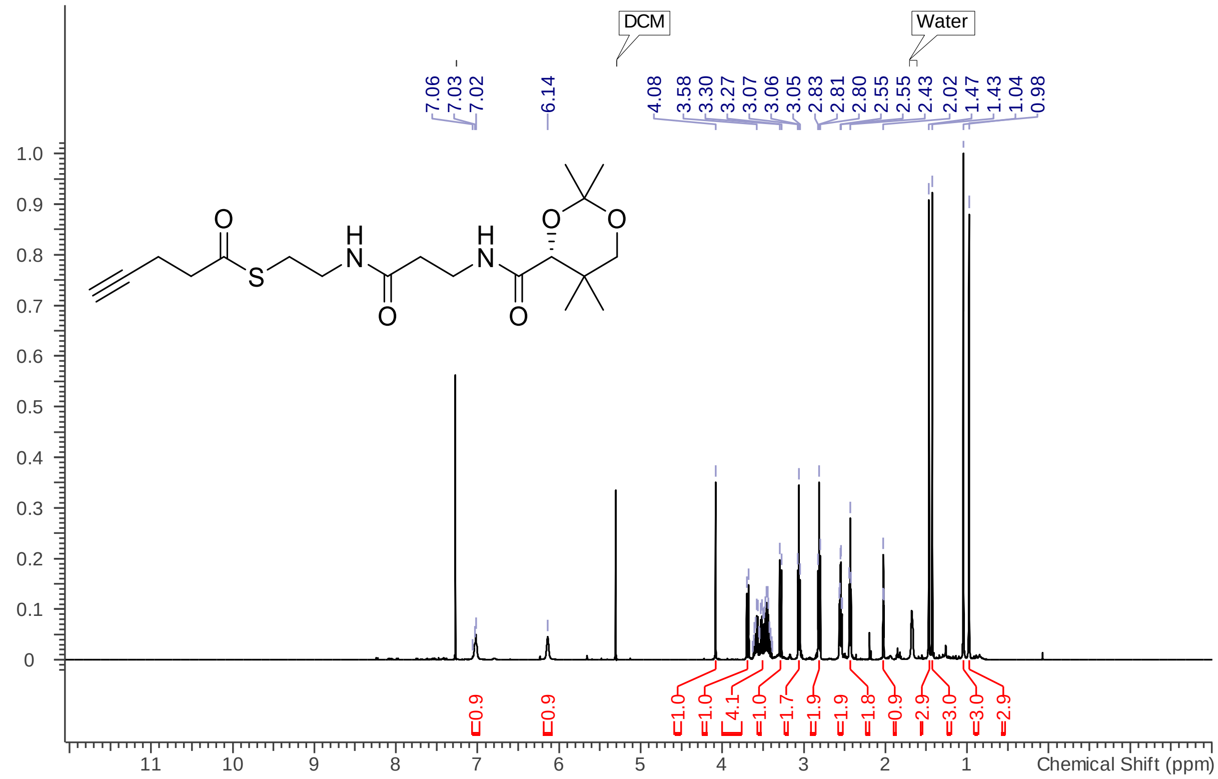

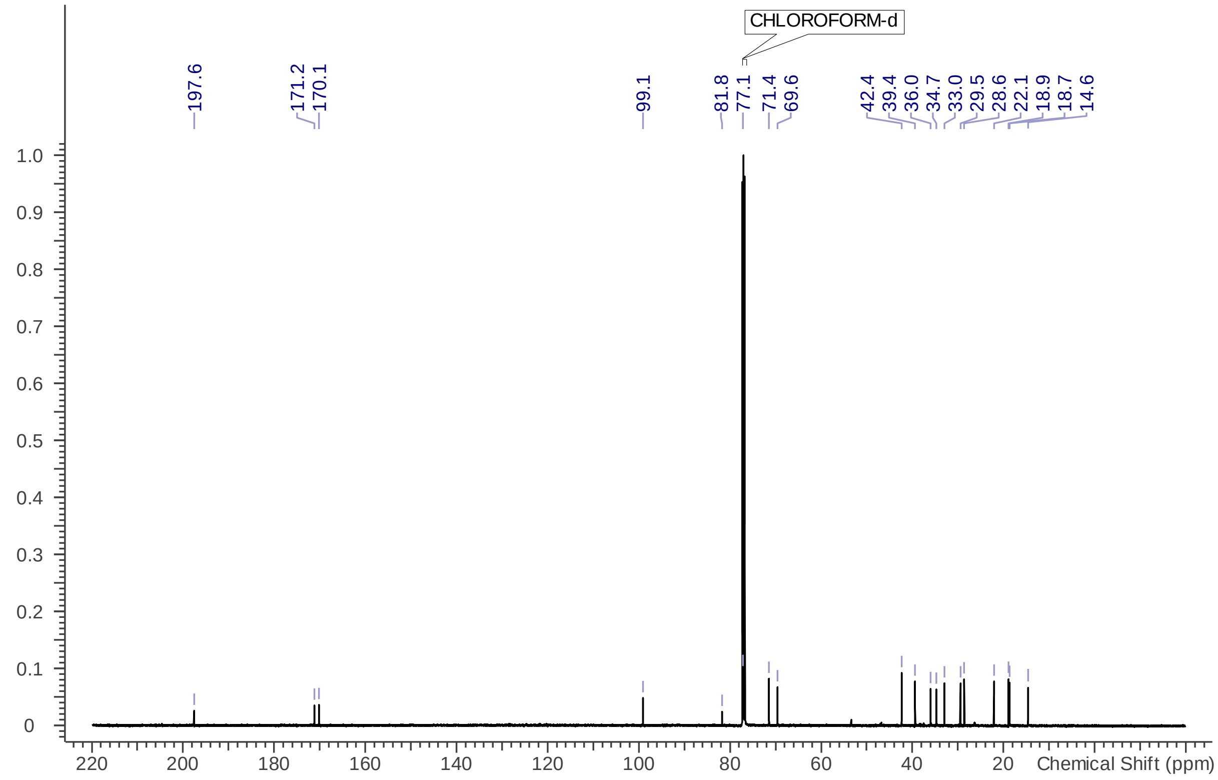

(*R*)-*S*-(2-(3-(2,4-Dihydroxy-3,3-dimethylbutanamido)propanamido)ethyl) pent-4-yne-thioate (39)

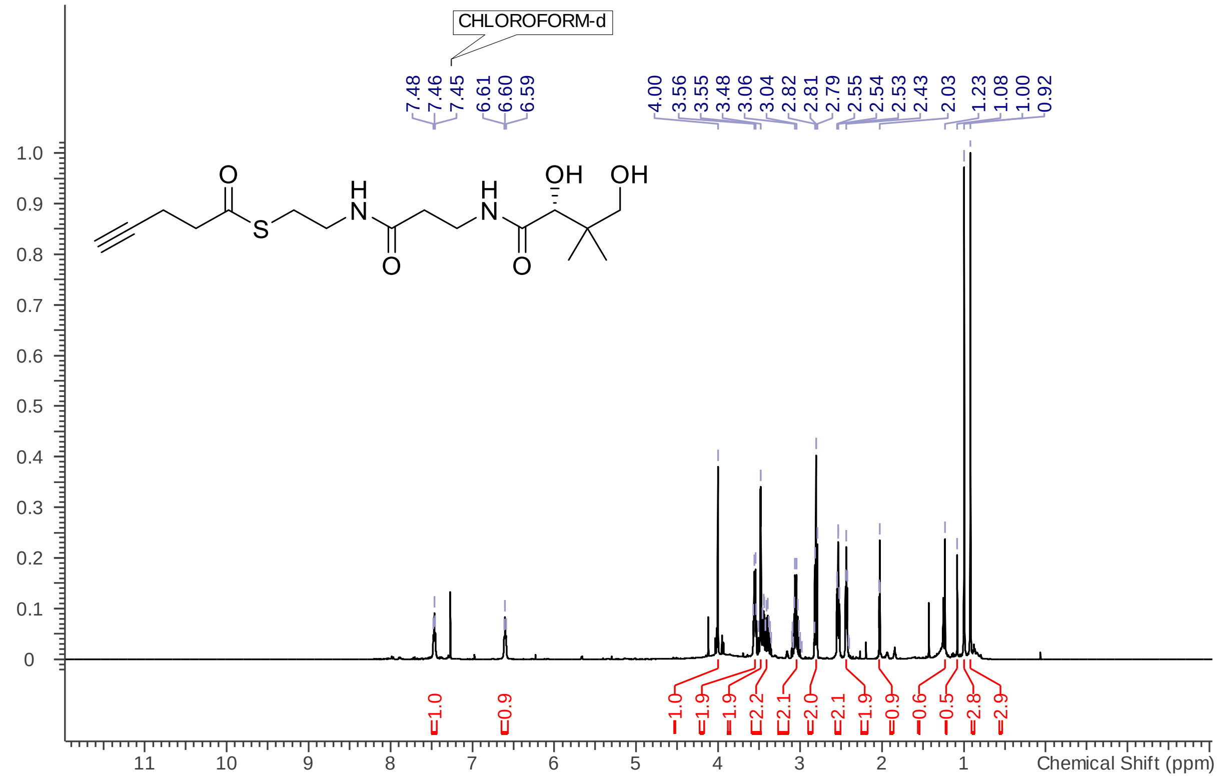

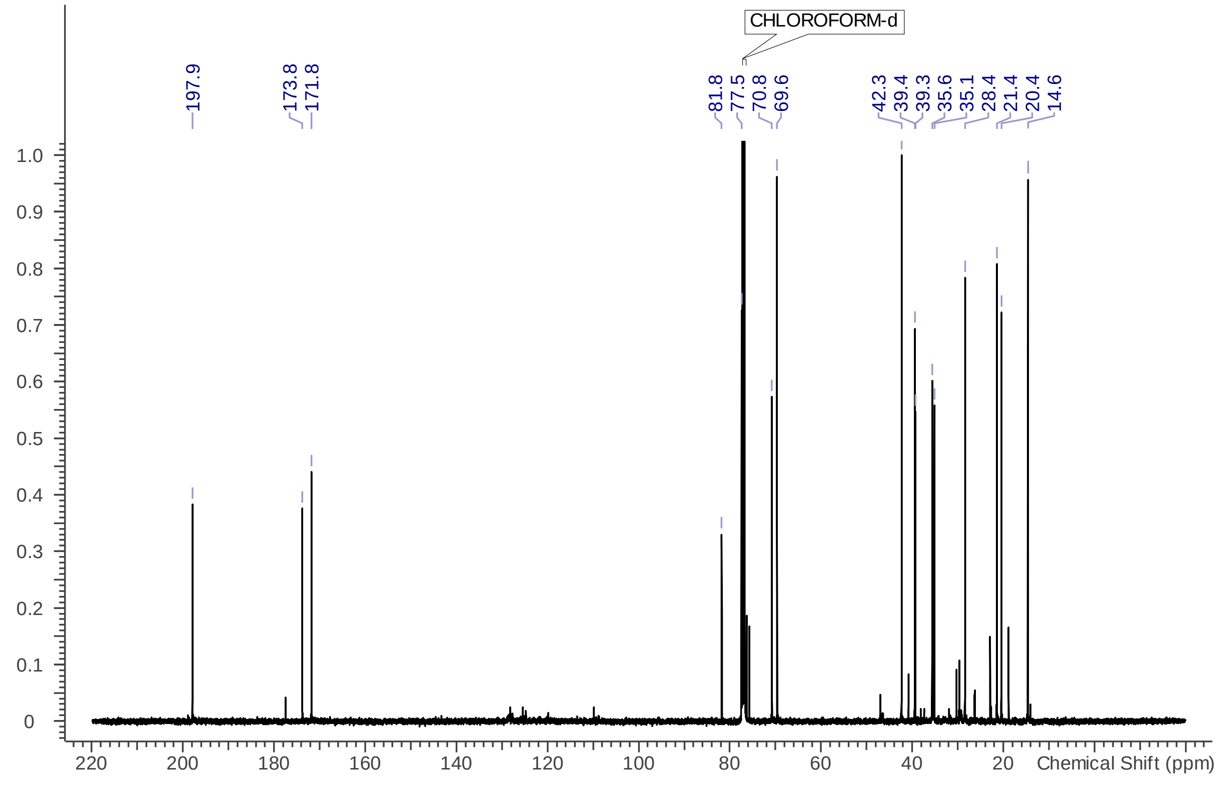

Ethyl (*E*)-3-methylhex-2-enoate (40a)

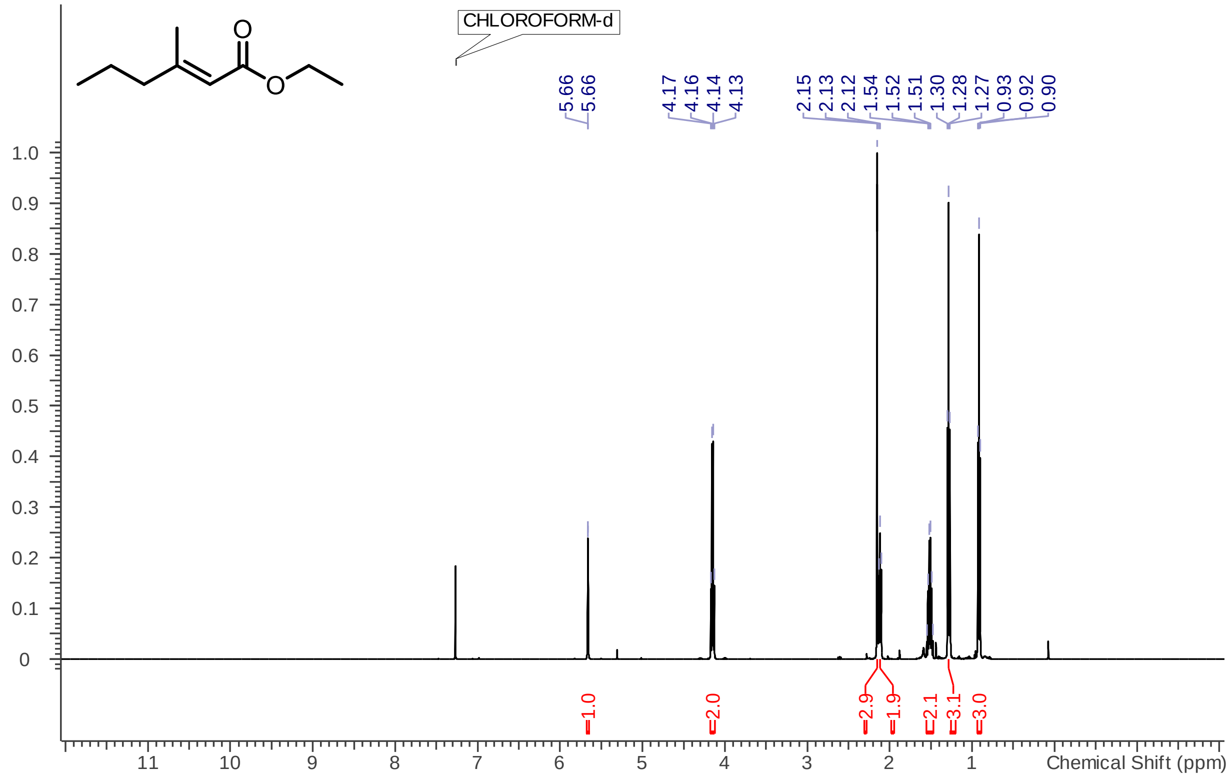

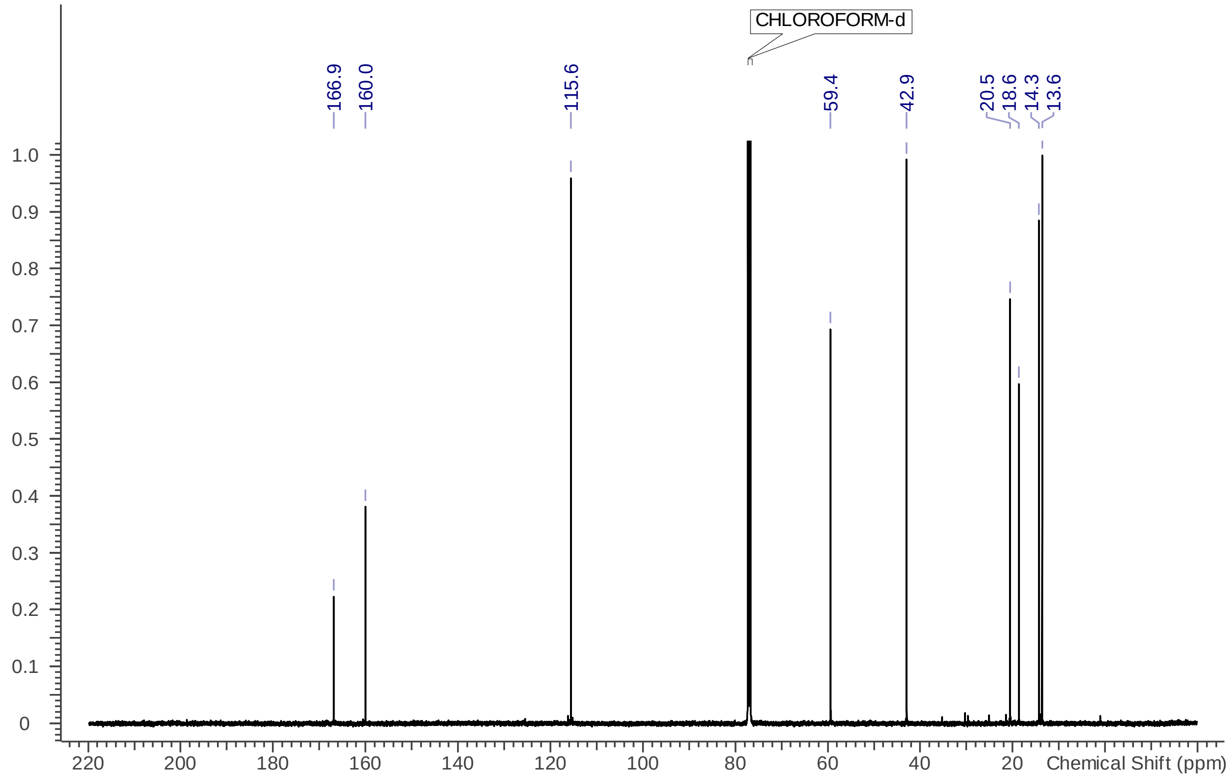

Ethyl (2*E*,4*E*)-2,5-dimethylocta-2,4-dienoate (40b)

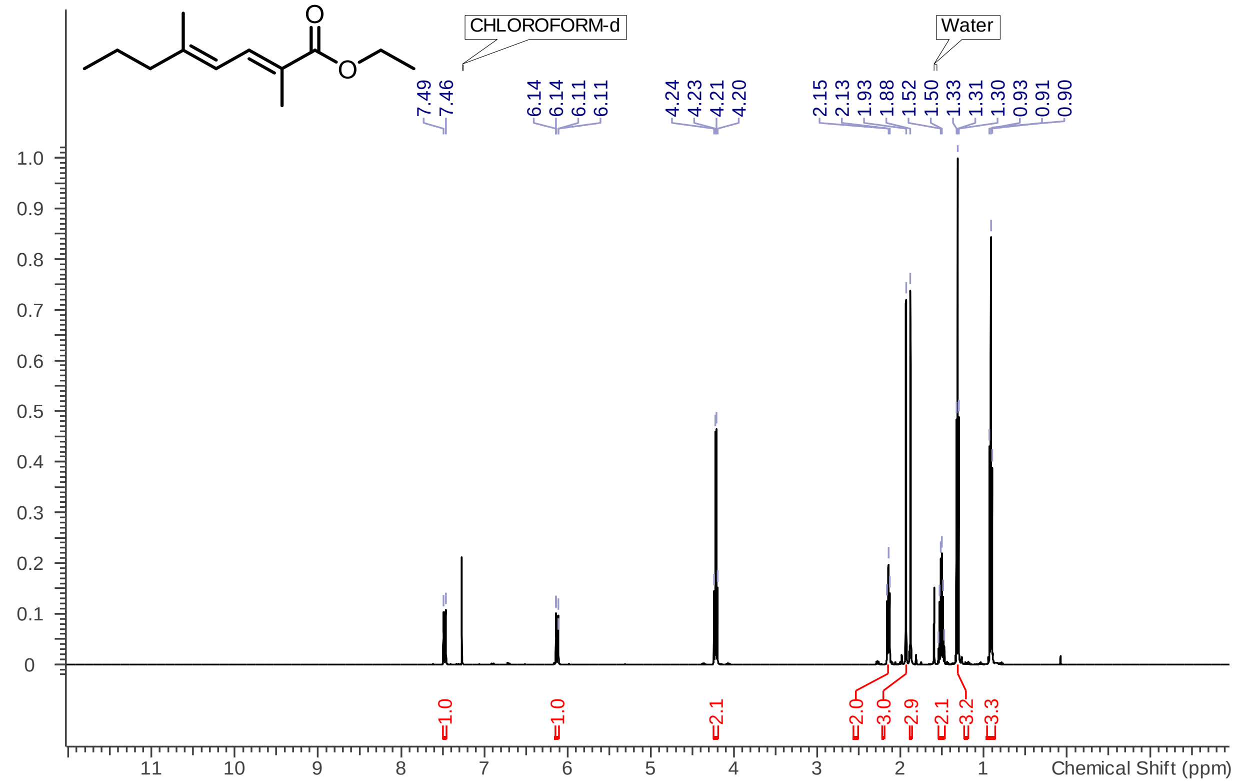

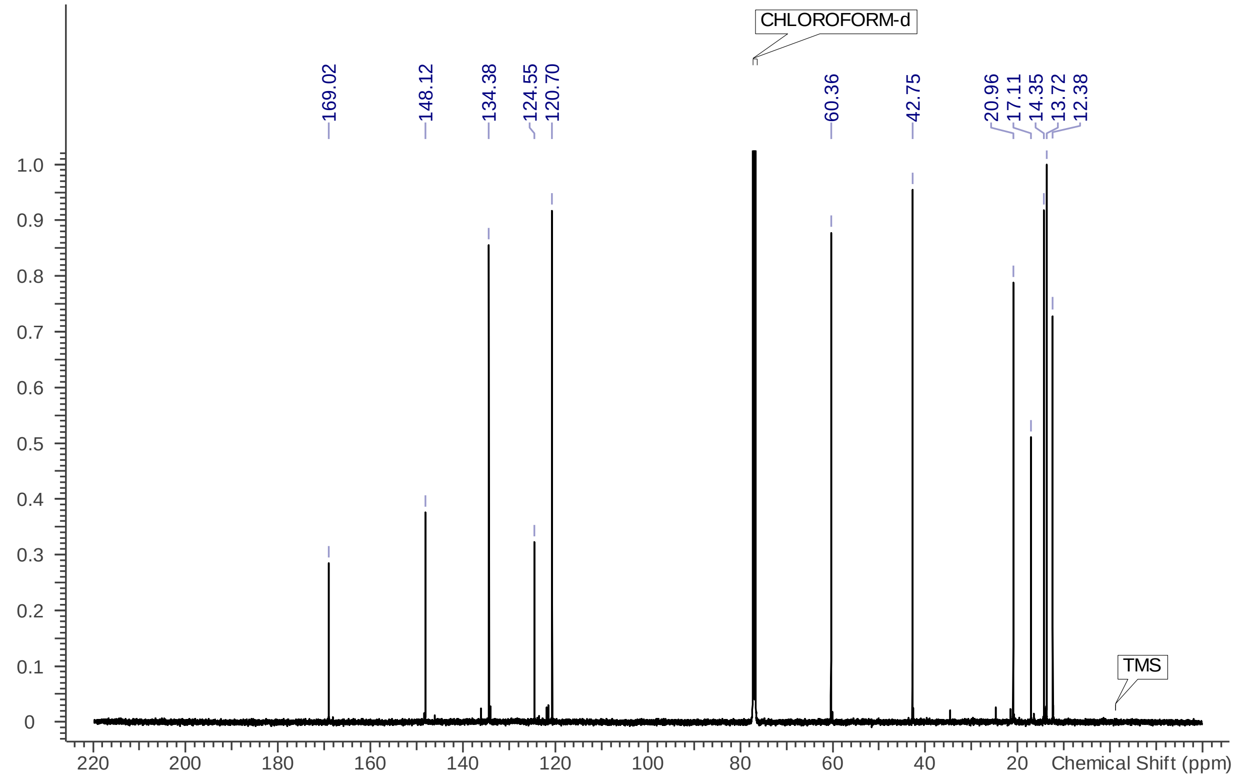

(2*E*,4*E*)-2,5-Dimethylocta-2,4-dienoic acid (40c)

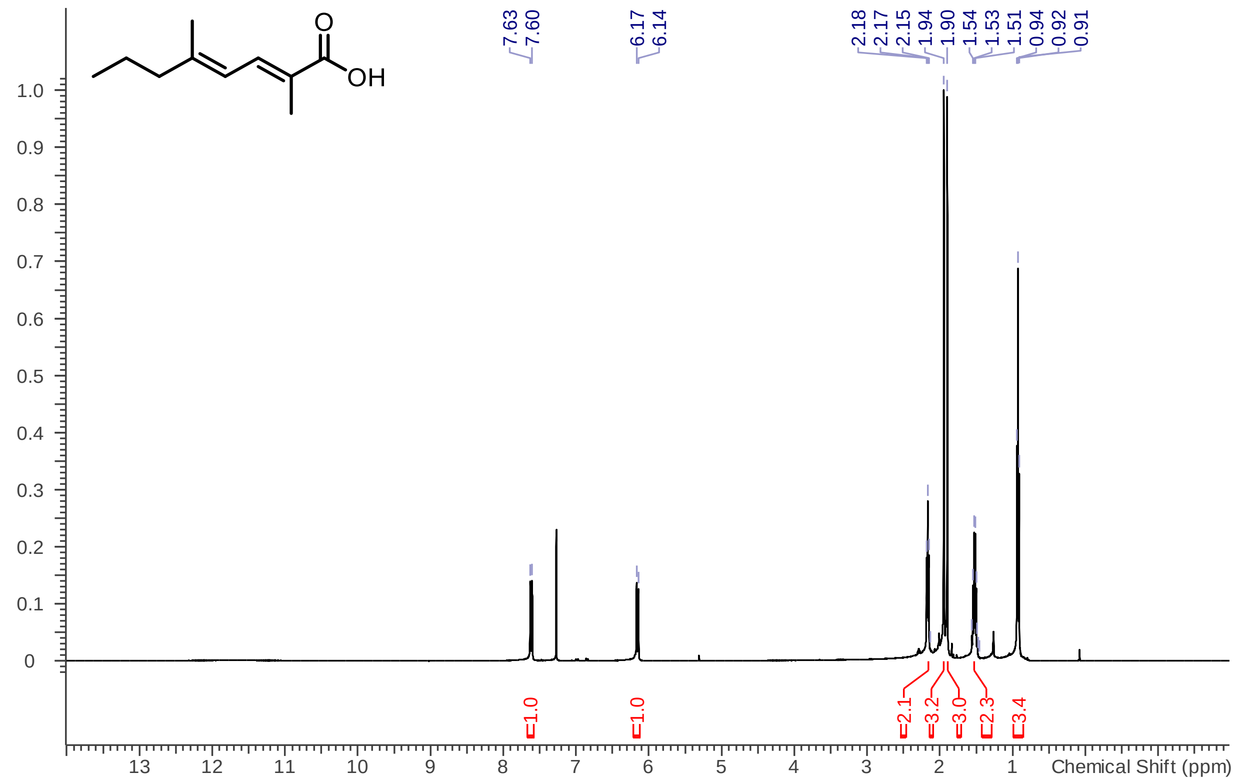

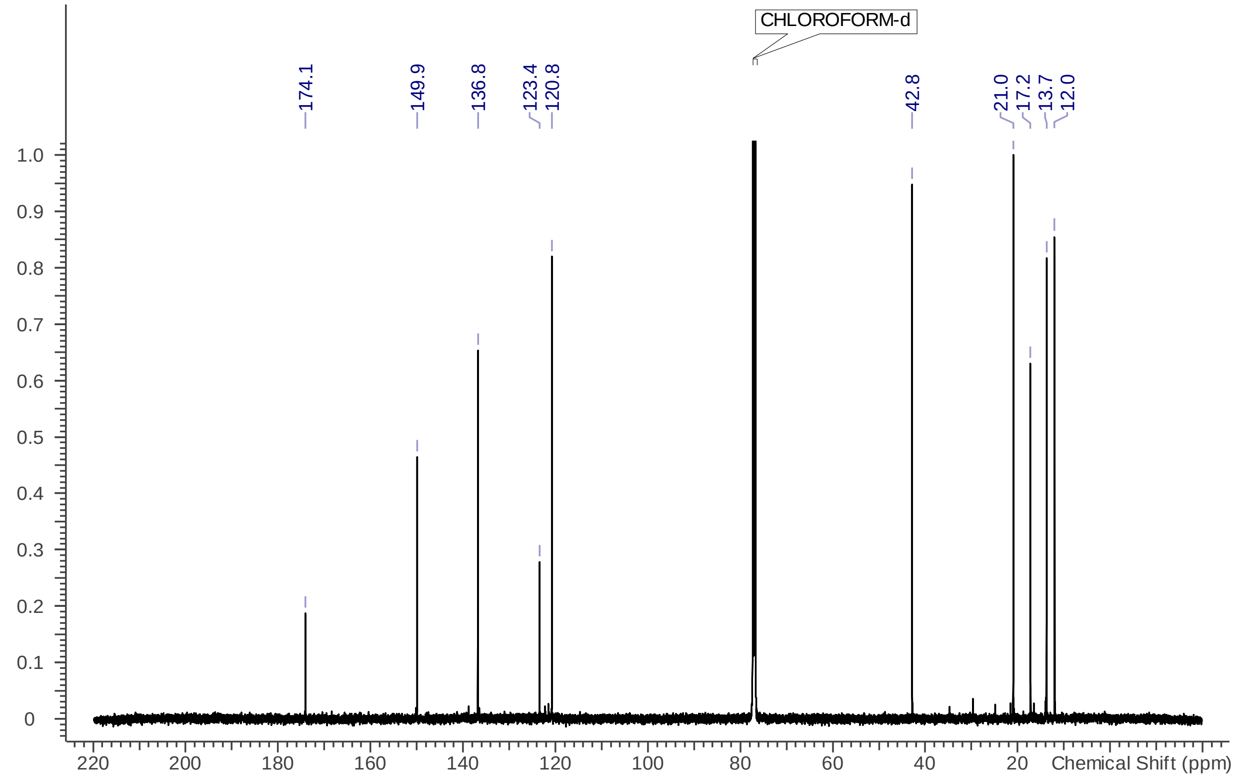

S-(2-Acetamidoethyl) (2E,4E)-2,5-dimethylocta-2,4-dienethioate (40)

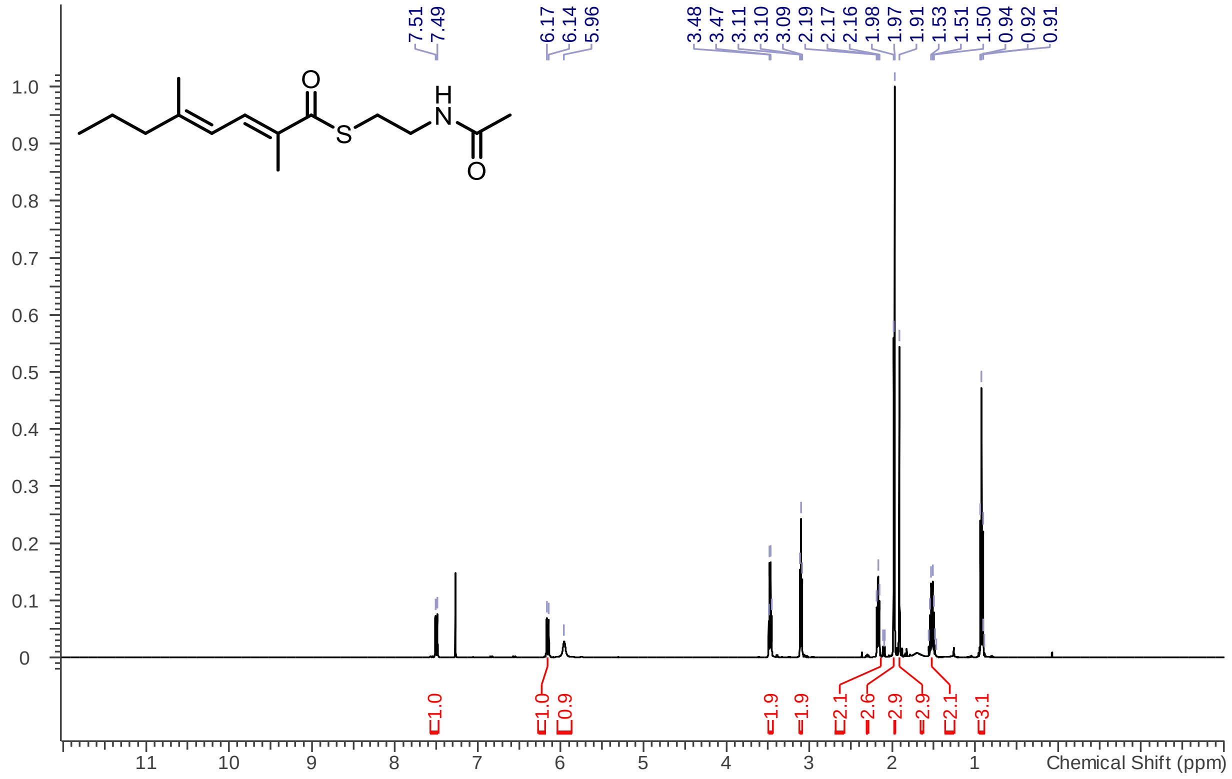

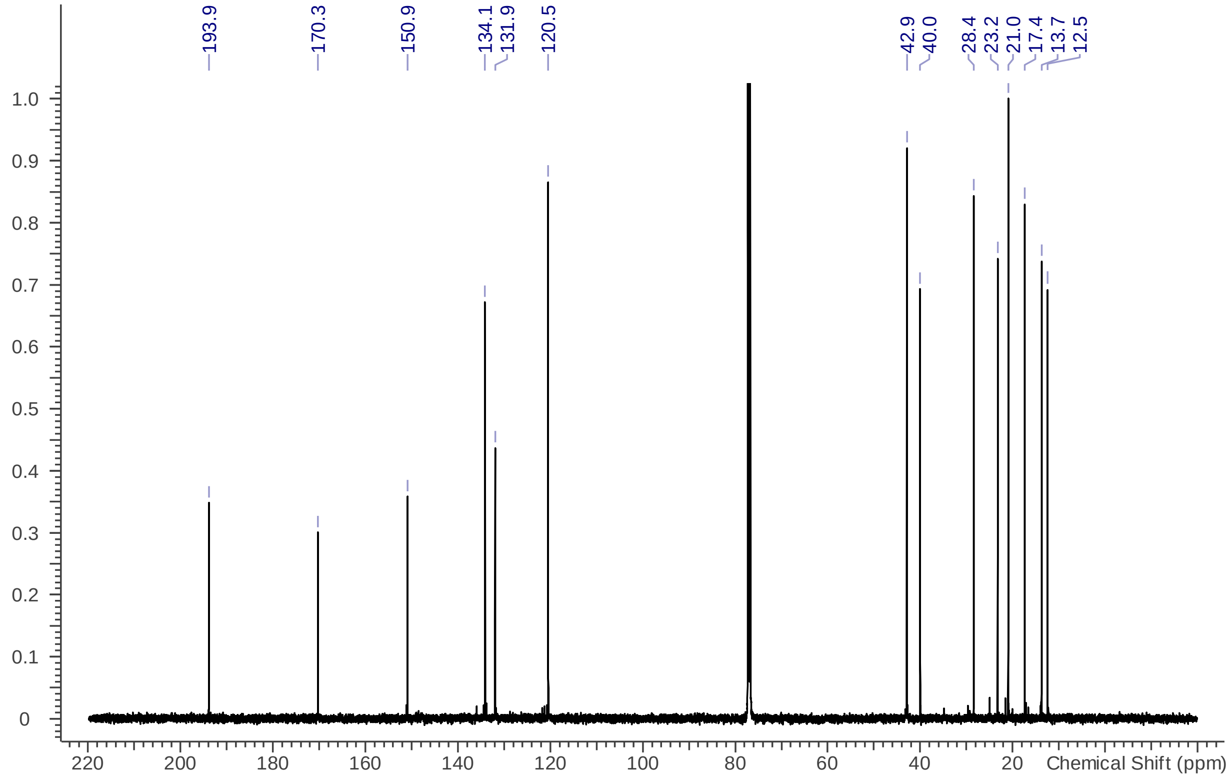

Ethyl (*E*)-3-methylhept-2-enoate (41a)

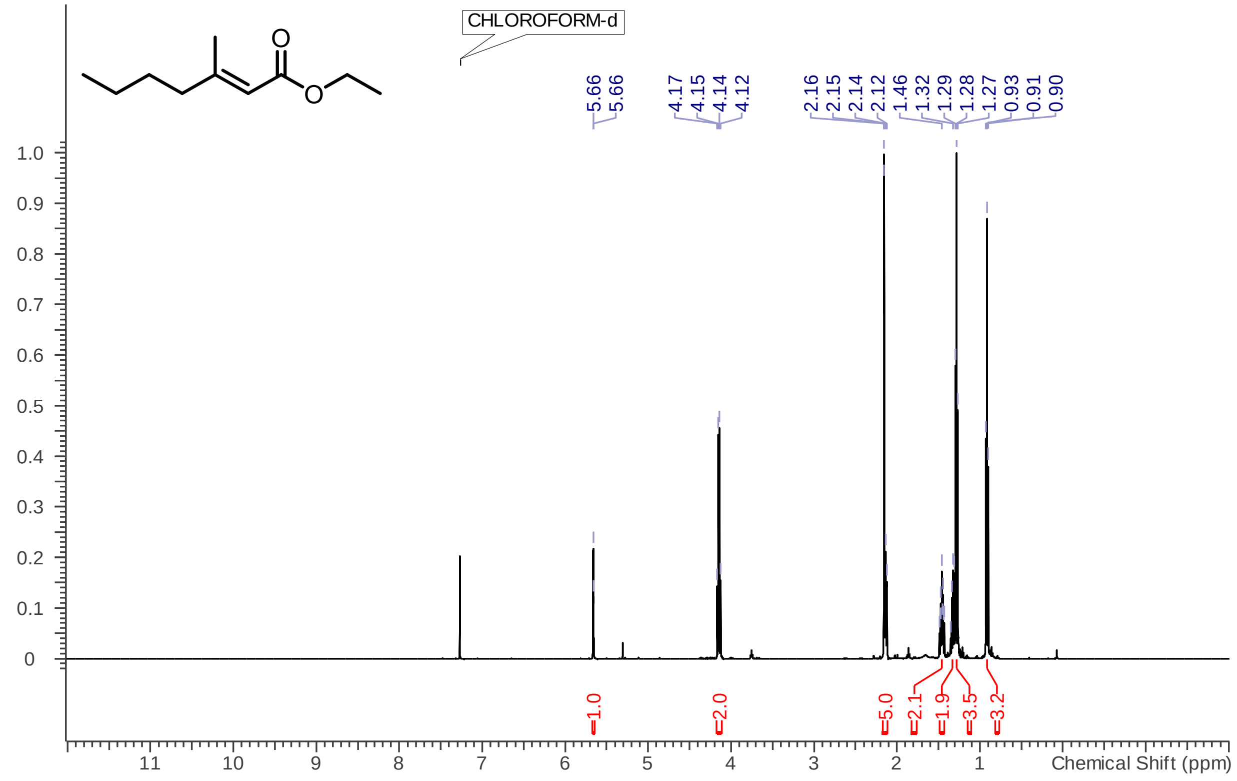

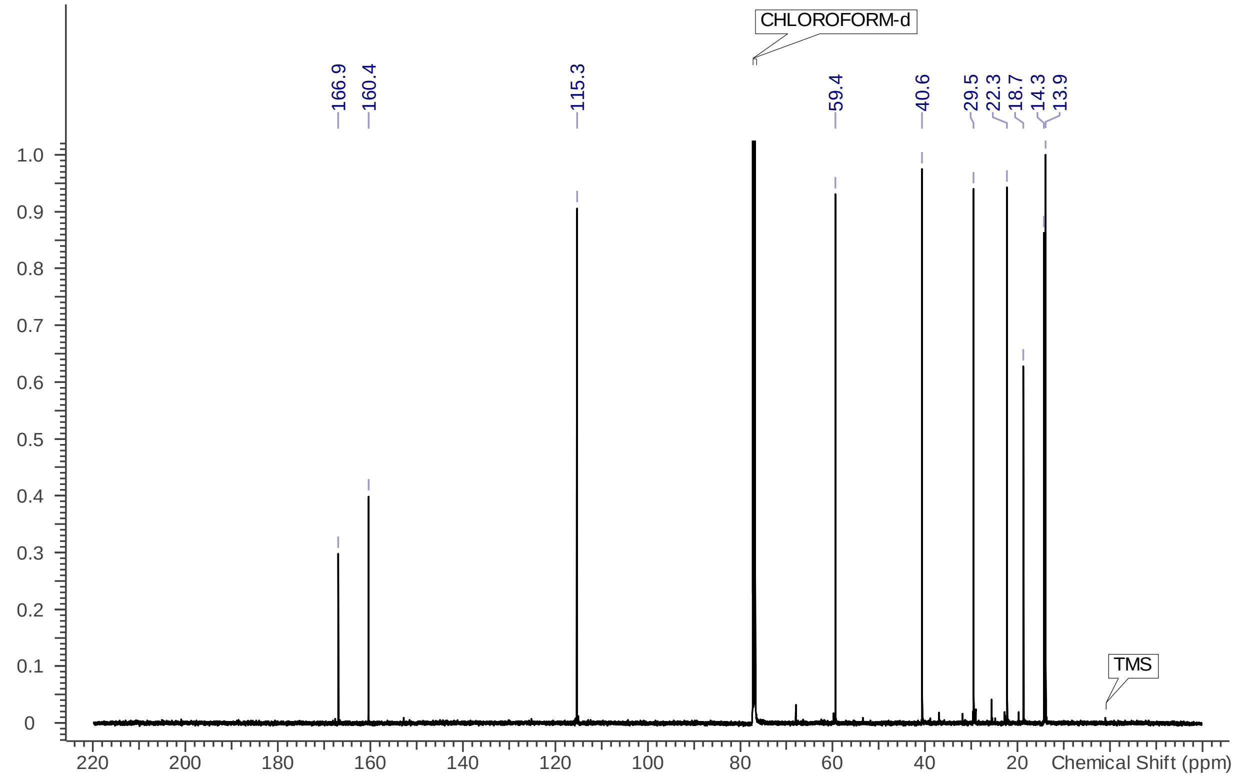

Ethyl (2*E*,4*E*)-2,5-dimethylnona-2,4-dienoate (41b)

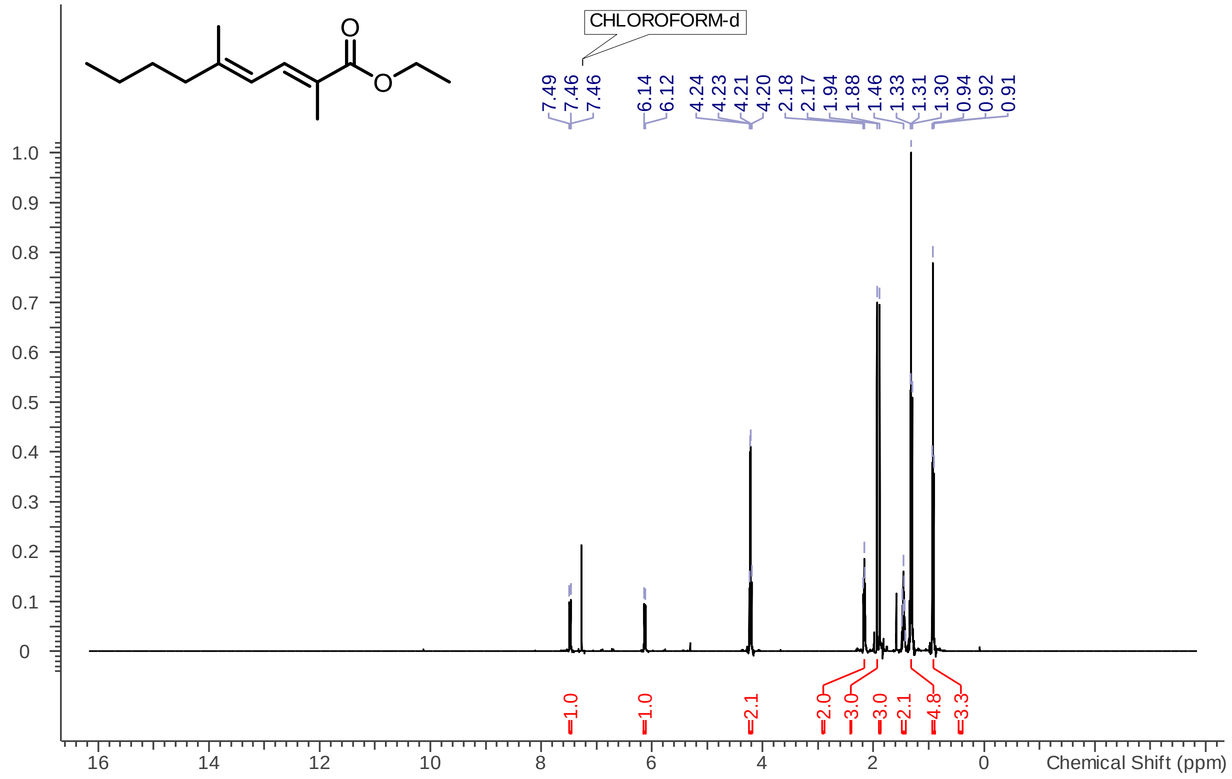

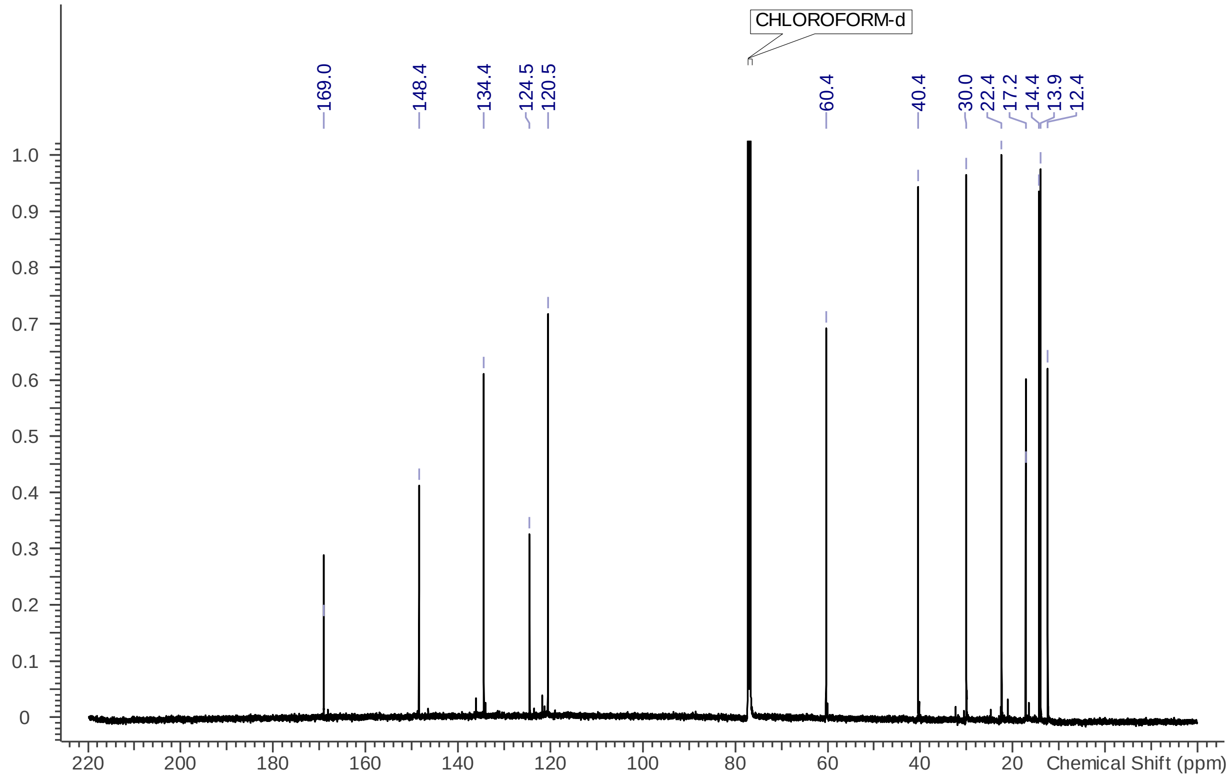

(2*E*,4*E*)-2,5-Dimethylnona-2,4-dienoic acid (41c)

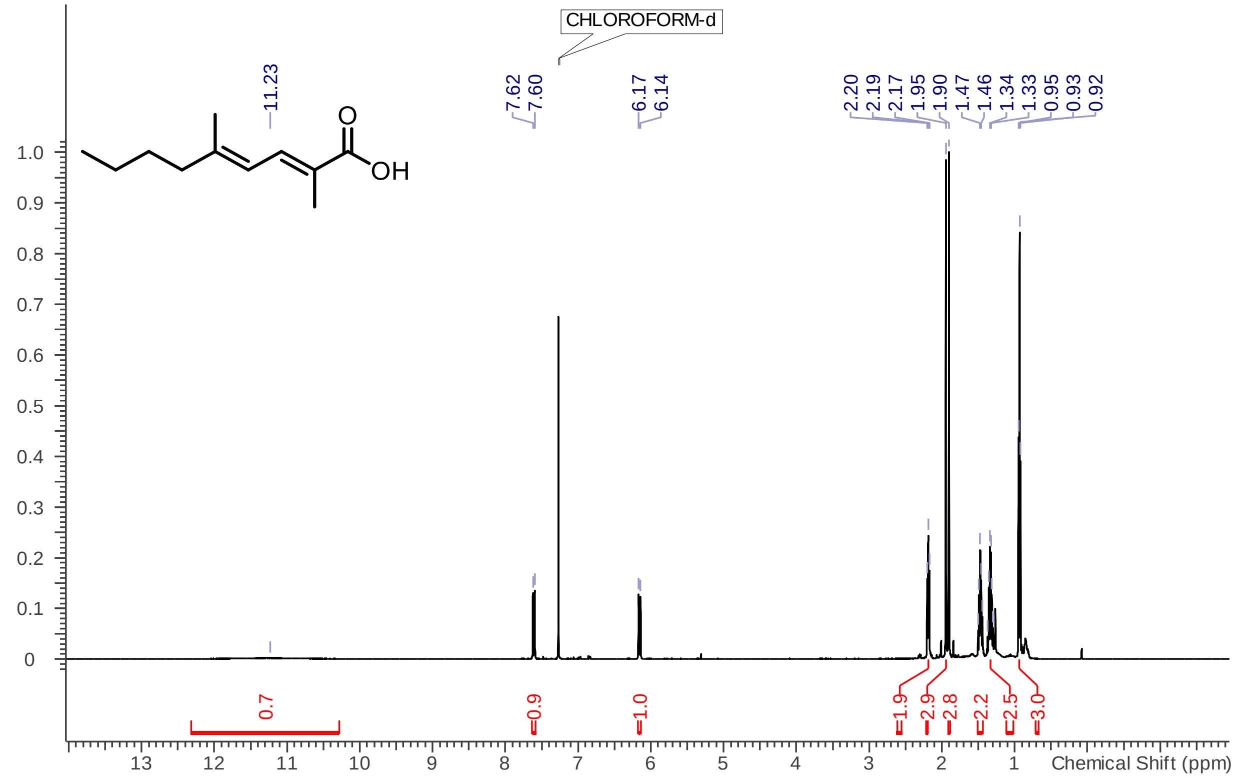

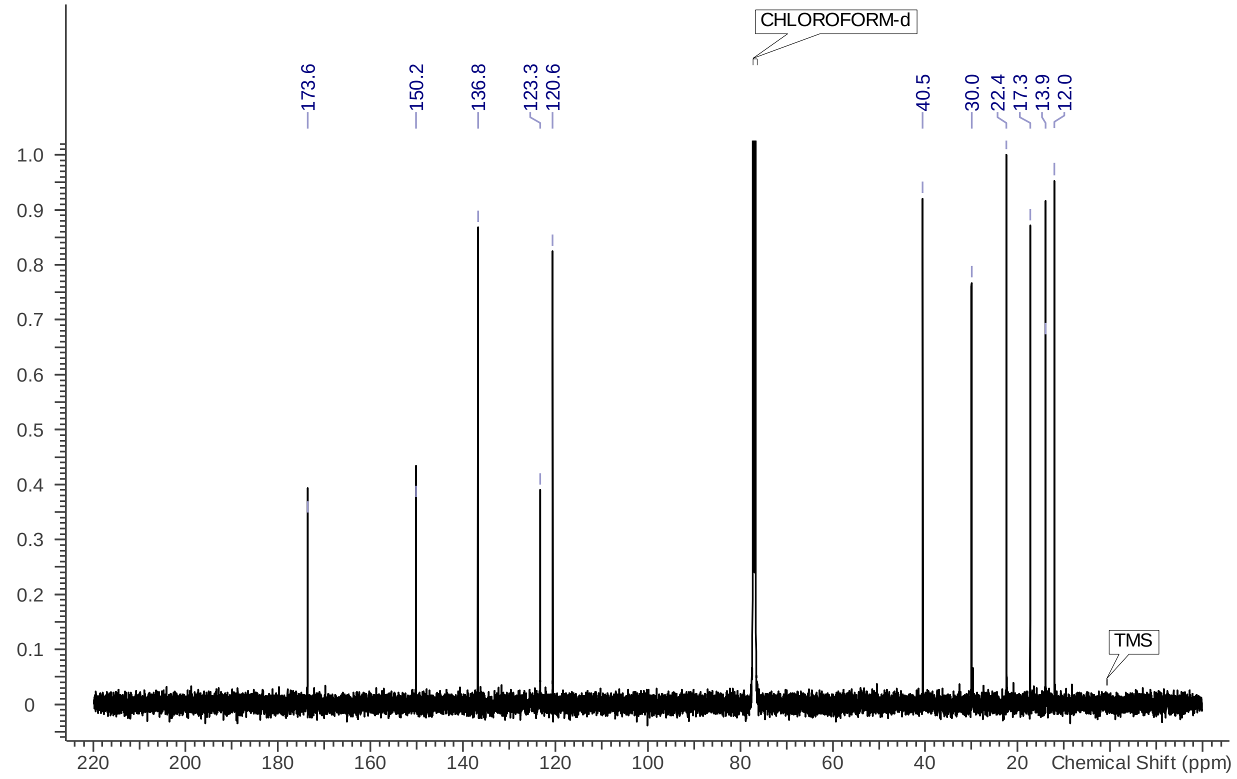

S-(2-Acetamidoethyl) (2E,4E)-2,5-dimethylnona-2,4-dienethioate (41)

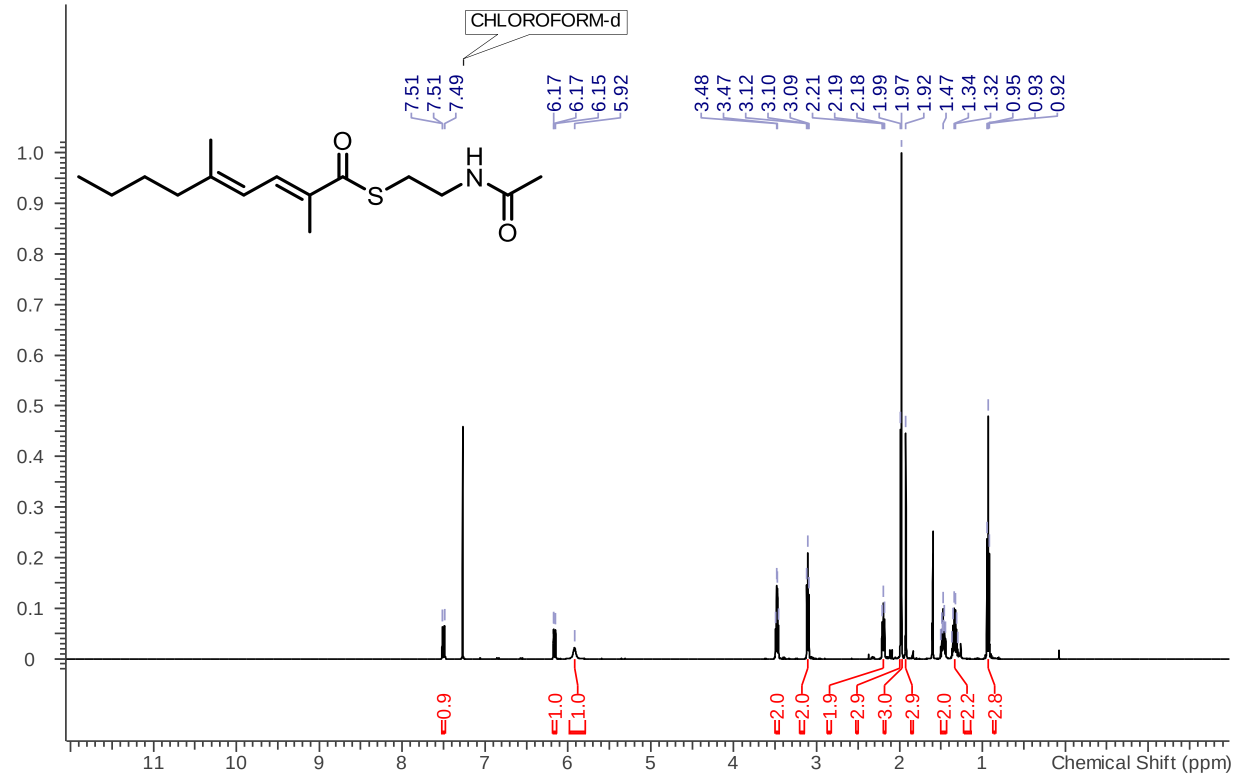

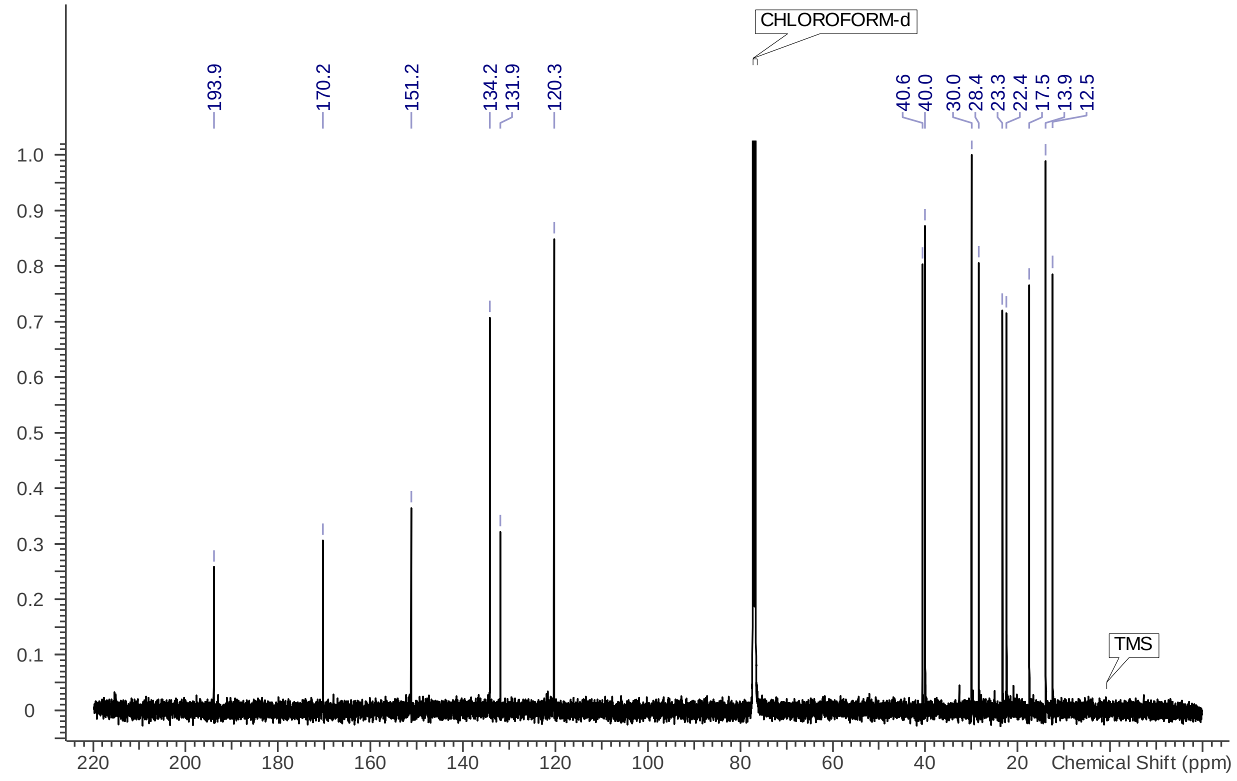

Ethyl (*E*)-6,6,6-trifluoro-3-methylhex-2-enoate (42a)

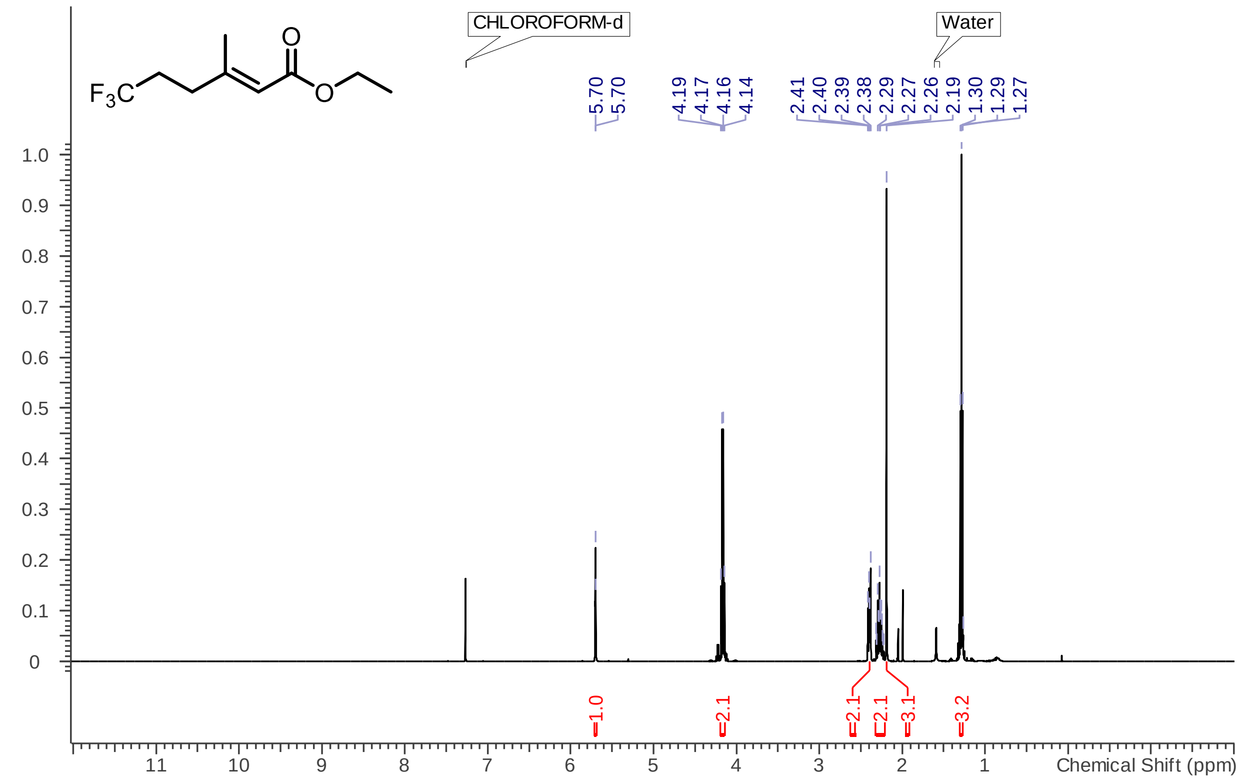

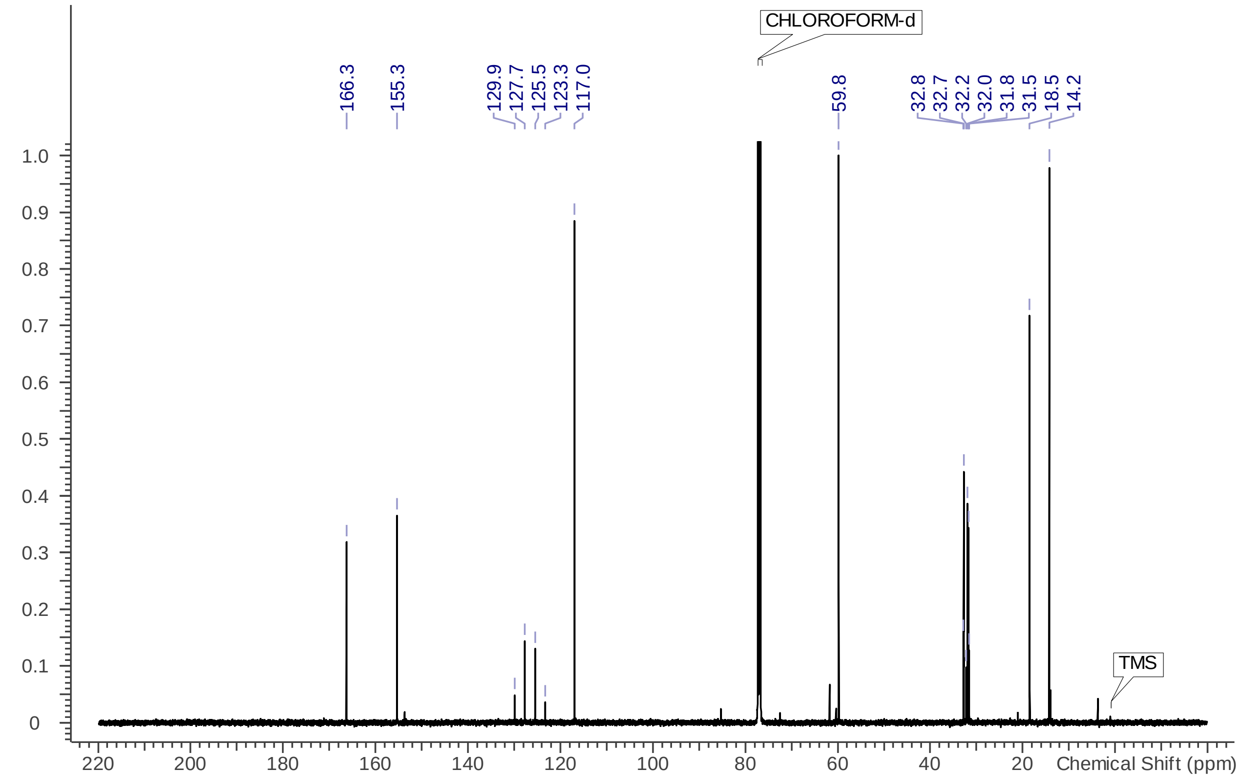

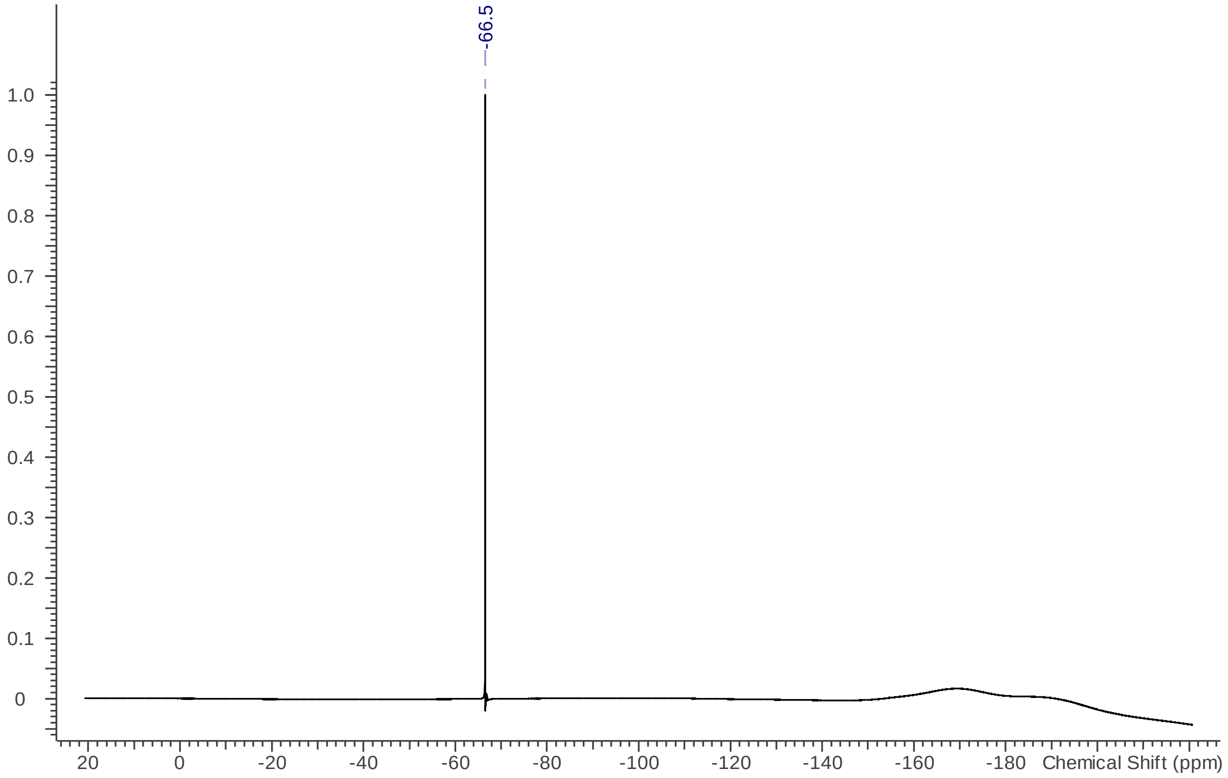

-66.5
1.0
0.9
0.8
0.7
0.6
0.5
0.4
0.3
0.2
0.1
0
20
0
-20
-40
-60
-80
-100
-120
-140
-160
-180
Chemical Shift (ppm)

Ethyl (2*E*,4*E*)-8,8,8-trifluoro-2,5-dimethylocta-2,4-dienoate (42b)

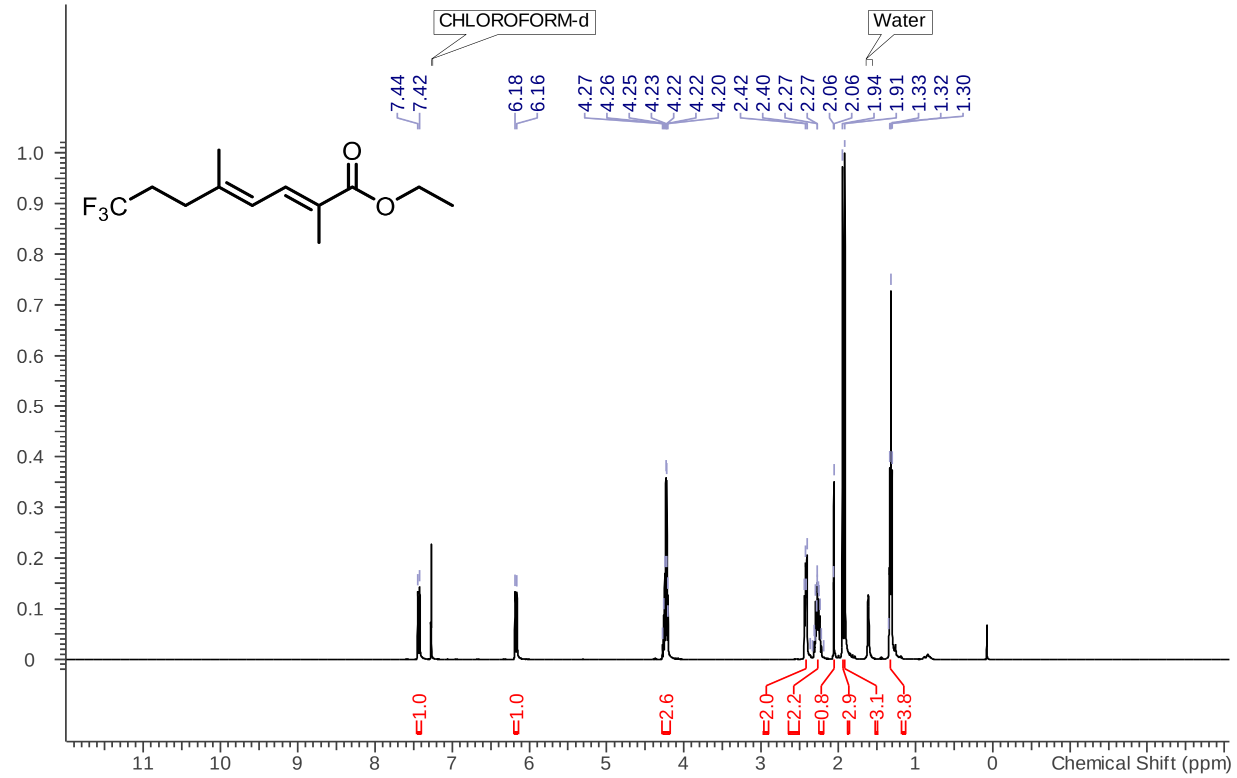

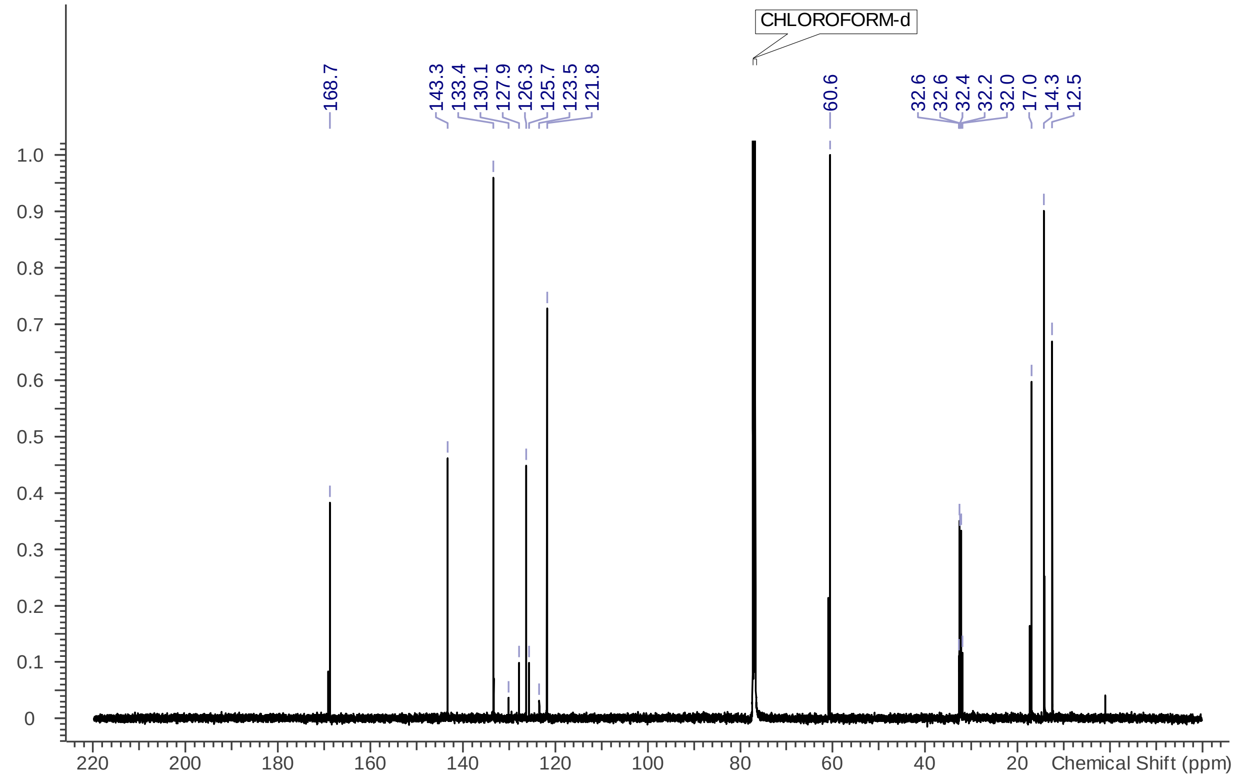

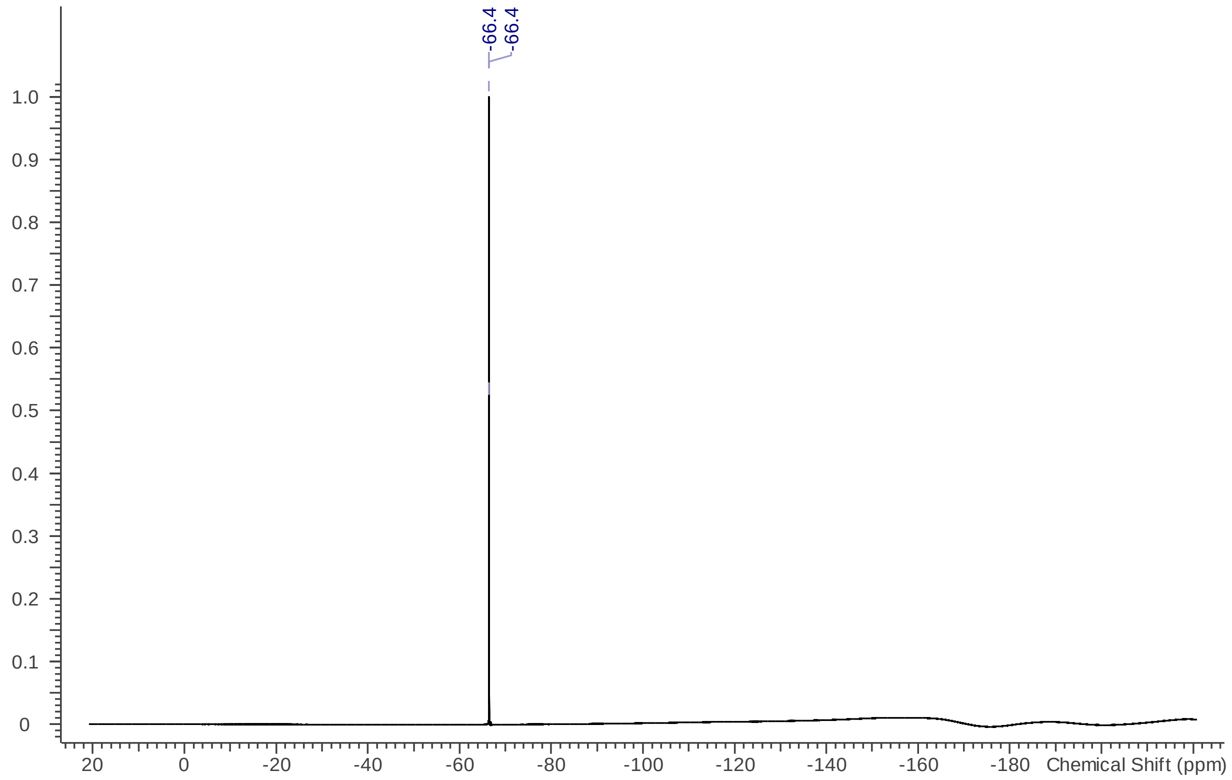
-66.4
-66.4
1.0
0.9
0.8
0.7
0.6
0.5
0.4
0.3
0.2
0.1
0
20
0
-20
-40
-60
-80
-100
-120
-140
-160
-180
Chemical Shift (ppm)

(2*E*,4*E*)-8,8,8-Trifluoro-2,5-dimethylocta-2,4-dienoic acid (42c)

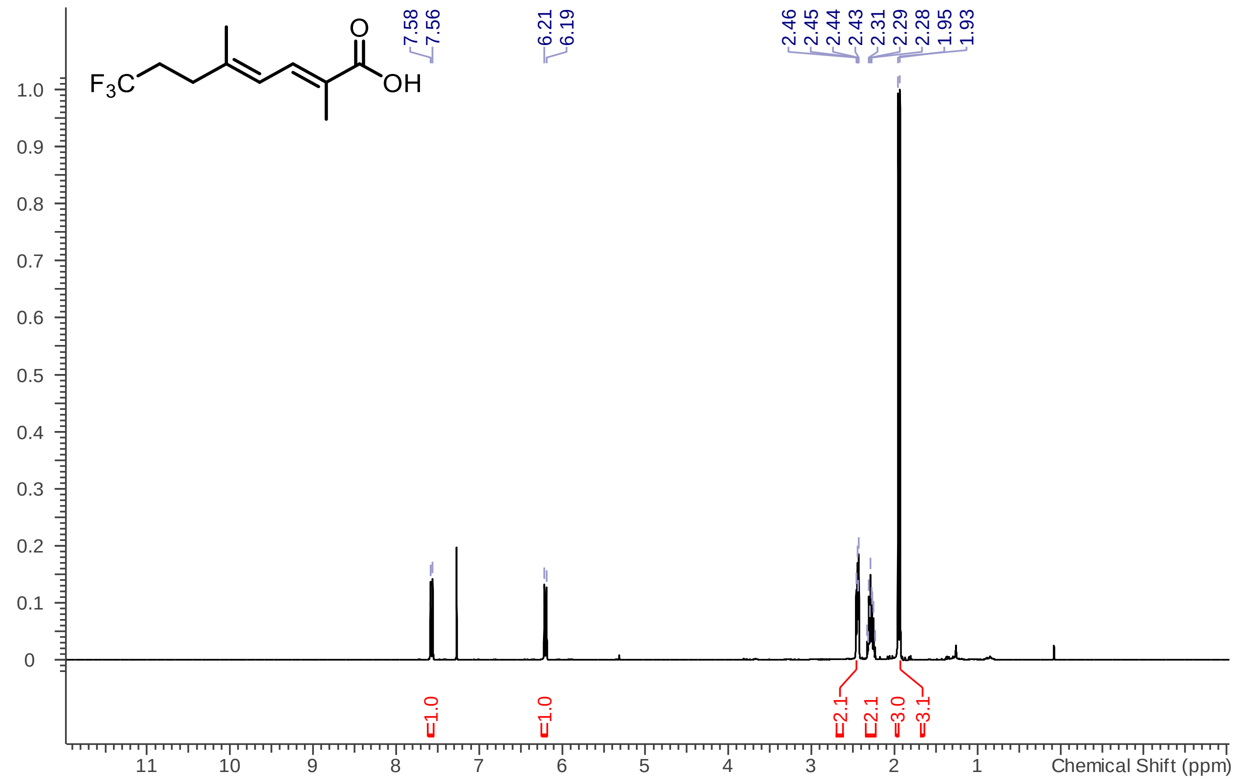

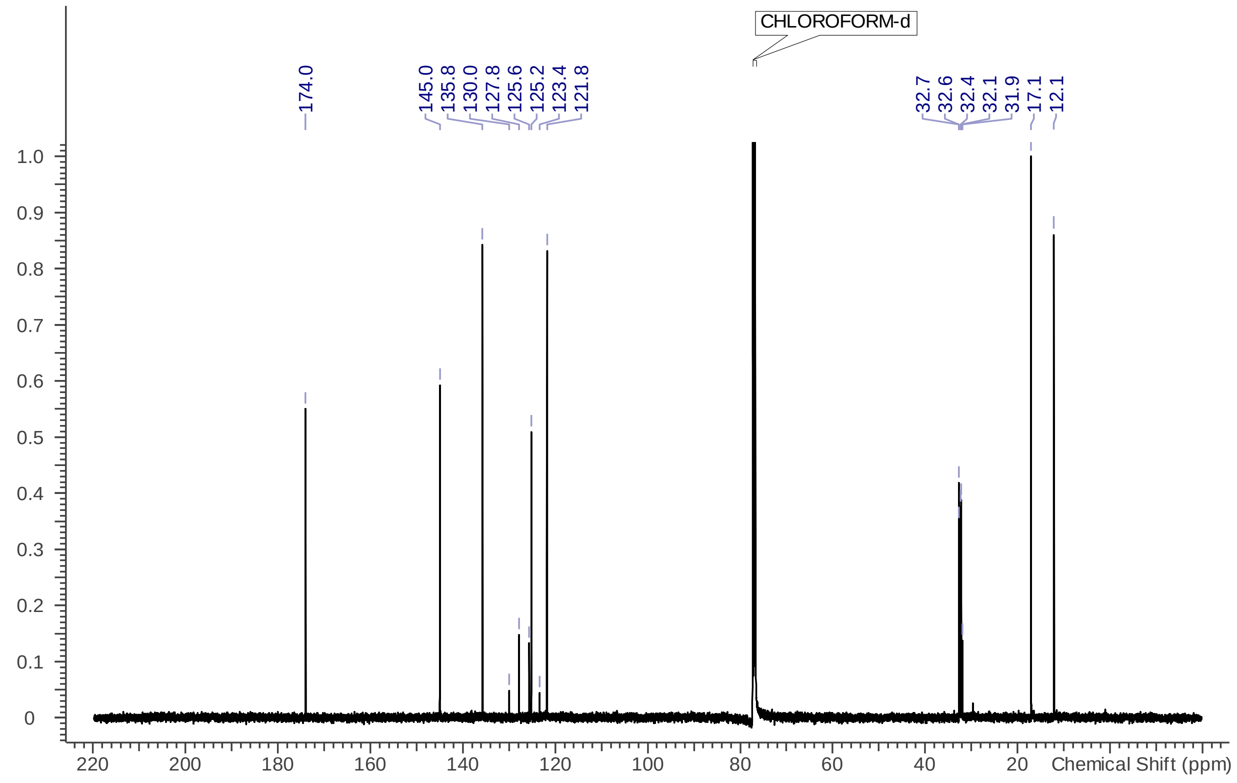

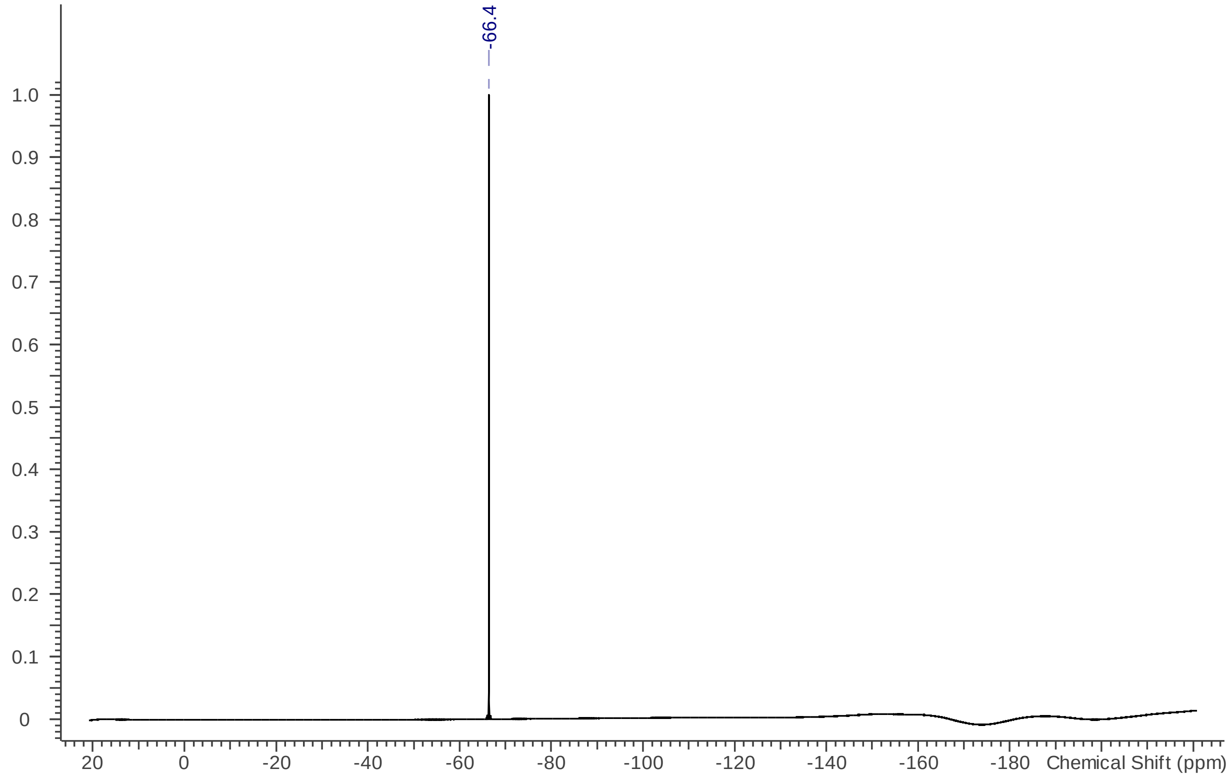
-66.4
1.0
0.9
0.8
0.7
0.6
0.5
0.4
0.3
0.2
0.1
0
20
0
-20
-40
-60
-80
-100
-120
-140
-160
-180
Chemical Shift (ppm)

S-(2-Acetamidoethyl) (2E,4E)-8,8,8-trifluoro-2,5-dimethylocta-2,4-dienethioate (42)

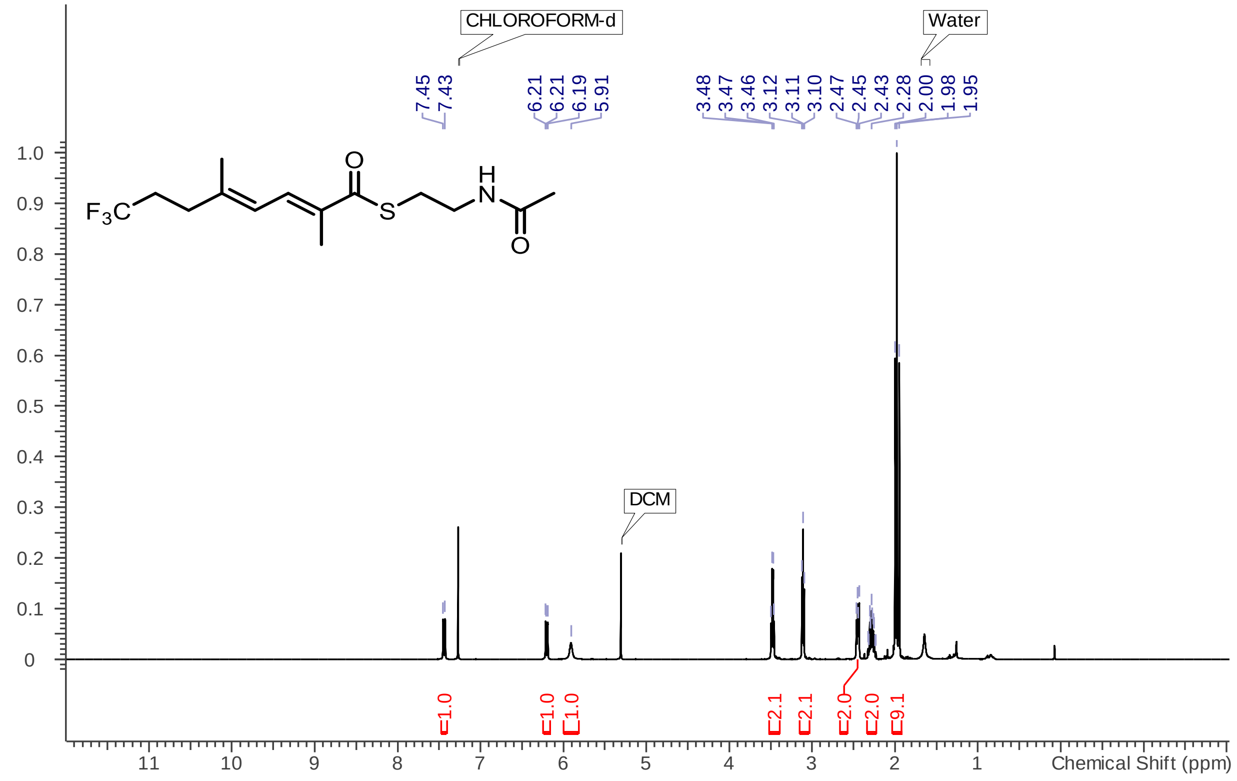

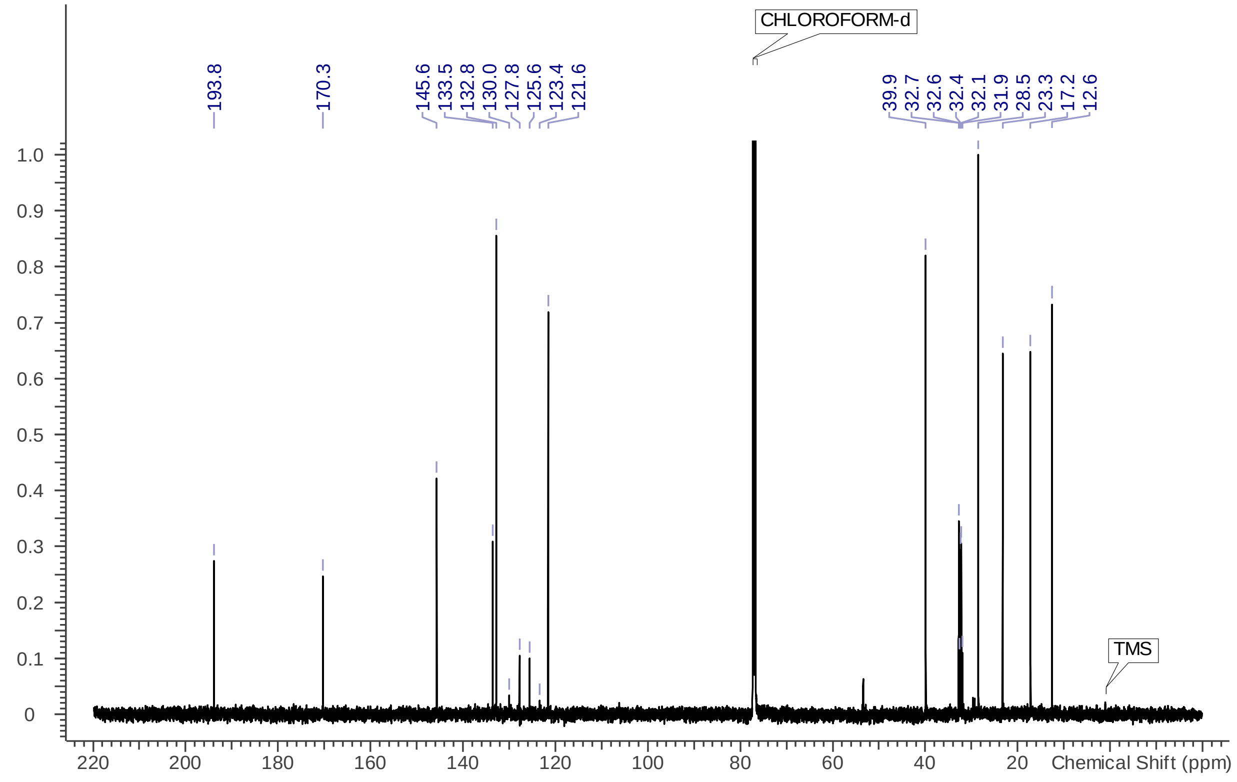

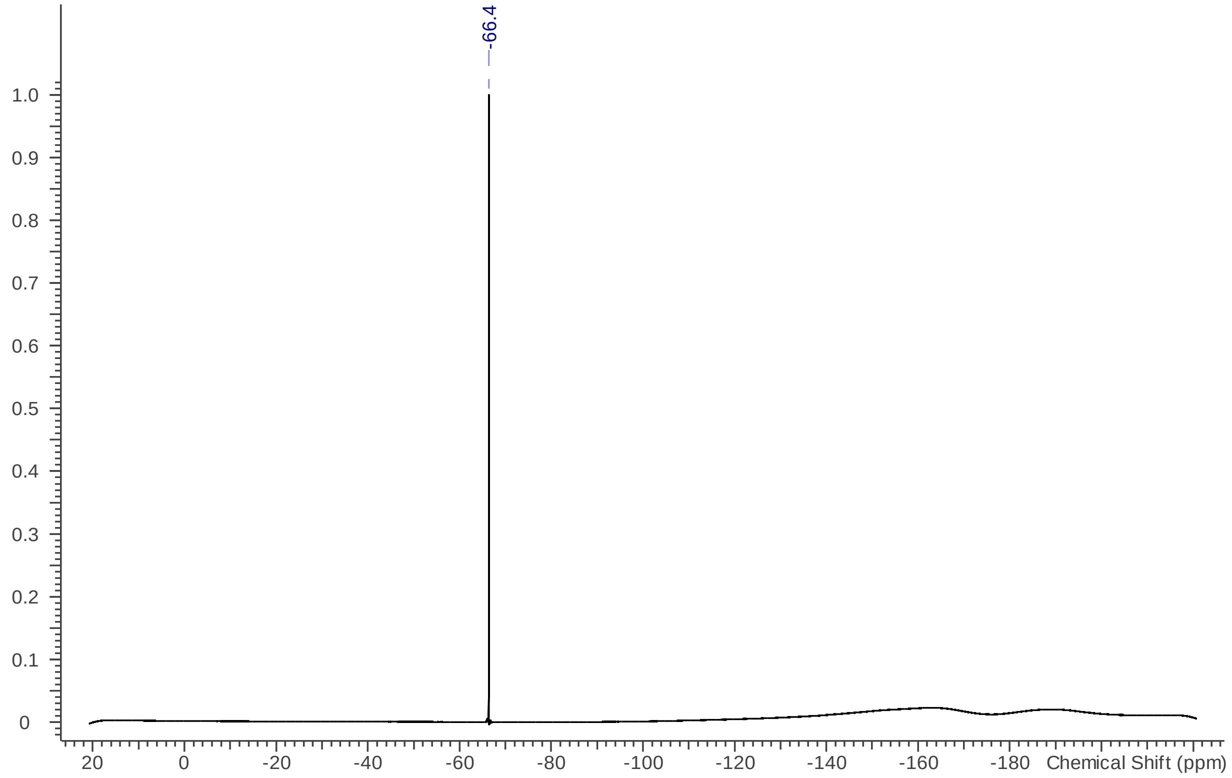
-66.4
1.0
0.9
0.8
0.7
0.6
0.5
0.4
0.3
0.2
0.1
0
20
0
-20
-40
-60
-80
-100
-120
-140
-160
-180
Chemical Shift (ppm)

(4-Bromobut-1-yn-1-yl)trimethylsilane (43aa)

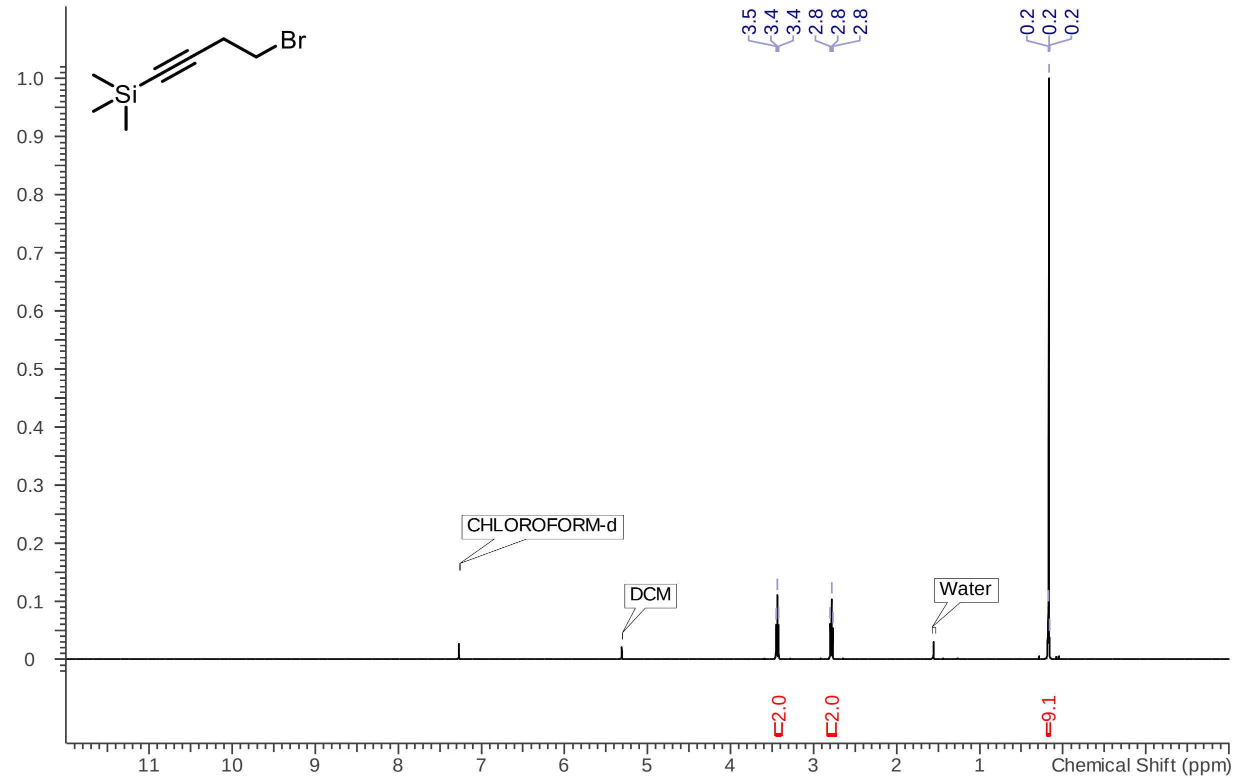

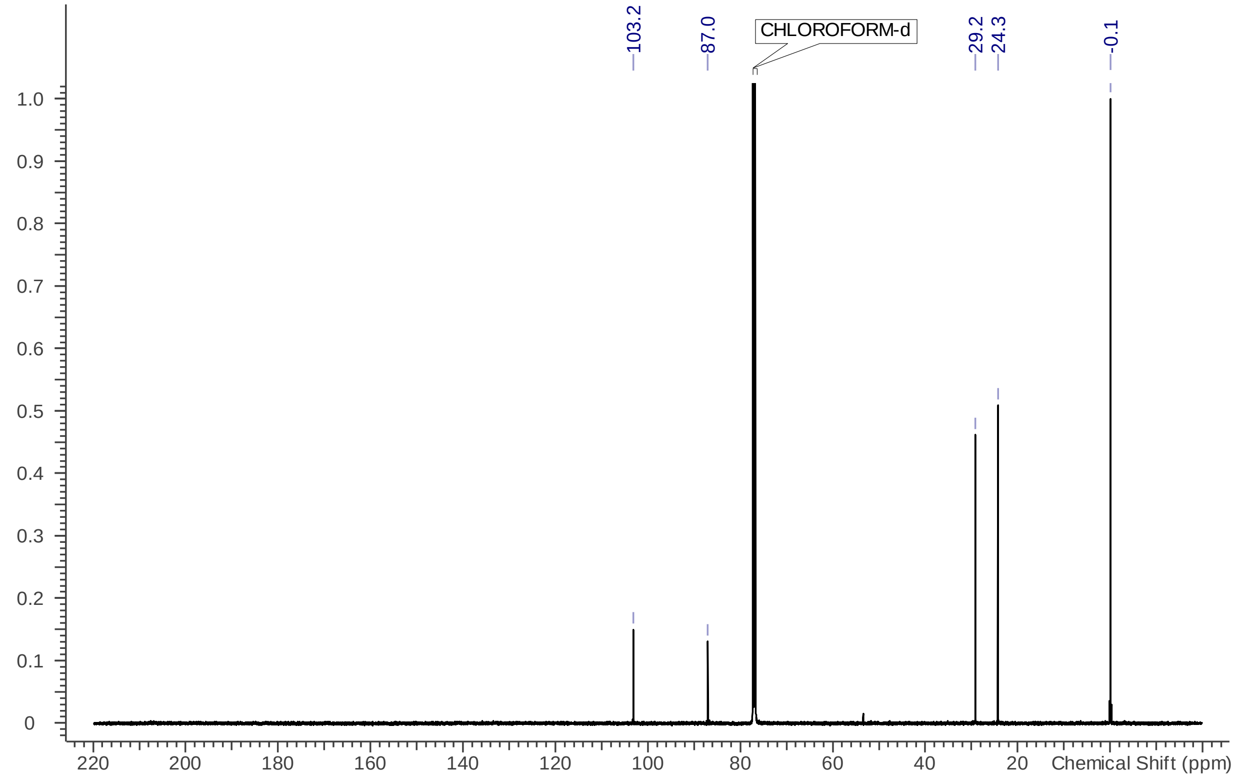

Ethyl (*E*)-3-methyl-7-(trimethylsilyl)hept-2-en-6-ynoate (43a)

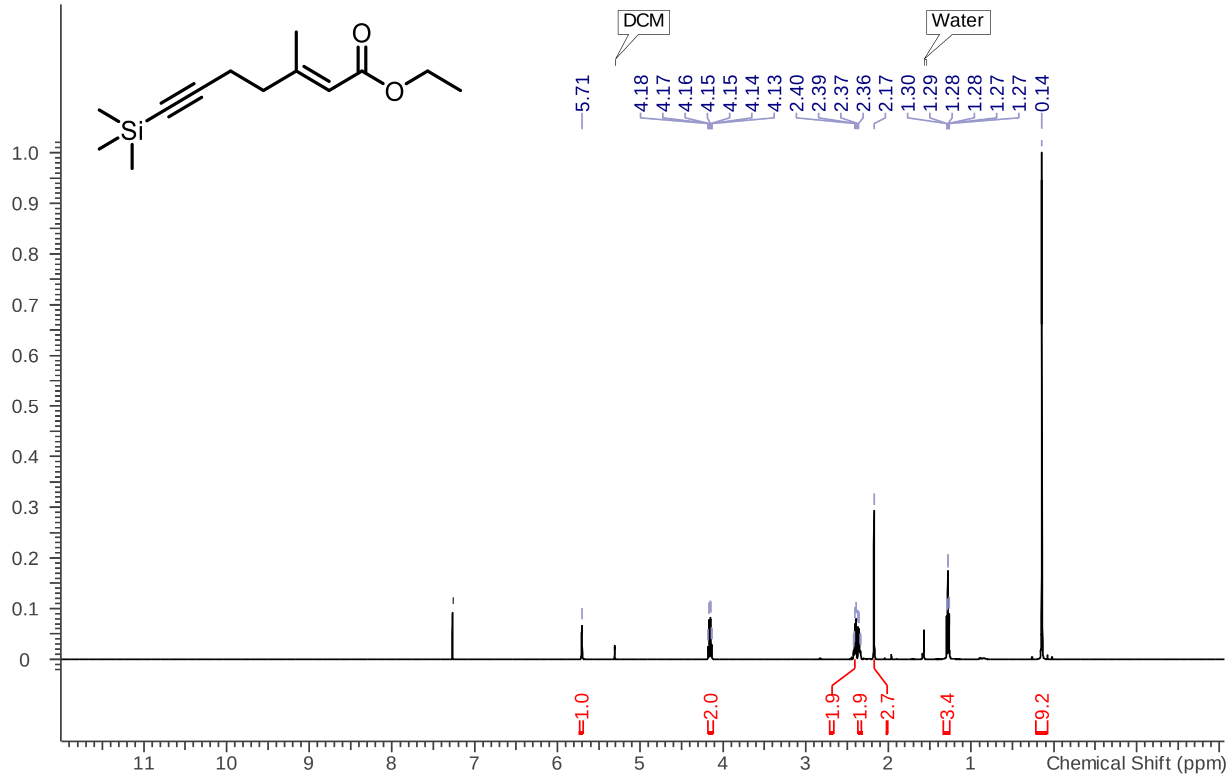

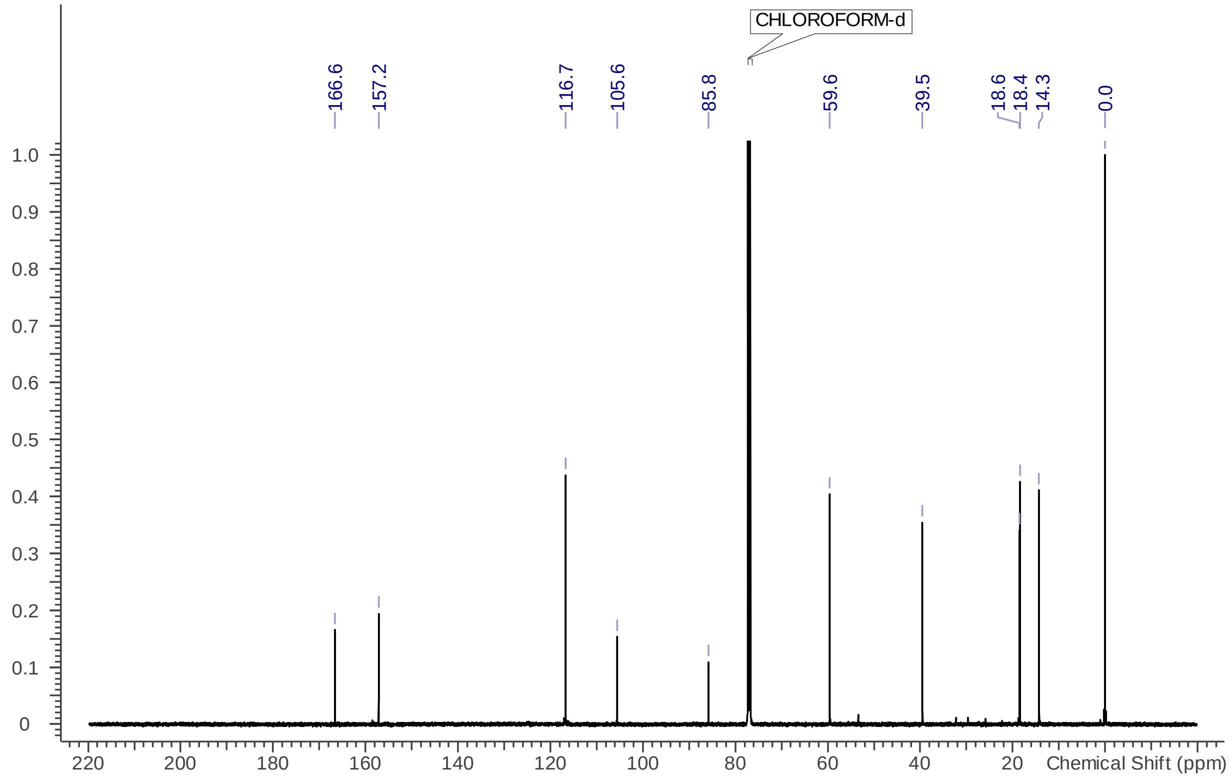

Ethyl (2*E*,4*E*)-2,5-dimethyl-9-(trimethylsilyl)nona-2,4-dien-8-ynoate (43b)

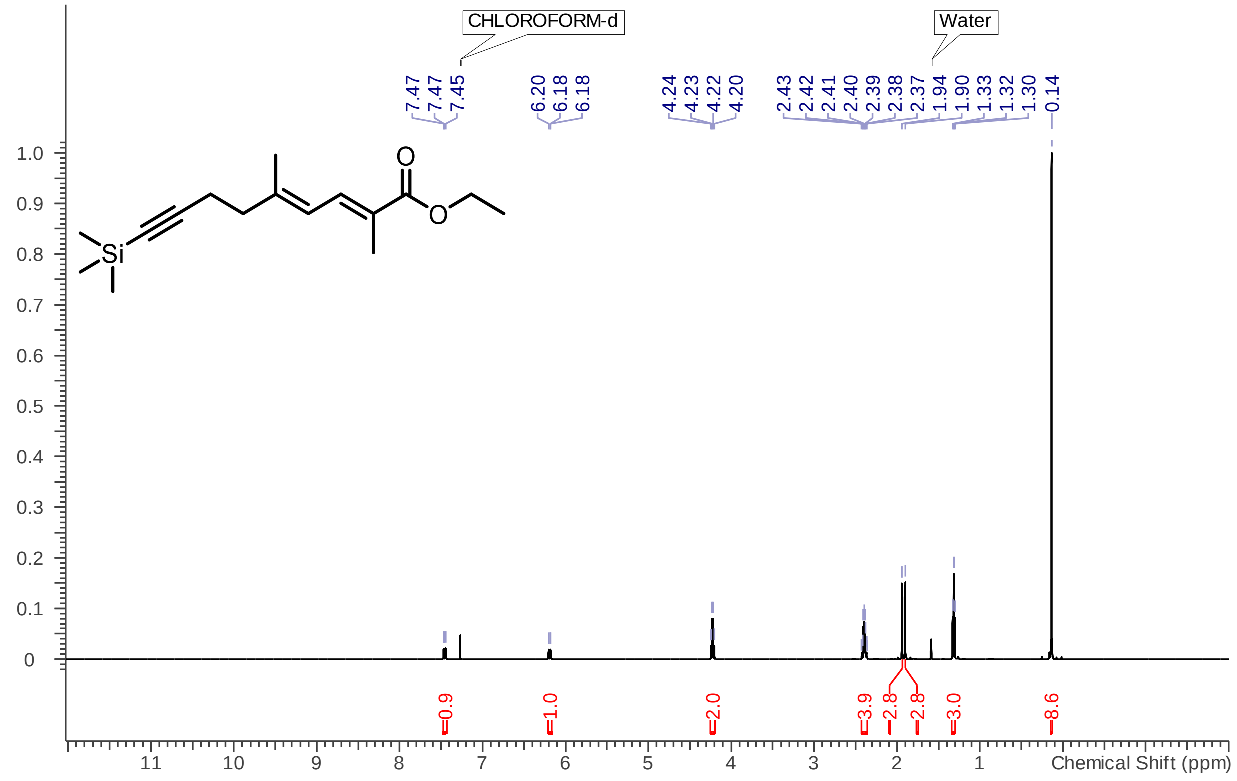

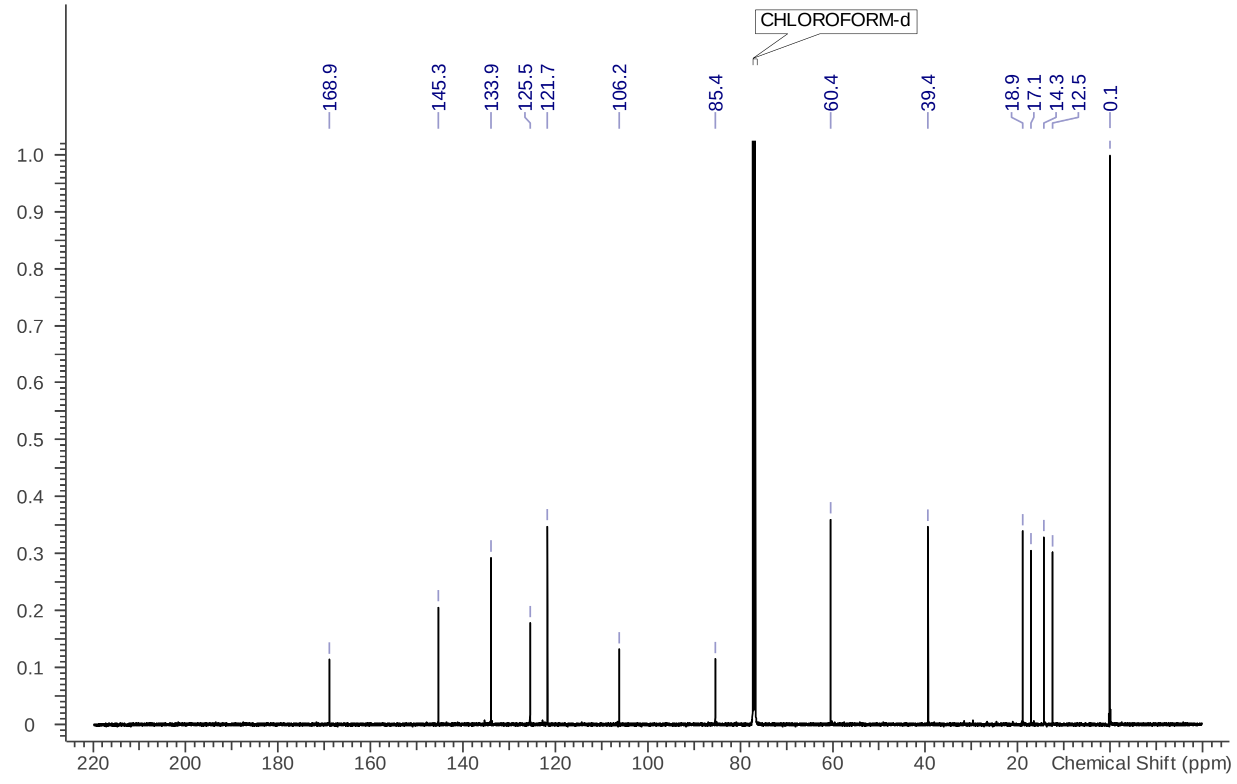

(2*E*,4*E*)-2,5-Dimethylnona-2,4-dien-8-ynoic acid (43c)

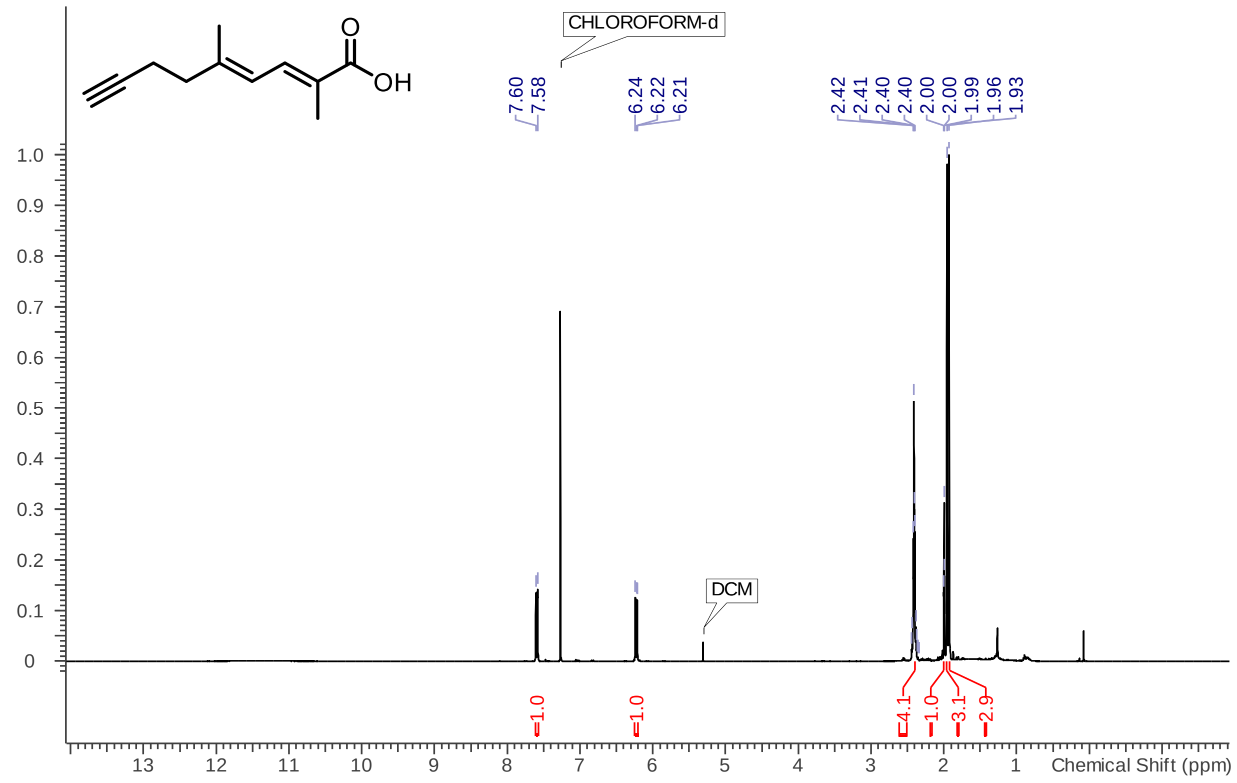

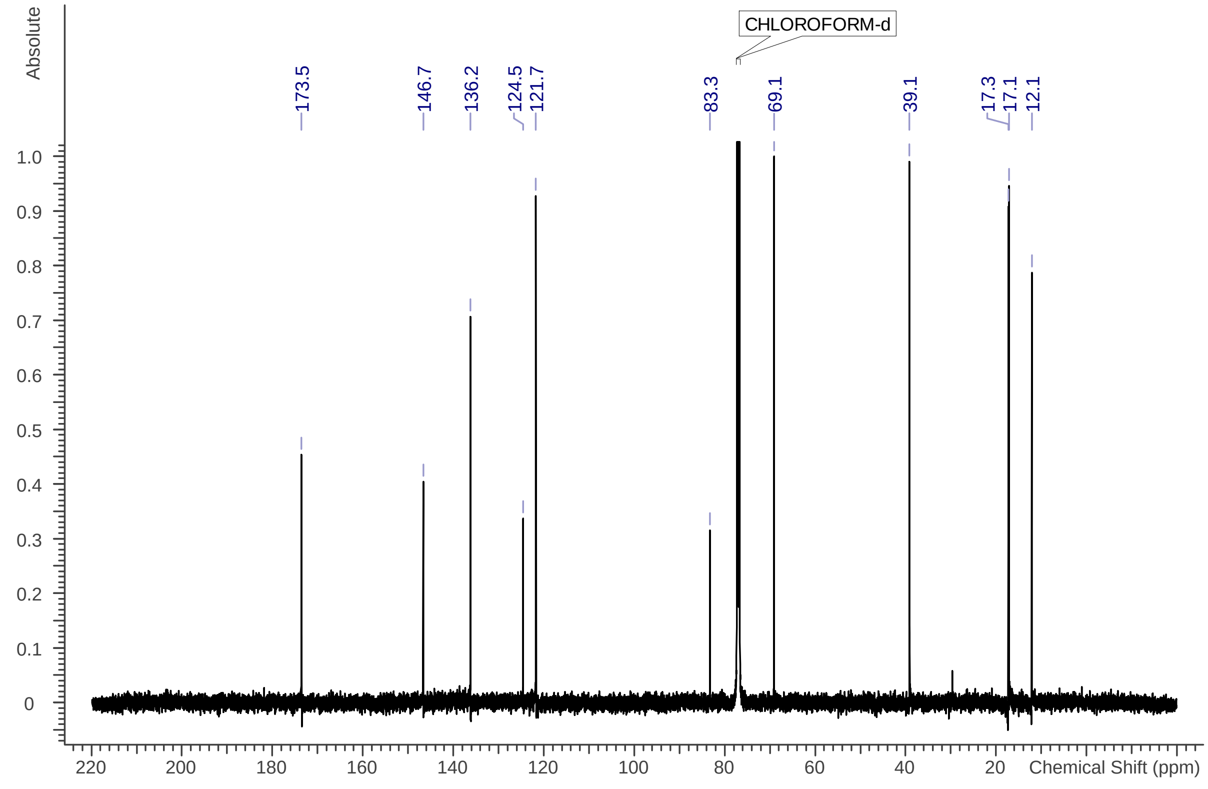

_S_-(2-Acetamidoethyl) (2_E_,4_E_)-2,5-dimethylnona-2,4-dien-8-ynethioate (43)

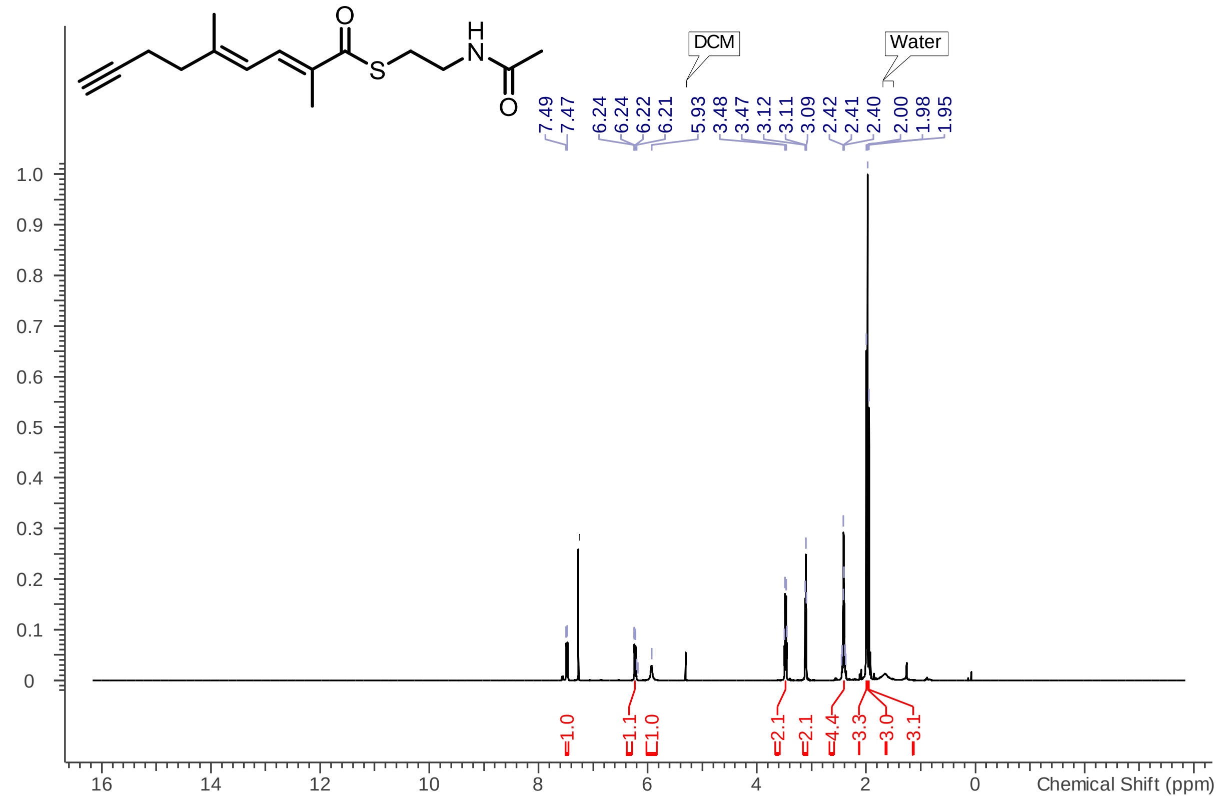

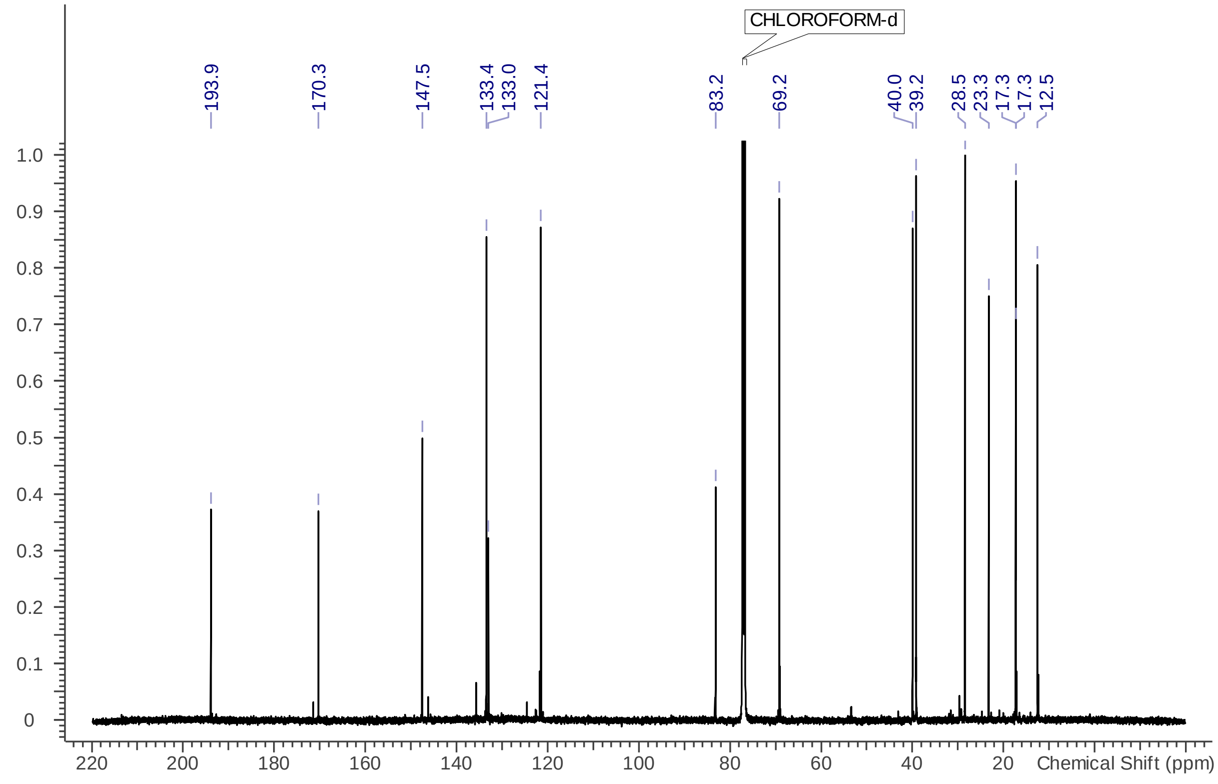

Ethyl (*E*)-3-cyclohexylbut-2-enoate (44a)

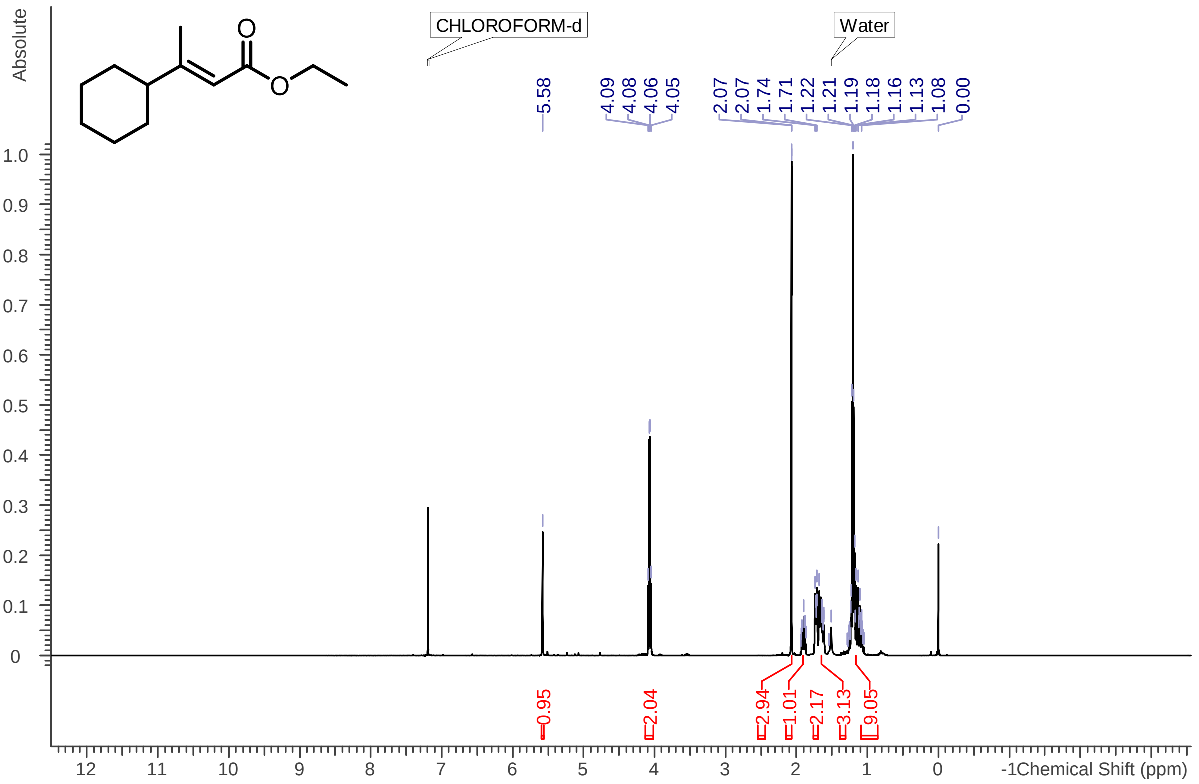

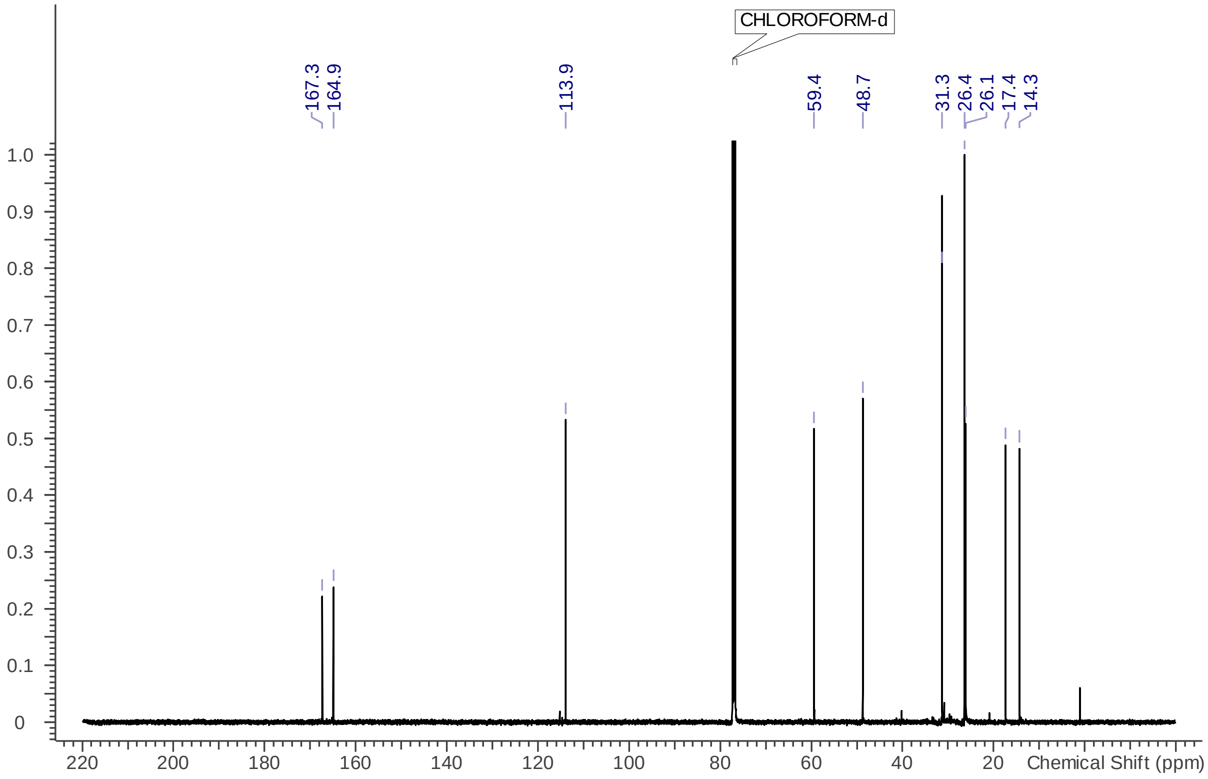

Ethyl (2*E*,4*E*)-5-cyclohexyl-2-methylhexa-2,4-dienoate (44b)

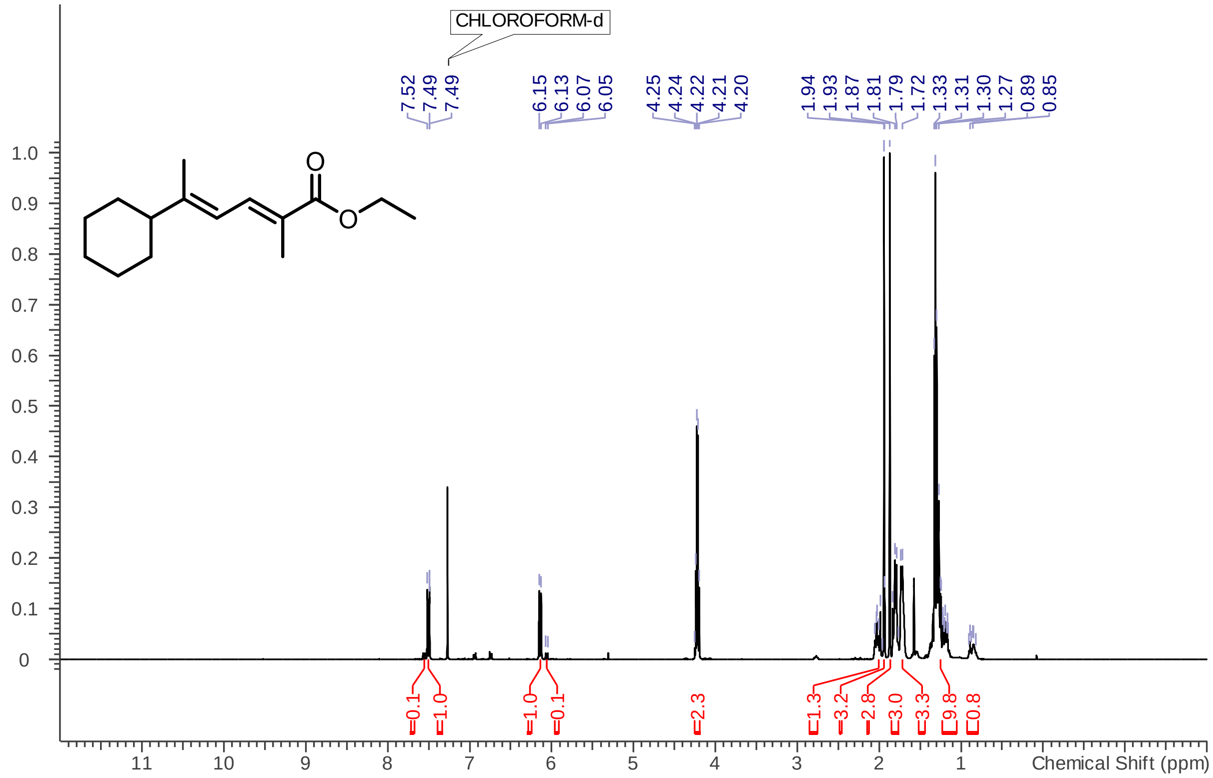

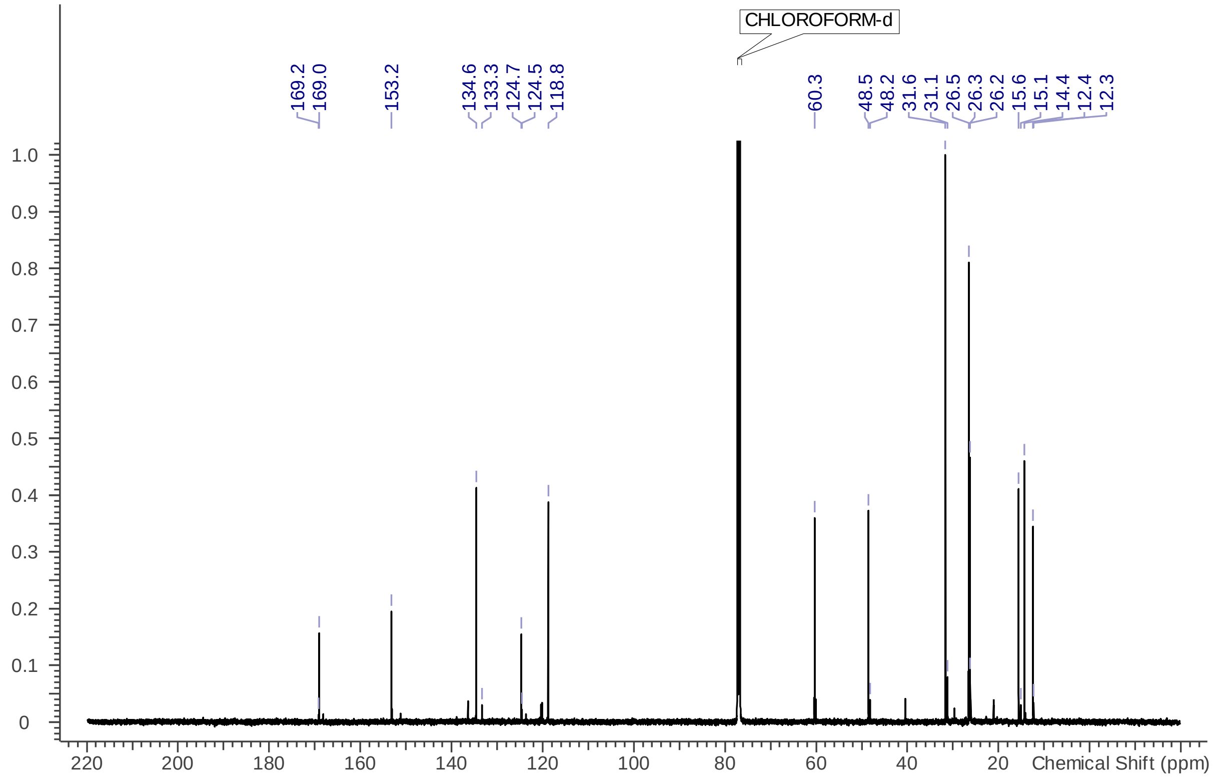

(2*E*,4*E*)-5-Cyclohexyl-2-methylhexa-2,4-dienoic acid (44c)

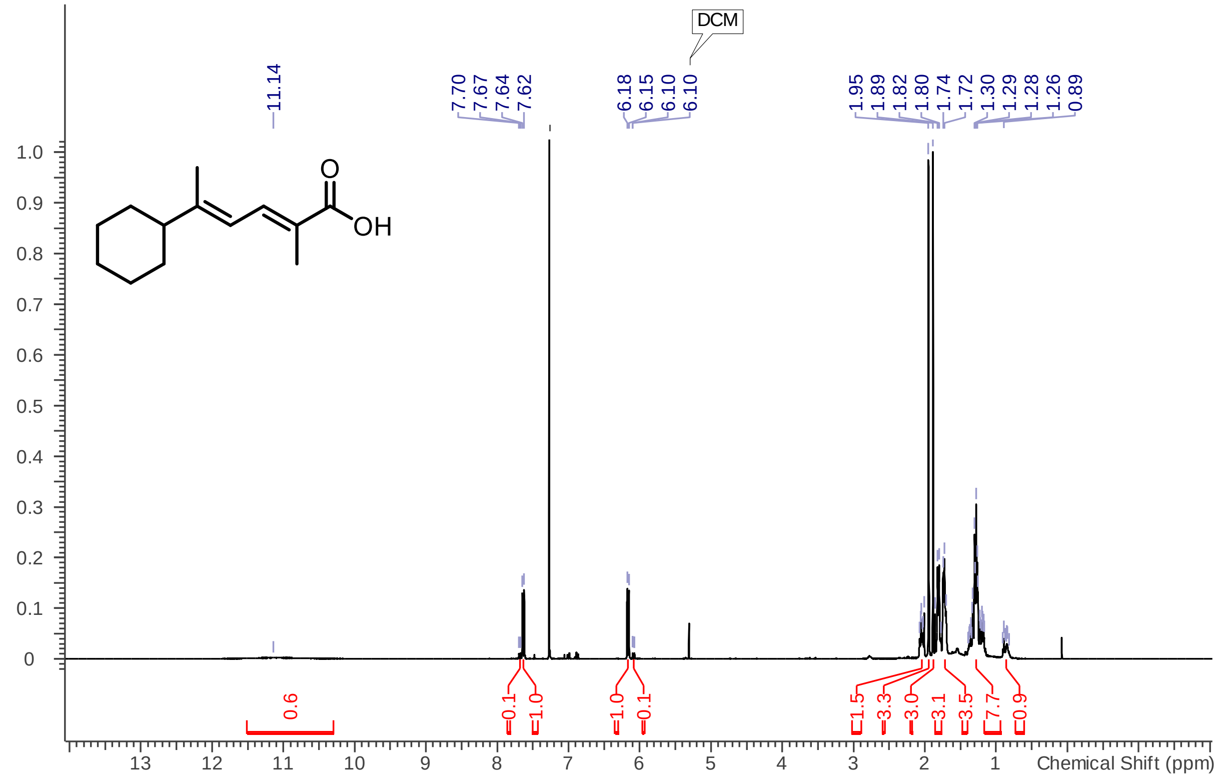

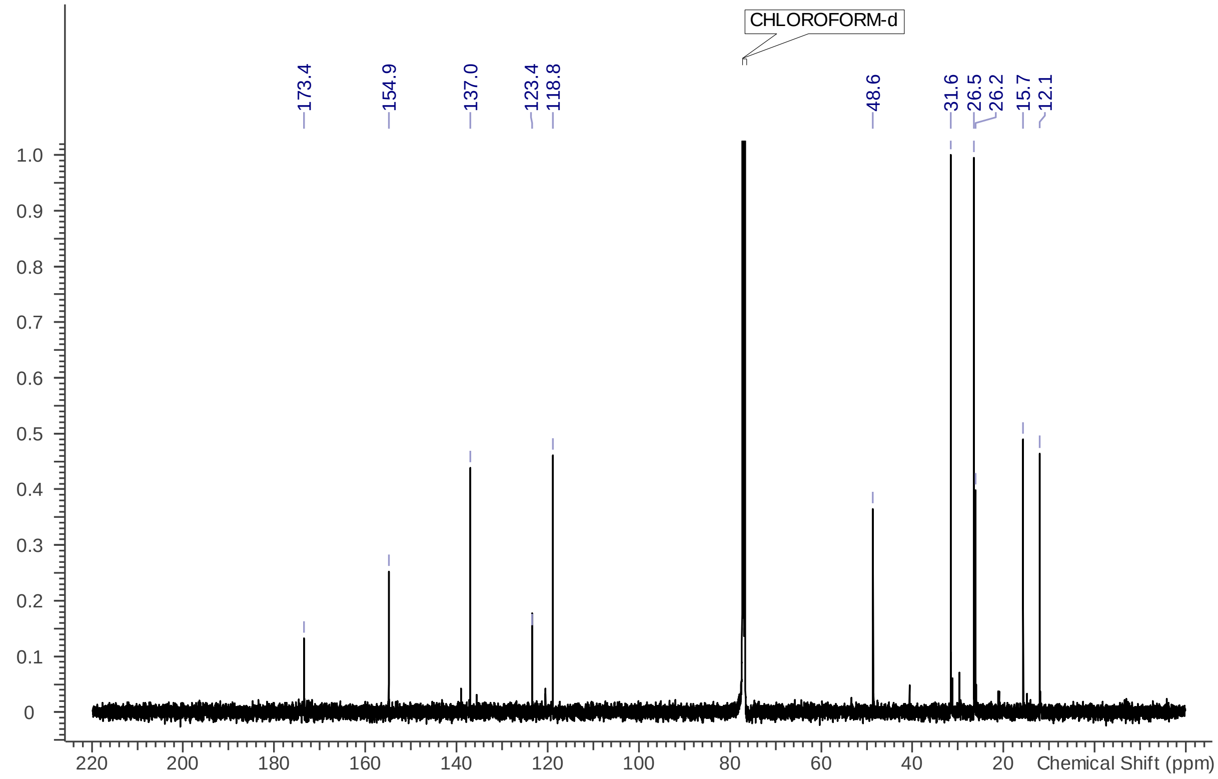

_S_-(2-Acetamidoethyl) (2_E_,4_E_)-5-cyclohexyl-2-methylhexa-2,4-dienethioate (44)

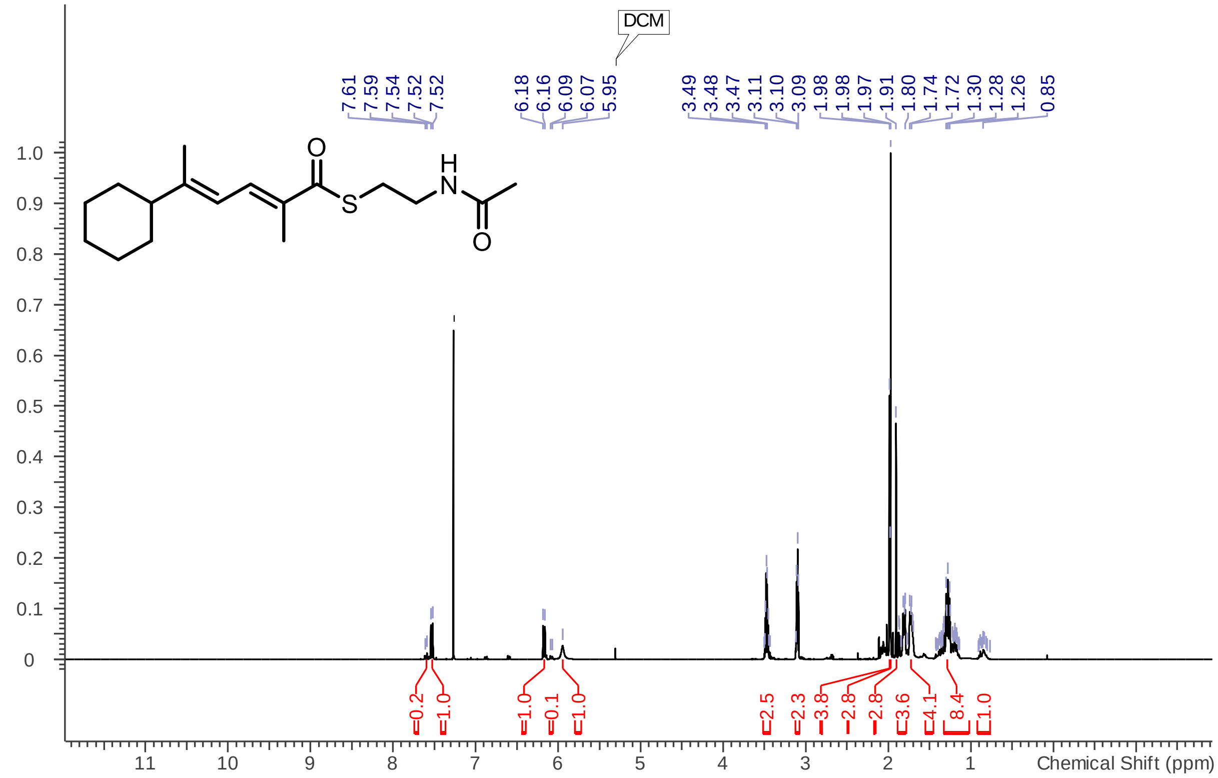

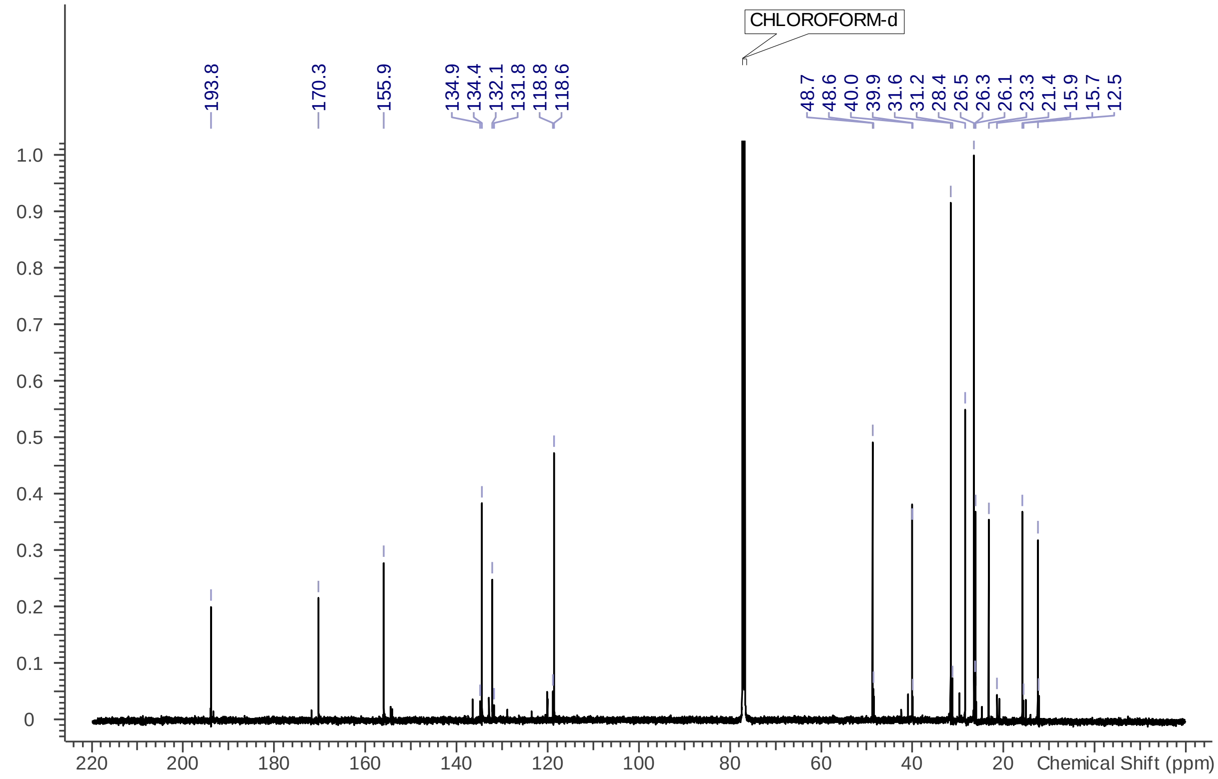

Ethyl (2*E*,4*E*)-2-methylocta-2,4-dienoate (45b)

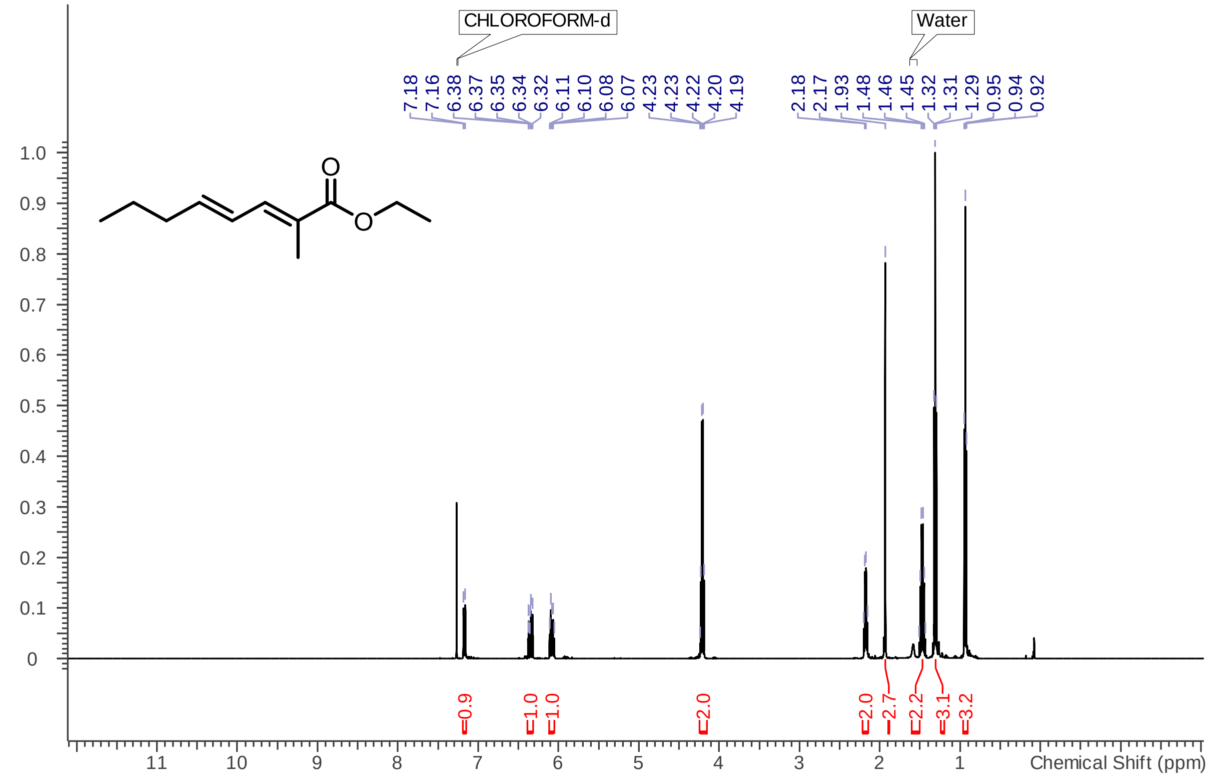

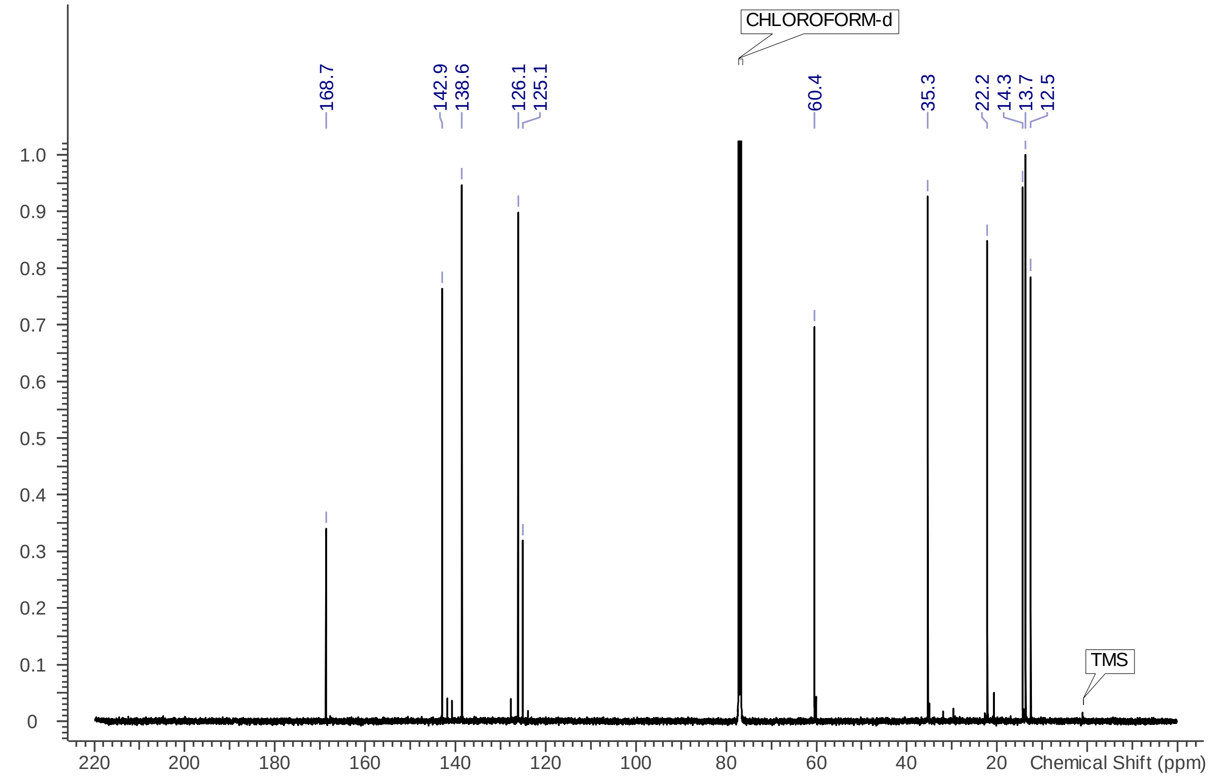

(2*E*,4*E*)-2-Methylocta-2,4-dienoic acid (45c)

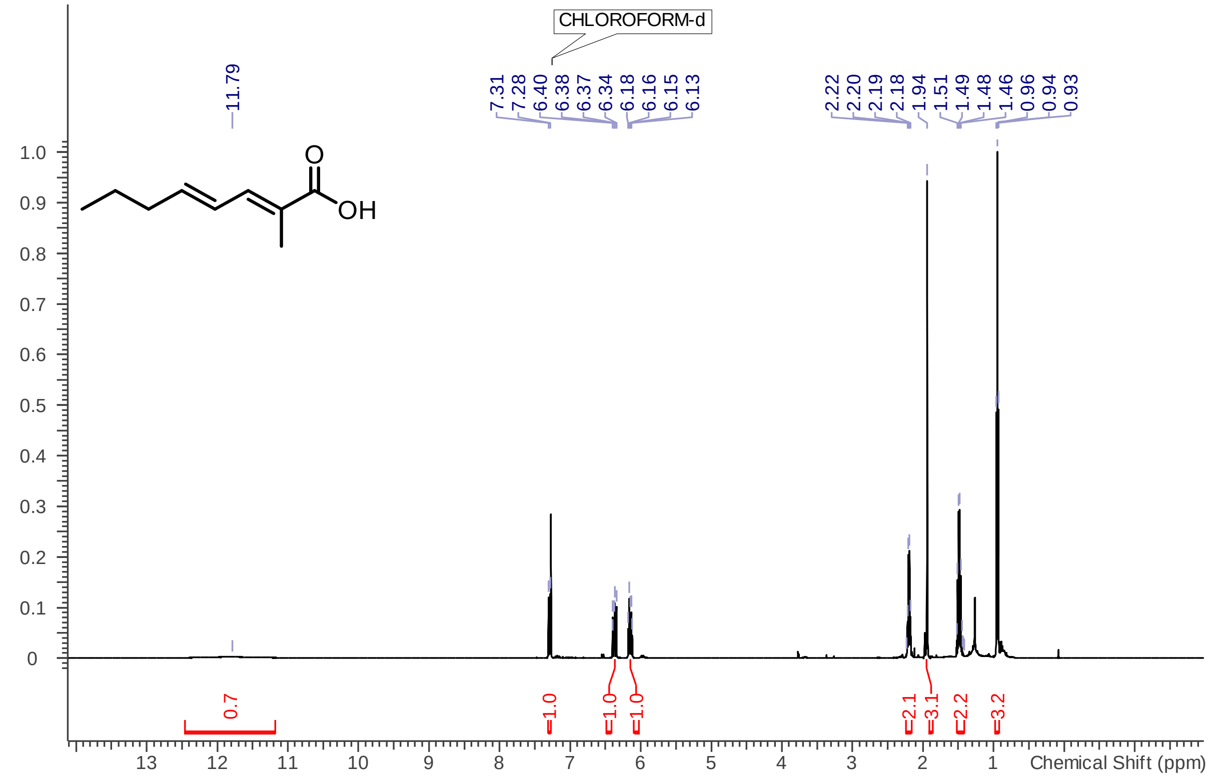

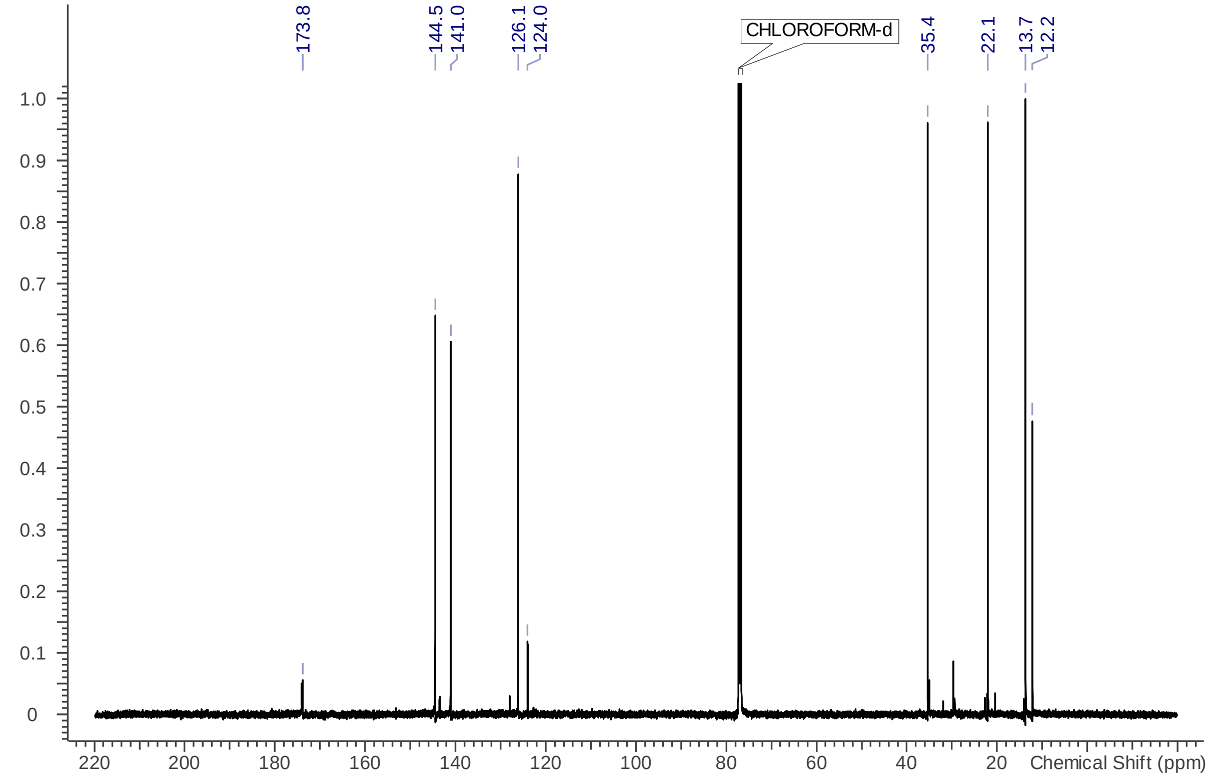

S-(2-Acetamidoethyl) (2E,4E)-2-methylocta-2,4-dienethioate (45)

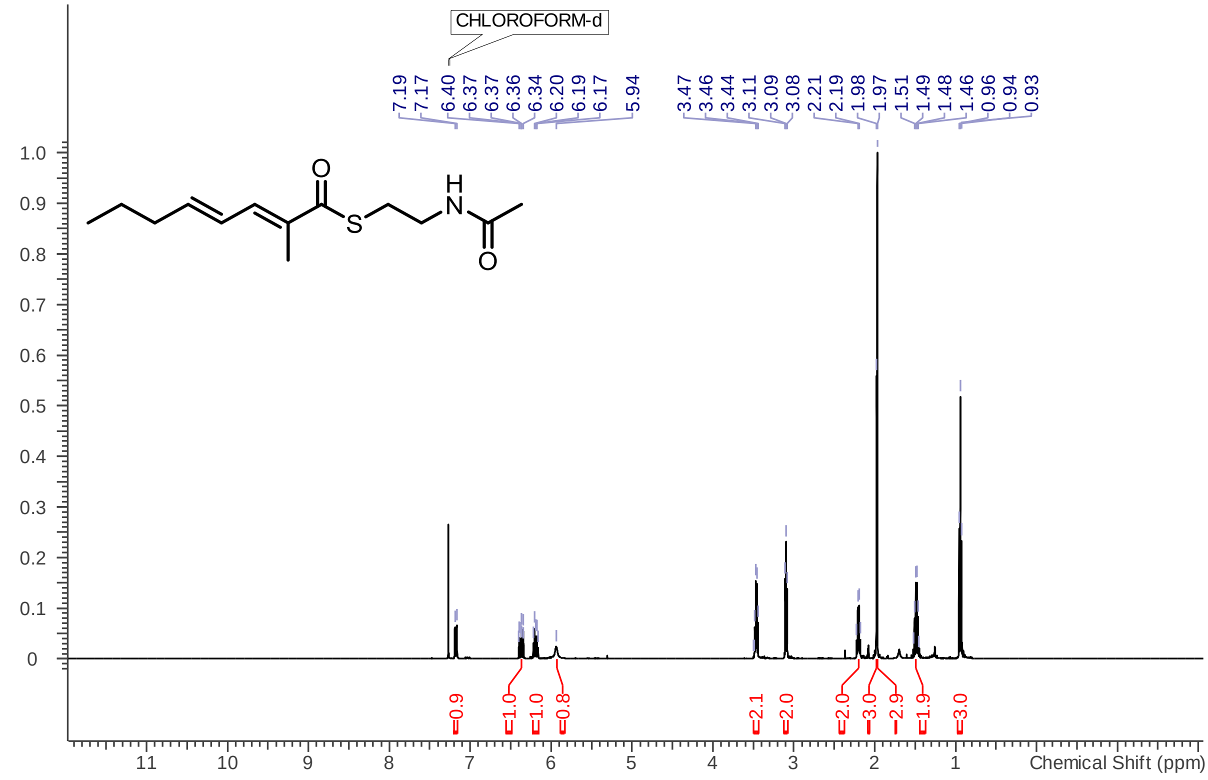

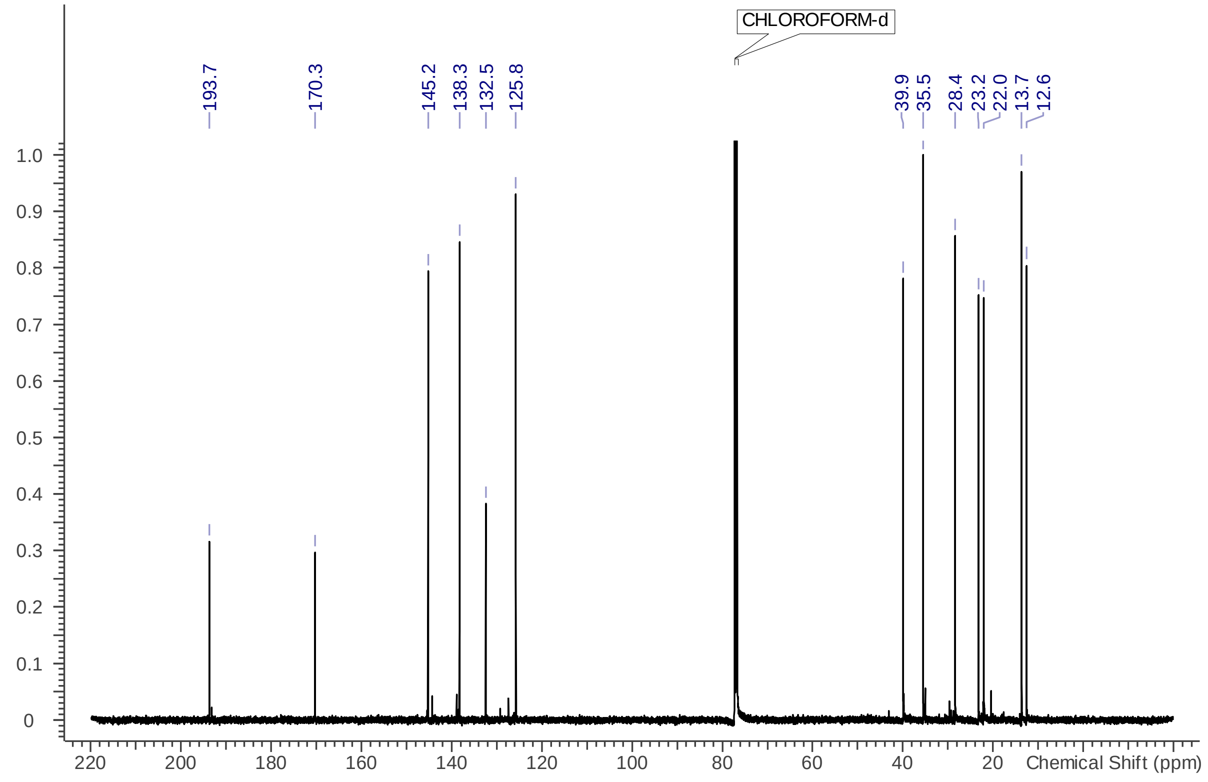

Ethyl (2*E*,4*E*)-5-methylocta-2,4-dienoate (46b)

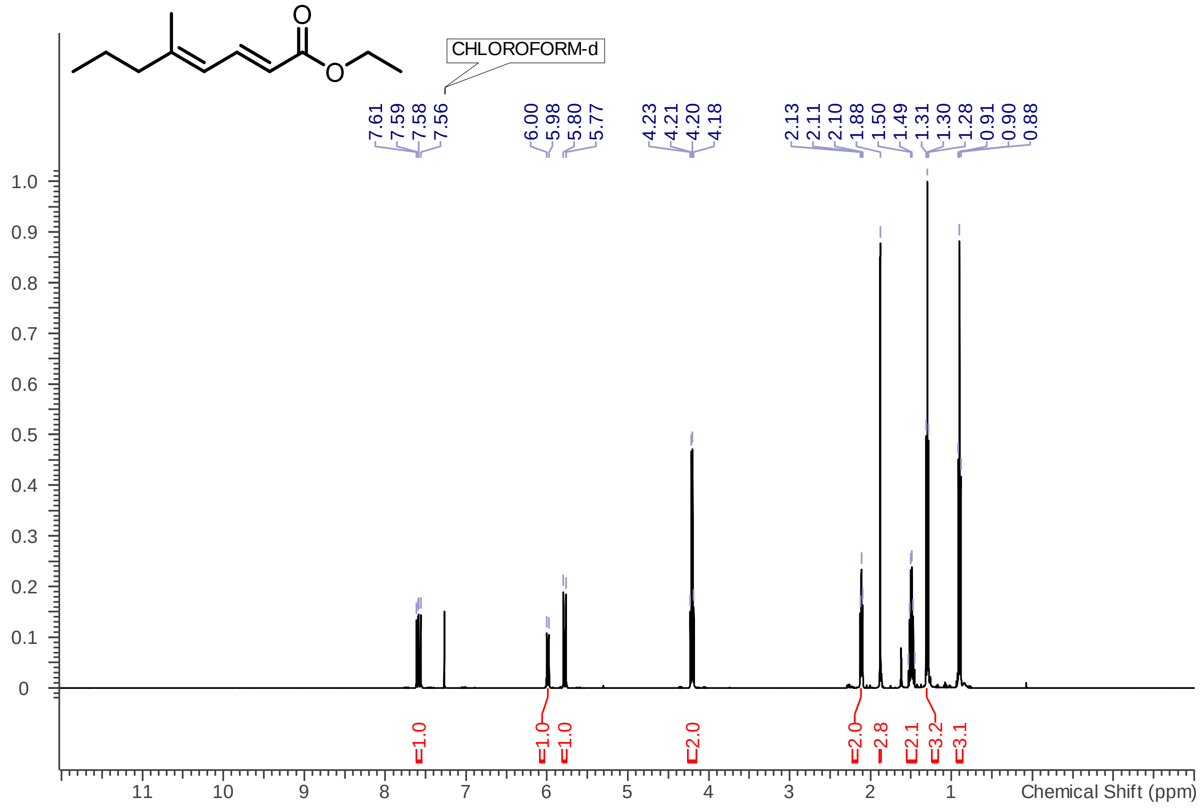

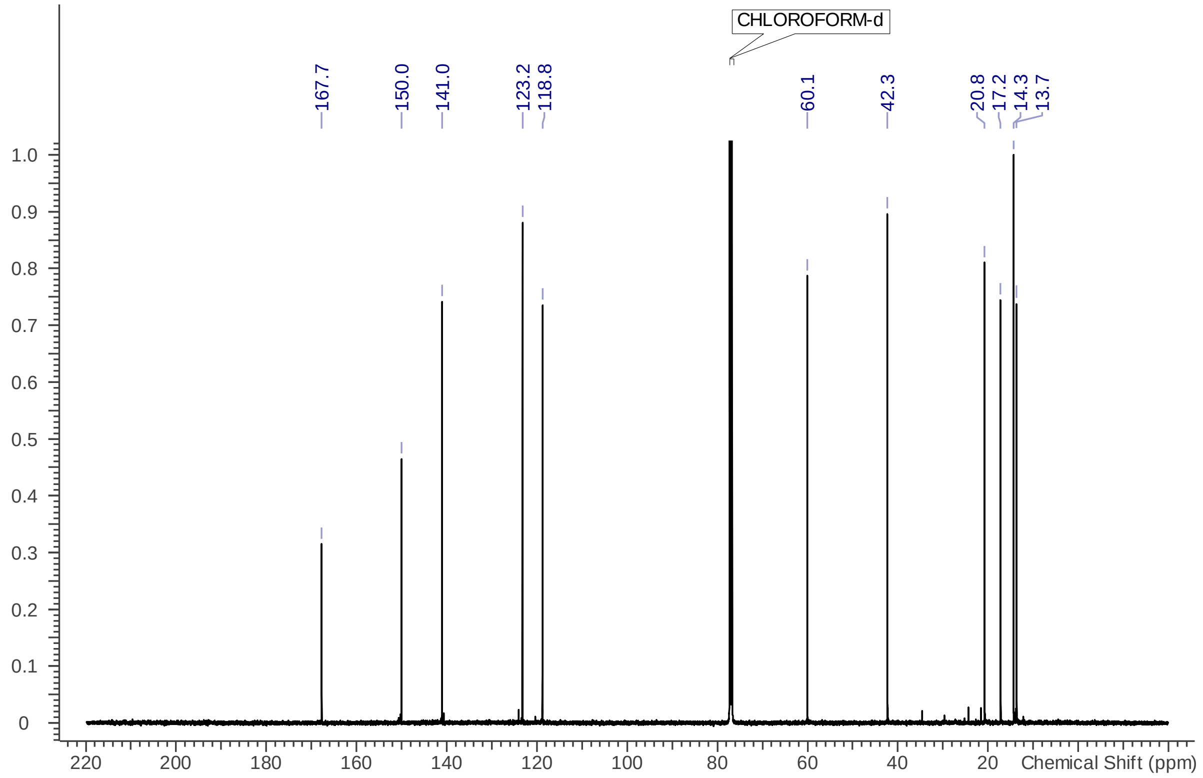

(2*E*,4*E*)-5-Methylocta-2,4-dienoic acid (46c)

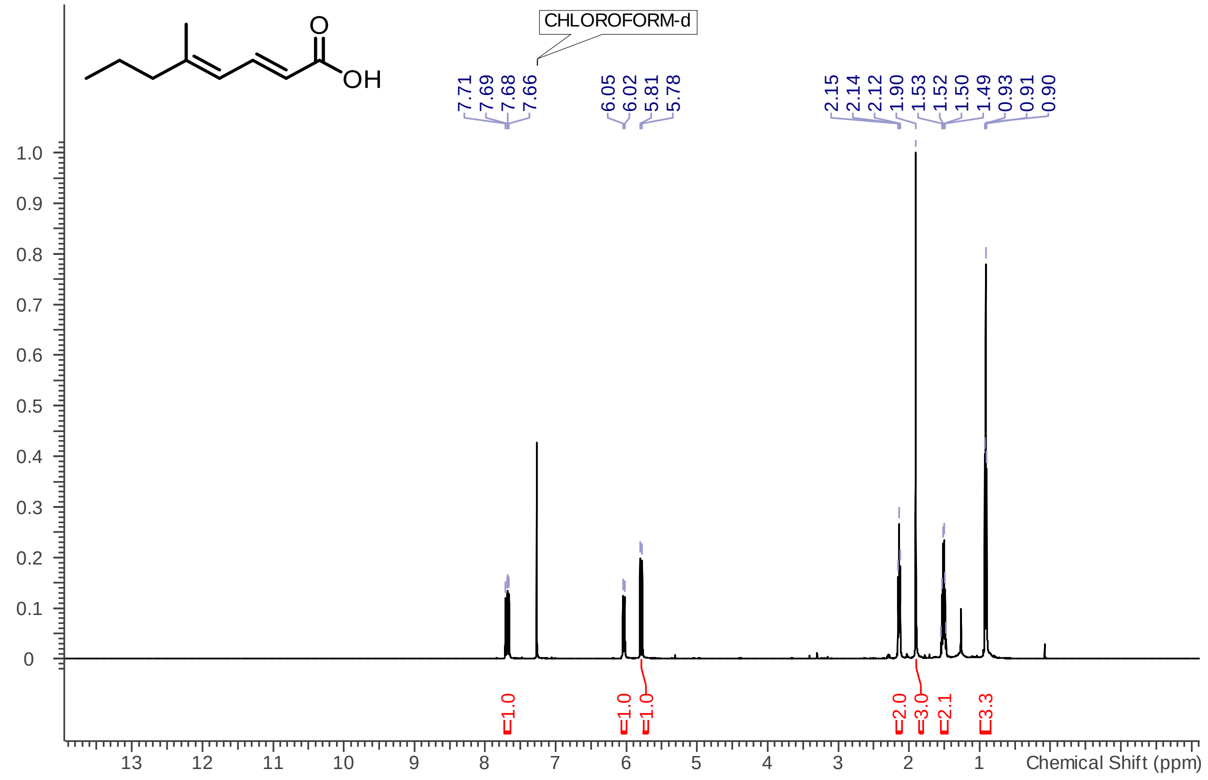

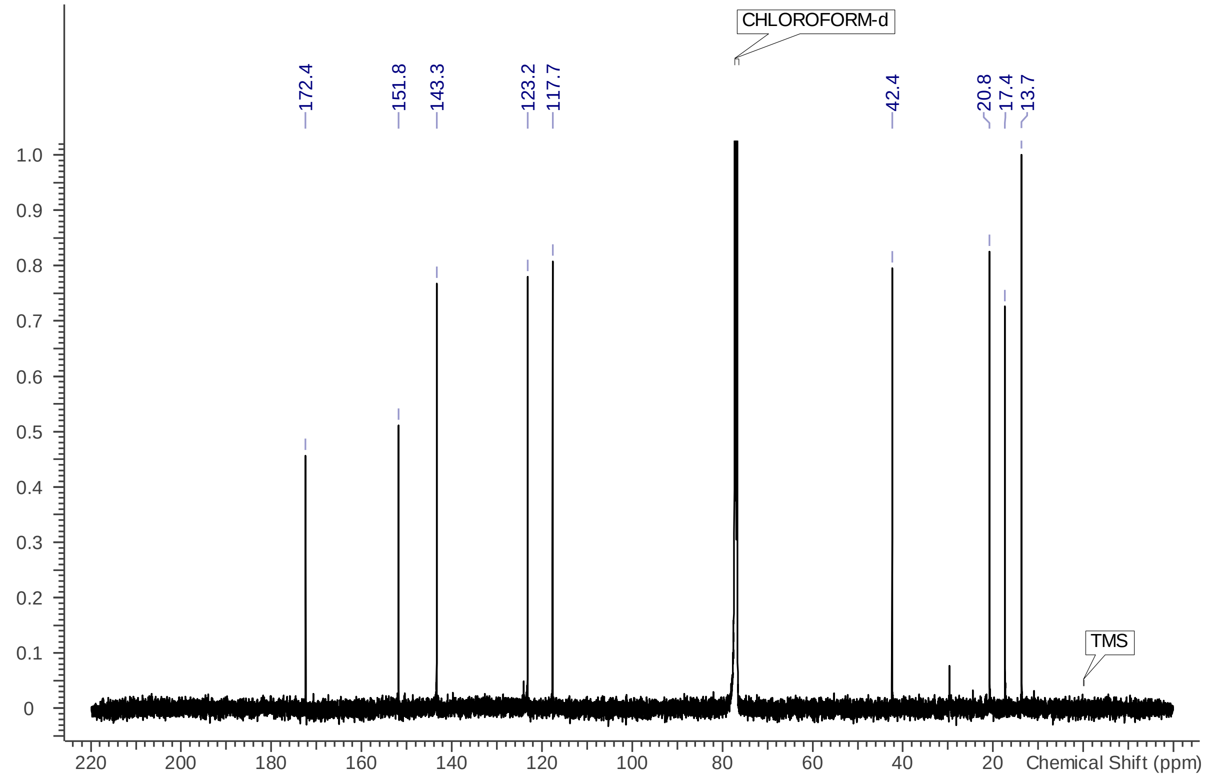

S-(2-Acetamidoethyl) (2*E*,4*E*)-5-methylocta-2,4-dienethioate (46)

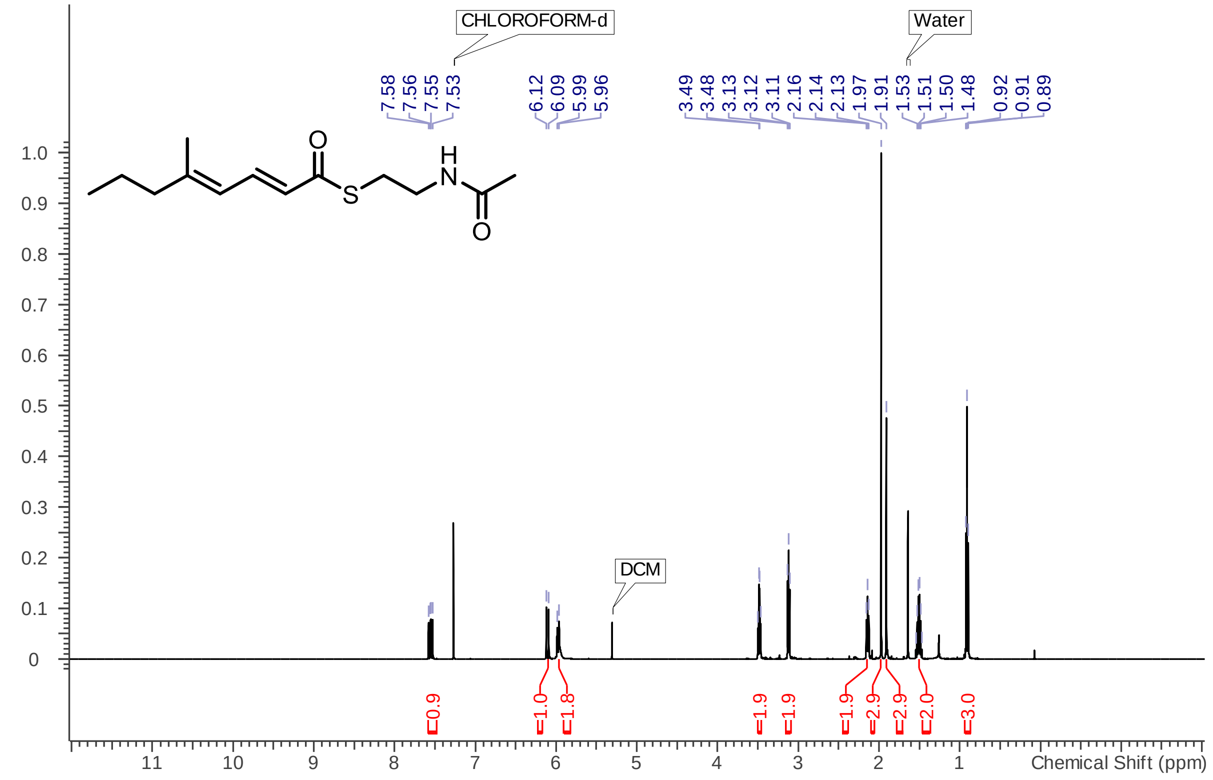

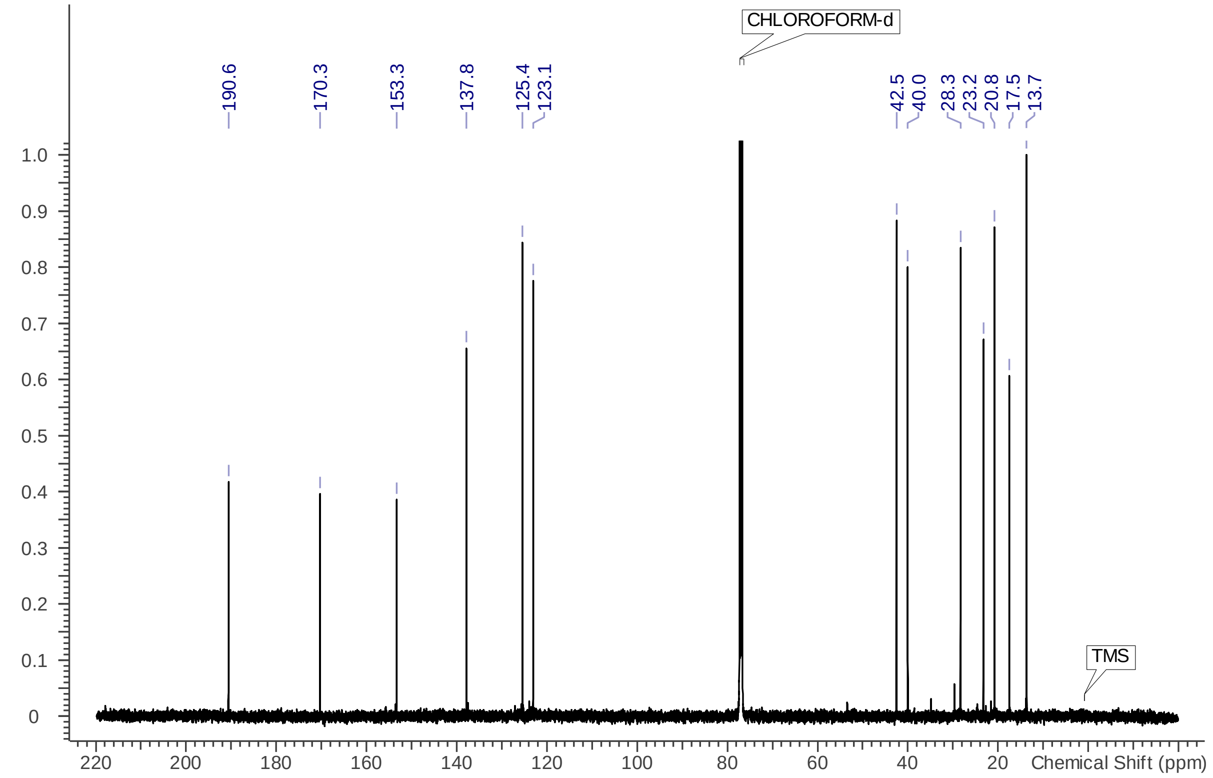

Ethyl (2*E*,4*E*)-octa-2,4-dienoate (47b)

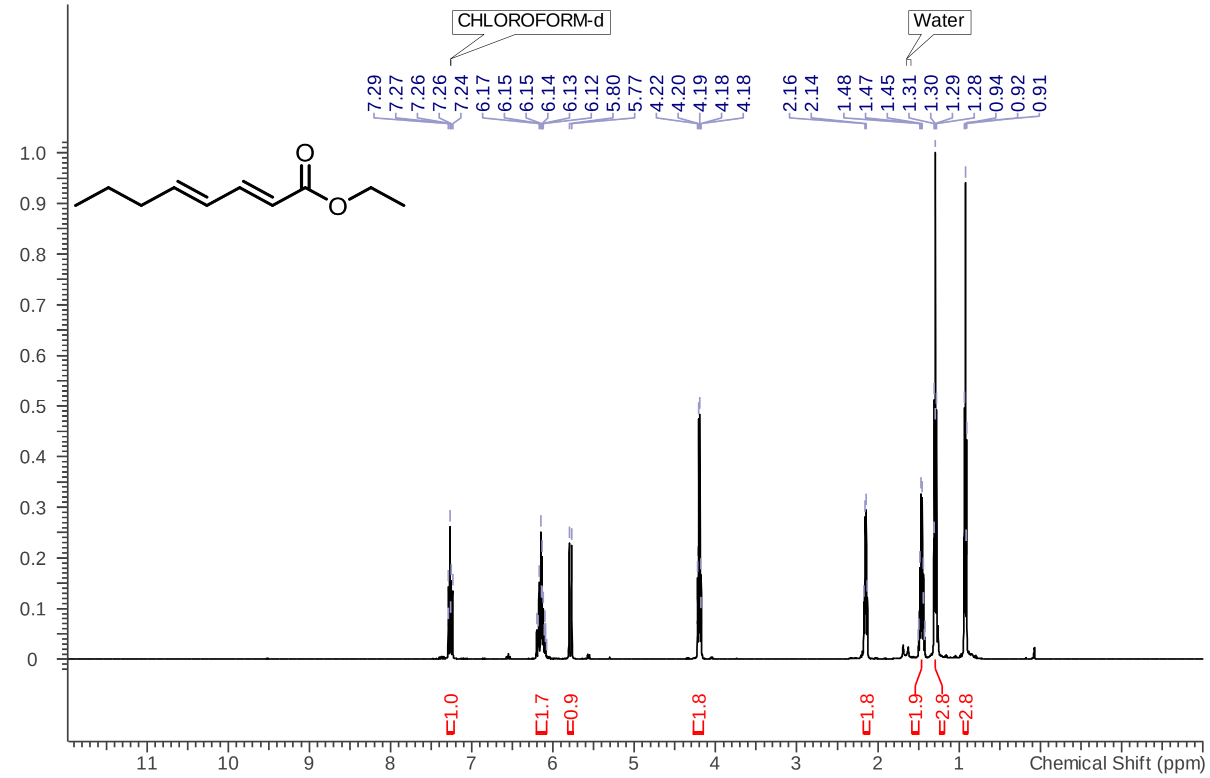

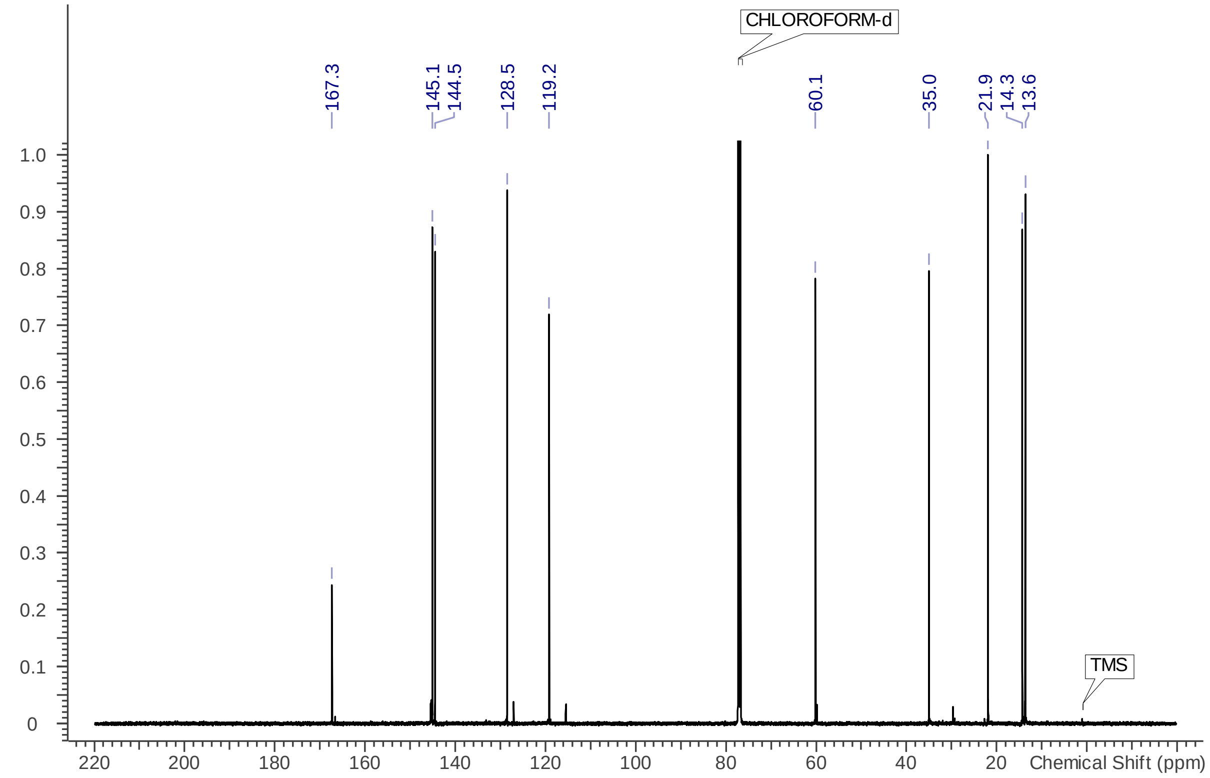

(2*E*,4*E*)-Octa-2,4-dienoic acid (47c)

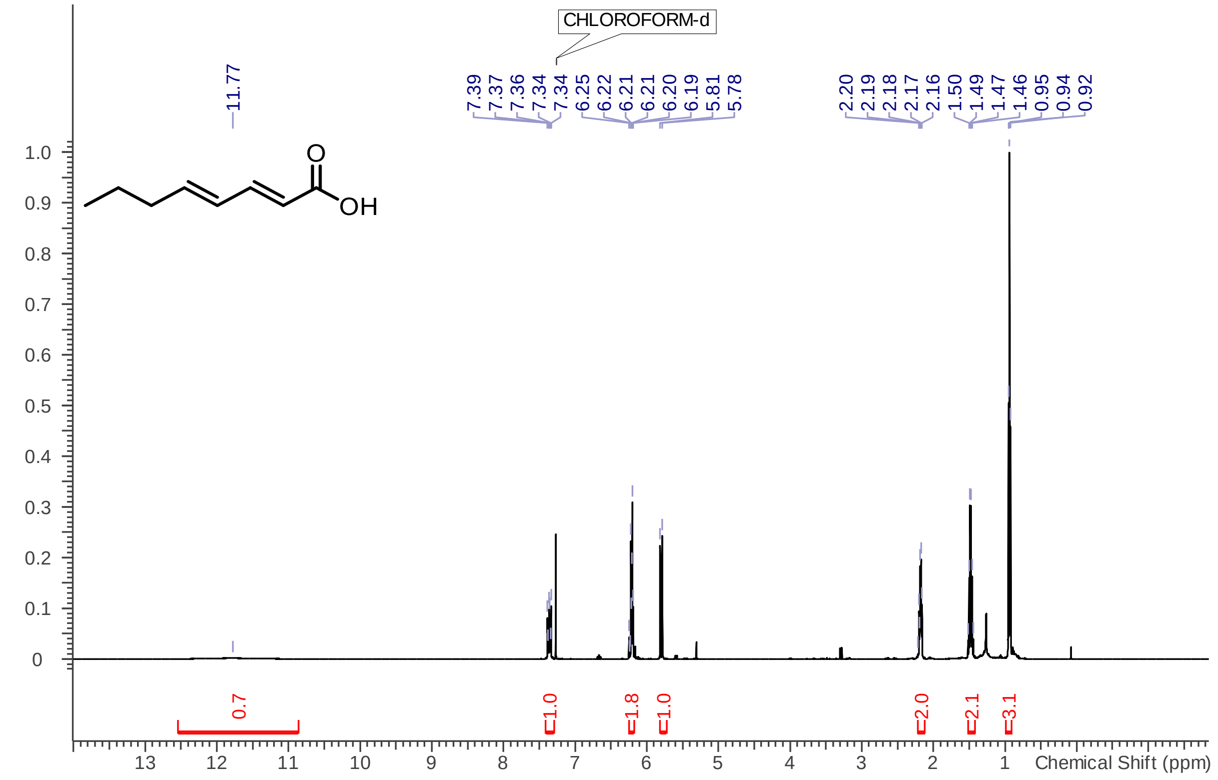

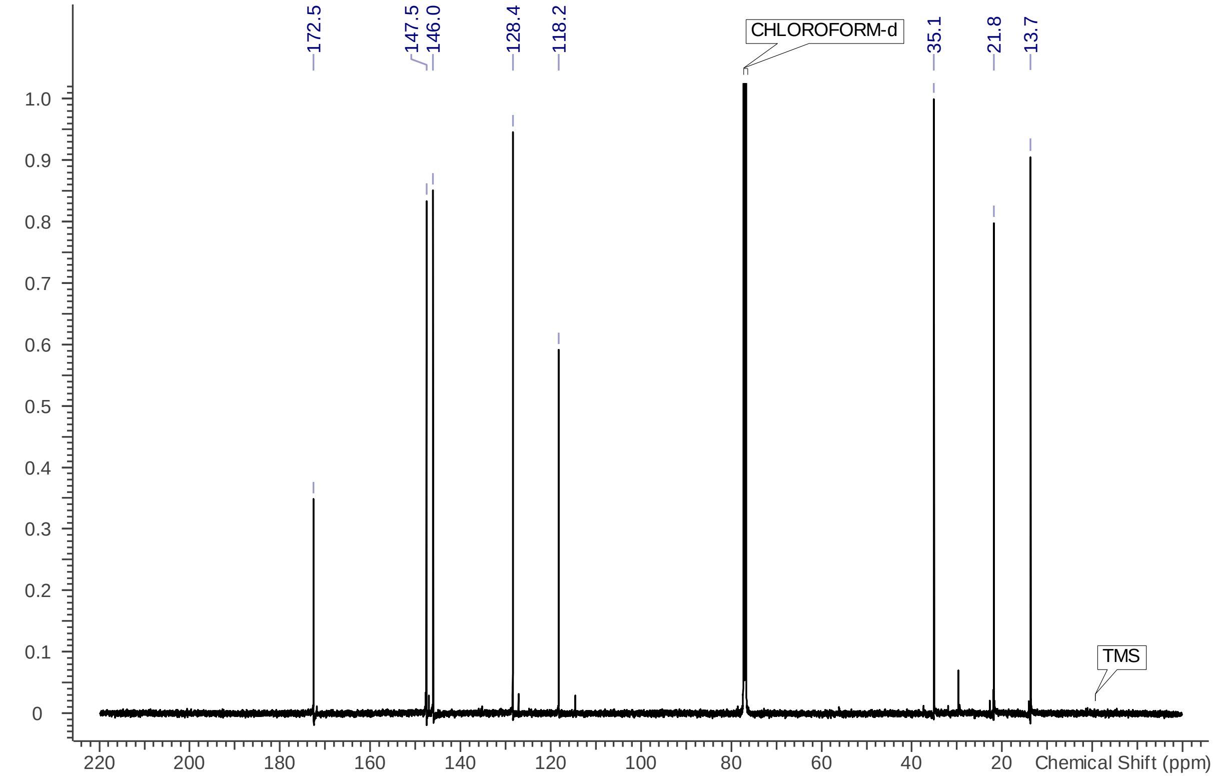

S-(2-Acetamidoethyl) (2E,4E)-octa-2,4-dienethioate (47)

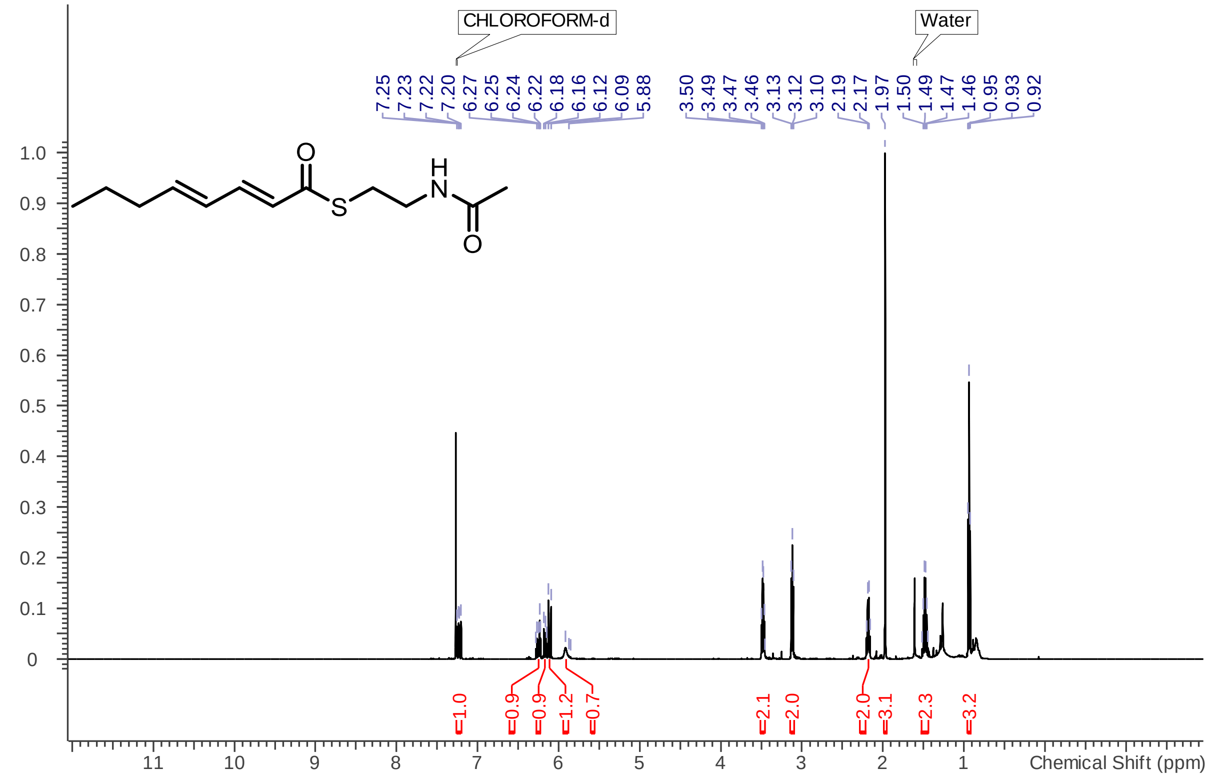

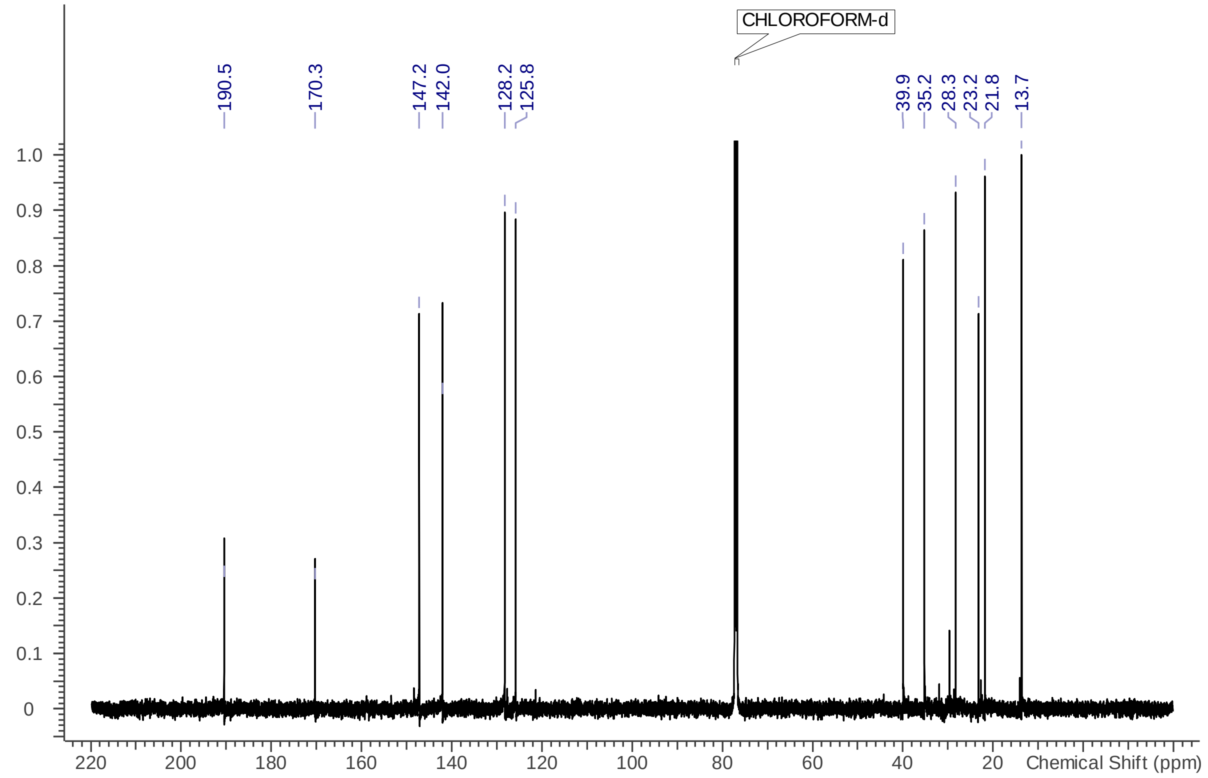

S-Phenyl (2*E*,4*E*)-2,5-dimethylocta-2,4-dienethioate (48)

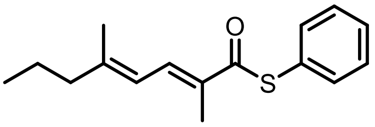

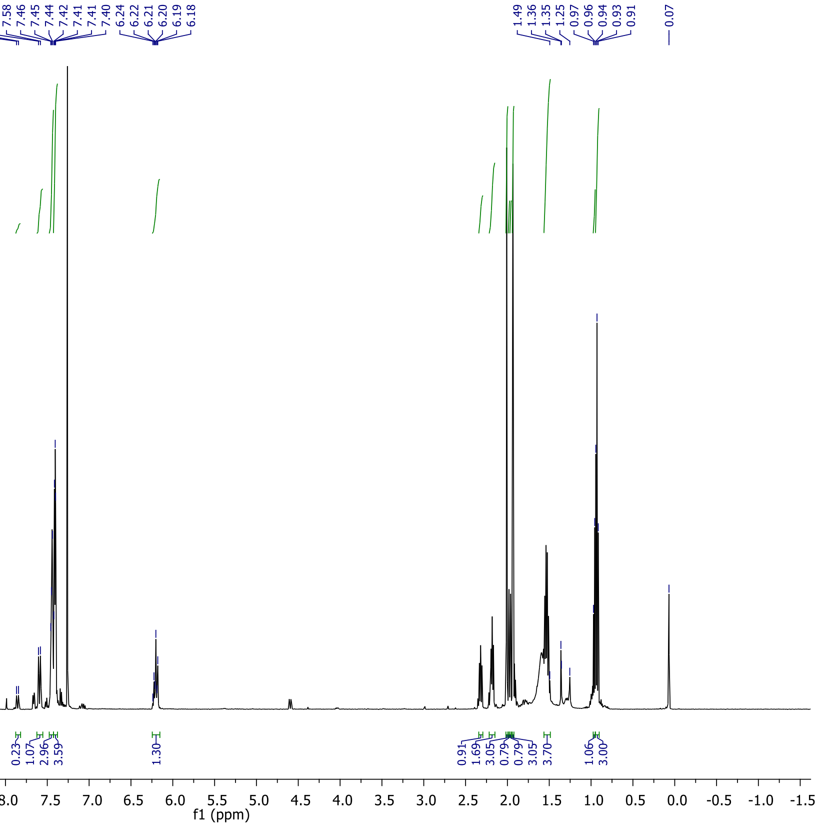

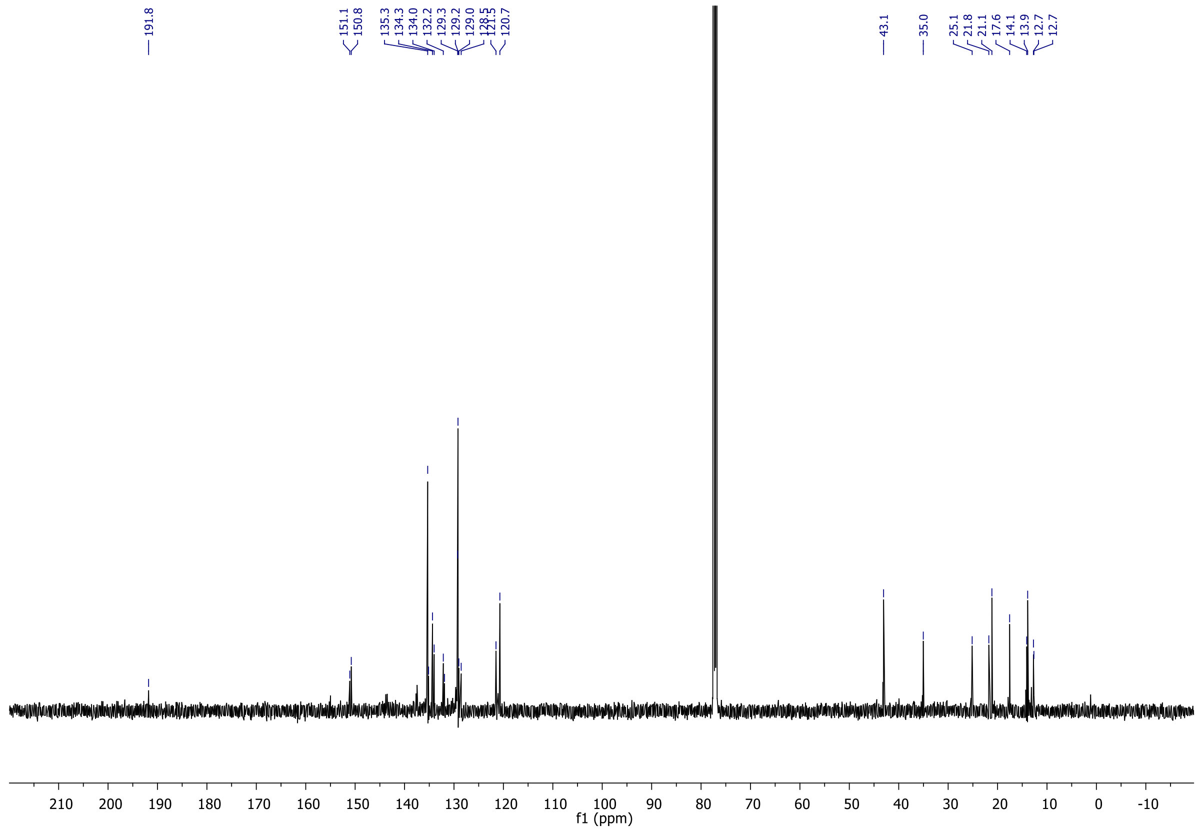

***S*-(2-(3-((*R*)-2,2,5,5-Tetramethyl-1,3-dioxane-4-carboxamido)propanamido)ethyl) (2*E*,4*E*)-2,5-dimethylocta-2,4-dienethioate (49a)**

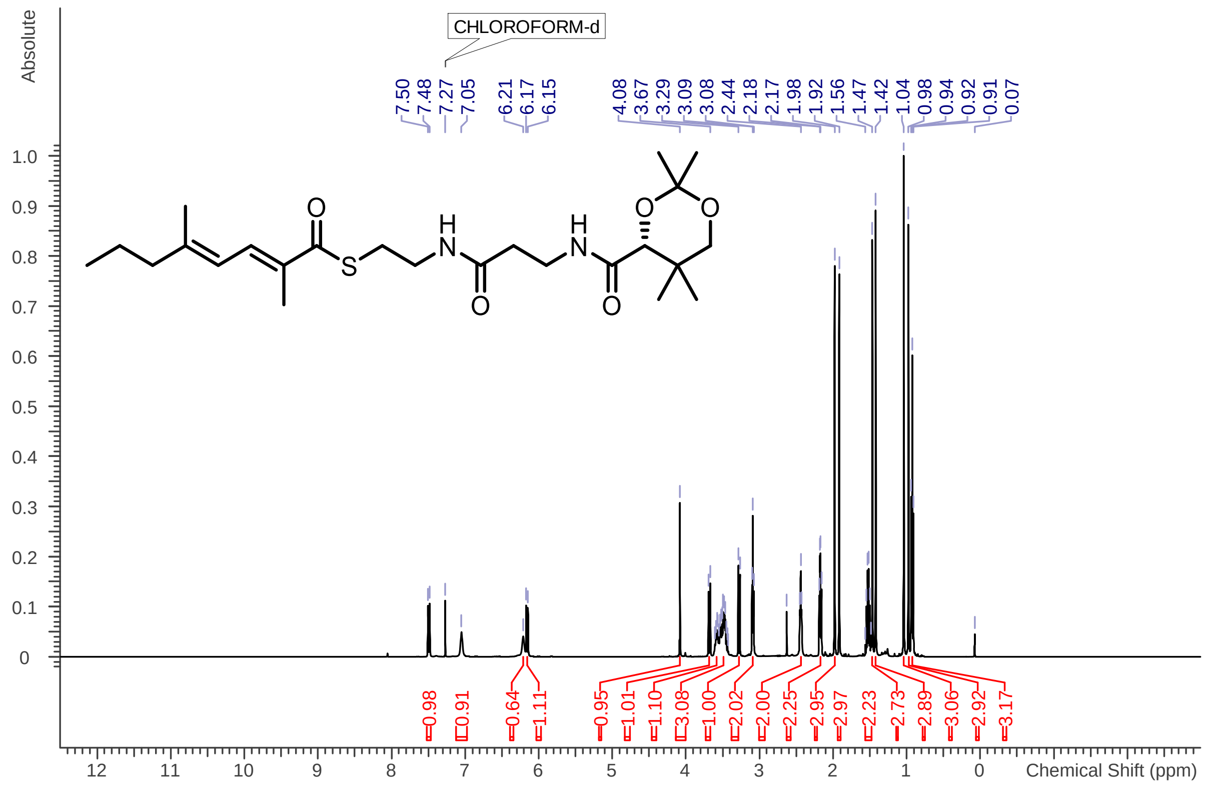

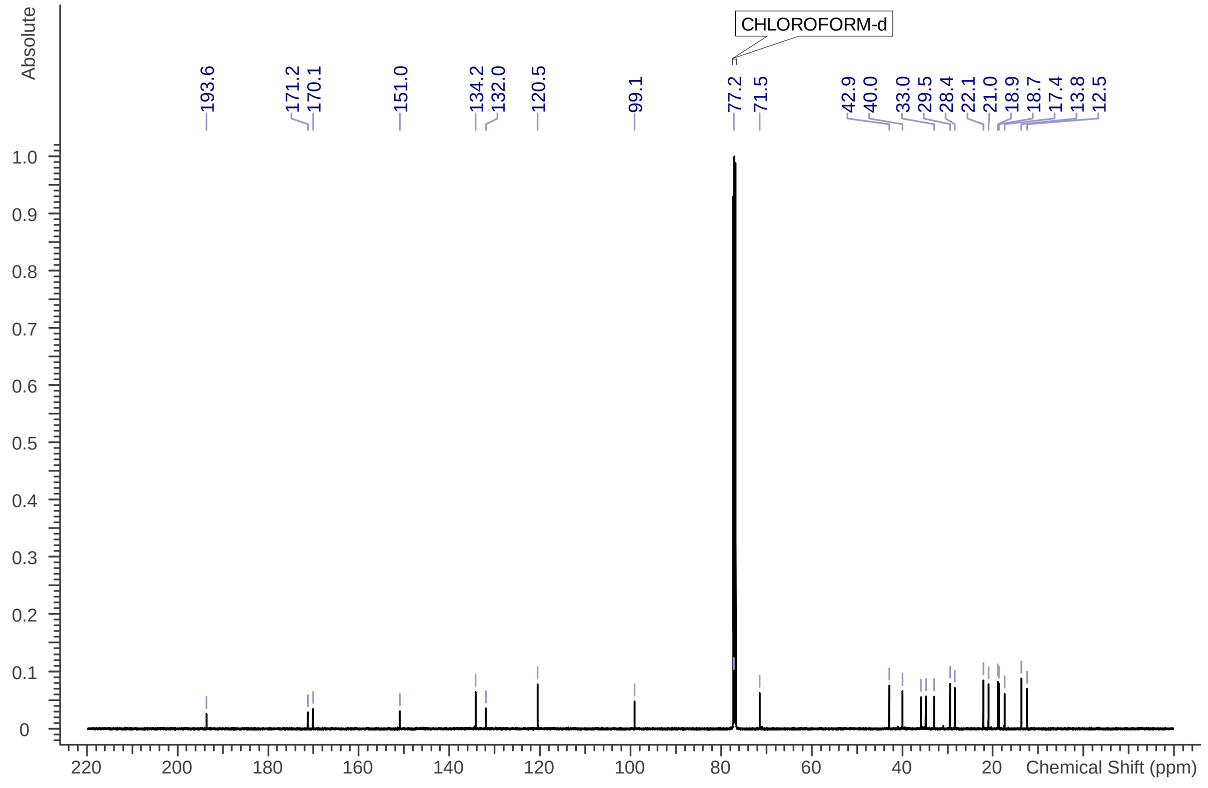

S-(2-(3-((R)-2,4-Dihydroxy-3,3-dimethylbutanamido)propanamido)ethyl) (2E,4E)-2,5-dimethylocta-2,4-dienethioate (49)

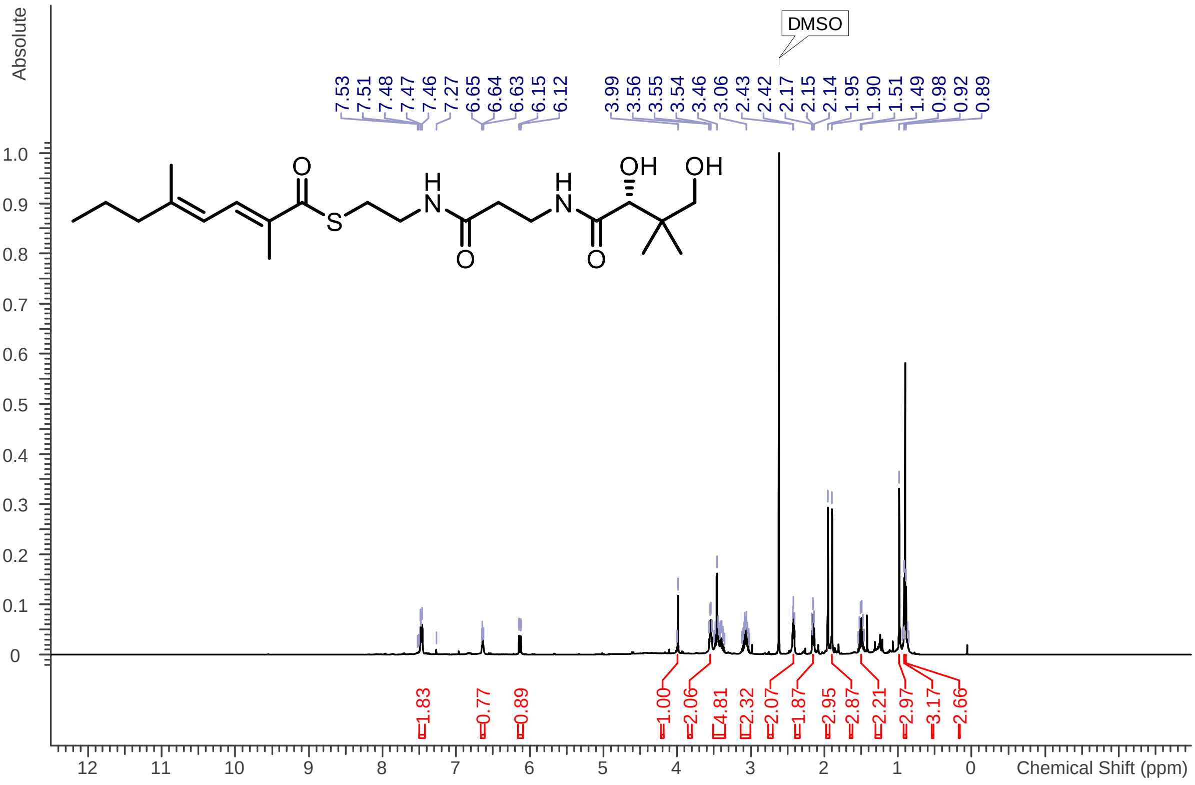

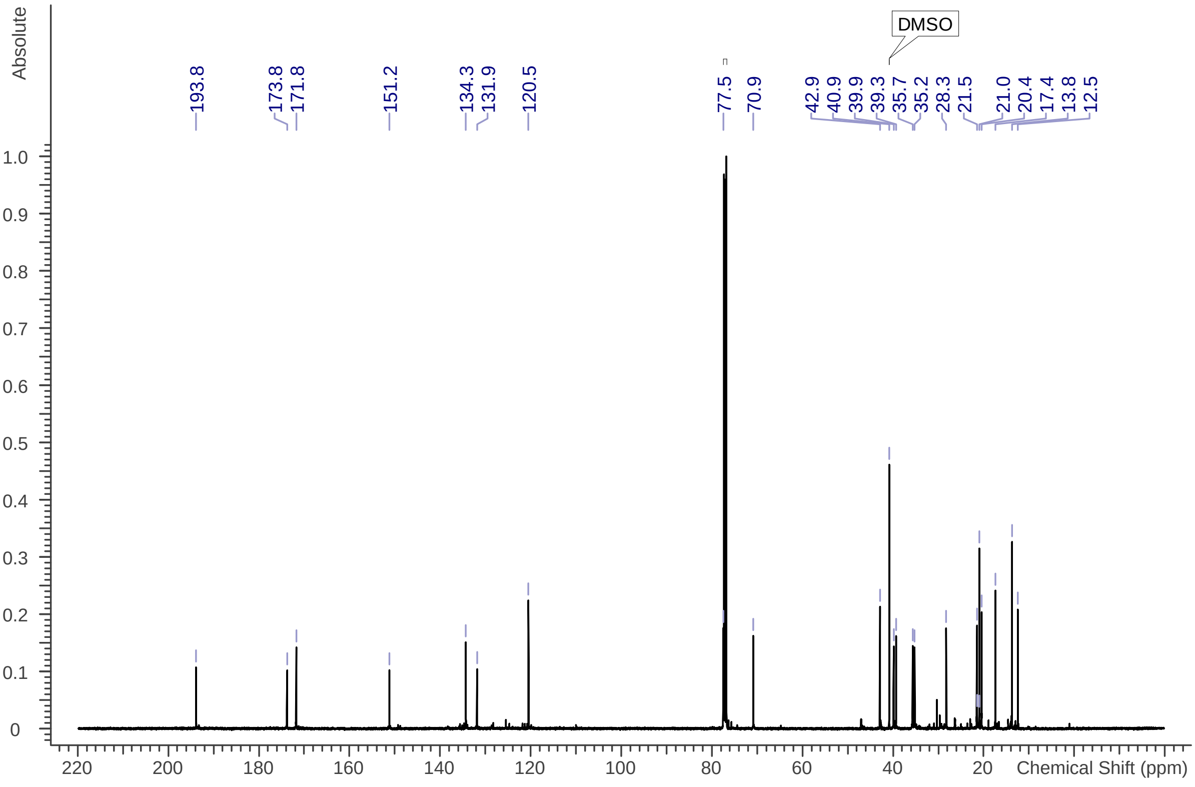

Methyl ((*R*,*E*)-5-(4-hydroxy-2-oxo-3-((2*E*,4*E*)-8,8,8-trifluoro-2,5-dimethylocta-2,4-dienoyl)-2H-pyran-6-yl)hex-1-en-1-yl)carbamate (8)

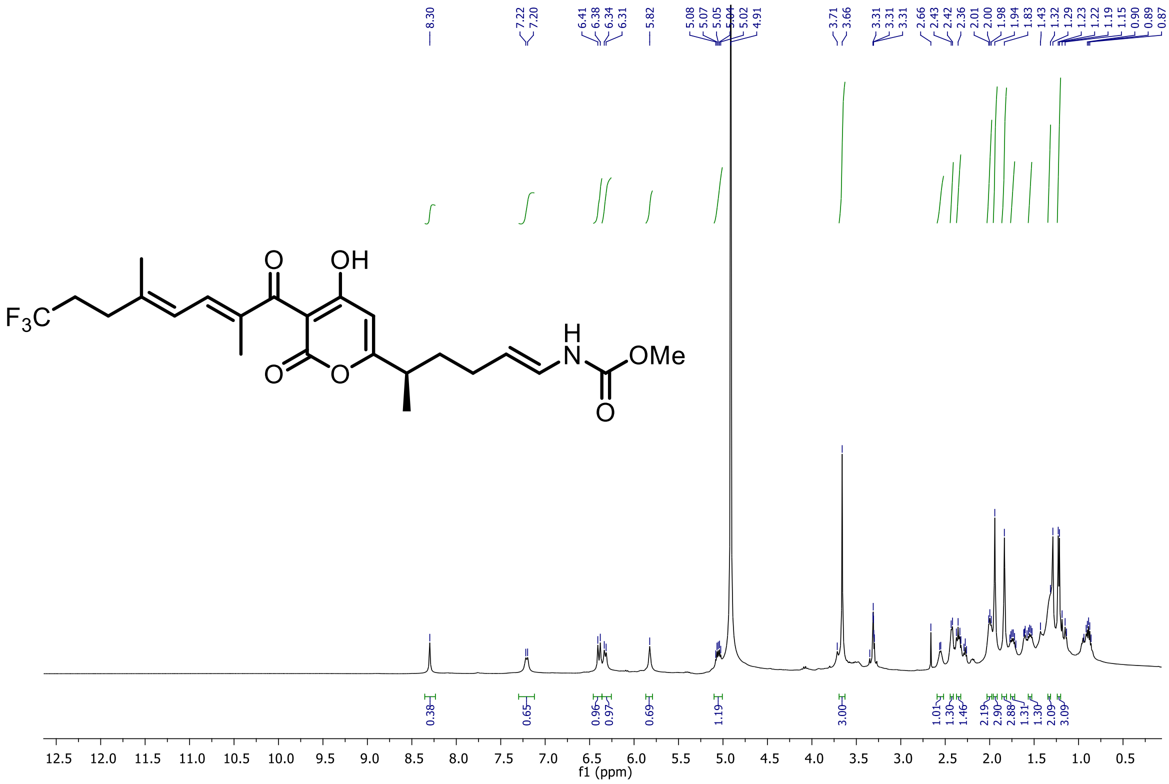

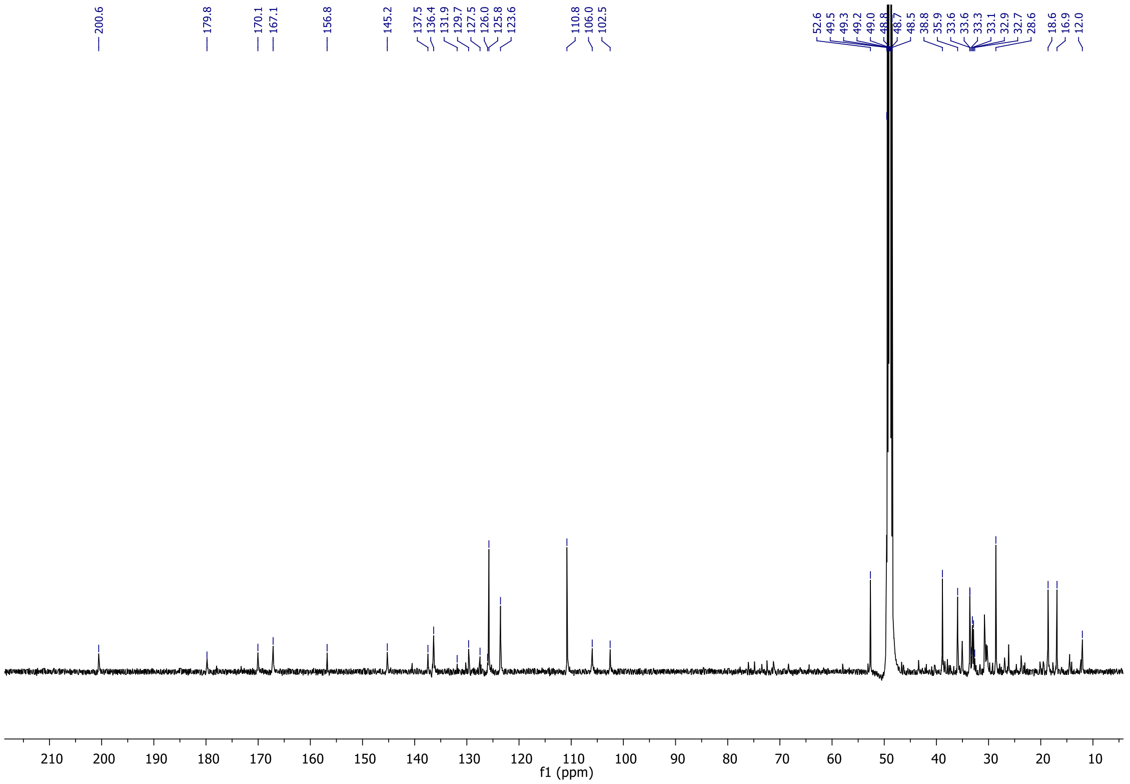

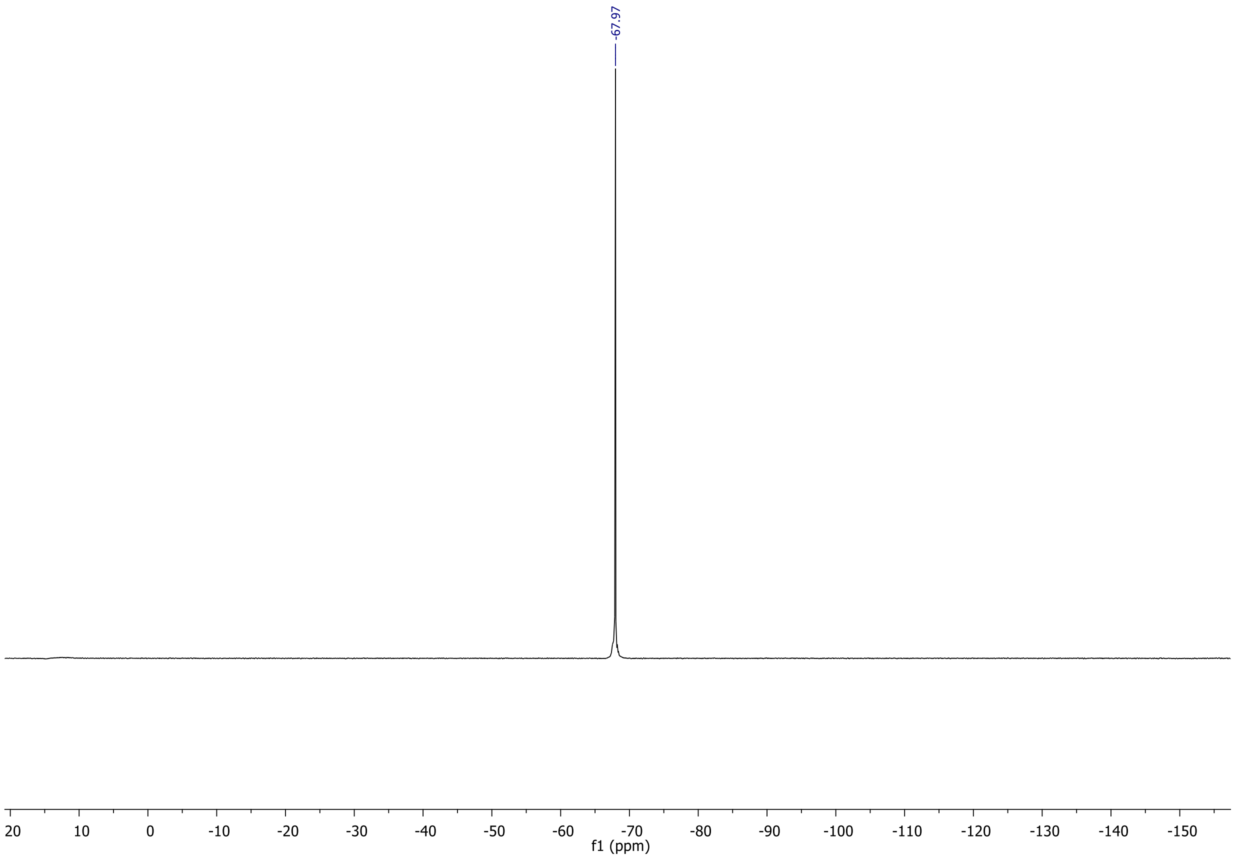

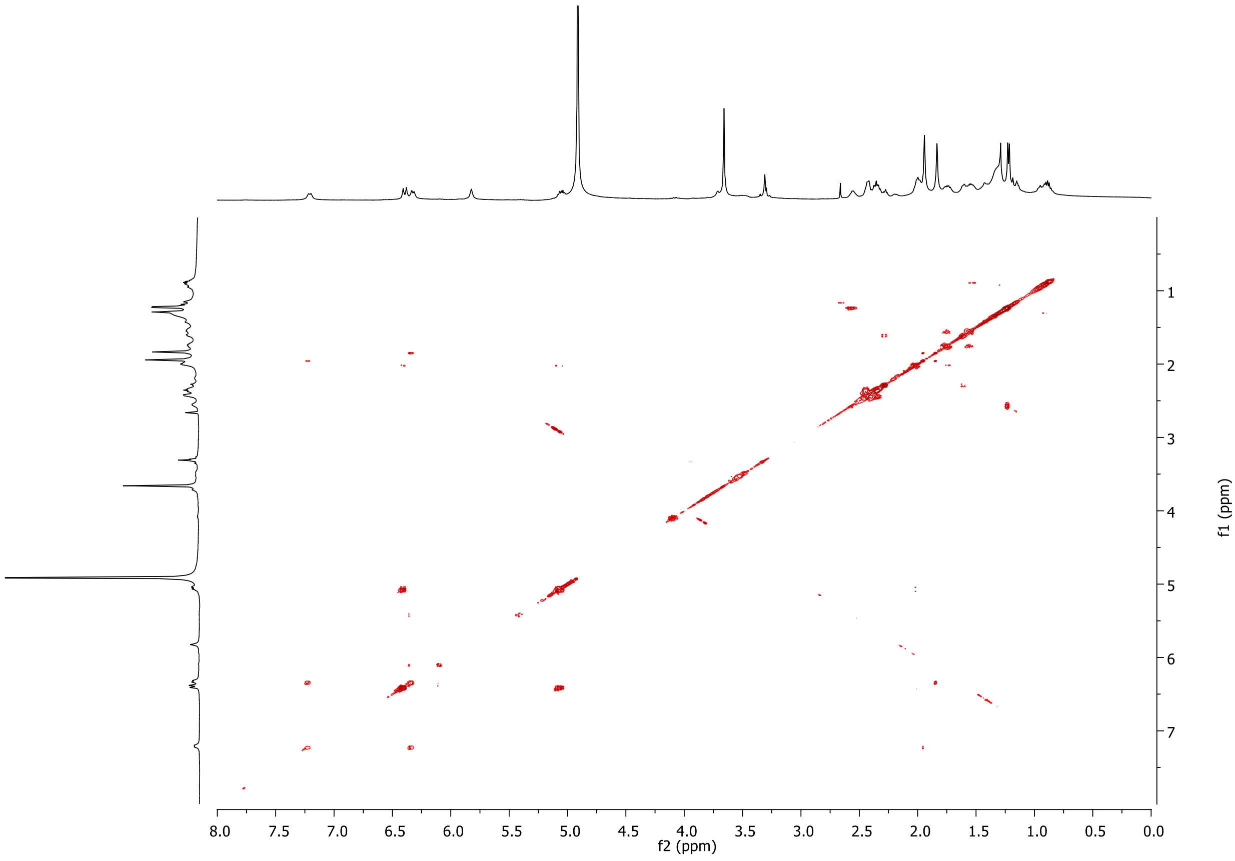

COSY spectrum of **8** in MeOH-*d*$_4$ (500 MHz).

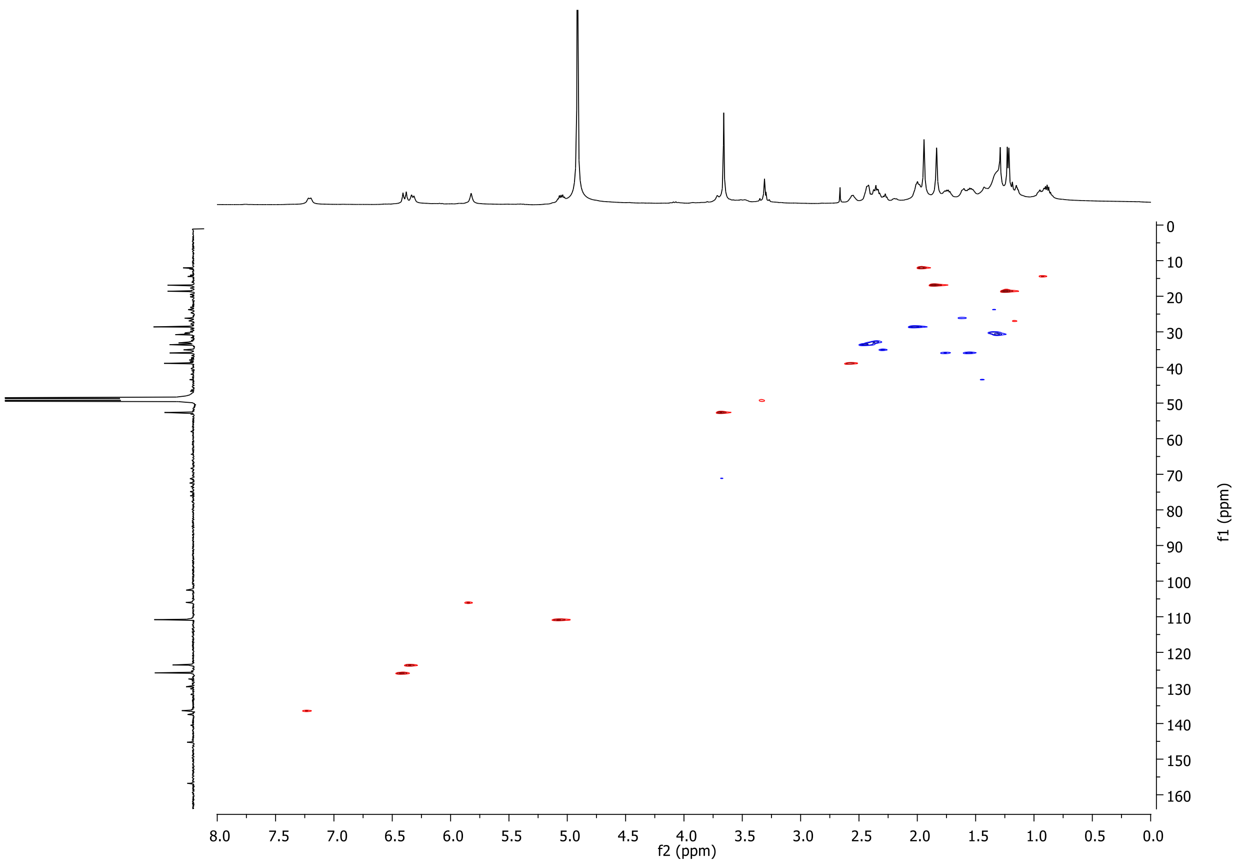

HSQC spectrum of **8** in MeOH-d_4 (500/126 MHz).

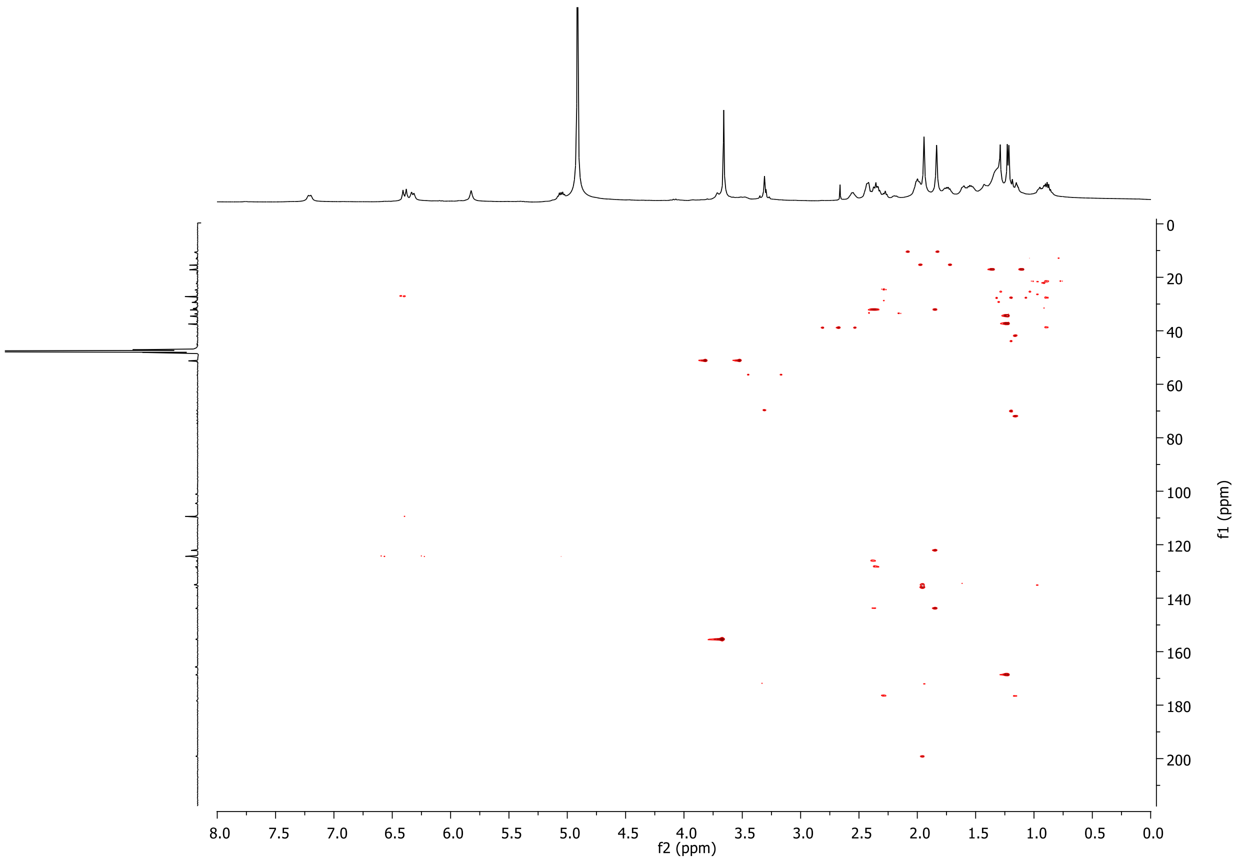

HMBC spectrum of **8** in MeOH-d_4 (500/126 MHz).

References

[1] J. Mukhopadhyay, K. Das, S. Ismail, D. Koppstein, M. Jang, B. Hudson, S. Sarafianos, S. Tuske, J. Patel, R. Jansen et al., *Cell* **2008**, *135*, 295.
[2] *Molecular cloning*. *A laboratory manual*, Cold Spring Harbor Laboratory Press, Cold Spring Harbor, N.Y, **2012**.
[3] Y. Zhang, F. Buchholz, J. P. Muyrers, A. F. Stewart, *Nature genetics* **1998**, *20*, 123.
[4] H. Sucipto, D. Pogorevc, E. Luxenburger, S. C. Wenzel, R. Müller, *Metabolic engineering* **2017**, *44*, 160.
[5] K. Kashefi, P. L. Hartzell, *Molecular microbiology* **1995**, *15*, 483.
[6] D. Pogorevc, F. Panter, C. Schillinger, R. Jansen, S. C. Wenzel, R. Müller, *Metabolic engineering* **2019**, *55*, 201.
[7] A. Rentsch, M. Kalesse, *Angewandte Chemie (International ed. in English)* **2012**, *51*, 11381.
[8] X. Wei, R. J. Taylor, *Tetrahedron Letters* **1998**, *39*, 3815.
[9] J. H. Sahner, H. Sucipto, S. C. Wenzel, M. Groh, R. W. Hartmann, R. Müller, *Chembiochem : a European journal of chemical biology* **2015**, *16*, 946.
[10] I. R. G. Thistlethwaite, F. M. Bull, C. Cui, P. D. Walker, S.-S. Gao, L. Wang, Z. Song, J. Masschelein, R. Lavigne, M. P. Crump et al., *Chemical science* **2017**, *8*, 6196.
[11] M. Simon, K. Karaghiosoff, P. Knochel, *Organic letters* **2018**, *20*, 3518.
[12] *Catalogue of Mutations in Mycobacterium Tuberculosis Complex and Their Association with Drug Resistance*, World Health Organization, Geneva, **2021**.
[13] Q. Wang, H. I. M. Boshoff, *Methods in molecular biology (Clifton, N.J.)* **2021**, *2314*, 595.
